# Copy number variation-based genome wide association study reveals additional variants contributing to meat quality in Swine

**DOI:** 10.1038/srep12535

**Published:** 2015-08-03

**Authors:** Ligang Wang, Lingyang Xu, Xin Liu, Tian Zhang, Na Li, El Hamidi Hay, Yuebo Zhang, Hua Yan, Kebin Zhao, George E Liu, Longchao Zhang, Lixian Wang

**Affiliations:** 1 Key Laboratory of Farm Animal Genetic Resources and Germplasm Innovation of Ministry of Agriculture of China, Institute of Animal Science, Chinese Academy of Agricultural Sciences, Beijing 100193, China; 2 Animal Genomics and Improvement Laboratory, BARC, USDA-ARS, Beltsville, Maryland 20705, USA; 3 Jilin Academy of Agricultural Sciences, Changchun 130033, China

## Abstract

Pork quality is important both to the meat processing industry and consumers’ purchasing attitude. Copy number variation (CNV) is a burgeoning kind of variants that may influence meat quality. In this study, a genome-wide association study (GWAS) was performed between CNVs and meat quality traits in swine. After false discovery rate (FDR) correction, a total of 8 CNVs on 6 chromosomes were identified to be significantly associated with at least one meat quality trait. All of the 8 CNVs were verified by next generation sequencing and six of them were verified by qPCR. Only the haplotype block containing CNV12 is adjacent to significant SNPs associated with meat quality, suggesting the effects of those CNVs were not likely captured by tag SNPs. The DNA dosage and EST expression of CNV12, which overlap with an obesity related gene Netrin-1 (*Ntn1*), were consistent with *Ntn1* RNA expression, suggesting the CNV12 might be involved in the expression regulation of *Ntn1* and finally influence meat quality. We concluded that CNVs may contribute to the genetic variations of meat quality beyond SNPs, and several candidate CNVs were worth further exploration.

As a global food source, pork has unquestionable significant implications on the livelihood of human beings. Pork quality is important to the meat processing industry and consumers’ purchasing attitude^1^, thus, it is one of the main selection objective in breeding plans for most of the pig breeding organizations and enterprises^2^. Meat quality traits are complex and usually influenced by multiple genes or QTLs, therefore, the genetic improvement of these traits is rather slow. However, genome-wide association studies (GWAS), linkage mapping, and selective sweep analysis have been successfully employed to investigate significant gene markers for pH, tenderness, meat color, intramuscular fat (IMF) content^1^,^2^,^3^,^4^,^5^,^6^,^7^,^8^,^9^.

To date, a total of 12,618 QTLs from 461 publications have been recorded in PigQTLdb (http://www.animalgenome.org/cgi-bin/QTLdb/SS/index, released at Feb 11, 2015)^10^. Among these QTLs, more than half (7,014) were associated with meat and carcass quality traits. However, the limited density of microsatellite (SSR) markers resulted in inaccurate QTL mapping (Some segments are larger than 20 cM^11^, even cover a whole chromosome^12^). Long-term fine mapping experiments are needed to refine their locations and investigate causative variants^13^. Additionally, although genome wide association studies identified significant SNPs associated with meat quality traits, these SNPs explain a small portion of the genetic variance^2^,^4^. Alternative variances, which could explain the “missing heritability” of meat quality traits, were urgently needed^14^.

Copy number variations (CNVs) are currently accepted as a common source of genetic variation, and the “missing heritability” could partially be explained by CNVs as reported in several human studies^15^,^16^,^17^,^18^,^19^. In animals, there is strong evidence of the effects CNVs may have on disease resistance and economically important traits, such as milk production^13^, gastrointestinal nematodes resistance^20^, residual feed intake^21^, Marek’s disease-resistance^22^, late-feathering^23^, and squamous cell carcinoma of the digit^24^. Min pig is well known in China for its excellent meat quality (with 5% IMF in LM at 240-day-old) and good cold resistance capacity, and Large-white pig is a normal lean meat type breed with normal meat quality. The intercross of Min × Large-white is a good model to analyze the genetics of meat quality^2^. The objective of this work was to perform systematic CNV association analysis with meat quality traits using the Porcine SNP60 Genotyping BeadChip (Illumina, San Diego, CA, USA), analyze the joint or independent effects of CNVs and SNPs, and provide some helpful information to identify genetic markers that may be suitable for inclusion in genetic improvement program.

## Results

### Trait properties and correlations

Two IMF traits (IMF content and marbling), 6 meat color traits (color L6*, color a6*, color b6*, color L24*, color a24*, and color b24*), 2 pH traits (pH24 and pH6), moisture, and shearing force (SFN) were analyzed in this paper. The summary of means, standard deviations (SD), genetic and phenotype correlations of the traits are provided in Table 1. From the result, there was a small difference between genetic and phenotype correlations. As respect, the genetic correlations between marbling and IMF, between each trait for meat color, between 2 pH values were high. The correlations between different kinds of traits such as color with IMF, pH with IMF, were low except the moisture and IMF.

### CNV segmentation and genotyping

In the 678 samples of the three-generation pigs, a total of 32,544 distinct segments were detected using the multivariate method of CNAM in SVS. After merging across samples, 48 nonredundant CNVs were left for subsequent association test (Table S1). Within these 48 segments, each sample was genotyped (i.e., called as a loss, neutral or gain event) according to a three state model with strict threshold levels of marker mean ±0.5. Since the multivariate CNAM method was developed to identify common CNVs, only those segments with frequencies above 0.4% were retained for further analysis in order to filter away false positive calls. A total of 15 CNVs ranging in size from 34,076 bp to 1.10 Mb were retained (frequency > 0.04) (Table 2). These 15 CNVs have an estimated average size and SNP count of 208.53 Kb and 3.27 Mb, respectively.

### Quality assessment of CNVs by using qPCR and NGS data

We first compared all of the 15 CNVs with the results from 7 previously published reports^25^,^26^,^27^,^28^,^29^,^30^,^31^. Twelve out the 15 CNVs (80%) were found previously reported (Table 2 and Table S2), and the remaining 3 non-overlapping CNVs (CNV2, 6 and 13) had high frequencies (ranging from 26 to 83%) and large marker mean changes (ranging from −0.39 to −1.24, deviated from 0), suggesting they are probably real CNVs (Table 2). Two complementary methods, quantitative PCR (qPCR) and next generation sequencing (NGS), were also performed to confirm the existence of detected CNVs. In the qPCR validation, we systematically assessed the overall agreement rate of detected CNVs with qPCR results. All the primers and results of qPCR are listed in Table S3 and Table S4. Totally, the detection power for the qPCR validation is 73.3% (11/15). In the NGS validation, we chose 12 F0 pigs for CNV calling, the sequencing depth of coverage for each animal varied from 4.7× to 8.4×. All of the NGS-based CNVs which were overlapped with significantly associated CNVs are listed in Table S5. All of the CNVs were confirmed with the Read depth (RD) method ranged from 0 to 4.53^32^. Detailed NGS analyses will be presented in a separate manuscript.

### CNV association analysis

**Figure 1:**
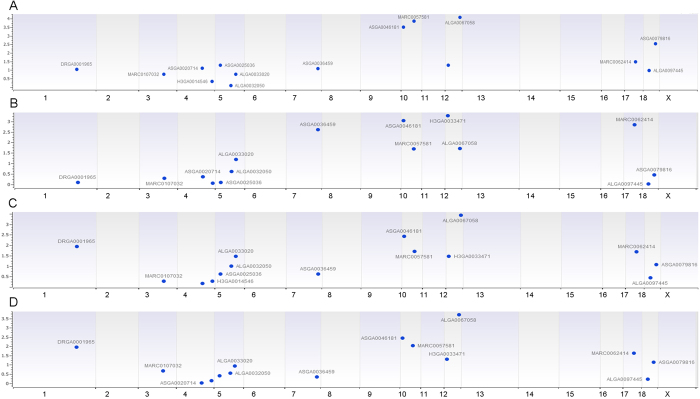
Manhattan plots of associated CNVs for meat quality traits using linear regression model. (**A**) pH value at 6 h postmortem, (**B**) color b* at 24 h postmortem, (**C**) marbling, and (**D**) intramuscular fat. Negative log10-transformed P values from a genome-wide scan are plotted against genomic coordinates on 18 autosomal chromosomes.

All of the 602 F2 generation pigs were employed to test association between CNVs and rEBV data. We identified a total of 8 CNVs that were significantly associated with at least one trait using a linear regression model (Fig. 1 & S1 and Table 2). Among those 8 associated CNVs, the CNV with the highest frequency (87.46%) was found at chr1: 242,457,549–242,519,391, while the CNV with the lowest frequency (35.10%) was localized at chr18: 46,776,812–46,983,072 (Table 2 and Table S6). Among the 12 traits, only four traits (b24*, marbling, ph6 and IMF) had significantly associated CNVs. Two CNVs: CNV9 (chr10: 9,369,752–9,462,206), and CNV12 (chr12: 56,893,678–57,020,468) had p values < 0.05 after FDR correction for more than two traits. Their frequencies were 46.76% and 79.35%, respectively.

### Annotation of QTLs and genes within and near CNVs

QTL locations on the updated porcine genome sequence assembly (Sscrofa10.2) were retrieved from the PigQTLdb to compare our 8 associated CNVs with previously reported QTL locations. When considered together, 3 of the 8 significant CNVs overlapped with at least one of the known QTLs for meat quality (Table S7). For CNV1 (chr1: 242,457,549–242,519,391), which was significantly associated with IMF, two QTLs for percentage type I fibers (QTL 2794)^33^ and diameter of type IIb muscle fibers (QTL 2795)^33^ were overlapped. For CNV10 (chr10: 49,173,528–49,255,139), which was significantly associated with pH6 and IMF, one QTL for Marbling (QTL 3280)^34^ was overlapped. For CNV12 (chr12: 56,893,678–57,020,468), which was significantly associated with pH6, IMF and Marbling, three QTLs for CIE- color a* (QTL 21403)^35^ and Percentage type IIb fibers (QTL 7036 and 7021)^36^ were overlapped. The CNV9 (chr10: 9,369,752–9,462,206) was also near the previously reported meat quality QTL regions (pH 24 hr post mortem (ham QTL 18702)^9^, pH 24 h post mortem (ham QTL 18681)^9^ and CIE-color a* (QTL 3067)^37^).

Based on the Sscrofa10.2 sequence assembly, pig gene annotations within the 8 CNVs and flanking regions (500 Kb in both downstream and upstream directions) were summarized in Table S8. Among the 80 genes retrieved from the 8 regions, 22 cases were uncharacterized protein coding genes, 7 cases were RNA coding genes, and 51 cases were known protein-coding genes. Since only a limited number of genes in the pig genome have been annotated, we converted the pig official gene symbols to orthologous human genes by BioMart before Gene Ontology (GO) and pathway analysis. Ten statistically significant GO terms (P < 0.05, Table S9) and none significant Kyoto Encyclopedia of Genes and Genomes (KEGG) pathways were identified. A total of 12 detected genes were associated with GO terms consisting of secretion by cell, G-protein signaling, coupled to cAMP nucleotide second messenger, cAMP-mediated signaling, secretion, G-protein signaling, coupled to cyclic nucleotide second messenger, cyclic-nucleotide-mediated signaling, neuromuscular junction development, positive regulation of peptide secretion, positive regulation of cell proliferation, cell death, and death.

All of the 51 protein coding genes were searched on GenBank for their other functional information. *NOD1* (the nucleotide-binding oligomerization domain-containing protein 1 gene) was found overlapped with CNV15 (chr18: 46,776,812–46,983,072), and was expressed increasingly in the adipose tissue of women with gestational diabetes^38^. Approximately 25 Kb downstream of the same CNV, pleckstrin homology domain containing, family A (phosphoinositide binding specific) member 8 gene (*PLEKHA8*) encodes the protein of the four-phosphate adaptor protein 2 (FAPP2) which could connect vesicular transport with lipid synthesis^39^. Netrin-1 (*Ntn1*) gene which covers CNV12 (chr12: 56,893,678-57,020,468), has been reported to be highly expressed in obese but not lean adipose tissue of humans and mice^40^.

### SNP association analysis

The result of GWAS based on SNPs was shown in Table S10. A total of 24 SNPs were genome-wide significantly associated with IMF, Marbling, Moisture, SFN, Ph6, a6* and a24*. Among the 24 significant SNPs (Table 3 and Fig. S2), 18 SNPs were located on Chromosome 12, one SNP located on Chromosomes 2 and 3 respectively. Eight SNPs were significantly associated with more than one trait. There were many overlapping significant SNPs for IMF and moisture (5/5 of moisture) and for IMF and Marbling (8/10 of IMF), which was in concordance with the results of significant associated CNVs.

### Relationship between associated CNVs and associated SNPs

**Figure 2:**
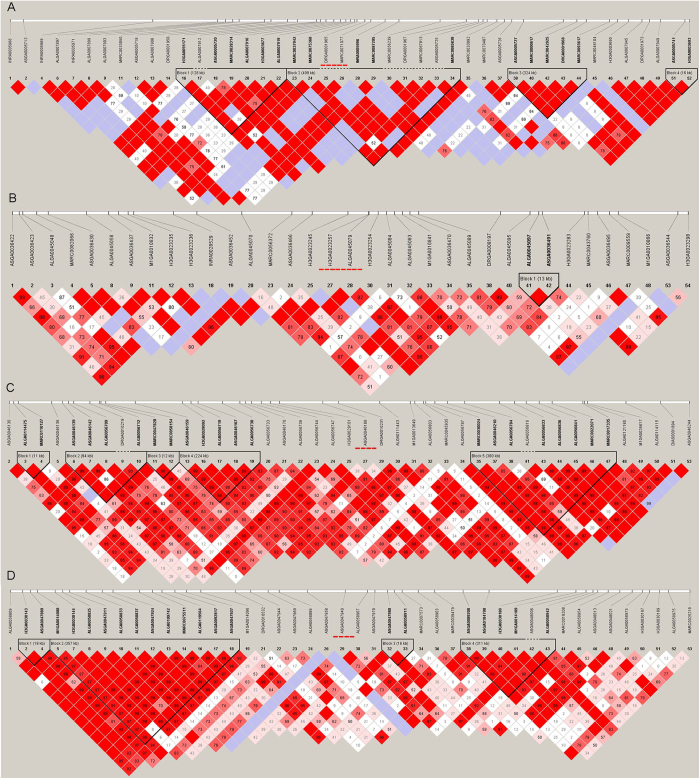
Haplotype analysis of CNV1, CNV8, CNV9, and CNV10. (**A**) CNV1, (**B**) CNV8, (**C**) CNV9, and (**D**) CNV10. Black bar represents CNV and * represents significant tag SNPs.

**Figure 3:**
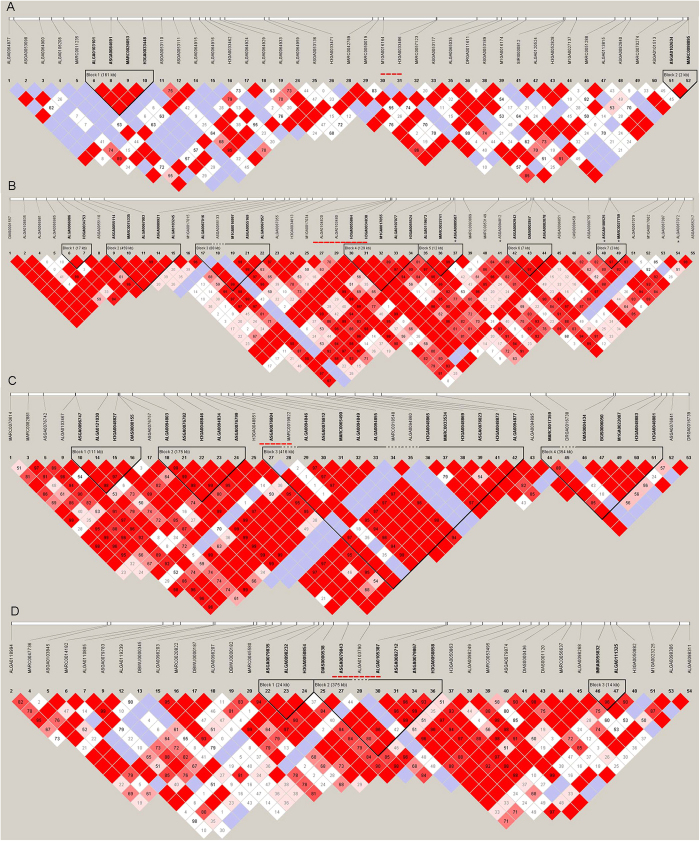
Haplotype analysis of CNV11, CNV12, CNV13, and CNV15. (**A**) CNV11, (**B**) CNV12, (**C**) CNV13, and (**D**) CNV15. Black bar represents CNV and * represents significant tag SNPs.

For all of the 8 possible combinations of CNVs and traits, we found no significant SNPs directly overlapping with them. Haplotype analysis for the CNV regions which included 25 SNPs both downstream and upstream of associated CNVs were used to detected the linkage relationship between CNVs and neighboring SNPs. And the results (Figs 2 and 3) showed four cases (CNV1, CNV12, CNV13, and CNV15) were enclosed in a haplotype block with other SNPs, none cases where CNVs directly overlapped with significantly associated SNPs, and only the block containing CNV12 is adjacent to IMF and marbling significantly associated SNPs (about downstreams of 300 kb).

## Discussions

In conventional CNV discovery studies, researchers usually try to identify as many CNV regions as possible. But in this CNV-based GWAS, the algorithm intended to identify the common CNVs shared among samples in order to detect associations with meat quality traits. Thus, only 48 CNVs were detected and only 15 CNVs were retained after quality control. However, the validation results indicate that all the 15 CNVs may be real. Our results indicated that there is a small discrepancy (27.6%) between qPCR and SVS 8.2 CNV callings. As we know, small variations such as SNPs, small indels may influence the hybridization of the qPCR primers and finally influence the amplification efficiency. Moreover, in this study, the overlapping rate between NGS RD-based CNV and SVS 8.2 CNV callings (100%) is better than previous NGS reports, and this is probably because we use the F0 generation pigs.

Illumina Porcine SNP60 BeadChip is designed using Duroc, Landrace, Pietran, and Large White SNP information. GWAS research using this chip have been successfully carried out in intercross population such as Large white × Min, Large white × Erhualian, Iberian × Landrace^2^,^4^,^41^. In the results of CNV-based association, we found 8 CNVs were significantly associated with at least one meat quality trait. These results indicate that CNVs may contribute to the difference of meat quality. The results reveal alternative variations can explain the missing heritability of complex traits.

Among these 8 CNVs, one of the interesting CNVs is CNV15. When analyzing genes within and near CNVs, we found two genes, one gene (*NOD1*) within the CNV and one gene (*PLEKHA8*) near the CNV. The *PLEKHA8* gene encodes the protein of the four-phosphate adaptor protein 2 (FAPP2) which could connect vesicular transport with lipid synthesis^39^. FAPP2 has a pleckstrin homology (PH) domain, which can bind selectively phosphatidylinositol-4-phosphate^42^. And as CNV15 was significantly associated with pH24, we proposed that it might have some relationship with FAPP2.

**Figure 4:**
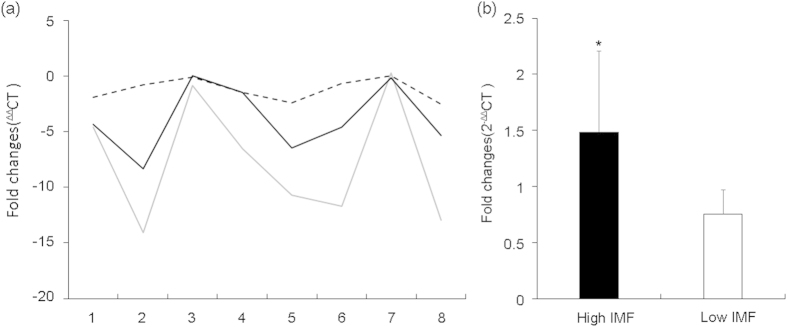
The expression of CNV12 and *Ntn1*. (**a**) is the expression pattern of CNV12 and *Ntn1*, black line represents the expressions of CNV12 EST, grey line corresponds to the expressions of *Ntn1* RNA, and dotted line the DNA copy number of CNV12. (**b**) is the expression of *Ntn1*-RNA between two IMF group, High IMF and Low IMF represent the high and low groups, * represents significant differences.

Another interesting CNV was CNV12. After retrieving the sequence of CNV12 and blast the sequence with NCBI database, we found that CNV12 was located in the predicted gene of *Ntn1*. As the Blast results match one 853 bp pig EST (BW980235 full-length enriched swine cDNA library, adult intestine Sus scrofa cDNA clone ITT010048D02 5′, mRNA sequence), we inferred that there may be some problems in the genome assembling. In order to explore the relationship between CNV12 and *Ntn1*, we first carried out qPCR to investigate the relationship between copy number and EST expression of CNV12 and then investigate the correlation between the expressions of CNV12-EST and *Ntn1*-RNA. The primers were shown in Table S11, and the results were shown in Table 4 and Fig. 4. The expression pattern was similar between the DNA dosage and EST expression, and similar pattern appeared in the expressions of CNV12-EST and *Ntn1*-RNA, suggesting CNV12 might be involved in the regulation of *Ntn1* directly. Moreover, 20 individuals from high (>5.3) and low (<1.4) IMF groups were used to explore the relationship between *Ntn1*-RNA expression and IMF. Figure 4 also showed that the fold changes of *Ntn1*-RNA expression is significant different between the two groups (with means of 1.49 and 0.76, P values < 0.05). In previous reports, *Ntn1* was highly expressed in obese but not lean adipose tissue of humans and mice^40^ which is consistent with our results, and one of *Ntn1* receptors, adenosine a2b receptor (*a2bR*) could inhibit adipogenesis^43^. Thus, we inferred that increased Ntn1 may bind with a2bR, and the decreasing of a2bR may lead to less inhibition effects on adipogenesis.

In current study, we carried out the first genome wide CNV association analysis using CNVs in pig population. However, as the Multi-variate algorithm are limited to detect small, common CNVs, some CNVs beyond the detection of Multi-variate method and the rare CNVs involved in complex traits are remain largely unknown^44^. Further, the estimation effect of CNV contributing to complex trait by integrating both SNPs, CNVs and other genomic variants will further help comprehensively understand the mechanism underline complex quantitative trait^45^. In the analysis of relationship between associated CNVs and associated SNPs, we found only one block is adjacent to tagged SNPs. Our results revealed a relatively lower CNV tagging rate comparing to cattle study^13^, this finding may be due to the limited sample size for common CNVs detection in CNV analysis. Thus, further analyses with a large sample size are needed to explore the precise relationship between CNVs and neighboring SNPs in pig.

In summary, previous SNP-based GWAS have been successfully used to identify significant genes or loci for complex traits. In this CNV-based GWAS study, our results indicate that meat quality traits were probably influenced by CNVs, and some of the CNVs may independently play their roles in determining the traits. These results provide us with an added source of variation to explain the missing heritability of complex traits, and the joint analysis of CNVs and SNPs could be a powerful way to potentially identify the causes of complex traits. The several candidate CNVs associated with meat quality such as CNV12 in SSC12 reported in this paper are worth further exploration.

## Methods

The methods were carried out in accordance with the approved guidelines.

### Ethics statements

All animals used in this study were handled and kept following the standard guidelines of experimental animals established by Ministry of Science and Technology (Beijing, China). All of the animal experiments were approved by the Institute of Animal Science, Chinese Academy of Agricultural Sciences (CAAS) (Beijing, China).

### Phenotypic and rEBV values

In this study, a three-generation Large White × Min resource population (678 pigs, including 602 F2 individuals) was genotyped using Illumina PorcineSNP60 arrays. Phenotypic data of twelve meat quality traits, including IMF, marbling, moisture, shearing force, pH6, color L6*, color a6*, color b6*, Ph24, color L24*, color a24*, and color b24* in the longissimus muscle (LM) were measured for all of the 602F2 individuals following the standard guidelines of the US National Pork Producers Council (NPPC). The pH values were measured by a HANNA HI8424NEW pH Meter (HANNA, Cluj Napoca, Romania) at 6 h and 24 h postmortem. Meat color L* represented lightness, color a* represented redness and color b* represented yellowness on the cut surface of the LM were evaluated at 6 h post mortem and 24 h postmortem using a CR-410 Minolta Chroma Meter (Konica Minolta, Tokyo, Japan). Marbling scores were analyzed by a standard NPPC photographic reference (1, 2, 3, 4, 5, 6, with 1 = devoid, 6 = overly abundant) to determine of LM at 24 h post-mortem and IMF content were measured using an ether extraction method (Soxtec Avanti 2055 Fat Extraction System, Foss Tecator, Denmark). After removing fixed non-genetic effects using DMU software (v6), rEBVs estimated by breeding values (EBV) plus residual which are used as the phenotypes in association testing of the 12 meat quality traits.

### CNV segmentation and genotyping and PCA-corrected association testing

The CNV segmentation and genotyping, and principal component analysis (PCA) corrected association testing were performed using Golden Helix SVS 8.2 (Golden Helix, Inc., Bozeman, MT, USA). The method and parameters are all follow the description of Xu *et al*.^13^. The array we used was Illumina Porcine SNP60 Beadchip, which contains 62,163 SNP probes. The GC correlation file was GC Reference sus_sscrofa_10.2 gc_digest.dsf. And finally, significant CNVs were considered at the level of P-value < 0.05 after FDR correction.

### Association studies based on SNPs

Mix Model and Regression—Genomic Control (GRAMMAR-GC) method was used for the associated analysis between SNPs and meat quality traits^46^,^47^. The genome-wide significance threshold was decided using Bonferroni correction, in which P-value (0.05) was divided by the number of effective SNPs (12,039) estimated using simple *M* method^48^.

### Haplotype block analysis and relationship between significantly combinative CNVs and SNPs

Haplotype block was employed to investigate linkage disequilibrium (LD) patterns in the regions containing significant associated CNVs using Haploview (v 4.2)^49^,^50^. In previous research, Du *et al*. presented a linkage disequilibrium (LD) map in pig population, and found the maximum segment was 1 cM (about 1 Mb)^51^. And as the average distance between SNPs was about 40 K in the PorcineSNP60 chip, 25 SNPs on the both upstream and downstream were selected directions of each CNV. All of the significant combinative CNVs (P-values < 0.05 after FDR correction) and SNPs (P-values < 4.15E-6) were used to analyze the relationship between significantly combinative CNVs and SNPs following the method described by Xu *et al*.^13^.

### Validation of qPCR and next generation sequencing

The qPCR amplification was performed using an ABI 7900HT instrument (Applied Biosystems, Inc., Foster City, CA) in 384-well optical PCR plates. SYBR® Green primers were designed to query CNVs using the Primer 6 software. A 15-mlsystem containing 15 ng of genomic DNA, 150 nM each for the primers, and SYBR® Select Master Mix (ABI part number 4472908) were used for the reaction. The glucagon gene (GCG)^52^ was used as control of single copy control. Copy number was calculated by the method of 2^−ΔΔCT^ 53,54, where Δ CT is the differential value of target region cycle threshold (CT) of and the control region CT. And moreover, 2^−ΔΔCT^ stand for the comparison of the ^Δ^ CT value of samples with CNV to those without CNV. The PCR cycle was: 2 min at 50 °C, 10 min at 95 °C, 40 cycles of 15 sec at 95 °C and 1 min at 60 °C. A list of these 16 pair of primer sequences (CNVs and GCG) is shown in Table S3.

Twelve pigs (8 Min pigs and 4 Large White pigs) in the F0 generation were sequenced using Illumina HiSeq 2000. Every pig was sequenced with paired-end reads (100 bp) in two 500 bp insert size genomic DNA libraries. All paired-end reads were mapped to the genome Sscrofa10.2 (ftp://ftp.ensembl.org/pub/release-77/fasta/sus_scrofa/dna/Sus_scrofa.Sscrofa10.2.dna.toplevel.fa.gz) using BWA v0.5.9^55^ with the parameter(−t 4 −k 32 −M and −R), and the BAM data were merged and sorted using Samtools v0.1.18^56^. The CNVs were detected using CNVnator v0.3 software according to the previous studies with the parameter (-call 100)^32^. CNV calls were filtered by the criteria of P-value < 0.01and size > 1 Kb. Calls overlapping with gaps which are larger than or equal to 5 bp in the reference genome were also excluded.

### Gene content and functional analysis

The pig annotated genes were downloaded from BioMart (http://www.biomart.org/). Genes overlapping with detected CNVs and near the detected CNVs (<100 Kb) were picked out for further analysis. Annotation analysis were performed with the DAVID (http://david.abcc.ncifcrf.gov/)^57^ for Gene Ontology (GO) terms^58^ and Kyoto Encyclopedia of Genes and Genomes (KEGG) pathway analysis^59^ to provide insight into the functional enrichment of copy number variable genes.

## Additional Information

**How to cite this article**: Wang, L. *et al*. Copy number variation-based genome wide association study reveals additional variants contributing to meat quality in Swine. *Sci. Rep*. **5**, 12535; doi: 10.1038/srep12535 (2015).

## Supplementary Material

Supplementary Information

## Figures and Tables

**Table 1:** Means and phenotypic and genetic correlation of twelve meat quality traits.

	pH6	pH24	L6*	a6*	b6*	L24*	a24*	b24*	Marbling	Moisture	SFN	IMF
PH6	5.90 ± 0.31	***0.56***	***−0.50***	0.00	***−0.50***	***−***0.39	0.13	***−***0.15	0.25	0.00	***−***0.06	0.20
PH24	***0.56***	5.83 ± 0.30	***−***0.28	***−***0.06	***−***0.31	***−***0.39	0.03	***−***0.30	0.07	0.08	***−***0.17	0.01
L6^*^	***−0.50***	***−***0.34	47.68 ± 4.2	***−***0.05	***0.75***	***0.75***	***−***0.42	0.48	***−***0.11	0.05	***−***0.14	***−***0.06
a6^*^	***−***0.09	0.00	0.09	13.86 ± 2.28	0.23	***−***0.09	0.41	0.05	0.18	***−***0.19	***−***0.09	0.34
b6^*^	***−***0.22	***−***0.25	0.55	***−***0.19	7.10 ± 1.89	***0.63***	***−***0.18	***0.51***	0.03	***−***0.12	***−***0.15	0.11
L24^*^	***−***0.36	***−***0.40	***0.71***	***−***0.09	0.48	49.96 ± 3.88	***−***0.44	***0.67***	***−***0.01	***−***0.03	***−***0.10	0.04
a24^*^	0.02	0.05	***−***0.27	***0.63***	***−0.53***	***−***0.31	14.26 ± 1.83	***−***0.07	0.16	***−***0.21	***−***0.01	0.25
b24^*^	0.06	***−***0.33	0.24	***−***0.41	***0.66***	0.43	***−0.51***	7.24 ± 1.91	0.18	***−***0.21	***−***0.04	0.28
Marbling	0.17	0.00	***−***0.02	0.02	0.23	0.04	***−***0.10	0.24	2.81 ± 1.03	***−***0.48	***−***0.20	***0.65***
Moisture	***−***0.06	0.05	0.06	***−***0.24	***−***0.01	0.02	***−***0.21	***−***0.03	***−***0.42	73.28 ± 1.91	0.10	***−0.69***
SFN	***−***0.04	***−***0.07	***−***0.16	0.10	***−***0.23	***−***0.14	0.19	***−***0.16	***−***0.23	0.06	5.12 ± 1.15	***−***0.14
IMF	0.22	***−***0.06	***−***0.04	0.25	0.11	0.03	0.15	0.19	***0.61***	***−0.73***	***−***0.12	2.85 ± 1.82

1. Means ± SD were on diagonals, genetic correlations were above diagonals, phenotypic correlations were below diagonals. The absolute values ≥0.50 were shown in bold and italic.

2. pH6, pH24, L6*, a6*, b6*, L24*, a24*, b24*, SFN, and IMF were stand for pH value at 6 h postmortem, pH value at 24 h postmortem, color L* at 6 h postmortem, color a* at 6 h postmortem, color b* at 6 h postmortem, color L* at 24 h postmortem, color a* at 24 h postmortem, color b* at 24 h postmortem, share force, and intramuscular fat.

**Table 2:** Description of 15 CNVs detected and their association with traits.

No.	Chromosome Name	Start Position	End Position	Length (bp)	Frequency of CNV	P value after FDR correction				validation		
						b24*	Marbling	pH6	IMF	NGS	QPCR	Published paper
CNV1	1	242457549	242519391	61843	0.87	0.9608	0.0586	0.1401	***0.0435***	Yes	No	Yes
CNV2	3	94706101	94868661	162561	0.83	0.7124	0.5848	0.2257	0.3560	Yes	—	No
CNV3	4	96277909	96381947	104039	0.26	0.6598	0.7011	0.1532	0.9950	Yes	Yes	Yes
CNV4	4	133873894	133948941	75048	0.40	0.9770	0.6258	0.4987	0.7785	Yes	Yes	Yes
CNV5	5	21339891	22435998	1096108	0.14	1.0000	0.3587	0.1169	0.5496	Yes	Yes	Yes
CNV6	5	60936295	61005896	69602	0.26	0.4648	0.1723	0.8430	0.4360	Yes	Yes	No
CNV7	5	79366287	79807784	441498	0.72	0.1436	0.0756	0.2109	0.2191	Yes	Yes	Yes
CNV8	7	121924542	122002552	78011	0.85	***0.0093***	0.3351	0.1381	0.5722	Yes	No	Yes
CNV9	10	9369752	9462206	92455	0.47	***0.0069***	***0.0283***	***0.0016***	***0.0272***	Yes	Yes	Yes
CNV10	10	49173528	49255139	81612	0.57	0.0522	0.0750	***0.0010***	***0.0481***	Yes	Yes	Yes
CNV11	12	11462476	11720468	257993	0.58	***0.0080***	0.0877	0.1325	0.1275	Yes	No	Yes
CNV12	12	56893678	57020468	126791	0.79	0.0598	***0.0055***	***0.0012***	***0.0030***	Yes	Yes	Yes
CNV13	17	41839309	41873384	34076	0.53	***0.0074***	0.0625	0.0989	0.0717	Yes	Yes	No
CNV14	18	23383197	23623258	240062	0.43	0.9936	0.4533	0.1470	0.7194	Yes	Yes	Yes
CNV15	18	46776812	46983072	206261	0.35	0.6018	0.1609	***0.0110***	0.1617	Yes	Yes	Yes

1. p values < 0.05 after FDR correction were shown in bold and italic.

2. pH6, b24*, and IMF were stand for pH value at 6 h postmortem, color b* at 24 h postmortem, and intramuscular fat.

**Table 3:** Genome-wide significant SNPs with 7 meat quality traits.

SNPs	Chromosome	Position	pH6	a6*	a24*	SFN	IMF	Marbling	Moisture
H3GA0056170	NA	NA	1.98E-05	3.63E-02	4.05E-01	4.72E-01	1.32E-02	***5.60E-07***	4.99E-04
ALGA0107518	NA	NA	3.06E-05	3.26E-02	3.53E-01	5.40E-01	1.87E-02	***9.67E-07***	6.37E-04
MARC0004712	NA	NA	7.55E-06	3.18E-03	2.02E-01	6.69E-01	8.12E-03	***9.85E-07***	4.59E-04
ASGA0085522	NA	NA	6.12E-05	5.85E-03	6.53E-02	2.29E-01	1.63E-03	***2.00E-06***	4.21E-04
ASGA0008649	2	5642038	6.81E-01	6.94E-01	2.02E-01	***4.41E-06***	3.55E-01	7.34E-02	4.04E-01
ALGA0066945	3	139930024	3.76E-05	***3.56E-07***	***2.07E-06***	3.07E-01	***3.60E-11***	***1.08E-09***	***9.61E-08***
ASGA0054854	12	47528805	7.68E-04	1.07E-04	2.48E-03	2.83E-01	***2.69E-06***	***2.51E-06***	4.89E-04
M1GA0016908	12	52692402	1.43E-02	2.12E-05	8.99E-05	4.67E-02	***3.22E-06***	3.29E-04	3.09E-04
ASGA0102838	12	55575876	9.77E-05	***4.29E-07***	***2.72E-06***	6.29E-01	***3.15E-09***	***2.83E-08***	***3.50E-06***
ASGA0089507	12	57195654	1.16E-04	7.74E-06	1.83E-03	3.19E-01	***2.89E-06***	***5.77E-07***	2.26E-05
ASGA0094812	12	57394039	***2.54E-06***	***6.88E-07***	4.72E-05	6.31E-01	***1.53E-09***	***2.73E-11***	***1.57E-09***
ASGA0100525	12	57622308	1.52E-05	1.26E-03	3.87E-02	8.99E-01	8.85E-03	***2.49E-06***	2.24E-03
MARC0027759	12	57625866	***2.05E-06***	8.94E-04	4.36E-02	6.47E-01	4.41E-03	4.37E-06	1.34E-03
ALGA0067072	12	57831831	1.06E-05	2.79E-03	8.39E-02	7.39E-01	9.81E-03	***3.11E-06***	1.11E-03
ALGA0067099	12	57950908	8.25E-06	3.77E-03	2.51E-01	7.41E-01	1.10E-02	***1.39E-06***	6.56E-04
ALGA0067119	12	58078076	1.29E-01	8.15E-05	7.98E-05	2.48E-02	***9.53E-07***	2.17E-04	2.80E-05
DIAS0000861	12	58132333	1.35E-05	3.68E-03	1.95E-01	5.33E-01	5.17E-03	***7.24E-07***	6.27E-04
MARC0017000	12	58347308	1.29E-05	1.85E-05	1.25E-03	4.45E-01	***1.79E-08***	***3.81E-10***	***9.99E-09***
MARC0030345	12	58934290	5.42E-06	2.77E-03	1.50E-01	8.19E-01	1.12E-02	***1.49E-06***	1.85E-03
MARC0009546	12	58942845	8.62E-06	3.07E-03	1.72E-01	8.88E-01	1.27E-02	***1.07E-06***	9.41E-04
M1GA0017195	12	60768750	2.67E-02	1.64E-02	9.28E-02	3.86E-02	***1.03E-05***	***3.55E-06***	1.48E-05
ASGA0084548	12	60923280	1.05E-03	4.42E-04	1.28E-02	6.38E-02	1.68E-04	***6.17E-07***	7.74E-05
ASGA0099873	12	61061041	1.87E-04	2.82E-04	9.06E-03	3.86E-02	5.68E-05	***1.61E-07***	2.12E-04
ALGA0109745	12	61142611	1.51E-03	1.61E-05	3.47E-04	1.25E-01	8.42E-07	3.80E-08	1.94E-06

1. p values < 4.56E-6 after FDR correction were shown in bold and italic.

2. pH6, a6*, a24*, SFN, and IMF were stand for pH value at 6 h postmortem, color a* at 6 h postmortem, color a* at 24 h postmortem, share force, and intramuscular fat.

**Table 4:** Results of expression for CNVR12 and *Ntn1*.

Individual	^ΔΔ^CT (Fold changes)		
	CNV12-DNA	CNV12-EST	***Ntn1***-RNA
1	***−***1.89	***−***4.59	***−***4.34
2	***−***0.79	***−***14.07	***−***8.35
3	***−***0.10	***−***0.87	0.00
4	***−***1.48	***−***6.54	***−***1.50
5	***−***2.41	***−***10.68	***−***6.46
6	***−***0.65	***−***11.68	***−***4.60
7	0.00	0.23	***−***0.17
8	***−***2.56	***−***12.94	***−***5.36

## References

[b1] Nonneman D. J. . Genome-wide association of meat quality traits and tenderness in swine. J Anim Sci 91, 4043–4050 (2013). 23942702 10.2527/jas.2013-6255

[b2] Luo W. . Genome-wide association analysis of meat quality traits in a porcine Large White x Minzhu intercross population. Int J Biol Sci 8, 580–595 (2012). 22532790 10.7150/ijbs.3614 PMC3334672

[b3] Serenius T., Sevon-Aimonen M. L., Kause A., Mantysaari E. A. & Maki-Tanila A. Genetic associations of prolificacy with performance, carcass, meat quality, and leg conformation traits in the Finnish Landrace and Large White pig populations. J Anim Sci 82, 2301–2306 (2004). 15318728 10.2527/2004.8282301x

[b4] Ma J. . Genome-wide association study of meat quality traits in a White DurocxErhualian F2 intercross and Chinese Sutai pigs. PLoS One 8, e64047 (2013). 23724019 10.1371/journal.pone.0064047 PMC3665833

[b5] Sanchez M. P. . A genome-wide association study of production traits in a commercial population of Large White pigs: evidence of haplotypes affecting meat quality. Genet Sel Evol 46, 12 (2014). 24528607 10.1186/1297-9686-46-12 PMC3975960

[b6] Ernst C. W. & Steibel J. P. Molecular advances in QTL discovery and application in pig breeding. Trends Genet 29, 215–224 (2013). 23498076 10.1016/j.tig.2013.02.002

[b7] Heidt H. . A genetical genomics approach reveals new candidates and confirms known candidate genes for drip loss in a porcine resource population. Mamm Genome 24, 416–426 (2013). 24026665 10.1007/s00335-013-9473-z

[b8] Zambonelli P. . SNPs detection in DHPS-WDR83 overlapping genes mapping on porcine chromosome 2 in a QTL region for meat pH. BMC Genet 14, 99 (2013). 24103193 10.1186/1471-2156-14-99 PMC4124853

[b9] Duthie C. A. . Quantitative trait loci for meat quality traits in pigs considering imprinting and epistatic effects. Meat Science 87, 394–402 (2011). 21146324 10.1016/j.meatsci.2010.11.017

[b10] Hu Z. L., Park C. A., Wu X. L. & Reecy J. M. Animal QTLdb: an improved database tool for livestock animal QTL/association data dissemination in the post-genome era. Nucleic Acids Res 41, D871–D879 (2013). 23180796 10.1093/nar/gks1150 PMC3531174

[b11] Soller M., Weigend S., Romanov M. N., Dekkers J. C. & Lamont S. J. Strategies to assess structural variation in the chicken genome and its associations with biodiversity and biological performance. Poult Sci 85, 2061–2078 (2006). 17135660 10.1093/ps/85.12.2061

[b12] Schreiweis M. A., Hester P. Y. & Moody D. E. Identification of quantitative trait loci associated with bone traits and body weight in an F2 resource population of chickens. Genet Sel Evol 37, 677–698 (2005). 16277974 10.1186/1297-9686-37-7-677 PMC2697244

[b13] Xu L. . Genome wide CNV analysis reveals additional variants associated with milk production traits in Holsteins. BMC Genomics 15, 683 (2014). 25128478 10.1186/1471-2164-15-683 PMC4152564

[b14] Manolio T. A. . Finding the missing heritability of complex diseases. Nature 461, 747–753 (2009). 19812666 10.1038/nature08494 PMC2831613

[b15] Ionita-Laza I., Rogers A. J., Lange C., Raby B. A. & Lee C. Genetic association analysis of copy-number variation (CNV) in human disease pathogenesis. Genomics 93, 22–26 (2009). 18822366 10.1016/j.ygeno.2008.08.012 PMC2631358

[b16] Fanciulli M. . FCGR3B copy number variation is associated with susceptibility to systemic, but not organ-specific, autoimmunity. Nat Genet 39, 721–723 (2007). 17529978 10.1038/ng2046 PMC2742197

[b17] Sebat J. . Strong association of de novo copy number mutations with autism. Science 316, 445–449 (2007). 17363630 10.1126/science.1138659 PMC2993504

[b18] Walsh T. . Rare structural variants disrupt multiple genes in neurodevelopmental pathways in schizophrenia. Science 320, 539–543 (2008). 18369103 10.1126/science.1155174

[b19] Yang Y. . Gene copy-number variation and associated polymorphisms of complement component C4 in human systemic lupus erythematosus (SLE): Low copy number is a risk factor for and high copy number is a protective factor against SLE susceptibility in European Americans. Am J Hum Genet 80, 1037–1054 (2007). 17503323 10.1086/518257 PMC1867093

[b20] Xu L. Y. . A genome-wide survey reveals a deletion polymorphism associated with resistance to gastrointestinal nematodes in Angus cattle. Funct Integr Genomic 14, 333–339 (2014). 10.1007/s10142-014-0371-6 24718732

[b21] Hou Y. L. . Analysis of copy number variations in Holstein cows identify potential mechanisms contributing to differences in residual feed intake. Funct Integr Genomic 12, 717–723 (2012). 10.1007/s10142-012-0295-y 22991089

[b22] Luo J. . Genome-wide copy number variant analysis in inbred chickens lines with different susceptibility to Marek’s disease. G3 3, 217–223 (2013). 23390598 10.1534/g3.112.005132 PMC3564982

[b23] Wang X. & Byers S. Copy Number Variation in Chickens: A Review and Future Prospects. Microarrays 3, 24–38 (2014). 10.3390/microarrays3010024 PMC5003453 27605028

[b24] Karyadi D. M. . A Copy Number Variant at the KITLG Locus Likely Confers Risk for Canine Squamous Cell Carcinoma of the Digit. Plos Genet 9, e1003409 (2013). 23555311 10.1371/journal.pgen.1003409 PMC3610924

[b25] Jiang J. C. . Global copy number analyses by next generation sequencing provide insight into pig genome variation. Bmc Genomics 15, 593 (2014). 25023178 10.1186/1471-2164-15-593 PMC4111851

[b26] Ramayo-Caldas Y. . Copy number variation in the porcine genome inferred from a 60 k SNP BeadChip. BMC Genomics 11, 593 (2010). 20969757 10.1186/1471-2164-11-593 PMC3091738

[b27] Chen C. Y. . A comprehensive survey of copy number variation in 18 diverse pig populations and identification of candidate copy number variable genes associated with complex traits. BMC Genomics 13, 733 (2012). 23270433 10.1186/1471-2164-13-733 PMC3543711

[b28] Paudel Y. . Evolutionary dynamics of copy number variation in pig genomes in the context of adaptation and domestication. BMC Genomics 14, 449 (2013). 23829399 10.1186/1471-2164-14-449 PMC3716681

[b29] Wang J. Y. . A genome-wide detection of copy number variations using SNP genotyping arrays in swine. BMC Genomics 13, 273 (2012). 22726314 10.1186/1471-2164-13-273 PMC3464621

[b30] Wang J. Y. . Identification of Genome-Wide Copy Number Variations among Diverse Pig Breeds Using SNP Genotyping Arrays. Plos One 8, e68683 (2013). 23935880 10.1371/journal.pone.0068683 PMC3720780

[b31] Wang L. G. . Genome-Wide Copy Number Variations Inferred from SNP Genotyping Arrays Using a Large White and Minzhu Intercross Population. Plos One 8, e74879 (2013). 24098353 10.1371/journal.pone.0074879 PMC3787955

[b32] Abyzov A., Urban A. E., Snyder M. & Gerstein M. CNVnator: An approach to discover, genotype, and characterize typical and atypical CNVs from family and population genome sequencing. Genome Res 21, 974–984 (2011). 21324876 10.1101/gr.114876.110 PMC3106330

[b33] Wimmers K. . QTL for microstructural and biophysical muscle properties and body composition in pigs. BMC Genet 7, 15 (2006). 16526961 10.1186/1471-2156-7-15 PMC1456989

[b34] Edwards D. B. . Quantitative trait locus mapping in an F2 Duroc x Pietrain resource population: II. Carcass and meat quality traits. J Anim Sci 86, 254–266 (2008). 17965326 10.2527/jas.2006-626

[b35] Choi I. . Identification of Carcass and Meat Quality QTL in an F(2) Duroc x Pietrain Pig Resource Population Using Different Least-Squares Analysis Models. Front Genet 2, 18 (2011). 22303314 10.3389/fgene.2011.00018 PMC3268573

[b36] Estelle J. . A quantitative trait locus genome scan for porcine muscle fiber traits reveals overdominance and epistasis. J Anim Sci 86, 3290–3299 (2008). 18641172 10.2527/jas.2008-1034

[b37] van Wijk H. J. . Identification of quantitative trait loci for carcass composition and pork quality traits in a commercial finishing cross. J Anim Sci 84, 789–799 (2006). 16543555 10.2527/2006.844789x

[b38] Lappas M. NOD1 expression is increased in the adipose tissue of women with gestational diabetes. J Endocrinol 222, 99–112 (2014). 24829218 10.1530/JOE-14-0179

[b39] D’Angelo G., Rega L. R. & De Matteis M. A. Connecting vesicular transport with lipid synthesis: FAPP2. Bba-Mol Cell Biol L 1821, 1089–1095 (2012). 10.1016/j.bbalip.2012.01.003 PMC4331668 22266015

[b40] Ramkhelawon B. . Netrin-1 promotes adipose tissue macrophage retention and insulin resistance in obesity. Nat Med 20, 377–384 (2014). 24584118 10.1038/nm.3467 PMC3981930

[b41] Ramayo-Caldas Y. . Genome-wide association study for intramuscular fatty acid composition in an Iberian x Landrace cross. J Anim Sci 90, 2883–2893 (2012). 22785162 10.2527/jas.2011-4900

[b42] Dowler S. . Identification of pleckstrin-homology-domain-containing proteins with novel phosphoinositide-binding specificities. Biochem J 351, 19–31 (2000). 11001876 10.1042/0264-6021:3510019 PMC1221362

[b43] Llambi F., Causeret F., Bloch-Gallego E. & Mehlen P. Netrin-1 acts as a survival factor via its receptors UNC5H and DCC. Embo Journal 20, 2715–2722 (2001). 11387206 10.1093/emboj/20.11.2715 PMC125255

[b44] Xu L., Hou Y., Bickhart D., Song J. & Liu G. Comparative Analysis of CNV Calling Algorithms: Literature Survey and a Case Study Using Bovine High-Density SNP Data. Microarrays 2, 171–185 (2013). 10.3390/microarrays2030171 PMC5003459 27605188

[b45] Korn J. M. . Integrated genotype calling and association analysis of SNPs, common copy number polymorphisms and rare CNVs. Nat Genet 40, 1253–1260 (2008). 18776909 10.1038/ng.237 PMC2756534

[b46] Aulchenko Y. S., de Koning D. J. & Haley C. Genomewide rapid association using mixed model and regression: A fast and simple method for genomewide pedigree-based quantitative trait loci association analysis. Genetics 177, 577–585 (2007). 17660554 10.1534/genetics.107.075614 PMC2013682

[b47] Amin N., van Duijn C. M. & Aulchenko Y. S. A genomic background based method for association analysis in related individuals. PLoS One 2, e1274 (2007). 18060068 10.1371/journal.pone.0001274 PMC2093991

[b48] Gao X. Y., Stamier J. & Martin E. R. A multiple testing correction method for genetic association studies using correlated single nucleotide polymorphisms. Genet Epidemiol 32, 361–369 (2008). 18271029 10.1002/gepi.20310

[b49] Barrett J. C., Fry B., Maller J. & Daly M. J. Haploview: analysis and visualization of LD and haplotype maps. Bioinformatics 21, 263–265 (2005). 15297300 10.1093/bioinformatics/bth457

[b50] Gabriel S. B. . The structure of haplotype blocks in the human genome. Science 296, 2225–2229 (2002). 12029063 10.1126/science.1069424

[b51] Du F. X., Clutter, A. C. & Lohuis, M. M. Characterizing linkage disequilibrium in pig populations. Int J Biol Sci 3, 166–178 (2007). 17384735 10.7150/ijbs.3.166 PMC1802018

[b52] Ballester M., Castello A., Ibanez E., Sanchez A. & Folch J. M. Real-time quantitative PCR-based system for determining transgene copy number in transgenic animals. Biotechniques 37, 610–613 (2004). 15517974 10.2144/04374ST06

[b53] Livak K. J. & Schmittgen T. D. Analysis of relative gene expression data using real-time quantitative PCR and the 2(T)(-Delta Delta C) method. Methods 25, 402–408 (2001). 11846609 10.1006/meth.2001.1262

[b54] Graubert T. A. . A high-resolution map of segmental DNA copy number variation in the mouse genome. Plos Genet 3, e3 (2007). 17206864 10.1371/journal.pgen.0030003 PMC1761046

[b55] Li H. & Durbin R. Fast and accurate short read alignment with Burrows-Wheeler transform. Bioinformatics 25, 1754–1760 (2009). 19451168 10.1093/bioinformatics/btp324 PMC2705234

[b56] Li H. . The Sequence Alignment/Map format and SAMtools. Bioinformatics 25, 2078–2079 (2009). 19505943 10.1093/bioinformatics/btp352 PMC2723002

[b57] Huang D. W., Sherman B. T. & Lempicki R. A. Systematic and integrative analysis of large gene lists using DAVID bioinformatics resources. Nat Protoc 4, 44–57 (2009). 19131956 10.1038/nprot.2008.211

[b58] Ashburner M. . Gene Ontology: tool for the unification of biology. Nat Genet 25, 25–29 (2000). 10802651 10.1038/75556 PMC3037419

[b59] Kanehisa M., Goto S., Furumichi M., Tanabe M. & Hirakawa M. KEGG for representation and analysis of molecular networks involving diseases and drugs. Nucleic Acids Res 38, D355–D360 (2010). 19880382 10.1093/nar/gkp896 PMC2808910

