# Supplementary material for: Copy number variation-based genome wide association study reveals additional variants contributing to meat quality in Swine
Source: Sci Rep. 2015 Aug 3;5:12535. doi: 10.1038/srep12535 (PMC4522650; doi:10.1038/srep12535)
Supplement: Supplementary Information [file srep12535-s1.doc]

# Copy number variation-based genome wide association study reveals additional variants contributing to meat quality in Swine

**Authors:** Ligang Wang^1*^, Lingyang Xu^1, 2*^, Xin Liu^1*^, Tian Zhang^1^, Na Li^1, 3^, El Hamidi Hay^2^, Yuebo Zhang^1^, Hua Yan^1^, Kebin Zhao^1^, George E Liu^2*^, Longchao Zhang^1*^ and Lixian Wang^1*^

**Institute, address, country:**

1. Key Laboratory of Farm Animal Genetic Resources and Germplasm Innovation of Ministry of Agriculture of China, Institute of Animal Science, Chinese Academy of Agricultural Sciences, Beijing 100193, China.
2. Animal Genomics and Improvement Laboratory, BARC, USDA-ARS, Beltsville, Maryland 20705, USA
3. Jilin Academy of Agricultural Sciences, Changchun 130033, China

^*^ These authors contributed equally to this work.

**Corresponding authors:**

Lixian Wang, Institute of Animal Science, Chinese Academy of Agricultural Sciences, Beijing 100193, China. Fax: +86-10-62818771. Email: wanglixian@caas.cn

George E Liu, Animal Genomics and Improvement Laboratory, BARC, USDA-ARS, Beltsville, Maryland 20705, USA. Fax: +1-301-504-8414. Email:George.Liu@ars.usda.gov

Longchao Zhang, Institute of Animal Science, Chinese Academy of Agricultural Sciences, Beijing 100193, China. Fax: +86-10-62818771. Email:zhlchias@163.com.


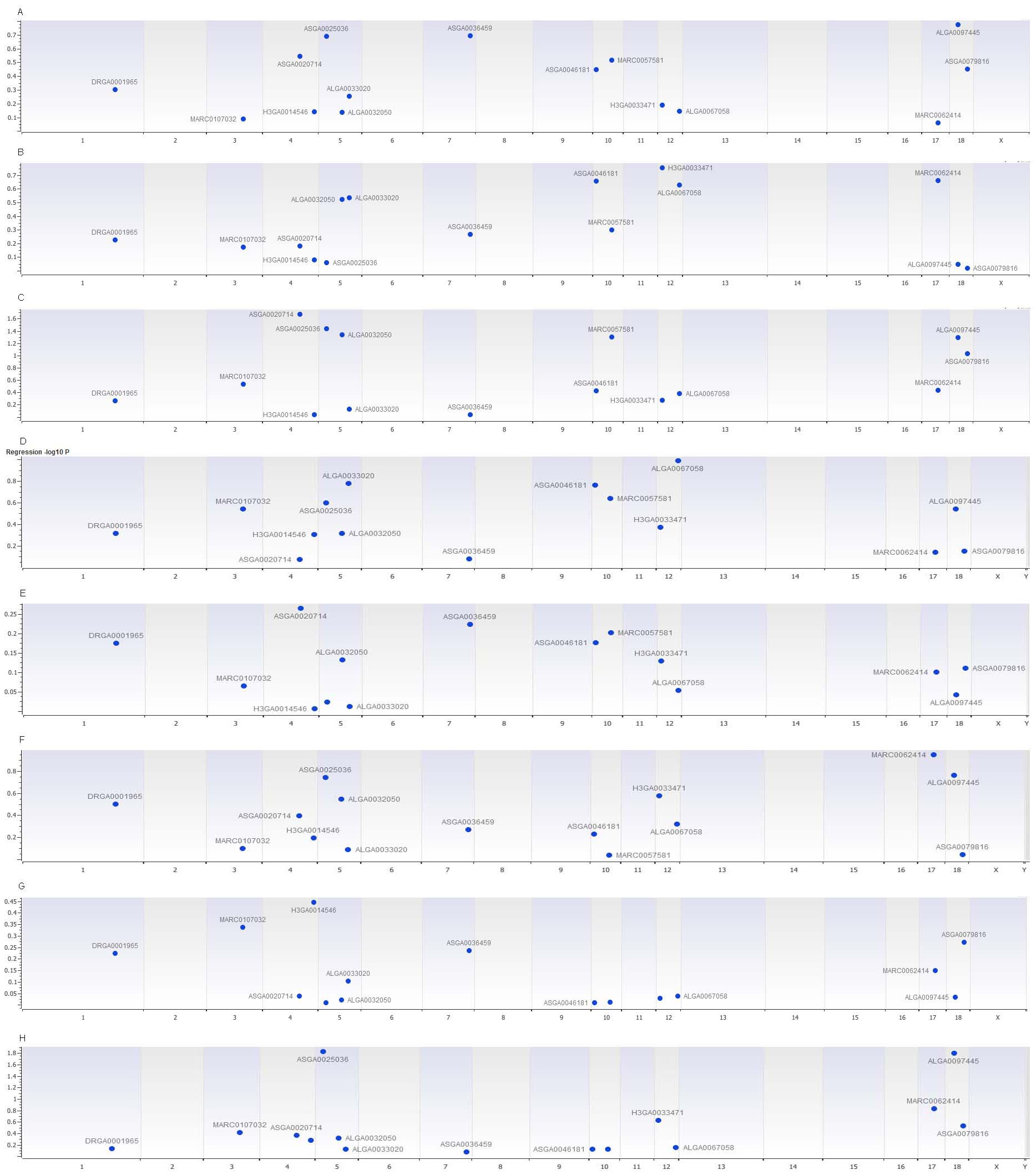
**Figure S1. Manhattan plots of associated CNVs for other meat quality traits.**

**A: pH value at 24h postmortem, B: color L* at 6h postmortem, C: color a* at 6h postmortem, D: color b* at 6h postmortem, E: color L* at 24h postmortem, F: color a* at 24h postmortem, G: moisture, H: share force.**


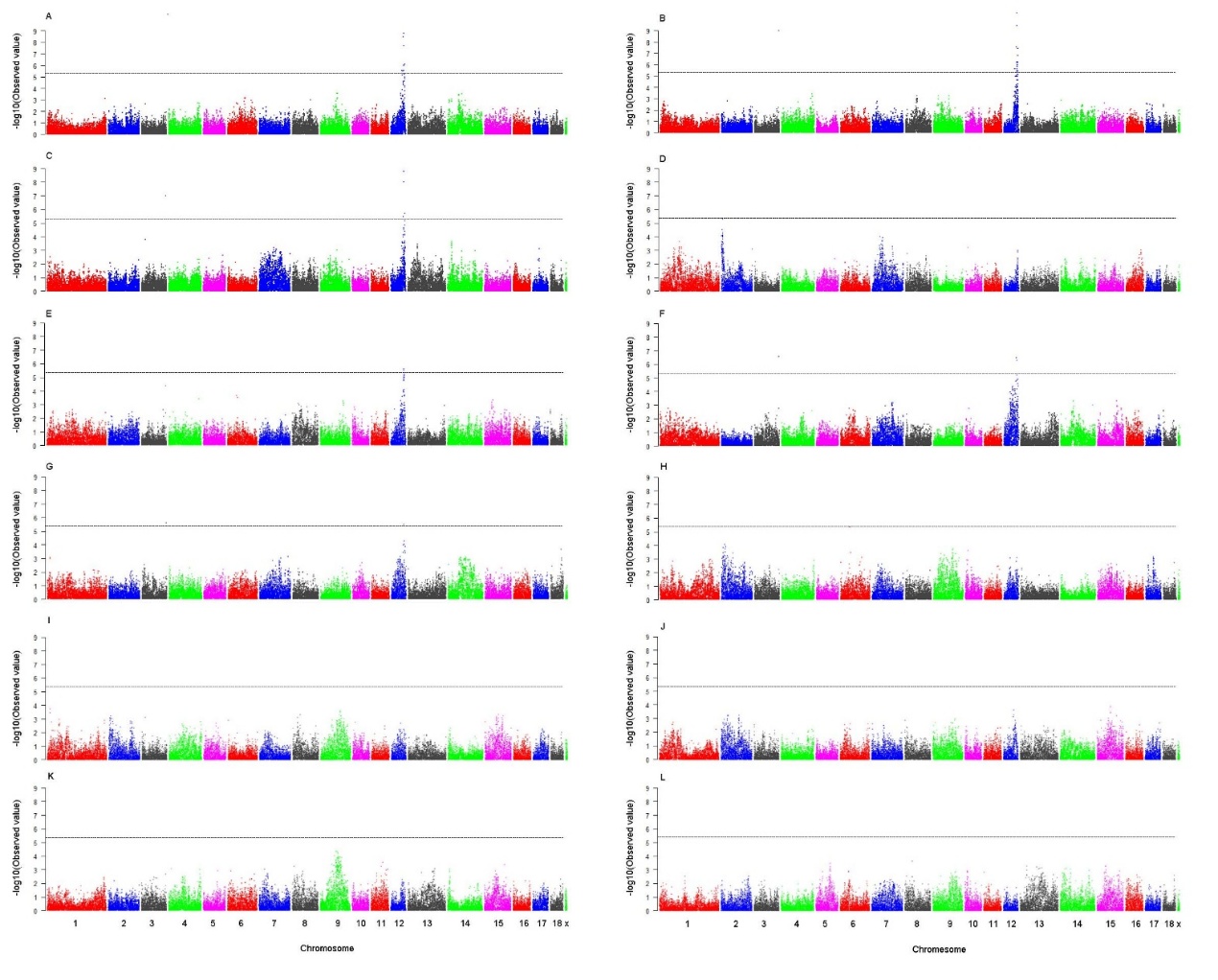
**Figure S2. Manhattan plots of associated SNPs for meat quality traits.**

**A: intramuscular fat. B: marbling, C: moisture, D: share force, E: pH value at 6h postmortem, F: color a* at 6h postmortem, G: color a* at 24h postmortem, H: color L* at 6h postmortem, I: color L* at 24h postmortem, J: color b* at 6h postmortem, K: color b* at 24h postmortem, L: pH value at 24h postmortem.**

**Table S1. The detailed information of each CNV.**

| **ID** | **Chromosome** | **Start**  **Position** | **End Position** | **Segment Mean** | **Start Marker** | **End Marker** | **Length (bp)** | **Marker Mean** | **Num 3 State Loss** | **Num 3 State Neutral** | **Num 3 State Gain** | **3 State Threshold - Mean** |
| --- | --- | --- | --- | --- | --- | --- | --- | --- | --- | --- | --- | --- |
| CNV1 | 1 | 242457549 | 242519391 | -0.92276 | DRGA0001965 | ASGA0005724 | 61843 | -1.19621 | 593 | 85 | 0 | 1.496211 |
| CNV2 | 3 | 94706101 | 94868661 | -1.06309 | MARC0107032 | H3GA0010040 | 162561 | -1.01019 | 566 | 112 | 0 | 1.310192 |
| CNV3 | 4 | 96277909 | 96381947 | -1.70528 | ASGA0020714 | ALGA0026572 | 104039 | -0.54853 | 173 | 505 | 0 | 0.848527 |
| CNV4 | 4 | 133873894 | 133948941 | -1.80739 | H3GA0014546 | ASGA0022780 | 75048 | -0.696 | 272 | 406 | 0 | 0.995998 |
| CNV5 | 5 | 21339891 | 22435998 | 0.055571 | ASGA0025036 | ALGA0031235 | 1096108 | -0.11551 | 89 | 583 | 6 | 0.415511 |
| CNV6 | 5 | 60936295 | 61005896 | 0.26284 | ALGA0032050 | ALGA0032054 | 69602 | -0.37836 | 175 | 503 | 0 | 0.678357 |
| CNV7 | 5 | 79366287 | 79807784 | -1.54033 | ALGA0033020 | ALGA0033021 | 441498 | -0.77276 | 490 | 188 | 0 | 1.072755 |
| CNV8 | 7 | 121924542 | 122002552 | -0.56298 | ASGA0036459 | MARC0079126 | 78011 | -0.69691 | 574 | 104 | 0 | 0.99691 |
| CNV9 | 10 | 9369752 | 9462206 | -0.34494 | ASGA0046181 | H3GA0029109 | 92455 | -0.7072 | 317 | 361 | 0 | 1.007197 |
| CNV10 | 10 | 49173528 | 49255139 | -0.47897 | MARC0057581 | ALGA0058870 | 81612 | -0.51376 | 384 | 294 | 0 | 0.813761 |
| CNV11 | 12 | 11462476 | 11720468 | -0.35804 | H3GA0033471 | MARC0042769 | 257993 | -0.92986 | 393 | 285 | 0 | 1.229863 |
| CNV12 | 12 | 56893678 | 57020468 | -1.61852 | ALGA0067058 | H3GA0034838 | 126791 | -1.24373 | 538 | 140 | 0 | 1.543726 |
| CNV13 | 17 | 41839309 | 41873384 | -0.01808 | MARC0062414 | MARC0019922 | 34076 | -1.12977 | 358 | 320 | 0 | 1.429769 |
| CNV14 | 18 | 23383197 | 23623258 | -0.36216 | ALGA0097445 | H3GA0050584 | 240062 | -0.45638 | 294 | 384 | 0 | 0.75638 |
| CNV15 | 18 | 46776812 | 46983072 | -1.43318 | ASGA0079816 | ALGA0098221 | 206261 | -0.5501 | 238 | 440 | 0 | 0.850104 |
| CNV16 | 1 | 342481 | 242428833 | -0.08323 | ASGA0000014 | MARC0075368 | 2.42E+08 | -0.01926 | 0 | 678 | 0 | 0.319256 |
| CNV17 | 1 | 242534196 | 315187351 | -0.11794 | INRA0005996 | MARC0030490 | 72653156 | -0.03117 | 0 | 678 | 0 | 0.33117 |
| CNV18 | 2 | 211101 | 162093226 | -0.11144 | MARC0033927 | MARC0102890 | 1.62E+08 | -0.03816 | 0 | 678 | 0 | 0.338159 |
| CNV19 | 3 | 1615014 | 94536421 | -0.08549 | M1GA0024177 | ALGA0020036 | 92921408 | -0.0303 | 0 | 678 | 0 | 0.330303 |
| CNV20 | 3 | 94887165 | 144742787 | -0.05615 | INRA0011050 | MARC0038959 | 49855623 | -0.03435 | 0 | 678 | 0 | 0.334353 |
| CNV21 | 4 | 95368 | 96252758 | -0.07873 | ASGA0016959 | ALGA0026566 | 96157391 | -0.025 | 0 | 678 | 0 | 0.324998 |
| CNV22 | 4 | 96479166 | 133848589 | -0.05197 | ASGA0020720 | ALGA0029016 | 37369424 | -0.02617 | 0 | 678 | 0 | 0.326174 |
| CNV23 | 4 | 133976248 | 143462966 | -0.05706 | ASGA0022785 | MARC0113713 | 9486719 | -0.02805 | 0 | 678 | 0 | 0.328053 |
| CNV24 | 5 | 189721 | 21273976 | -0.06271 | ASGA0027336 | ASGA0025032 | 21084256 | -0.02128 | 0 | 678 | 0 | 0.321284 |
| CNV25 | 5 | 22475419 | 60919433 | -0.10799 | ALGA0031238 | ALGA0032049 | 38444015 | -0.02704 | 0 | 678 | 0 | 0.327043 |
| CNV26 | 5 | 61020546 | 79322855 | -0.08094 | ASGA0025616 | ALGA0033016 | 18302310 | -0.00542 | 0 | 678 | 0 | 0.305417 |
| CNV27 | 5 | 79848149 | 110924766 | -0.0735 | ASGA0026383 | MARC0091287 | 31076618 | -0.02287 | 0 | 678 | 0 | 0.322871 |
| CNV28 | 6 | 2085723 | 157758951 | -0.10429 | MARC0036664 | ALGA0037844 | 1.56E+08 | -0.02677 | 0 | 678 | 0 | 0.326769 |
| CNV29 | 7 | 48748 | 121907514 | -0.07909 | INRA0022919 | H3GA0023245 | 1.22E+08 | -0.02789 | 0 | 678 | 0 | 0.327886 |
| CNV30 | 7 | 122018910 | 134718268 | -0.12326 | H3GA0023254 | MARC0098408 | 12699359 | -0.01888 | 0 | 678 | 0 | 0.318884 |
| CNV31 | 8 | 424993 | 147961912 | -0.09637 | H3GA0055033 | ALGA0107103 | 1.48E+08 | -0.02191 | 0 | 678 | 0 | 0.321912 |
| CNV32 | 9 | 92982 | 153224139 | -0.08433 | MARC0030884 | ALGA0056062 | 1.53E+08 | -0.02833 | 0 | 678 | 0 | 0.328331 |
| CNV33 | 10 | 54839 | 9336230 | -0.13879 | H3GA0055101 | H3GA0029101 | 9281392 | -0.01992 | 0 | 678 | 0 | 0.319917 |
| CNV34 | 10 | 9488500 | 49147705 | -0.07081 | DRGA0010231 | ALGA0058889 | 39659206 | -0.0002 | 0 | 678 | 0 | 0.300197 |
| CNV35 | 10 | 49305902 | 78719383 | -0.08652 | ASGA0047949 | DRGA0010743 | 29413482 | -0.00975 | 0 | 678 | 0 | 0.30975 |
| CNV36 | 11 | 22488 | 87141309 | -0.12761 | H3GA0030836 | MARC0041857 | 87118822 | -0.00358 | 0 | 678 | 0 | 0.303575 |
| CNV37 | 12 | 190362 | 11402966 | -0.13115 | MARC0089921 | ASGA0053136 | 11212605 | 0.01101 | 0 | 678 | 0 | 0.288986 |
| CNV38 | 12 | 11737023 | 56868845 | -0.09055 | MARC0050019 | M1GA0017034 | 45131823 | -0.00318 | 0 | 678 | 0 | 0.303179 |
| CNV39 | 12 | 57032666 | 63239985 | -0.14437 | M1GA0017055 | ALGA0067326 | 6207320 | 0.00245 | 0 | 678 | 0 | 0.297553 |
| CNV40 | 13 | 163156 | 217743121 | -0.08753 | ALGA0067341 | H3GA0038233 | 2.18E+08 | -0.00278 | 0 | 678 | 0 | 0.302776 |
| CNV41 | 14 | 167845 | 153786761 | -0.06603 | H3GA0038239 | H3GA0043634 | 1.54E+08 | -0.00327 | 0 | 678 | 0 | 0.303271 |
| CNV42 | 15 | 115529 | 157414092 | -0.09112 | MARC0089826 | DRGA0015684 | 1.57E+08 | -0.00941 | 0 | 678 | 0 | 0.309406 |
| CNV43 | 16 | 15148 | 86875355 | -0.09005 | H3GA0046562 | ASGA0074889 | 86860208 | -0.01789 | 0 | 678 | 0 | 0.317892 |
| CNV44 | 17 | 15175 | 41814763 | -0.07973 | ASGA0074891 | H3GA0048851 | 41799589 | -0.01064 | 0 | 678 | 0 | 0.310637 |
| CNV45 | 17 | 41888162 | 69344035 | -0.10059 | ALGA0094846 | INRA0055080 | 27455874 | -0.02689 | 0 | 678 | 0 | 0.326891 |
| CNV46 | 18 | 24285 | 23353953 | -0.05235 | ASGA0050035 | ALGA0097451 | 23329669 | -0.018 | 0 | 678 | 0 | 0.318 |
| CNV47 | 18 | 23648007 | 46758597 | -0.10509 | CASI0005720 | DIAS0000538 | 23110591 | -0.01681 | 0 | 678 | 0 | 0.316812 |
| CNV48 | 18 | 47086284 | 60537055 | -0.10743 | CASI0007635 | M1GA0023446 | 13450772 | -0.03024 | 0 | 678 | 0 | 0.330241 |

**Table S2. Comparison between CNVs detected in the study with previous reports.**

| **Our results** | | | | **Previous study** | | |
| --- | --- | --- | --- | --- | --- | --- |
| **CNV ID** | **Chr.** | **Start** | **End** | **Start** | **End** | **Study** |
| CNV1 | 1 | 242457549 | 242519391 | 242519391 | 242587191 | Wang,et al.2013 |
| CNV2 | 3 | 94706101 | 94868661 | - | - | - |
| CNV3 | 4 | 96277909 | 96381947 | 96368763 | 96393645 | Paudel, et al. 2013 |
| CNV4 | 4 | 133873894 | 133948941 | 133890364 | 133908147 | Paudel, et al. 2013 |
|  |  |  |  | 133906816 | 133908147 | Paudel, et al. 2013 |
|  |  |  |  | 133925395 | 133929031 | Paudel, et al. 2013 |
|  |  |  |  | 132376175 | 137076639 | Wang,et al.2013 |
| CNV5 | 5 | 21339891 | 22435998 | 21352658 | 21354063 | Paudel, et al. 2013 |
|  |  |  |  | 21352658 | 21354063 | Paudel, et al. 2013 |
|  |  |  |  | 21400655 | 21417241 | Paudel, et al. 2013 |
|  |  |  |  | 21400725 | 21410080 | Paudel, et al. 2013 |
|  |  |  |  | 21400725 | 21410080 | Paudel, et al. 2013 |
|  |  |  |  | 21412183 | 21423468 | Paudel, et al. 2013 |
|  |  |  |  | 21419510 | 21423463 | Paudel, et al. 2013 |
|  |  |  |  | 21423524 | 21466393 | Paudel, et al. 2013 |
|  |  |  |  | 21425286 | 21426314 | Paudel, et al. 2013 |
|  |  |  |  | 21425286 | 21426314 | Paudel, et al. 2013 |
|  |  |  |  | 21435158 | 21447549 | Paudel, et al. 2013 |
|  |  |  |  | 21435158 | 21458364 | Paudel, et al. 2013 |
|  |  |  |  | 21447758 | 21458364 | Paudel, et al. 2013 |
|  |  |  |  | 21447758 | 21449842 | Paudel, et al. 2013 |
|  |  |  |  | 21451603 | 21466393 | Paudel, et al. 2013 |
|  |  |  |  | 21466507 | 21500868 | Paudel, et al. 2013 |
|  |  |  |  | 21472070 | 21473323 | Paudel, et al. 2013 |
|  |  |  |  | 21472070 | 21473323 | Paudel, et al. 2013 |
|  |  |  |  | 21472070 | 21473321 | Paudel, et al. 2013 |
|  |  |  |  | 21482565 | 21484931 | Paudel, et al. 2013 |
|  |  |  |  | 21495268 | 21500814 | Paudel, et al. 2013 |
|  |  |  |  | 21499444 | 21500868 | Paudel, et al. 2013 |
|  |  |  |  | 21500989 | 21508446 | Paudel, et al. 2013 |
|  |  |  |  | 21500989 | 21503462 | Paudel, et al. 2013 |
|  |  |  |  | 21506281 | 21511607 | Paudel, et al. 2013 |
|  |  |  |  | 21546483 | 21553251 | Paudel, et al. 2013 |
|  |  |  |  | 21546483 | 21560021 | Paudel, et al. 2013 |
|  |  |  |  | 21546483 | 21604092 | Paudel, et al. 2013 |
|  |  |  |  | 21589729 | 21596565 | Paudel, et al. 2013 |
|  |  |  |  | 21597866 | 21601531 | Paudel, et al. 2013 |
|  |  |  |  | 21600009 | 21623744 | Paudel, et al. 2013 |
|  |  |  |  | 21606912 | 21621352 | Paudel, et al. 2013 |
|  |  |  |  | 21674132 | 21676876 | Paudel, et al. 2013 |
|  |  |  |  | 21711877 | 21718644 | Paudel, et al. 2013 |
|  |  |  |  | 21711877 | 21725420 | Paudel, et al. 2013 |
|  |  |  |  | 21711877 | 21780280 | Paudel, et al. 2013 |
|  |  |  |  | 21744034 | 21745381 | Paudel, et al. 2013 |
|  |  |  |  | 21757474 | 21764328 | Paudel, et al. 2013 |
|  |  |  |  | 21765973 | 21769115 | Paudel, et al. 2013 |
|  |  |  |  | 21781920 | 21802340 | Paudel, et al. 2013 |
|  |  |  |  | 21826873 | 21829024 | Paudel, et al. 2013 |
|  |  |  |  | 21827004 | 21828262 | Paudel, et al. 2013 |
|  |  |  |  | 21837218 | 21838469 | Paudel, et al. 2013 |
|  |  |  |  | 21854271 | 21857928 | Paudel, et al. 2013 |
|  |  |  |  | 21855118 | 21856380 | Paudel, et al. 2013 |
|  |  |  |  | 21907929 | 21930649 | Paudel, et al. 2013 |
|  |  |  |  | 21907929 | 21929449 | Paudel, et al. 2013 |
|  |  |  |  | 21929449 | 21930649 | Paudel, et al. 2013 |
|  |  |  |  | 22070697 | 22075837 | Paudel, et al. 2013 |
|  |  |  |  | 22075957 | 22086191 | Paudel, et al. 2013 |
|  |  |  |  | 22095901 | 22098844 | Paudel, et al. 2013 |
|  |  |  |  | 22110932 | 22112625 | Paudel, et al. 2013 |
|  |  |  |  | 22111011 | 22112075 | Paudel, et al. 2013 |
|  |  |  |  | 22132982 | 22175526 | Paudel, et al. 2013 |
|  |  |  |  | 22156567 | 22158979 | Paudel, et al. 2013 |
|  |  |  |  | 22169587 | 22175095 | Paudel, et al. 2013 |
|  |  |  |  | 22171668 | 22172694 | Paudel, et al. 2013 |
|  |  |  |  | 22176447 | 22178885 | Paudel, et al. 2013 |
|  |  |  |  | 22212829 | 22213919 | Paudel, et al. 2013 |
|  |  |  |  | 22229672 | 22243232 | Paudel, et al. 2013 |
|  |  |  |  | 22230023 | 22243232 | Paudel, et al. 2013 |
|  |  |  |  | 22293233 | 22313758 | Paudel, et al. 2013 |
|  |  |  |  | 22314253 | 22319761 | Paudel, et al. 2013 |
|  |  |  |  | 22316334 | 22317360 | Paudel, et al. 2013 |
|  |  |  |  | 22406663 | 22407727 | Paudel, et al. 2013 |
|  |  |  |  | 22416536 | 22421454 | Paudel, et al. 2013 |
|  |  |  |  | 22423845 | 22433397 | Paudel, et al. 2013 |
|  |  |  |  | 21238084 | 22435998 | Wang,et al.2013 |
| CNV6 | 5 | 60936295 | 61005896 | - | - | - |
| CNV7 | 5 | 79366287 | 79807784 | 79793905 | 79803761 | Paudel, et al. 2013 |
|  |  |  |  | 79804427 | 79805921 | Paudel, et al. 2013 |
| CNV8 | 7 | 121924542 | 122002552 | 121886213 | 121905454 | Paudel, et al. 2013 |
| CNV9 | 10 | 9369752 | 9462206 | 8641431 | 11098302 | Wang,et al.2013 |
| CNV10 | 10 | 49173528 | 49255139 | 48604454 | 52249156 | Wang,et al.2013 |
| CNV11 | 12 | 11462476 | 11720468 | 11498272 | 11509249 | Paudel, et al. 2013 |
|  |  |  |  | 11498272 | 11517985 | Paudel, et al. 2013 |
|  |  |  |  | 11498272 | 11517985 | Paudel, et al. 2013 |
|  |  |  |  | 11511645 | 11517338 | Paudel, et al. 2013 |
|  |  |  |  | 11567999 | 11603308 | Paudel, et al. 2013 |
|  |  |  |  | 11567999 | 11599828 | Paudel, et al. 2013 |
|  |  |  |  | 11600100 | 11603308 | Paudel, et al. 2013 |
|  |  |  |  | 11604311 | 11632158 | Paudel, et al. 2013 |
|  |  |  |  | 11604311 | 11632158 | Paudel, et al. 2013 |
|  |  |  |  | 11647583 | 11654209 | Paudel, et al. 2013 |
|  |  |  |  | 11672261 | 11677952 | Paudel, et al. 2013 |
|  |  |  |  | 11672261 | 11677952 | Paudel, et al. 2013 |
|  |  |  |  | 11678093 | 11689263 | Paudel, et al. 2013 |
|  |  |  |  | 11689384 | 11731208 | Paudel, et al. 2013 |
|  |  |  |  | 11706675 | 11753823 | Wang,et al.2013 |
| CNV12 | 12 | 56893678 | 57020468 | 56935662 | 56974319 | Paudel, et al. 2013 |
| CNV13 | 17 | 41839309 | 41873384 | - | - | - |
| CNV14 | 18 | 23383197 | 23623258 | 23355377 | 23377678 | Paudel, et al. 2013 |
|  |  |  |  | 23418826 | 23430632 | Paudel, et al. 2013 |
|  |  |  |  | 23430733 | 23442223 | Paudel, et al. 2013 |
|  |  |  |  | 23515022 | 23517523 | Paudel, et al. 2013 |
|  |  |  |  | 23546438 | 23559389 | Paudel, et al. 2013 |
|  |  |  |  | 23555482 | 23558621 | Paudel, et al. 2013 |
|  |  |  |  | 23604042 | 23606742 | Paudel, et al. 2013 |
|  |  |  |  | 23005936 | 24112411 | Wang,et al.2013 |
| CNV15 | 18 | 46776812 | 46983072 | 46783986 | 46799426 | Paudel, et al. 2013 |
|  |  |  |  | 46805667 | 46814845 | Paudel, et al. 2013 |

**Table S3.** The primers used in quantitative PCR validation analysis.

| **NO.** | **Primers** | |
| --- | --- | --- |
|  | **Sense** | **Antisense** |
| CNV1 | CAACACAGGAGAACAAGGACAG | GGACTACGGAATAGCAAGCATT |
| CNV2 | CCAGATAGCAAGGCAGACAGT | CCAGATAGCAAGGCAGACAGT |
| CNV3 | GCCTCTTCTCTTCCTCACCTT | AGTCCTTATCACAGCAGCAATG |
| CNV4 | CCTCCTCCGATGTAAGACCAA | CTCTCACCACCTCTCCAAGTT |
| CNV5 | TTGAGAATCCGACCAGTATCCA | AGGACTTGAGCCGCATCTG |
| CNV6 | CACCACAGCCACAGCATCT | AACGAACCTGACTACCATCCAA |
| CNV7 | CCACTAAGCCACAACAGGAAC | GGAGGATTCTTGCCACATCTTC |
| CNV8 | TATTGCTTCCTGGACCTGTCA | GTCACAAGGCTCAGAGGCT |
| CNV9 | GACAGACAGACAGACAGACAGA | GCCACCTGATGCCGTAGAT |
| CNV10 | ATTAGAACACAGACGGTAGG | TGAACAGATTGCTCCACAT |
| CNV11 | TGCCTTCCTTATCTCTGCTTCT | GTCTCCACCTTCGCTACTGT |
| CNV12 | CTGTCTGTCTGTCTGTCTGTCT | TGCTGTGGCTGTGGTGTAG |
| CNV13 | CAAGCAACAGCAGTGACAGT | TAGCAGCAACAACAACAACAAC |
| CNV14 | GCGTAACAGCAATGGTGATAGA | TGGCAACAATCTGGTGAATGAT |
| CNV15 | TTCTGCGGTTCCAATCATCAC | CCACTCATTCCATCTGCTTCTT |
| GCG | AAGCTTCAAACAGGGGTACAAT | CCACTTGGAATGTTACCCTAATG |

**Table S4. Results of the CNVs qPCR validation.**

| **ID.** | **916904** | **918605** | **918701** | **919802** | **919808** | **929610** | **1015103** | **1015105** | **1015107** | **1016907** | **1032007** | **1037317** |
| --- | --- | --- | --- | --- | --- | --- | --- | --- | --- | --- | --- | --- |
| CNV1 | 0.888679 | 1.094989 | 0.999994 | 0.900203 | 1.050631 | 0.816039 | 0.949415 | 0.824256 | 1.030923 | 1.257546 | 1.452034 | 1.118044 |
| CNV2 | - | - | - | - | - | - | - | - | - | - | - | - |
| CNV3 | 0.915943 | 0.853805 | 0.565088 | 0.856605 | 1.041812 | 0.750221 | 0.786522 | 0.789999 | 0.845464 | 1.102306 | 0.729433 | 0.999991 |
| CNV4 | 0.715389 | 0.710885 | 0.760288 | 0.695672 | 0.834982 | 0.482725 | 0.3965 | 0.573404 | 0.713784 | 0.999992 | 0.793255 | 0.833341 |
| CNV5 | 1.647247 | 1.825882 | 1.366102 | 1.717776 | 1.88621 | 1.945446 | 1.405328 | 1.920651 | 1.000032 | 3.244024 | 1.843289 | 1.633212 |
| CNV6 | 0.949919 | 0.666434 | 0.714796 | 0.78442 | 1.007929 | 0.879566 | 0.745628 | 0.7196 | 0.899633 | 1.000011 | 0.849158 | 1.121027 |
| CNV7 | 0.558066 | 0.47836 | 0.579029 | 0.466179 | 0.220391 | 0.411741 | 0.587643 | 0.541617 | 0.562371 | 1.000016 | 0.576443 | 0.555449 |
| CNV8 | 0.719374 | 0.408267 | 0.448278 | 0.410719 | 0.496591 | 1 | 0.401906 | 0.669824 | 0.662901 | 0.59797 | 0.720835 | 1.441459 |
| CNV9 | 1.000003 | 0.925332 | 0.476123 | 1.120923 | 0.977158 | 0.452385 | 0.867421 | 1.063825 | 1.079904 | 1.073442 | 1.03104 | 0.99504 |
| CNV10 | 0.827392 | 0.641227 | 0.740128 | 0.999994 | 0.629078 | 0.386737 | 0.717547 | 0.442345 | 0.839672 | 1.072547 | 0.933398 | 0.490519 |
| CNV11 | 0.876561 | 1.044094 | 0.739271 | 1.063305 | 0.74782 | 0.999994 | 1.076019 | 0.883588 | 0.908506 | 0.912906 | 1.07421 | 0.875372 |
| CNV12 | 0.673183 | 0.58305 | 2.857274 | 0.298324 | 0.324573 | 0.620386 | 0.151708 | 0.364984 | 0.531046 | 10.30891 | 0.249013 | 1.000002 |
| CNV13 | 0.999986 | 1.095471 | 0.962493 | 1.141158 | 0.583002 | 1.420914 | 0.733032 | 0.892701 | 0.743894 | 0.841602 | 0.708424 | 0.451716 |
| CNV14 | 1.397134 | 1.091861 | 1.11623 | 1.424602 | 0.999987 | 1.01198 | 0.941289 | 0.673222 | 1.399404 | 1.226078 | 0.988975 | 1.151028 |
| CNV15 | 1.286106 | 0.51342 | 0.510082 | 14.17577 | 0.99999 | 0.591415 | 6.309261 | 1.181719 | 0.665377 | 0.394686 | 11.36558 | 1.793754 |

**Table S5. Overlapped CNVs detected by CNVnator.**

| **Individual** | **Chr** | **Start** | **End** | **RD** |
| --- | --- | --- | --- | --- |
| l1 | 1 | 241838501 | 242510500 | 2.8736 |
| m6 | 1 | 242306001 | 242509700 | 1.97324 |
| m4 | 1 | 242335701 | 242508600 | 1.67295 |
| m3 | 1 | 242335701 | 242509600 | 1.51499 |
| m5 | 1 | 242336001 | 242510000 | 1.66542 |
| m7 | 1 | 242336001 | 242510300 | 1.92222 |
| m1 | 1 | 242336101 | 242510200 | 1.86978 |
| l4 | 1 | 242336201 | 242509700 | 2.38345 |
| m2 | 1 | 242336301 | 242510700 | 1.96506 |
| m8 | 1 | 242336801 | 242509700 | 1.27762 |
| l2 | 1 | 242346501 | 242509300 | 1.48997 |
| l2 | 1 | 242509301 | 242545500 | 0.437882 |
| m6 | 1 | 242509701 | 242524300 | 0.228589 |
| m4 | 1 | 242509701 | 242525000 | 0.21514 |
| m8 | 1 | 242509701 | 242546400 | 0.562628 |
| m5 | 1 | 242510001 | 242524400 | 0.283094 |
| m1 | 1 | 242510201 | 242546800 | 0.643453 |
| l1 | 1 | 242510501 | 242524700 | 0.278654 |
| m4 | 3 | 94656401 | 94834700 | 1.73664 |
| m3 | 3 | 94656401 | 94839600 | 1.47927 |
| m7 | 3 | 94656401 | 94840100 | 2.04122 |
| m1 | 3 | 94656401 | 94840200 | 1.77615 |
| m8 | 3 | 94656401 | 95088300 | 1.30665 |
| l2 | 3 | 94656401 | 95089800 | 1.47185 |
| l4 | 3 | 94656401 | 95089800 | 2.22911 |
| m6 | 3 | 94656401 | 95123700 | 2.18504 |
| m2 | 3 | 94656801 | 94840400 | 1.87347 |
| l1 | 3 | 94681401 | 95129200 | 2.62327 |
| m5 | 3 | 94709401 | 94755700 | 1.60412 |
| m5 | 3 | 94759001 | 95089900 | 1.62689 |
| m7 | 3 | 94840101 | 94846900 | 0.070517 |
| m1 | 3 | 94844501 | 94847100 | 0.446199 |
| m7 | 3 | 94846901 | 95089400 | 2.03258 |
| m1 | 3 | 94847101 | 94856700 | 1.96575 |
| m2 | 3 | 94847401 | 95089500 | 1.96228 |
| m4 | 3 | 94847801 | 95089600 | 1.74402 |
| m1 | 3 | 94859101 | 95090000 | 1.7282 |
| m3 | 3 | 94859901 | 94956800 | 1.51983 |
| m4 | 3 | 96126801 | 96302800 | 1.67013 |
| m4 | 3 | 96308501 | 96358900 | 0.000739 |
| m4 | 3 | 96358901 | 96490800 | 1.63148 |
| m4 | 3 | 133816701 | 134024400 | 2.06716 |
| m5 | 4 | 9607201 | 9672600 | 1.64415 |
| m7 | 4 | 96186901 | 96363500 | 2.01439 |
| l2 | 4 | 96187101 | 96369100 | 1.3338 |
| m2 | 4 | 96187201 | 96393000 | 2.21166 |
| m6 | 4 | 96187601 | 96367300 | 2.14302 |
| l1 | 4 | 96187901 | 96393100 | 3.17496 |
| l4 | 4 | 96188101 | 96393100 | 2.36553 |
| m1 | 4 | 96188501 | 96377100 | 2.08669 |
| m3 | 4 | 96236701 | 96368800 | 1.5399 |
| l2 | 4 | 96369101 | 96468000 | 0.288685 |
| m8 | 4 | 96369401 | 96468100 | 0.306846 |
| m3 | 4 | 96393101 | 96444600 | 0.008174 |
| m6 | 4 | 133534201 | 133925000 | 2.35566 |
| m1 | 4 | 133534201 | 133962500 | 2.32222 |
| l1 | 4 | 133534301 | 134023300 | 3.26544 |
| m7 | 4 | 133534401 | 133924800 | 2.1409 |
| m3 | 4 | 133717001 | 133970500 | 1.62317 |
| m2 | 4 | 133796601 | 134011700 | 2.57564 |
| m8 | 4 | 133846201 | 133855700 | 1.41811 |
| m5 | 4 | 133857301 | 133899000 | 2.17399 |
| l4 | 4 | 133885501 | 134417700 | 2.79031 |
| l2 | 4 | 133908201 | 133925300 | 1.33355 |
| m5 | 4 | 133909701 | 134010700 | 1.90283 |
| m6 | 4 | 133929101 | 134419700 | 2.643 |
| m7 | 4 | 133929701 | 134417700 | 2.38392 |
| l2 | 4 | 133942401 | 133961100 | 1.28265 |
| l4 | 5 | 21235401 | 21399000 | 3.21603 |
| m4 | 5 | 21235401 | 21402100 | 3.18102 |
| m7 | 5 | 21235401 | 21402100 | 4.15062 |
| m2 | 5 | 21235401 | 21403800 | 4.1255 |
| l1 | 5 | 21235401 | 21404100 | 3.90177 |
| m8 | 5 | 21235401 | 21405900 | 3.21124 |
| m1 | 5 | 21235401 | 21447100 | 3.65589 |
| m5 | 5 | 21235401 | 21463200 | 4.18643 |
| m6 | 5 | 21235401 | 21620200 | 3.93882 |
| l4 | 5 | 21403801 | 21420800 | 0.122126 |
| m4 | 5 | 21403901 | 21420800 | 0.154148 |
| m7 | 5 | 21403901 | 21420900 | 0.219489 |
| l1 | 5 | 21404101 | 21454800 | 0.325838 |
| l2 | 5 | 21404201 | 21420900 | 0.151258 |
| m4 | 5 | 21420801 | 21440200 | 2.78322 |
| m7 | 5 | 21420901 | 21439200 | 3.06609 |
| m2 | 5 | 21420901 | 21442200 | 2.14055 |
| m8 | 5 | 21421201 | 21442100 | 2.54389 |
| l4 | 5 | 21423601 | 21454400 | 0.343163 |
| l2 | 5 | 21423701 | 21476200 | 0.302813 |
| m4 | 5 | 21440201 | 21467500 | 0.524951 |
| m2 | 5 | 21442201 | 21506000 | 0.474902 |
| m1 | 5 | 21452801 | 21508900 | 1.948 |
| l1 | 5 | 21472101 | 21614500 | 1.76657 |
| m5 | 5 | 21473601 | 21499400 | 1.884 |
| m8 | 5 | 21476201 | 21482200 | 1.80266 |
| m8 | 5 | 21484801 | 21511000 | 0.712798 |
| m5 | 5 | 21499401 | 21501700 | 0.24045 |
| l2 | 5 | 21499601 | 21510700 | 0.425909 |
| m7 | 5 | 21501701 | 21522500 | 2.16685 |
| m5 | 5 | 21501701 | 21619900 | 2.05583 |
| m2 | 5 | 21506001 | 21536200 | 1.8554 |
| m4 | 5 | 21510501 | 21549500 | 1.76755 |
| l4 | 5 | 21510601 | 21542500 | 1.4969 |
| m1 | 5 | 21511001 | 21517100 | 2.51676 |
| m8 | 5 | 21511001 | 21573300 | 1.85299 |
| l2 | 5 | 21517601 | 21676300 | 0.494301 |
| m1 | 5 | 21526601 | 21573300 | 2.22418 |
| m7 | 5 | 21527201 | 21619900 | 1.65572 |
| m2 | 5 | 21543201 | 21604100 | 0.711193 |
| m1 | 5 | 21589701 | 21623400 | 2.3145 |
| m8 | 5 | 21596401 | 21619500 | 1.60044 |
| m4 | 5 | 21602101 | 21612900 | 1.65454 |
| m8 | 5 | 21619501 | 21673800 | 0.055654 |
| m4 | 5 | 21619701 | 21675600 | 0.02539 |
| l1 | 5 | 21619801 | 21673800 | 0.049461 |
| m7 | 5 | 21619901 | 21674400 | 0.029993 |
| m5 | 5 | 21619901 | 21674400 | 0.054802 |
| m6 | 5 | 21620201 | 21674000 | 0.057163 |
| l4 | 5 | 21620401 | 21675700 | 0.044369 |
| m2 | 5 | 21621601 | 21673800 | 0.006511 |
| m1 | 5 | 21623401 | 21673800 | 0.002302 |
| m1 | 5 | 21673801 | 21688400 | 1.85407 |
| m6 | 5 | 21674001 | 21695300 | 2.8526 |
| m7 | 5 | 21674401 | 21695500 | 2.26135 |
| m5 | 5 | 21674401 | 21695500 | 2.32799 |
| m4 | 5 | 21675601 | 21688300 | 2.12698 |
| m2 | 5 | 21675701 | 21778200 | 1.6741 |
| m8 | 5 | 21676701 | 21691000 | 1.95897 |
| l2 | 5 | 21683501 | 21751400 | 0.248468 |
| m4 | 5 | 21688301 | 21743900 | 0.744278 |
| m1 | 5 | 21688401 | 21712600 | 0.620007 |
| l1 | 5 | 21693901 | 21832800 | 0.581854 |
| l4 | 5 | 21694001 | 21833000 | 0.454876 |
| m8 | 5 | 21695501 | 21743600 | 0.604409 |
| m6 | 5 | 21727301 | 21856400 | 1.9532 |
| m7 | 5 | 21743901 | 21754800 | 2.64125 |
| m4 | 5 | 21743901 | 21774600 | 1.64445 |
| m5 | 5 | 21744001 | 21770100 | 2.18648 |
| m1 | 5 | 21750101 | 21770400 | 1.77923 |
| m8 | 5 | 21750301 | 21754900 | 2.56045 |
| l2 | 5 | 21754901 | 21782300 | 0.206371 |
| m8 | 5 | 21754901 | 21827500 | 0.720301 |
| m4 | 5 | 21774601 | 21803000 | 0.260773 |
| m1 | 5 | 21783501 | 21786600 | 1.84621 |
| l2 | 5 | 21787901 | 21931700 | 0.255918 |
| m2 | 5 | 21794601 | 21857900 | 1.95603 |
| m5 | 5 | 21802801 | 21857900 | 1.78637 |
| m4 | 5 | 21803001 | 21854100 | 1.53683 |
| m7 | 5 | 21804301 | 21854100 | 1.88272 |
| m8 | 5 | 21827501 | 21856400 | 1.30568 |
| m1 | 5 | 21832601 | 21854100 | 2.27887 |
| m1 | 5 | 21854101 | 21907900 | 0.033064 |
| m4 | 5 | 21854101 | 21908200 | 0.024556 |
| m7 | 5 | 21854101 | 21908200 | 0.049771 |
| l4 | 5 | 21856001 | 21931600 | 0.184745 |
| m8 | 5 | 21856401 | 21908000 | 0.017028 |
| m6 | 5 | 21856401 | 21908200 | 0.025214 |
| l1 | 5 | 21857801 | 21931300 | 0.209957 |
| m2 | 5 | 21857901 | 21908000 | 0.000969 |
| m5 | 5 | 21857901 | 21908400 | 0.002665 |
| m2 | 5 | 21908001 | 22082800 | 3.8089 |
| m4 | 5 | 21908201 | 21916100 | 1.84845 |
| m6 | 5 | 21908201 | 22083200 | 4.5319 |
| m7 | 5 | 21908201 | 22239700 | 3.42844 |
| m5 | 5 | 21908401 | 22079600 | 3.98747 |
| m8 | 5 | 21916401 | 21931700 | 0.655948 |
| l1 | 5 | 21931301 | 22078600 | 4.0771 |
| m1 | 5 | 21931401 | 22078000 | 3.36481 |
| m4 | 5 | 21931501 | 22168800 | 2.62232 |
| l4 | 5 | 21931601 | 22077200 | 3.29992 |
| m8 | 5 | 21931701 | 22000900 | 3.64392 |
| l2 | 5 | 21931701 | 22026300 | 2.44606 |
| m8 | 5 | 22004701 | 22111000 | 2.00375 |
| l2 | 5 | 22078401 | 22087000 | 0.557804 |
| m2 | 5 | 22085501 | 22169100 | 2.57182 |
| l1 | 5 | 22085601 | 22112200 | 2.18298 |
| m5 | 5 | 22085601 | 22242900 | 2.77968 |
| m1 | 5 | 22085801 | 22107900 | 2.44447 |
| l4 | 5 | 22085801 | 22111900 | 2.02479 |
| m6 | 5 | 22085901 | 22241400 | 3.37045 |
| l4 | 5 | 22111901 | 22162300 | 0.55049 |
| l1 | 5 | 22112201 | 22152300 | 0.525447 |
| l2 | 5 | 22112701 | 22159700 | 0.352055 |
| m1 | 5 | 22135001 | 22181400 | 2.64693 |
| l1 | 5 | 22152301 | 22233000 | 2.90393 |
| l4 | 5 | 22162301 | 22168800 | 2.01322 |
| m8 | 5 | 22169201 | 22175400 | 0.676218 |
| m8 | 5 | 22175401 | 22221100 | 2.41787 |
| m4 | 5 | 22175501 | 22231300 | 2.65508 |
| m2 | 5 | 22175701 | 22181100 | 2.81443 |
| l4 | 5 | 22179501 | 22232500 | 3.03882 |
| m2 | 5 | 22184601 | 22231400 | 3.27439 |
| m1 | 5 | 22184801 | 22227000 | 4.30233 |
| l2 | 5 | 22188301 | 22219100 | 2.00218 |
| l2 | 5 | 22227001 | 22339500 | 0.259142 |
| m8 | 5 | 22229701 | 22293600 | 0.171113 |
| l4 | 5 | 22232501 | 22311100 | 0.196904 |
| l1 | 5 | 22233001 | 22312400 | 0.260387 |
| m4 | 5 | 22239401 | 22293600 | 0.028238 |
| m7 | 5 | 22239701 | 22293700 | 0.048389 |
| m1 | 5 | 22241301 | 22293300 | 0.02231 |
| m6 | 5 | 22241401 | 22293300 | 0.026036 |
| l3 | 5 | 22242501 | 22294500 | 0 |
| m5 | 5 | 22242901 | 22293600 | 0.004066 |
| m2 | 5 | 22243101 | 22293700 | 0.002879 |
| m6 | 5 | 22293301 | 22575500 | 3.16538 |
| m5 | 5 | 22293601 | 22300500 | 1.7019 |
| m5 | 5 | 22300501 | 22304900 | 0.526291 |
| m4 | 5 | 22301301 | 22304800 | 0.150041 |
| m1 | 5 | 22304301 | 22575200 | 2.46527 |
| m5 | 5 | 22304901 | 22330600 | 1.68368 |
| m7 | 5 | 22310601 | 22575500 | 2.17761 |
| l1 | 5 | 22312401 | 22389600 | 1.62863 |
| m8 | 5 | 22314101 | 22320400 | 0.493419 |
| m5 | 5 | 22330601 | 22332700 | 0.254695 |
| m5 | 5 | 22332701 | 22575100 | 2.47358 |
| m2 | 5 | 22338201 | 22575300 | 2.09213 |
| m4 | 5 | 22339901 | 22371300 | 1.77052 |
| m8 | 5 | 22340801 | 22373800 | 2.04357 |
| l4 | 5 | 22341001 | 22387100 | 1.64584 |
| m4 | 5 | 22376701 | 22388300 | 1.90264 |
| l2 | 5 | 22385401 | 22427400 | 0.603088 |
| m4 | 5 | 22397901 | 22575300 | 1.8603 |
| l1 | 5 | 22410701 | 22519500 | 2.74127 |
| m8 | 5 | 22427401 | 22573600 | 1.60865 |
| m4 | 5 | 60470201 | 61145800 | 1.60118 |
| m1 | 5 | 60470201 | 61145800 | 1.93902 |
| l4 | 5 | 60470201 | 61145800 | 2.33326 |
| l1 | 5 | 60470201 | 61145900 | 2.91848 |
| m6 | 5 | 60765201 | 61145700 | 2.22174 |
| m2 | 5 | 60767501 | 61145900 | 2.20887 |
| m5 | 5 | 60767901 | 60981800 | 1.74003 |
| m7 | 5 | 60768101 | 60969600 | 1.96528 |
| l2 | 5 | 60777301 | 61145800 | 1.39173 |
| m3 | 5 | 60819501 | 61145900 | 1.55559 |
| m7 | 5 | 60971201 | 61145900 | 2.09292 |
| m5 | 5 | 60988001 | 61026100 | 1.82729 |
| l1 | 5 | 78855301 | 79504100 | 3.0253 |
| l2 | 5 | 78858501 | 79504100 | 1.36207 |
| m1 | 5 | 78858601 | 79504100 | 1.98199 |
| m2 | 5 | 78881701 | 79504100 | 2.17986 |
| m6 | 5 | 78883501 | 79504100 | 2.22166 |
| l4 | 5 | 78883501 | 79504100 | 2.34409 |
| m7 | 5 | 78883701 | 79504100 | 2.03553 |
| m5 | 5 | 78898701 | 79504100 | 1.73691 |
| m4 | 5 | 78913201 | 79504100 | 1.62982 |
| m8 | 5 | 79332801 | 79504100 | 1.27571 |
| l1 | 5 | 79504101 | 79554100 | 0 |
| m2 | 5 | 79504101 | 79554100 | 0 |
| m3 | 5 | 79504101 | 79554100 | 0 |
| m4 | 5 | 79504101 | 79554100 | 0 |
| m5 | 5 | 79504101 | 79554100 | 0 |
| m6 | 5 | 79504101 | 79554100 | 0 |
| m7 | 5 | 79504101 | 79554100 | 0 |
| m8 | 5 | 79504101 | 79554100 | 0 |
| l4 | 5 | 79504101 | 79554100 | 0.001258 |
| l2 | 5 | 79504101 | 79554200 | 0.000925 |
| m1 | 5 | 79504101 | 79554200 | 0.001544 |
| m8 | 5 | 79554101 | 79564600 | 1.26506 |
| m5 | 5 | 79554101 | 79565600 | 1.89284 |
| m3 | 5 | 79554101 | 79786200 | 1.54527 |
| m4 | 5 | 79554101 | 79791500 | 1.78149 |
| m7 | 5 | 79554101 | 79794400 | 2.0818 |
| m2 | 5 | 79554101 | 79794600 | 2.06054 |
| m6 | 5 | 79554101 | 80064900 | 2.25224 |
| l1 | 5 | 79554101 | 80076800 | 3.02104 |
| l4 | 5 | 79554101 | 80234400 | 2.32103 |
| m1 | 5 | 79554201 | 79735800 | 1.82216 |
| l2 | 5 | 79575501 | 79794100 | 1.4739 |
| m5 | 5 | 79579901 | 80034000 | 1.76765 |
| m1 | 5 | 79744001 | 79794600 | 1.78068 |
| m1 | 5 | 79803401 | 80034200 | 2.17128 |
| l2 | 5 | 79806101 | 80031700 | 1.33027 |
| m7 | 5 | 79806901 | 80034000 | 2.08511 |
| m2 | 5 | 79806901 | 80034200 | 2.36861 |
| m1 | 7 | 11958701 | 12370000 | 2.7375 |
| m2 | 7 | 121822301 | 121925700 | 2.30945 |
| l1 | 7 | 121823101 | 121925700 | 3.61461 |
| l4 | 7 | 121823701 | 121925700 | 2.66579 |
| m3 | 7 | 121831801 | 121925700 | 1.49252 |
| m7 | 7 | 121892001 | 121925700 | 2.1666 |
| m4 | 7 | 121905601 | 121925700 | 2.04274 |
| m5 | 7 | 121905601 | 121925700 | 2.20576 |
| m6 | 7 | 121905601 | 121925700 | 2.83289 |
| l2 | 7 | 121920401 | 121983800 | 0.107465 |
| l1 | 7 | 121925701 | 121975700 | 0 |
| l4 | 7 | 121925701 | 121975700 | 0 |
| m2 | 7 | 121925701 | 121975700 | 0 |
| m5 | 7 | 121925701 | 121976100 | 0.004153 |
| m6 | 7 | 121925701 | 121977100 | 0.018006 |
| m7 | 7 | 121925701 | 121977500 | 0.025089 |
| m3 | 7 | 121925701 | 121983500 | 0.098041 |
| m8 | 7 | 121925701 | 121984000 | 0.066741 |
| m4 | 7 | 121925701 | 121984000 | 0.100833 |
| l1 | 7 | 121975701 | 122030400 | 3.69647 |
| m2 | 7 | 121975701 | 122052500 | 2.54285 |
| m6 | 7 | 121983201 | 122055500 | 2.27251 |
| m3 | 7 | 121983501 | 122030500 | 1.86573 |
| m5 | 7 | 121983601 | 121988800 | 2.37421 |
| l4 | 7 | 121983901 | 121992800 | 2.6367 |
| m8 | 7 | 121984001 | 121993000 | 1.33493 |
| m4 | 7 | 121984001 | 122030900 | 1.80985 |
| l2 | 7 | 121992701 | 122001800 | 0.646848 |
| m8 | 7 | 121993001 | 121998700 | 0.249026 |
| m5 | 7 | 121998701 | 122043800 | 2.0859 |
| l4 | 7 | 121999801 | 122030200 | 3.14566 |
| l2 | 7 | 122001801 | 122020300 | 1.25768 |
| m6 | 10 | 9058501 | 9542300 | 2.25204 |
| m1 | 10 | 9069901 | 9540800 | 2.33715 |
| l1 | 10 | 9080501 | 9542200 | 3.4356 |
| m7 | 10 | 9081001 | 9541700 | 2.1639 |
| m5 | 10 | 9081201 | 9542200 | 1.94112 |
| l4 | 10 | 9084501 | 9541200 | 2.61419 |
| m4 | 10 | 9086001 | 9542200 | 1.63092 |
| m2 | 10 | 9086001 | 9542200 | 2.57615 |
| m3 | 10 | 9086401 | 9542000 | 1.61716 |
| l2 | 10 | 9100401 | 9542200 | 1.3047 |
| m7 | 10 | 48811101 | 49305200 | 2.12213 |
| m2 | 10 | 48811101 | 49306000 | 2.39982 |
| m6 | 10 | 48884401 | 49306000 | 2.24334 |
| m1 | 10 | 48885901 | 49305800 | 2.1657 |
| l1 | 10 | 48885901 | 49306100 | 3.07572 |
| l4 | 10 | 48886301 | 49305600 | 2.31052 |
| l2 | 10 | 48934501 | 49180100 | 1.29866 |
| m3 | 10 | 48935501 | 49233000 | 1.54321 |
| m5 | 10 | 48936201 | 49296200 | 1.82005 |
| m4 | 10 | 48995401 | 49184900 | 1.594 |
| m4 | 10 | 48995401 | 49184900 | 1.594 |
| l2 | 10 | 49190701 | 49305700 | 1.31812 |
| m4 | 10 | 49195401 | 49305900 | 1.63847 |
| m4 | 10 | 49195401 | 49305900 | 1.63847 |
| m3 | 10 | 49253501 | 49305900 | 1.48436 |
| l4 | 11 | 11269501 | 11709300 | 2.13244 |
| l4 | 11 | 11717601 | 11751400 | 2.46999 |
| l1 | 12 | 11127801 | 11498200 | 2.56813 |
| m2 | 12 | 11353501 | 11490400 | 2.03941 |
| m7 | 12 | 11421901 | 11498200 | 1.98422 |
| m3 | 12 | 11440501 | 11490300 | 1.59437 |
| m4 | 12 | 11440501 | 11498100 | 1.68388 |
| m6 | 12 | 11440501 | 11498200 | 2.35348 |
| l2 | 12 | 11440601 | 11489600 | 1.45668 |
| m5 | 12 | 11440601 | 11498200 | 1.71136 |
| m1 | 12 | 11441301 | 11498200 | 2.03615 |
| m8 | 12 | 11441601 | 11490100 | 1.27964 |
| m8 | 12 | 11490101 | 11493900 | 0.517438 |
| m2 | 12 | 11494601 | 11498100 | 2.77461 |
| m8 | 12 | 11498001 | 11654200 | 0.302813 |
| m4 | 12 | 11498101 | 11647600 | 0.368856 |
| l2 | 12 | 11498101 | 11649600 | 0.291249 |
| m2 | 12 | 11498101 | 11649600 | 0.453827 |
| m1 | 12 | 11498201 | 11568100 | 0.170394 |
| m5 | 12 | 11498201 | 11568900 | 0.163142 |
| m6 | 12 | 11498201 | 11568900 | 0.164902 |
| m7 | 12 | 11498201 | 11632700 | 0.414929 |
| m3 | 12 | 11498201 | 11647800 | 0.320569 |
| l1 | 12 | 11516901 | 11568300 | 0.005774 |
| m5 | 12 | 11576701 | 11649100 | 0.636588 |
| m1 | 12 | 11591901 | 11648900 | 0.583451 |
| m6 | 12 | 11600401 | 11611700 | 0.456113 |
| l1 | 12 | 11639401 | 11659300 | 2.13254 |
| m7 | 12 | 11648701 | 11754400 | 1.52605 |
| m5 | 12 | 11649101 | 11658900 | 1.79694 |
| m6 | 12 | 11649101 | 11659700 | 2.10812 |
| m2 | 12 | 11649601 | 11659700 | 2.09126 |
| m4 | 12 | 11650001 | 11660000 | 1.57694 |
| m1 | 12 | 11651001 | 11660000 | 1.92923 |
| m1 | 12 | 11660001 | 11665900 | 0.498129 |
| m8 | 12 | 11660001 | 11678500 | 0.473433 |
| l2 | 12 | 11660001 | 11678800 | 0.488143 |
| m4 | 12 | 11660001 | 11679300 | 0.705104 |
| m1 | 12 | 11673701 | 11678800 | 0.348793 |
| m6 | 12 | 11678301 | 11713000 | 1.8412 |
| m2 | 12 | 11678801 | 11699300 | 1.47799 |
| l1 | 12 | 11679001 | 11812100 | 1.7421 |
| m1 | 12 | 11690801 | 11706200 | 1.5418 |
| m2 | 12 | 11699301 | 11708400 | 0.661637 |
| m1 | 12 | 11706201 | 11708600 | 0.096676 |
| m2 | 12 | 11708401 | 11713400 | 2.01991 |
| m1 | 12 | 11708601 | 11758200 | 1.57957 |
| m4 | 12 | 11712401 | 11721100 | 0.596412 |
| m8 | 12 | 11712501 | 11722100 | 0.495107 |
| m1 | 12 | 56496701 | 57072600 | 3.04281 |
| l1 | 12 | 56498001 | 57072600 | 4.12526 |
| m2 | 12 | 56504501 | 57072600 | 3.22573 |
| m7 | 12 | 56506701 | 57072600 | 2.39926 |
| l4 | 12 | 56516901 | 57072600 | 3.22649 |
| m6 | 12 | 56523501 | 57072600 | 2.72209 |
| m5 | 12 | 56523701 | 57072600 | 2.16106 |
| m3 | 12 | 56528401 | 56936100 | 2.02049 |
| m4 | 12 | 56645101 | 56936600 | 1.94565 |
| l2 | 12 | 56933101 | 56973800 | 0.558579 |
| m8 | 12 | 56936001 | 56973700 | 0.600424 |
| m4 | 12 | 56972701 | 57072600 | 2.16634 |
| m3 | 12 | 56973701 | 57072600 | 2.49492 |
| m2 | 17 | 41575001 | 42324300 | 2.90491 |
| m5 | 17 | 41575001 | 42324700 | 2.03025 |
| m1 | 17 | 41575001 | 42326900 | 2.69912 |
| l1 | 17 | 41575001 | 42326900 | 3.72113 |
| m6 | 17 | 41578701 | 42324800 | 2.49601 |
| m3 | 17 | 41580401 | 42322600 | 1.7213 |
| m7 | 17 | 41616301 | 42324700 | 2.35231 |
| m4 | 17 | 41620601 | 42323000 | 1.73038 |
| l1 | 18 | 22774101 | 23433100 | 2.72164 |
| m6 | 18 | 23368101 | 23471500 | 1.83133 |
| l2 | 18 | 23377801 | 23416200 | 1.52203 |
| m1 | 18 | 23377801 | 23417500 | 1.71339 |
| m3 | 18 | 23377801 | 23418500 | 1.47189 |
| m4 | 18 | 23377901 | 23417400 | 1.71797 |
| m5 | 18 | 23378001 | 23417500 | 1.57285 |
| m2 | 18 | 23378001 | 23417500 | 1.91887 |
| l4 | 18 | 23378101 | 23417500 | 2.26104 |
| m7 | 18 | 23381601 | 23417500 | 2.08801 |
| m1 | 18 | 23417501 | 23424800 | 0.720438 |
| m3 | 18 | 23418501 | 23442400 | 0.742473 |
| m5 | 18 | 23421301 | 23442500 | 0.675203 |
| m8 | 18 | 23422201 | 23442300 | 0.640142 |
| m1 | 18 | 23424801 | 23451600 | 1.2972 |
| l4 | 18 | 23438901 | 23471800 | 1.75162 |
| l2 | 18 | 23442301 | 23453800 | 1.54161 |
| m8 | 18 | 23442301 | 23453900 | 1.367 |
| m3 | 18 | 23442401 | 23453800 | 1.42877 |
| m2 | 18 | 23442501 | 23453600 | 1.99472 |
| m5 | 18 | 23442501 | 23473100 | 1.25762 |
| m4 | 18 | 23442601 | 23451900 | 1.58869 |
| l1 | 18 | 23442601 | 23473700 | 2.217 |
| m7 | 18 | 23443201 | 23471600 | 1.68589 |
| m8 | 18 | 23453901 | 23457400 | 0.368256 |
| m2 | 18 | 23461101 | 23471500 | 1.86286 |
| m1 | 18 | 23461901 | 23471600 | 1.7342 |
| l2 | 18 | 23465801 | 23471300 | 1.55901 |
| m3 | 18 | 23471301 | 23505600 | 0.727877 |
| m8 | 18 | 23471501 | 23492800 | 0.632854 |
| m5 | 18 | 23473101 | 23493000 | 0.714708 |
| l2 | 18 | 23477101 | 23506400 | 0.682579 |
| m8 | 18 | 23496101 | 23505600 | 0.4808 |
| m3 | 18 | 23505601 | 23515400 | 1.45387 |
| l4 | 18 | 23505801 | 23546300 | 2.35808 |
| l2 | 18 | 23506401 | 23515600 | 1.62977 |
| m7 | 18 | 23506901 | 23514500 | 2.2921 |
| m6 | 18 | 23507001 | 23515200 | 2.21585 |
| l1 | 18 | 23507301 | 24127800 | 2.61008 |
| m4 | 18 | 23507401 | 23546400 | 1.66829 |
| m2 | 18 | 23508501 | 23544900 | 1.92755 |
| m3 | 18 | 23517101 | 23545900 | 1.5786 |
| m1 | 18 | 23517401 | 23545700 | 1.83241 |
| m6 | 18 | 23518201 | 23546300 | 2.24896 |
| m7 | 18 | 23518701 | 23545200 | 2.02523 |
| m5 | 18 | 23518701 | 23546300 | 1.71605 |
| l2 | 18 | 23519301 | 23546300 | 1.50634 |
| m1 | 18 | 23545701 | 23559900 | 0.520146 |
| m8 | 18 | 23546301 | 23560100 | 0.584025 |
| l2 | 18 | 23546301 | 23560400 | 0.67058 |
| m4 | 18 | 23549901 | 23559900 | 0.372524 |
| m5 | 18 | 23551901 | 23560100 | 0.223491 |
| m3 | 18 | 23553501 | 23559700 | 0.24025 |
| m3 | 18 | 23559701 | 23615100 | 1.40807 |
| m4 | 18 | 23559901 | 23604300 | 1.57667 |
| m1 | 18 | 23559901 | 23614300 | 1.60085 |
| m7 | 18 | 23559901 | 23614400 | 1.96732 |
| m2 | 18 | 23559901 | 23629300 | 2.0031 |
| l4 | 18 | 23559901 | 23861300 | 2.28608 |
| m5 | 18 | 23560101 | 23614400 | 1.46132 |
| m6 | 18 | 23560201 | 23615400 | 2.21018 |
| l2 | 18 | 23560401 | 23631400 | 1.49753 |
| m4 | 18 | 23606101 | 23614300 | 1.67274 |
| m8 | 18 | 23606701 | 23632300 | 1.30024 |
| m4 | 18 | 23614301 | 23637800 | 0.024471 |
| m1 | 18 | 23614301 | 23637900 | 0.01147 |
| m7 | 18 | 23614401 | 23637800 | 0.027703 |
| m5 | 18 | 23614401 | 23637900 | 0.024503 |
| m3 | 18 | 23615101 | 23637800 | 0.610542 |
| l1 | 18 | 46545701 | 47777100 | 3.43472 |
| m1 | 18 | 46546001 | 47088700 | 2.39071 |
| m6 | 18 | 46596601 | 47206900 | 2.33046 |
| m2 | 18 | 46742101 | 46810800 | 2.05743 |
| m7 | 18 | 46747801 | 46954000 | 2.01596 |
| l2 | 18 | 46748801 | 46783100 | 1.28574 |
| m3 | 18 | 46749801 | 46785600 | 1.724 |
| m4 | 18 | 46749901 | 46783700 | 1.62146 |
| l4 | 18 | 46749901 | 46958300 | 2.30098 |
| m5 | 18 | 46753901 | 46940500 | 1.78839 |
| l2 | 18 | 46783101 | 46799000 | 0.638391 |
| m8 | 18 | 46784001 | 46799200 | 0.600837 |
| l2 | 18 | 46799001 | 46805700 | 1.61184 |
| m3 | 18 | 46799101 | 46807900 | 1.87298 |
| m4 | 18 | 46800401 | 46807900 | 2.12552 |
| m8 | 18 | 46810501 | 46821200 | 0.734189 |
| m4 | 18 | 46821801 | 46882500 | 1.73052 |
| l2 | 18 | 46821901 | 46846600 | 1.48617 |
| m3 | 18 | 46822001 | 46954300 | 1.56915 |
| m2 | 18 | 46823701 | 47088300 | 2.46962 |
| l2 | 18 | 46854901 | 46954100 | 1.38626 |
| m4 | 18 | 46888301 | 46953900 | 1.72641 |
| m5 | 18 | 46946501 | 46954100 | 2.42421 |
| l2 | 18 | 46954101 | 46969800 | 0.667188 |
| m8 | 18 | 46954401 | 46969800 | 0.571494 |
| m7 | 18 | 46969401 | 47206800 | 2.23955 |
| m5 | 18 | 46969701 | 47088800 | 2.05736 |
| l2 | 18 | 46969801 | 47013200 | 1.39207 |
| l4 | 18 | 46969801 | 47776700 | 2.59465 |
| m4 | 18 | 46970401 | 47206800 | 1.68438 |
| m3 | 18 | 46970601 | 46974400 | 2.20706 |
| m3 | 18 | 46976301 | 47088600 | 1.72461 |

**Table S6. Association of CNVs with meat quality traits.**

| **Predictor** | **Chr** | **Position** | **FDR** | | | | | | | | | | | |
| --- | --- | --- | --- | --- | --- | --- | --- | --- | --- | --- | --- | --- | --- | --- |
|  |  |  | **IMF** | **Marbling** | **Moisture** | **SFN** | **pH6** | **a6*** | **a24*** | **L6*** | **L24*** | **b6*** | **b24*** | **Ph24** |
| ASGA0000014 | 1 | 342481 | ? | ? | ? | ? | ? | ? | ? | ? | ? | ? | ? | ? |
| DRGA0001965 | 1 | 242457549 | 0.0435 | 0.058624 | 1 | 1 | 0.140067 | 0.685539 | 0.797521 | 1 | 1 | 0.734529 | 0.960842 | 0.942211 |
| INRA0005996 | 1 | 242534196 | ? | ? | ? | ? | ? | ? | ? | ? | ? | ? | ? | ? |
| MARC0033927 | 2 | 211101 | ? | ? | ? | ? | ? | ? | ? | ? | ? | ? | ? | ? |
| M1GA0024177 | 3 | 1615014 | ? | ? | ? | ? | ? | ? | ? | ? | ? | ? | ? | ? |
| MARC0107032 | 3 | 94706101 | 0.356045 | 0.584762 | 1 | 0.963412 | 0.225693 | 0.62976 | 1 | 0.923251 | 1 | 0.721046 | 0.712376 | 0.880303 |
| INRA0011050 | 3 | 94887165 | ? | ? | ? | ? | ? | ? | ? | ? | ? | ? | ? | ? |
| ASGA0016959 | 4 | 95368 | ? | ? | ? | ? | ? | ? | ? | ? | ? | ? | ? | ? |
| ASGA0020714 | 4 | 96277909 | 0.994966 | 0.701142 | 1 | 0.94103 | 0.153214 | 0.319909 | 0.868221 | 0.99924 | 1 | 0.856404 | 0.659782 | 1 |
| ASGA0020720 | 4 | 96479166 | ? | ? | ? | ? | ? | ? | ? | ? | ? | ? | ? | ? |
| H3GA0014546 | 4 | 133873894 | 0.77853 | 0.625841 | 1 | 0.882446 | 0.498735 | 0.993915 | 0.882285 | 1 | 0.989133 | 0.684039 | 0.97702 | 0.907215 |
| ASGA0022785 | 4 | 133976248 | ? | ? | ? | ? | ? | ? | ? | ? | ? | ? | ? | ? |
| ASGA0027336 | 5 | 189721 | ? | ? | ? | ? | ? | ? | ? | ? | ? | ? | ? | ? |
| ASGA0025036 | 5 | 21339891 | 0.549599 | 0.358694 | 0.98397 | 0.224223 | 0.116857 | 0.278043 | 0.912893 | 1 | 1 | 0.76106 | 1 | 1 |
| ALGA0031238 | 5 | 22475419 | ? | ? | ? | ? | ? | ? | ? | ? | ? | ? | ? | ? |
| ALGA0032050 | 5 | 60936295 | 0.436037 | 0.172328 | 1 | 0.908606 | 0.843036 | 0.232638 | 0.863815 | 0.753215 | 1 | 0.81376 | 0.464794 | 0.845504 |
| ASGA0025616 | 5 | 61020546 | ? | ? | ? | ? | ? | ? | ? | ? | ? | ? | ? | ? |
| ALGA0033020 | 5 | 79366287 | 0.219085 | 0.075593 | 1 | 0.81835 | 0.210887 | 0.866031 | 0.950015 | 0.882903 | 1 | 1 | 0.143629 | 0.932737 |
| ASGA0026383 | 5 | 79848149 | ? | ? | ? | ? | ? | ? | ? | ? | ? | ? | ? | ? |
| MARC0036664 | 6 | 2085723 | ? | ? | ? | ? | ? | ? | ? | ? | ? | ? | ? | ? |
| INRA0022919 | 7 | 48748 | ? | ? | ? | ? | ? | ? | ? | ? | ? | ? | ? | ? |
| ASGA0036459 | 7 | 121924542 | 0.572235 | 0.335115 | 1 | 0.85552 | 0.138145 | 0.936638 | 0.897645 | 1 | 1 | 0.901705 | 0.009306 | 1 |
| H3GA0023254 | 7 | 122018910 | ? | ? | ? | ? | ? | ? | ? | ? | ? | ? | ? | ? |
| H3GA0055033 | 8 | 424993 | ? | ? | ? | ? | ? | ? | ? | ? | ? | ? | ? | ? |
| MARC0030884 | 9 | 92982 | ? | ? | ? | ? | ? | ? | ? | ? | ? | ? | ? | ? |
| H3GA0055101 | 10 | 54839 | ? | ? | ? | ? | ? | ? | ? | ? | ? | ? | ? | ? |
| ASGA0046181 | 10 | 9369752 | 0.027201 | 0.02834 | 1 | 0.881214 | 0.001561 | 0.626536 | 0.894388 | 1 | 1 | 0.868129 | 0.006865 | 0.770062 |
| DRGA0010231 | 10 | 9488500 | ? | ? | ? | ? | ? | ? | ? | ? | ? | ? | ? | ? |
| MARC0057581 | 10 | 49173528 | 0.048047 | 0.075045 | 1 | 0.946392 | 0.001033 | 0.190222 | 0.925502 | 1 | 1 | 0.871271 | 0.052212 | 0.921097 |
| ASGA0047949 | 10 | 49305902 | ? | ? | ? | ? | ? | ? | ? | ? | ? | ? | ? | ? |
| H3GA0030836 | 11 | 22488 | ? | ? | ? | ? | ? | ? | ? | ? | ? | ? | ? | ? |
| MARC0089921 | 12 | 190362 | ? | ? | ? | ? | ? | ? | ? | ? | ? | ? | ? | ? |
| H3GA0033471 | 12 | 11462476 | 0.127452 | 0.087693 | 1 | 0.894016 | 0.132472 | 0.732174 | 1 | 1 | 1 | 0.804488 | 0.007973 | 0.971885 |
| MARC0050019 | 12 | 11737023 | ? | ? | ? | ? | ? | ? | ? | ? | ? | ? | ? | ? |
| ALGA0067058 | 12 | 56893678 | 0.002972 | 0.005501 | 1 | 1 | 0.001239 | 0.633272 | 0.904673 | 0.887056 | 1 | 1 | 0.059764 | 0.977031 |
| M1GA0017055 | 12 | 57032666 | ? | ? | ? | ? | ? | ? | ? | ? | ? | ? | ? | ? |
| ALGA0067341 | 13 | 163156 | ? | ? | ? | ? | ? | ? | ? | ? | ? | ? | ? | ? |
| H3GA0038239 | 14 | 167845 | ? | ? | ? | ? | ? | ? | ? | ? | ? | ? | ? | ? |
| MARC0089826 | 15 | 115529 | ? | ? | ? | ? | ? | ? | ? | ? | ? | ? | ? | ? |
| H3GA0046562 | 16 | 15148 | ? | ? | ? | ? | ? | ? | ? | ? | ? | ? | ? | ? |
| ASGA0074891 | 17 | 15175 | ? | ? | ? | ? | ? | ? | ? | ? | ? | ? | ? | ? |
| MARC0062414 | 17 | 41839309 | 0.07174 | 0.06245 | 1 | 0.739915 | 0.098908 | 0.697454 | 1 | 1 | 1 | 0.847053 | 0.007387 | 0.871962 |
| ALGA0094846 | 17 | 41888162 | ? | ? | ? | ? | ? | ? | ? | ? | ? | ? | ? | ? |
| ASGA0050035 | 18 | 24285 | ? | ? | ? | ? | ? | ? | ? | ? | ? | ? | ? | ? |
| ALGA0097445 | 18 | 23383197 | 0.719386 | 0.45325 | 1 | 0.120318 | 0.147004 | 0.155802 | 1 | 0.965296 | 1 | 0.623885 | 0.993642 | 1 |
| CASI0005720 | 18 | 23648007 | ? | ? | ? | ? | ? | ? | ? | ? | ? | ? | ? | ? |
| ASGA0079816 | 18 | 46776812 | 0.161685 | 0.160925 | 1 | 0.891263 | 0.011022 | 0.235447 | 0.971689 | 0.968327 | 1 | 0.896558 | 0.601844 | 0.891348 |
| CASI0007635 | 18 | 47086284 | ? | ? | ? | ? | ? | ? | ? | ? | ? | ? | ? | ? |

Table S7. QTLs harbored within or partially overlapped with identified CNVs across the pig genome.

| **Chromosome Name** | **QTL Start(bp)** | **QTL End(bp)** | **QTL Description** |
| --- | --- | --- | --- |
| Chr.1 | 242497921 | 292955427 | Percentage type I fibers QTL (2794) |
| Chr.1 | 242497921 | 292955427 | Diameter of type IIb muscle fibers QTL (2795) |
| Chr.3 | 93197958 | 122295139 | pH for Semispinalis Capitis QTL (3844) |
| Chr.3 | 93197958 | 122295139 | pH 45 minutes post mortem QTL (21359) |
| Chr.3 | 93197958 | 122295139 | pH for Semispinalis Dorsi QTL (780) |
| Chr.4 | 96030439 | 96962176 | Linoleic acid content QTL (6392) |
| Chr.4 | 96030439 | 96962176 | Monounsaturated fatty acid content QTL (6393) |
| Chr.4 | 96030439 | 96962176 | Polyunsaturated fatty acid content QTL (6394) |
| Chr.4 | 96102583 | 97842792 | Palmitic acid content QTL (32104) |
| Chr.4 | 96381947 | 96479166 | Palmitoleic acid content QTL (32106) |
| Chr.4 | 96936045 | 98283003 | Ham weight QTL (932) |
| Chr.4 | 96936045 | 98283003 | Head weight QTL (936) |
| Chr.4 | 96936045 | 98283003 | Shoulder meat weight QTL (937) |
| Chr.4 | 96936045 | 98283003 | Loin muscle area QTL (945) |
| Chr.4 | 96936045 | 98283003 | Fat-cuts percentage QTL (950) |
| Chr.4 | 96936045 | 98283003 | Head weight QTL (1255) |
| Chr.4 | 96936045 | 98283003 | Shoulder external fat weight QTL (941) |
| Chr.4 | 96936045 | 98283003 | Ham weight QTL (811) |
| Chr.4 | 96936045 | 102054630 | Meat color-L QTL (233) |
| Chr.7 | 121170792 | 121907514 | Gestation length QTL (18174) |
| Chr.7 | 121907514 | 122108807 | Corpus luteum number QTL (31866) |
| Chr.7 | 122784847 | 122909260 | Average backfat thickness QTL (2914) |
| Chr.10 | 8541741 | 8594749 | pH 24 hr post mortem (ham) QTL (18702) |
| Chr.10 | 9061351 | 9211151 | pH 24 hr post mortem (ham) QTL (18681) |
| Chr.10 | 9308741 | 28168636 | Melanoma susceptibility QTL (7589) |
| Chr.10 | 9981187 | 16987451 | CIE-a* QTL (3067) |
| Chr.10 | 41334738 | 52252366 | Connective tissue amount QTL (21440) |
| Chr.10 | 41334738 | 52252366 | chew score QTL (3312) |
| Chr.10 | 41334738 | 52252366 | Body weight (slaughter) QTL (3278) |
| Chr.10 | 41334738 | 52252366 | Carcass weight (hot) QTL (3279) |
| Chr.10 | 41334738 | 52252366 | Marbling QTL (3280) |
| Chr.10 | 41334738 | 52252366 | Backfat linear at last rib QTL (3656) |
| Chr.10 | 41334738 | 61209673 | Hemoglobin QTL (5393) |
| Chr.10 | 41334738 | 61209673 | hematocrit QTL (5419) |
| Chr.10 | 41334738 | 61209673 | subjective abnormal odor QTL (4211) |
| Chr.10 | 41334738 | 61209673 | Teat number QTL (594) |
| Chr.10 | 41704037 | 42147656 | Myristic acid content QTL (7190) |
| Chr.10 | 43443512 | 43631569 | Cervical vertebra length QTL (9026) |
| Chr.10 | 43498290 | 57049354 | Lactate dehydrogenase level QTL (15111) |
| Chr.10 | 43498290 | 60475548 | Body weight (46 days) QTL (21263) |
| Chr.10 | 43812343 | 53626325 | Average daily gain (weaning to 25 kg) QTL (327) |
| Chr.10 | 43812343 | 53626325 | Hind leg conformation QTL (11847) |
| Chr.10 | 44144334 | 44342945 | Total number born alive QTL (18072) |
| Chr.10 | 44160167 | 52958690 | hematocrit QTL (7225) |
| Chr.10 | 45455212 | 45677304 | Hemoglobin QTL (7226) |
| Chr.10 | 45509990 | 45700943 | Average instron (star probe) force QTL (106) |
| Chr.10 | 46084707 | 46983347 | Feed conversion ratio QTL (29560) |
| Chr.10 | 46980658 | 50814251 | Umbilical hernia QTL (14317) |
| Chr.10 | 46980658 | 52252366 | Average instron (star probe) force QTL (2953) |
| Chr.10 | 46980658 | 61209673 | Linoleic acid content QTL (15861) |
| Chr.10 | 46980658 | 64951351 | Teat number QTL (2928) |
| Chr.10 | 49915725 | 49959130 | Mean corpuscular hemoglobin content QTL (7227) |
| Chr.10 | 49955416 | 49991301 | Mean corpuscular volume QTL (7228) |
| Chr.12 | 53974072 | 54026399 | Chronic pleuritis QTL (12277) |
| Chr.12 | 54326146 | 54441656 | Intramuscular fat content QTL (9037) |
| Chr.12 | 54489787 | 54668302 | Lymphocyte number QTL (5470) |
| Chr.12 | 54489787 | 54668302 | Segmented neutrophil number QTL (5482) |
| Chr.12 | 54489787 | 54668302 | Alkaline phosphatase activity QTL (6350) |
| Chr.12 | 54489787 | 54668302 | Sarcocystis miescheriana IgM levels QTL (7511) |
| Chr.12 | 54489787 | 54668302 | Melanoma susceptibility QTL (7575) |
| Chr.12 | 54489787 | 54668302 | Melanoma susceptibility QTL (7601) |
| Chr.12 | 54625522 | 54765397 | Total number born alive QTL (9806) |
| Chr.12 | 54625522 | 54765397 | Total number born QTL (9807) |
| Chr.12 | 54669135 | 54765397 | Immunoglobulin G level QTL (17869) |
| Chr.12 | 54669135 | 54765397 | White blood cell counts QTL (17870) |
| Chr.12 | 55482035 | 57621008 | post-stress mitogen induced IL-2 activity QTL (157) |
| Chr.12 | 56004411 | 59682626 | Number of visits to feeder per day QTL (22397) |
| Chr.12 | 56004411 | 59682626 | Number of visits to feeder per day QTL (22398) |
| Chr.12 | 56004411 | 59682626 | Number of visits to feeder per day QTL (22399) |
| Chr.12 | 56004411 | 59682626 | Number of visits to feeder per day QTL (22395) |
| Chr.12 | 56004411 | 59682626 | Number of visits to feeder per day QTL (22393) |
| Chr.12 | 56004411 | 59682626 | Number of visits to feeder per day QTL (22392) |
| Chr.12 | 56004411 | 59682626 | Number of visits to feeder per day QTL (22394) |
| Chr.12 | 56004411 | 59682626 | Number of visits to feeder per day QTL (22378) |
| Chr.12 | 56004411 | 59682626 | Number of visits to feeder per day QTL (22373) |
| Chr.12 | 56004411 | 59682626 | Number of visits to feeder per day QTL (22377) |
| Chr.12 | 56004411 | 59682626 | Number of visits to feeder per day QTL (22376) |
| Chr.12 | 56004411 | 59682626 | Number of visits to feeder per day QTL (22372) |
| Chr.12 | 56004411 | 59682626 | Number of visits to feeder per day QTL (22375) |
| Chr.12 | 56004411 | 59682626 | Number of visits to feeder per day QTL (22374) |
| Chr.12 | 56004411 | 59682626 | Number of visits to feeder per day QTL (22396) |
| Chr.12 | 56004411 | 59682626 | Loin muscle area QTL (22346) |
| Chr.12 | 56004411 | 59682626 | Myristic acid content QTL (22511) |
| Chr.12 | 56004411 | 59682626 | Palmitoleic acid content QTL (22512) |
| Chr.12 | 56004411 | 59682626 | Time in feeder per visit QTL (22383) |
| Chr.12 | 56004411 | 59682626 | Feed intake per feeding QTL (22538) |
| Chr.12 | 56004411 | 59682626 | Time in feeder per visit QTL (22419) |
| Chr.12 | 56004411 | 59682626 | Feed intake per feeding QTL (22537) |
| Chr.12 | 56004411 | 59682626 | Time in feeder per visit QTL (22418) |
| Chr.12 | 56004411 | 59682626 | Feed intake per feeding QTL (22535) |
| Chr.12 | 56004411 | 59682626 | Time in feeder per visit QTL (22417) |
| Chr.12 | 56004411 | 59682626 | Feed intake per feeding QTL (22536) |
| Chr.12 | 56160631 | 56142147 | Corpus luteum number QTL (31883) |
| Chr.12 | 47927603 | 59386165 | CIE-a* QTL (21403) |
| Chr.12 | 47927603 | 59386165 | Carcass weight (cold) QTL (3788) |
| Chr.12 | 47927603 | 59386165 | Backfat at tenth rib QTL (3824) |
| Chr.12 | 47927603 | 62961334 | Mean corpuscular hemoglobin content QTL (5424) |
| Chr.12 | 47927603 | 62961334 | Base excess QTL (6341) |
| Chr.12 | 47927603 | 62961334 | Percentage type IIb fibers QTL (7036) |
| Chr.12 | 47927603 | 62961334 | Percentage type IIb fibers QTL (7021) |
| Chr.12 | 47927603 | 62961334 | Adipocyte diameter QTL (12844) |
| Chr.18 | 46589140 | 116029014 | Corpus luteum number QTL (31909) |
| Chr.18 | 46687783 | 47658029 | Palmitic acid content QTL (17745) |

**Table S8. Annotation of genes in CNVs.**

| **Ensembl Gene ID** | **Chromosome Name** | **Gene Start (bp)** | **Gene End (bp)** | **Associated Gene Name** | **Description** |
| --- | --- | --- | --- | --- | --- |
| ENSSSCG00000005207 | 1 | 242045513 | 242108992 | ERMP1 | endoplasmic reticulum metallopeptidase 1 [Source:HGNC Symbol;Acc:HGNC:23703] |
| ENSSSCG00000005208 | 1 | 242115104 | 242228450 | KIAA1432 | KIAA1432 [Source:HGNC Symbol;Acc:HGNC:17686] |
| ENSSSCG00000005209 | 1 | 242260282 | 242261266 |  | 40S ribosomal protein S6 [Source:UniProtKB/TrEMBL;Acc:F1SML8] |
| ENSSSCG00000018137 | 1 | 242276465 | 242276569 | U6 | U6 spliceosomal RNA [Source:RFAM;Acc:RF00026] |
| ENSSSCG00000019937 | 1 | 242283095 | 242283224 | SNORA19 | Small nucleolar RNA SNORA19 [Source:RFAM;Acc:RF00413] |
| ENSSSCG00000005210 | 1 | 242298445 | 242324556 |  | Uncharacterized protein [Source:UniProtKB/TrEMBL;Acc:F1SK52] |
| ENSSSCG00000005211 | 1 | 242396225 | 242416185 | PDL1 | Sus scrofa CD274 molecule (CD274), mRNA. [Source:RefSeq mRNA;Acc:NM_001025221] |
| ENSSSCG00000005211 | 1 | 242396225 | 242416185 | PDL1 | Sus scrofa CD274 molecule (CD274), mRNA. [Source:RefSeq mRNA;Acc:NM_001025221] |
| ENSSSCG00000005212 | 1 | 242425744 | 242473399 | PLGRKT | plasminogen receptor, C-terminal lysine transmembrane protein [Source:HGNC Symbol;Acc:HGNC:23633] |
| ENSSSCG00000005216 | 1 | 242497239 | 242503501 |  | Sus scrofa relaxin (RLN), mRNA. [Source:RefSeq mRNA;Acc:NM_213872] |
| ENSSSCG00000005214 | 1 | 242534767 | 242561791 | INSL6 | insulin-like 6 [Source:HGNC Symbol;Acc:HGNC:6089] |
| ENSSSCG00000005215 | 1 | 242696463 | 242786177 | JAK2 | Sus scrofa Janus kinase 2 (JAK2), mRNA. [Source:RefSeq mRNA;Acc:NM_214113] |
| ENSSSCG00000005215 | 1 | 242696463 | 242786177 | JAK2 | Sus scrofa Janus kinase 2 (JAK2), mRNA. [Source:RefSeq mRNA;Acc:NM_214113] |
| ENSSSCG00000005215 | 1 | 242696463 | 242786177 | JAK2 | Sus scrofa Janus kinase 2 (JAK2), mRNA. [Source:RefSeq mRNA;Acc:NM_214113] |
| ENSSSCG00000005215 | 1 | 242696463 | 242786177 | JAK2 | Sus scrofa Janus kinase 2 (JAK2), mRNA. [Source:RefSeq mRNA;Acc:NM_214113] |
| ENSSSCG00000027893 | 1 | 242912773 | 242912879 | U6 | U6 spliceosomal RNA [Source:RFAM;Acc:RF00026] |
| ENSSSCG00000005217 | 1 | 242978574 | 243040568 | RCL1 | RNA terminal phosphate cyclase-like 1 [Source:HGNC Symbol;Acc:HGNC:17687] |
| ENSSSCG00000006340 | 4 | 95738941 | 95772729 | UAP1 | UDP-N-acteylglucosamine pyrophosphorylase 1 [Source:HGNC Symbol;Acc:HGNC:12457] |
| ENSSSCG00000006344 | 4 | 95859853 | 95906438 |  |  |
| ENSSSCG00000006343 | 4 | 95900184 | 95902590 | C1orf111 | chromosome 1 open reading frame 111 [Source:HGNC Symbol;Acc:HGNC:27648] |
| ENSSSCG00000006341 | 4 | 95921817 | 95926281 | SH2D1B | SH2 domain containing 1B [Source:HGNC Symbol;Acc:HGNC:30416] |
| ENSSSCG00000006342 | 4 | 95987540 | 95990458 |  |  |
| ENSSSCG00000022220 | 4 | 96044054 | 96087191 |  | Uncharacterized protein [Source:UniProtKB/TrEMBL;Acc:I3LML6] |
| ENSSSCG00000006345 | 4 | 96463144 | 96500755 | OLFML2B | olfactomedin-like 2B [Source:HGNC Symbol;Acc:HGNC:24558] |
| ENSSSCG00000018264 | 4 | 96477903 | 96477995 |  |  |
| ENSSSCG00000006346 | 4 | 96537111 | 96820806 | ATF6 | Uncharacterized protein [Source:UniProtKB/TrEMBL;Acc:F1S1C8] |
| ENSSSCG00000006347 | 4 | 96826973 | 96834005 |  | Uncharacterized protein [Source:UniProtKB/TrEMBL;Acc:F1S1C7] |
| ENSSSCG00000020133 | 4 | 96831605 | 96831670 | SNORD61 | Small nucleolar RNA SNORD61 [Source:RFAM;Acc:RF00270] |
| ENSSSCG00000006348 | 4 | 96850069 | 96855596 | FCRLB | Fc receptor-like B [Source:HGNC Symbol;Acc:HGNC:26431] |
| ENSSSCG00000006348 | 4 | 96850069 | 96855596 | FCRLB | Fc receptor-like B [Source:HGNC Symbol;Acc:HGNC:26431] |
| ENSSSCG00000020357 | 4 | 133617909 | 133618195 | 7SK | 7SK RNA [Source:RFAM;Acc:RF00100] |
| ENSSSCG00000006883 | 4 | 133994711 | 133998553 | RWDD3 | RWD domain containing 3 [Source:HGNC Symbol;Acc:HGNC:21393] |
| ENSSSCG00000006885 | 4 | 134085867 | 134202787 | ALG14 | ALG14, UDP-N-acetylglucosaminyltransferase subunit [Source:HGNC Symbol;Acc:HGNC:28287] |
| ENSSSCG00000021743 | 4 | 134226107 | 134257013 |  | Uncharacterized protein [Source:UniProtKB/TrEMBL;Acc:I3LLN7] |
| ENSSSCG00000006887 | 4 | 134245518 | 134269264 | CNN3 | Uncharacterized protein [Source:UniProtKB/TrEMBL;Acc:F1S543] |
| ENSSSCG00000006888 | 4 | 134354688 | 134382700 |  | Uncharacterized protein [Source:UniProtKB/TrEMBL;Acc:F1S542] |
| ENSSSCG00000026513 | 4 | 134387303 | 134406575 |  | Uncharacterized protein [Source:UniProtKB/TrEMBL;Acc:I3LD84] |
| ENSSSCG00000022447 | 4 | 134474325 | 134479725 | F3 | coagulation factor III (thromboplastin, tissue factor) [Source:HGNC Symbol;Acc:HGNC:3541] |
| ENSSSCG00000028620 | 4 | 134489857 | 134572224 | ABCD3 | Sus scrofa ATP-binding cassette, sub-family D (ALD), member 3 (ABCD3), mRNA. [Source:RefSeq mRNA;Acc:NM_001244133] |
| ENSSSCG00000028954 | 5 | 21236205 | 21237140 | OR6C3 | olfactory receptor, family 6, subfamily C, member 3 [Source:HGNC Symbol;Acc:HGNC:15437] |
| ENSSSCG00000026447 | 5 | 21256500 | 21258129 |  | Uncharacterized protein [Source:UniProtKB/TrEMBL;Acc:I3L565] |
| ENSSSCG00000024451 | 5 | 21266917 | 21267855 |  | Uncharacterized protein [Source:UniProtKB/TrEMBL;Acc:I3L943] |
| ENSSSCG00000021160 | 5 | 21279720 | 21280679 |  | Uncharacterized protein [Source:UniProtKB/TrEMBL;Acc:I3LGF8] |
| ENSSSCG00000028989 | 5 | 21296500 | 21297438 |  | Uncharacterized protein [Source:UniProtKB/TrEMBL;Acc:F1SQF4] |
| ENSSSCG00000000318 | 5 | 21324271 | 21325200 | OR6C4 | olfactory receptor, family 6, subfamily C, member 4 [Source:HGNC Symbol;Acc:HGNC:19632] |
| ENSSSCG00000000602 | 5 | 60736663 | 60851687 | RERG | RAS-like, estrogen-regulated, growth inhibitor [Source:HGNC Symbol;Acc:HGNC:15980] |
| ENSSSCG00000000603 | 5 | 60945101 | 60950201 | PDE6H | phosphodiesterase 6H, cGMP-specific, cone, gamma [Source:HGNC Symbol;Acc:HGNC:8790] |
| ENSSSCG00000000604 | 5 | 60965351 | 60987579 | ARHGDIB | Rho GDP dissociation inhibitor (GDI) beta [Source:HGNC Symbol;Acc:HGNC:679] |
| ENSSSCG00000000605 | 5 | 60989047 | 61014894 | ERP27 | endoplasmic reticulum protein 27 [Source:HGNC Symbol;Acc:HGNC:26495] |
| ENSSSCG00000000606 | 5 | 61054166 | 61058328 | MGP | Sus scrofa matrix Gla protein (MGP), mRNA. [Source:RefSeq mRNA;Acc:NM_214116] |
| ENSSSCG00000000607 | 5 | 61098145 | 61124875 | ART4 | Sus scrofa ADP-ribosyltransferase 4 (Dombrock blood group) (ART4), mRNA. [Source:RefSeq mRNA;Acc:NM_001243346] |
| ENSSSCG00000000607 | 5 | 61098145 | 61124875 | ART4 | Sus scrofa ADP-ribosyltransferase 4 (Dombrock blood group) (ART4), mRNA. [Source:RefSeq mRNA;Acc:NM_001243346] |
| ENSSSCG00000000607 | 5 | 61098145 | 61124875 | ART4 | Sus scrofa ADP-ribosyltransferase 4 (Dombrock blood group) (ART4), mRNA. [Source:RefSeq mRNA;Acc:NM_001243346] |
| ENSSSCG00000000607 | 5 | 61098145 | 61124875 | ART4 | Sus scrofa ADP-ribosyltransferase 4 (Dombrock blood group) (ART4), mRNA. [Source:RefSeq mRNA;Acc:NM_001243346] |
| ENSSSCG00000000608 | 5 | 61113389 | 61115083 |  | Uncharacterized protein [Source:UniProtKB/TrEMBL;Acc:F1SQW4] |
| ENSSSCG00000000609 | 5 | 61123957 | 61124688 | C12orf60 | chromosome 12 open reading frame 60 [Source:HGNC Symbol;Acc:HGNC:28726] |
| ENSSSCG00000000610 | 5 | 61135594 | 61136271 | SMCO3 | single-pass membrane protein with coiled-coil domains 3 [Source:HGNC Symbol;Acc:HGNC:34401] |
| ENSSSCG00000000611 | 5 | 61139249 | 61205929 | WBP11 | WW domain binding protein 11 [Source:HGNC Symbol;Acc:HGNC:16461] |
| ENSSSCG00000026724 | 5 | 61208222 | 61281901 | GUCY2C | guanylate cyclase 2C (heat stable enterotoxin receptor) [Source:HGNC Symbol;Acc:HGNC:4688] |
| ENSSSCG00000000613 | 5 | 61255167 | 61329426 |  | Sus scrofa guanylate cyclase 2C (heat stable enterotoxin receptor) (GUCY2C), mRNA. [Source:RefSeq mRNA;Acc:NM_214105] |
| ENSSSCG00000027602 | 5 | 61337116 | 61373313 | PLBD1 | phospholipase B domain containing 1 [Source:HGNC Symbol;Acc:HGNC:26215] |
| ENSSSCG00000000612 | 5 | 61444247 | 61571485 | ATF7IP | activating transcription factor 7 interacting protein [Source:HGNC Symbol;Acc:HGNC:20092] |
| ENSSSCG00000000612 | 5 | 61444247 | 61571485 | ATF7IP | activating transcription factor 7 interacting protein [Source:HGNC Symbol;Acc:HGNC:20092] |
| ENSSSCG00000000804 | 5 | 79126662 | 79217840 | ANO6 | anoctamin 6 [Source:HGNC Symbol;Acc:HGNC:25240] |
| ENSSSCG00000019963 | 5 | 79210271 | 79210379 | 5S_rRNA | 5S ribosomal RNA [Source:RFAM;Acc:RF00001] |
| ENSSSCG00000030685 | 5 | 79566481 | 79726285 | ARID2 | AT rich interactive domain 2 (ARID, RFX-like) [Source:HGNC Symbol;Acc:HGNC:18037] |
| ENSSSCG00000029372 | 5 | 79727239 | 79727353 |  |  |
| ENSSSCG00000000806 | 5 | 79736661 | 79785634 | SCAF11 | SR-related CTD-associated factor 11 [Source:HGNC Symbol;Acc:HGNC:10784] |
| ENSSSCG00000000806 | 5 | 79736661 | 79785634 | SCAF11 | SR-related CTD-associated factor 11 [Source:HGNC Symbol;Acc:HGNC:10784] |
| ENSSSCG00000000806 | 5 | 79736661 | 79785634 | SCAF11 | SR-related CTD-associated factor 11 [Source:HGNC Symbol;Acc:HGNC:10784] |
| ENSSSCG00000000807 | 5 | 79937452 | 80001030 | SLC38A1 | solute carrier family 38, member 1 [Source:HGNC Symbol;Acc:HGNC:13447] |
| ENSSSCG00000002464 | 7 | 121675708 | 121796718 | PRIMA1 | proline rich membrane anchor 1 [Source:HGNC Symbol;Acc:HGNC:18319] |
| ENSSSCG00000002467 | 7 | 122012985 | 122043651 | ASB2 | ankyrin repeat and SOCS box containing 2 [Source:HGNC Symbol;Acc:HGNC:16012] |
| ENSSSCG00000002469 | 7 | 122163461 | 122182984 | OTUB2 | OTU deubiquitinase, ubiquitin aldehyde binding 2 [Source:HGNC Symbol;Acc:HGNC:20351] |
| ENSSSCG00000002470 | 7 | 122186509 | 122207081 |  | Uncharacterized protein [Source:UniProtKB/TrEMBL;Acc:F1SCG7] |
| ENSSSCG00000002471 | 7 | 122215478 | 122220545 | ISG12(A) | Sus scrofa putative ISG12(a) protein (ISG12(A)), mRNA. [Source:RefSeq mRNA;Acc:NM_001198921] |
| ENSSSCG00000002472 | 7 | 122228313 | 122229025 | IFI27L2 | interferon, alpha-inducible protein 27-like 2 [Source:HGNC Symbol;Acc:HGNC:19753] |
| ENSSSCG00000023966 | 7 | 122374850 | 122383712 |  | Uncharacterized protein [Source:UniProtKB/TrEMBL;Acc:I3LKU6] |
| ENSSSCG00000002474 | 7 | 122462185 | 122531214 | PPP4R4 | protein phosphatase 4, regulatory subunit 4 [Source:HGNC Symbol;Acc:HGNC:23788] |
| ENSSSCG00000002475 | 7 | 122544966 | 122563955 | SERPINA6 | Sus scrofa serpin peptidase inhibitor, clade A (alpha-1 antiproteinase, antitrypsin), member 6 (SERPINA6), mRNA. [Source:RefSeq mRNA;Acc:NM_213812] |
| ENSSSCG00000027207 | 10 | 9647962 | 9812845 | GPATCH2 | G patch domain containing 2 [Source:HGNC Symbol;Acc:HGNC:25499] |
| ENSSSCG00000029417 | 10 | 9818047 | 9820864 |  | Uncharacterized protein [Source:UniProtKB/TrEMBL;Acc:I3LUW0] |
| ENSSSCG00000024004 | 10 | 9824897 | 9826103 |  |  |
| ENSSSCG00000029778 | 10 | 9884693 | 9979952 | SPATA17 | spermatogenesis associated 17 [Source:HGNC Symbol;Acc:HGNC:25184] |
| ENSSSCG00000011037 | 10 | 48738286 | 48829185 | TMEM236 | transmembrane protein 236 [Source:HGNC Symbol;Acc:HGNC:23473] |
| ENSSSCG00000011038 | 10 | 48856012 | 48960937 | MRC1 | mannose receptor, C type 1 [Source:HGNC Symbol;Acc:HGNC:7228] |
| ENSSSCG00000011038 | 10 | 48856012 | 48960937 | MRC1 | mannose receptor, C type 1 [Source:HGNC Symbol;Acc:HGNC:7228] |
| ENSSSCG00000011039 | 10 | 48975527 | 49054918 | SLC39A12 | solute carrier family 39 (zinc transporter), member 12 [Source:HGNC Symbol;Acc:HGNC:20860] |
| ENSSSCG00000020151 | 10 | 49297348 | 49297479 | 5S_rRNA | 5S ribosomal RNA [Source:RFAM;Acc:RF00001] |
| ENSSSCG00000011040 | 10 | 49568428 | 49626681 | CACNB2 | calcium channel, voltage-dependent, beta 2 subunit [Source:HGNC Symbol;Acc:HGNC:1402] |
| ENSSSCG00000019268 | 12 | 11333773 | 11333879 | U6 | U6 spliceosomal RNA [Source:RFAM;Acc:RF00026] |
| ENSSSCG00000027336 | 12 | 11409074 | 11414681 |  | Uncharacterized protein [Source:UniProtKB/TrEMBL;Acc:I3LSV2] |
| ENSSSCG00000025558 | 12 | 11415039 | 11426075 |  | Uncharacterized protein [Source:UniProtKB/TrEMBL;Acc:I3LPA6] |
| ENSSSCG00000017255 | 12 | 11441875 | 11488394 | ABCA5 | ATP-binding cassette, sub-family A (ABC1), member 5 [Source:HGNC Symbol;Acc:HGNC:35] |
| ENSSSCG00000017255 | 12 | 11441875 | 11488394 | ABCA5 | ATP-binding cassette, sub-family A (ABC1), member 5 [Source:HGNC Symbol;Acc:HGNC:35] |
| ENSSSCG00000017255 | 12 | 11441875 | 11488394 | ABCA5 | ATP-binding cassette, sub-family A (ABC1), member 5 [Source:HGNC Symbol;Acc:HGNC:35] |
| ENSSSCG00000027531 | 12 | 11570713 | 11598919 |  | Uncharacterized protein [Source:UniProtKB/TrEMBL;Acc:I3LB38] |
| ENSSSCG00000030523 | 12 | 11584316 | 11584422 | U6 | U6 spliceosomal RNA [Source:RFAM;Acc:RF00026] |
| ENSSSCG00000029730 | 12 | 11635217 | 11644867 |  | Uncharacterized protein [Source:UniProtKB/TrEMBL;Acc:I3LPG9] |
| ENSSSCG00000026094 | 12 | 11647563 | 11653258 |  | Uncharacterized protein [Source:UniProtKB/TrEMBL;Acc:I3LI01] |
| ENSSSCG00000029009 | 12 | 11678097 | 11687131 |  | Uncharacterized protein [Source:UniProtKB/TrEMBL;Acc:I3LDD8] |
| ENSSSCG00000027891 | 12 | 11687325 | 11689036 |  | Uncharacterized protein [Source:UniProtKB/TrEMBL;Acc:I3LCQ5] |
| ENSSSCG00000030082 | 12 | 11696953 | 11716938 |  | Uncharacterized protein [Source:UniProtKB/TrEMBL;Acc:I3L612] |
| ENSSSCG00000017257 | 12 | 11732391 | 11787345 |  | Uncharacterized protein [Source:UniProtKB/TrEMBL;Acc:F1RV25] |
| ENSSSCG00000026413 | 12 | 11772801 | 11772907 | U6 | U6 spliceosomal RNA [Source:RFAM;Acc:RF00026] |
| ENSSSCG00000017992 | 12 | 56657167 | 56658939 | MFSD6L | major facilitator superfamily domain containing 6-like [Source:HGNC Symbol;Acc:HGNC:26656] |
| ENSSSCG00000017990 | 12 | 56662053 | 56723007 | PIK3R6 | phosphoinositide-3-kinase, regulatory subunit 6 [Source:HGNC Symbol;Acc:HGNC:27101] |
| ENSSSCG00000017991 | 12 | 56731612 | 56799399 | PIK3R5 | phosphoinositide-3-kinase, regulatory subunit 5 [Source:HGNC Symbol;Acc:HGNC:30035] |
| ENSSSCG00000017993 | 12 | 56822669 | 56823739 | NTN1 | netrin 1 [Source:HGNC Symbol;Acc:HGNC:8029] |
| ENSSSCG00000017994 | 12 | 57334576 | 57339673 | WDR16 | WD repeat domain 16 [Source:HGNC Symbol;Acc:HGNC:16053] |
| ENSSSCG00000017995 | 12 | 57342299 | 57399793 | USP43 | ubiquitin specific peptidase 43 [Source:HGNC Symbol;Acc:HGNC:20072] |
| ENSSSCG00000017996 | 12 | 57405795 | 57419222 | DHRS7C | dehydrogenase/reductase (SDR family) member 7C [Source:HGNC Symbol;Acc:HGNC:32423] |
| ENSSSCG00000017997 | 12 | 57423508 | 57435711 |  | Uncharacterized protein [Source:UniProtKB/TrEMBL;Acc:F1SS85] |
| ENSSSCG00000017998 | 12 | 57438490 | 57493322 | GLP2R | glucagon-like peptide 2 receptor [Source:HGNC Symbol;Acc:HGNC:4325] |
| ENSSSCG00000024787 | 17 | 41689946 | 41699956 | BPIFA3 | BPI fold containing family A, member 3 [Source:HGNC Symbol;Acc:HGNC:16204] |
| ENSSSCG00000007260 | 17 | 41704948 | 41711958 | BPIFA1 | BPI fold containing family A, member 1 [Source:HGNC Symbol;Acc:HGNC:15749] |
| ENSSSCG00000007260 | 17 | 41704948 | 41711958 | BPIFA1 | BPI fold containing family A, member 1 [Source:HGNC Symbol;Acc:HGNC:15749] |
| ENSSSCG00000007260 | 17 | 41704948 | 41711958 | BPIFA1 | BPI fold containing family A, member 1 [Source:HGNC Symbol;Acc:HGNC:15749] |
| ENSSSCG00000007260 | 17 | 41704948 | 41711958 | BPIFA1 | BPI fold containing family A, member 1 [Source:HGNC Symbol;Acc:HGNC:15749] |
| ENSSSCG00000007260 | 17 | 41704948 | 41711958 | BPIFA1 | BPI fold containing family A, member 1 [Source:HGNC Symbol;Acc:HGNC:15749] |
| ENSSSCG00000007260 | 17 | 41704948 | 41711958 | BPIFA1 | BPI fold containing family A, member 1 [Source:HGNC Symbol;Acc:HGNC:15749] |
| ENSSSCG00000007260 | 17 | 41704948 | 41711958 | BPIFA1 | BPI fold containing family A, member 1 [Source:HGNC Symbol;Acc:HGNC:15749] |
| ENSSSCG00000007260 | 17 | 41704948 | 41711958 | BPIFA1 | BPI fold containing family A, member 1 [Source:HGNC Symbol;Acc:HGNC:15749] |
| ENSSSCG00000007261 | 17 | 41732635 | 41760088 | BPIFB1 | Sus scrofa BPI fold containing family B, member 1 (BPIFB1), mRNA. [Source:RefSeq mRNA;Acc:NM_001101032] |
| ENSSSCG00000007262 | 17 | 41763475 | 41786295 |  | Sus scrofa long palate lung and nasal epithelium protein 5 (BPIFB5), mRNA. [Source:RefSeq mRNA;Acc:NM_001101033] |
| ENSSSCG00000007263 | 17 | 41802797 | 41819073 |  | Uncharacterized protein [Source:UniProtKB/TrEMBL;Acc:F1S501] |
| ENSSSCG00000007263 | 17 | 41802797 | 41819073 |  | Uncharacterized protein [Source:UniProtKB/TrEMBL;Acc:F1S501] |
| ENSSSCG00000007264 | 17 | 41827904 | 41861498 | CDK5RAP1 | CDK5 regulatory subunit associated protein 1 [Source:HGNC Symbol;Acc:HGNC:15880] |
| ENSSSCG00000007265 | 17 | 41868153 | 41896580 | SNTA1 | syntrophin, alpha 1 [Source:HGNC Symbol;Acc:HGNC:11167] |
| ENSSSCG00000027410 | 17 | 41984968 | 42071429 | CBFA2T2 | core-binding factor, runt domain, alpha subunit 2; translocated to, 2 [Source:HGNC Symbol;Acc:HGNC:1536] |
| ENSSSCG00000027410 | 17 | 41984968 | 42071429 | CBFA2T2 | core-binding factor, runt domain, alpha subunit 2; translocated to, 2 [Source:HGNC Symbol;Acc:HGNC:1536] |
| ENSSSCG00000029626 | 17 | 42101648 | 42116081 | NECAB3 | N-terminal EF-hand calcium binding protein 3 [Source:HGNC Symbol;Acc:HGNC:15851] |
| ENSSSCG00000007267 | 17 | 42108762 | 42109499 | ACTL10 | actin-like 10 [Source:HGNC Symbol;Acc:HGNC:16127] |
| ENSSSCG00000007268 | 17 | 42116178 | 42121899 | E2F1 | E2F transcription factor 1 [Source:HGNC Symbol;Acc:HGNC:3113] |
| ENSSSCG00000007269 | 17 | 42143808 | 42150802 | PXMP4 | peroxisomal membrane protein 4, 24kDa [Source:HGNC Symbol;Acc:HGNC:15920] |
| ENSSSCG00000024787 | 17 | 42102480.19 | 42136469.2 | BPIFA3 | BPI fold containing family A, member 3 [Source:HGNC Symbol;Acc:HGNC:16205] |
| ENSSSCG00000007260 | 17 | 42126655.35 | 42161951.8 | BPIFA1 | BPI fold containing family A, member 1 [Source:HGNC Symbol;Acc:HGNC:15749] |
| ENSSSCG00000007260 | 17 | 42150830.5 | 42187434.4 | BPIFA1 | BPI fold containing family A, member 1 [Source:HGNC Symbol;Acc:HGNC:15749] |
| ENSSSCG00000022865 | 18 | 22609817 | 23086523 | GRM8 | glutamate receptor, metabotropic 8 [Source:HGNC Symbol;Acc:HGNC:4600] |
| ENSSSCG00000026390 | 18 | 23375402 | 23375508 | U6 | U6 spliceosomal RNA [Source:RFAM;Acc:RF00026] |
| ENSSSCG00000026580 | 18 | 23473296 | 23473402 | U6 | U6 spliceosomal RNA [Source:RFAM;Acc:RF00026] |
| ENSSSCG00000025642 | 18 | 23522451 | 23522710 | Metazoa_SRP | Metazoan signal recognition particle RNA [Source:RFAM;Acc:RF00017] |
| ENSSSCG00000016673 | 18 | 46427127 | 46436822 | GHRHR | growth hormone releasing hormone receptor [Source:HGNC Symbol;Acc:HGNC:4266] |
| ENSSSCG00000016674 | 18 | 46529495 | 46660391 | FAM188B | family with sequence similarity 188, member B [Source:HGNC Symbol;Acc:HGNC:21916] |
| ENSSSCG00000016674 | 18 | 46529495 | 46660391 | FAM188B | family with sequence similarity 188, member B [Source:HGNC Symbol;Acc:HGNC:21916] |
| ENSSSCG00000016674 | 18 | 46529495 | 46660391 | FAM188B | family with sequence similarity 188, member B [Source:HGNC Symbol;Acc:HGNC:21916] |
| ENSSSCG00000016676 | 18 | 46637157 | 46641921 |  |  |
| ENSSSCG00000016675 | 18 | 46648683 | 46651308 | CRHR2 | corticotropin releasing hormone receptor 2 [Source:HGNC Symbol;Acc:HGNC:2358] |
| ENSSSCG00000016677 | 18 | 46675442 | 46768425 | GARS | glycyl-tRNA synthetase [Source:HGNC Symbol;Acc:HGNC:4162] |
| ENSSSCG00000016679 | 18 | 46810848 | 46814429 |  | Uncharacterized protein [Source:UniProtKB/TrEMBL;Acc:F1SII1] |
| ENSSSCG00000016680 | 18 | 46871887 | 46909048 | ZNRF2 | zinc and ring finger 2 [Source:HGNC Symbol;Acc:HGNC:22316] |
| ENSSSCG00000016678 | 18 | 46956599 | 47012335 | NOD1 | nucleotide-binding oligomerization domain-containing protein 1 [Source:RefSeq peptide;Acc:NP_001107749] |
| ENSSSCG00000016678 | 18 | 46956599 | 47012335 | NOD1 | nucleotide-binding oligomerization domain-containing protein 1 [Source:RefSeq peptide;Acc:NP_001107749] |
| ENSSSCG00000016678 | 18 | 46956599 | 47012335 | NOD1 | nucleotide-binding oligomerization domain-containing protein 1 [Source:RefSeq peptide;Acc:NP_001107749] |
| ENSSSCG00000016682 | 18 | 47238397 | 47273722 | PLEKHA8 | pleckstrin homology domain containing, family A (phosphoinositide binding specific) member 8 [Source:HGNC Symbol;Acc:HGNC:30037] |
| ENSSSCG00000027449 | 18 | 47295861 | 47306104 | FKBP14 | FK506 binding protein 14, 22 kDa [Source:HGNC Symbol;Acc:HGNC:18625] |
| ENSSSCG00000016684 | 18 | 47342426 | 47378290 | SCRN1 | secernin 1 [Source:HGNC Symbol;Acc:HGNC:22192] |

Table S9. Gene Ontology and pathways of genes in CNVs regions.

| **Category** | **Term** | **Count** | **PValue** | **Genes** | **List Total** | **Pop Hits** | **Pop Total** | **Fold Enrichment** | **Bonferroni** | **FDR** |
| --- | --- | --- | --- | --- | --- | --- | --- | --- | --- | --- |
| GOTERM_BP_FAT | GO:0032940~secretion by cell | 4 | 0.007083 | SCRN1, GARS, NECAB3, GHRHR | 27 | 207 | 13528 | 9.681875 | 0.97447 | 9.770682 |
| GOTERM_BP_FAT | GO:0007188~G-protein signaling, coupled to cAMP nucleotide second messenger | 3 | 0.011499 | CRHR2, GLP2R, GHRHR | 27 | 85 | 13528 | 17.68366 | 0.99744 | 15.40392 |
| GOTERM_BP_FAT | GO:0019933~cAMP-mediated signaling | 3 | 0.014214 | CRHR2, GLP2R, GHRHR | 27 | 95 | 13528 | 15.82222 | 0.999381 | 18.70312 |
| GOTERM_BP_FAT | GO:0046903~secretion | 4 | 0.019253 | SCRN1, GARS, NECAB3, GHRHR | 27 | 300 | 13528 | 6.680494 | 0.999956 | 24.51196 |
| GOTERM_BP_FAT | GO:0007187~G-protein signaling, coupled to cyclic nucleotide second messenger | 3 | 0.020383 | CRHR2, GLP2R, GHRHR | 27 | 115 | 13528 | 13.07053 | 0.999976 | 25.76053 |
| GOTERM_BP_FAT | GO:0019935~cyclic-nucleotide-mediated signaling | 3 | 0.02562 | CRHR2, GLP2R, GHRHR | 27 | 130 | 13528 | 11.56239 | 0.999998 | 31.299 |
| GOTERM_BP_FAT | GO:0007528~neuromuscular junction development | 2 | 0.037771 | CACNB2, SNTA1 | 27 | 20 | 13528 | 50.1037 | 1 | 42.70245 |
| GOTERM_BP_FAT | GO:0002793~positive regulation of peptide secretion | 2 | 0.043317 | JAK2, GHRHR | 27 | 23 | 13528 | 43.56844 | 1 | 47.29818 |
| GOTERM_BP_FAT | GO:0008284~positive regulation of cell proliferation | 4 | 0.043945 | JAK2, GLP2R, NTN1, GHRHR | 27 | 414 | 13528 | 4.840938 | 1 | 47.79646 |
| GOTERM_BP_FAT | GO:0008219~cell death | 5 | 0.046633 | E2F1, NOD1, GARS, JAK2, NTN1 | 27 | 719 | 13528 | 3.484263 | 1 | 49.87973 |
| GOTERM_BP_FAT | GO:0016265~death | 5 | 0.047636 | E2F1, NOD1, GARS, JAK2, NTN1 | 27 | 724 | 13528 | 3.460201 | 1 | 50.63703 |
| GOTERM_BP_FAT | GO:0048741~skeletal muscle fiber development | 2 | 0.052492 | CACNB2, SNTA1 | 27 | 28 | 13528 | 35.78836 | 1 | 54.15519 |
| GOTERM_BP_FAT | GO:0048747~muscle fiber development | 2 | 0.065195 | CACNB2, SNTA1 | 27 | 35 | 13528 | 28.63069 | 1 | 62.28598 |
| GOTERM_BP_FAT | GO:0046887~positive regulation of hormone secretion | 2 | 0.066996 | JAK2, GHRHR | 27 | 36 | 13528 | 27.83539 | 1 | 63.3236 |
| GOTERM_BP_FAT | GO:0019932~second-messenger-mediated signaling | 3 | 0.074357 | CRHR2, GLP2R, GHRHR | 27 | 235 | 13528 | 6.396217 | 1 | 67.29364 |
| GOTERM_BP_FAT | GO:0051240~positive regulation of multicellular organismal process | 3 | 0.079345 | NOD1, JAK2, GHRHR | 27 | 244 | 13528 | 6.160291 | 1 | 69.75233 |
| GOTERM_BP_FAT | GO:0002791~regulation of peptide secretion | 2 | 0.084827 | JAK2, GHRHR | 27 | 46 | 13528 | 21.78422 | 1 | 72.25552 |
| GOTERM_BP_FAT | GO:0007242~intracellular signaling cascade | 6 | 0.087452 | CRHR2, NOD1, JAK2, GLP2R, ASB2, GHRHR | 27 | 1256 | 13528 | 2.393489 | 1 | 73.38479 |
| GOTERM_BP_FAT | GO:0055002~striated muscle cell development | 2 | 0.095367 | CACNB2, SNTA1 | 27 | 52 | 13528 | 19.27066 | 1 | 76.53572 |
| GOTERM_BP_FAT | GO:0019941~modification-dependent protein catabolic process | 4 | 0.096042 | ASB2, USP43, OTUB2, ZNRF2 | 27 | 574 | 13528 | 3.491547 | 1 | 76.78745 |
| GOTERM_BP_FAT | GO:0043632~modification-dependent macromolecule catabolic process | 4 | 0.096042 | ASB2, USP43, OTUB2, ZNRF2 | 27 | 574 | 13528 | 3.491547 | 1 | 76.78745 |
| GOTERM_MF_FAT | GO:0005509~calcium ion binding | 5 | 0.085545 | MRC1, FKBP14, CACNB2, NECAB3, SNTA1 | 25 | 919 | 12983 | 2.825462 | 0.99993 | 63.40314 |
| KEGG_PATHWAY | hsa04080:Neuroactive ligand-receptor interaction | 3 | 0.087006 | CRHR2, GLP2R, GHRHR | 11 | 256 | 5085 | 5.417259 | 0.986133 | 57.87741 |

**Table S10. Primers used in Ntn1 and CNV12 expression analysis.**

| **Type** | **NO.** | **Primers** | |
| --- | --- | --- | --- |
|  |  | **Sense** | **Antisense** |
| DNA | CNV12 | CTGTCTGTCTGTCTGTCTGTCT | TGCTGTGGCTGTGGTGTAG |
| RNA | CNV12 | GGTGTGGTGCGGACTTATCT | AGGACTTGCGTGTGGTGAC |
| RNA | *Ntn1* | GTCGCTCGGCAAGAAGTTC | CTCGTTCTGCTTGGTGATGG |

**Table S11. Association of SNPs with meat quality traits (1,000SNPs).**

| **Marker** | **Chr** | **Position** | **IMF** | **SFN** | **Marbling** | **B24** | **A24** | **L24** | **B6** | **A6** | **L6** | **pH24** | **pH6** | **Moisture** |
| --- | --- | --- | --- | --- | --- | --- | --- | --- | --- | --- | --- | --- | --- | --- |
| ALGA0066945 | 3 | 1.4E+08 | 3.6E-11 | 0.307137 | 1.08E-09 | 0.002567 | 2.07E-06 | 0.527036 | 0.721165 | 3.56E-07 | 0.049646 | 0.097551 | 3.76E-05 | 9.61E-08 |
| ASGA0094812 | 12 | 57394039 | 1.53E-09 | 0.631413 | 2.73E-11 | 0.014728 | 4.72E-05 | 0.353416 | 0.82894 | 6.88E-07 | 0.008619 | 0.091189 | 2.54E-06 | 1.57E-09 |
| ASGA0102838 | 12 | 55575876 | 3.15E-09 | 0.628693 | 2.83E-08 | 0.011907 | 2.72E-06 | 0.442032 | 0.690432 | 4.29E-07 | 0.045399 | 0.086167 | 9.77E-05 | 3.5E-06 |
| MARC0017000 | 12 | 58347308 | 1.79E-08 | 0.445401 | 3.81E-10 | 0.030011 | 0.001247 | 0.882526 | 0.879182 | 1.85E-05 | 0.017131 | 0.158113 | 1.29E-05 | 9.99E-09 |
| ALGA0109745 | 12 | 61142611 | 8.42E-07 | 0.125066 | 3.8E-08 | 0.021805 | 0.000347 | 0.644479 | 0.767508 | 1.61E-05 | 0.047968 | 0.442727 | 0.001514 | 1.94E-06 |
| ALGA0067119 | 12 | 58078076 | 9.53E-07 | 0.024819 | 0.000217 | 0.244917 | 7.98E-05 | 0.768013 | 0.135161 | 8.15E-05 | 0.187917 | 0.380038 | 0.128825 | 2.8E-05 |
| ASGA0054854 | 12 | 47528805 | 2.69E-06 | 0.282718 | 2.51E-06 | 0.015171 | 0.002484 | 0.47326 | 0.448533 | 0.000107 | 0.744051 | 0.672477 | 0.000768 | 0.000489 |
| ASGA0089507 | 12 | 57195654 | 2.89E-06 | 0.319134 | 5.77E-07 | 0.331967 | 0.001835 | 0.083911 | 0.063674 | 7.74E-06 | 0.001006 | 0.759921 | 0.000116 | 2.26E-05 |
| M1GA0016908 | 12 | 52692402 | 3.22E-06 | 0.04671 | 0.000329 | 0.15623 | 8.99E-05 | 0.339954 | 0.826048 | 2.12E-05 | 0.062846 | 0.465942 | 0.014324 | 0.000309 |
| MARC0022924 | 12 | 59594821 | 5.87E-06 | 0.410898 | 2.44E-05 | 0.028428 | 0.035938 | 0.869556 | 0.138834 | 0.046359 | 0.710531 | 0.144325 | 0.161266 | 0.000265 |
| ALGA0108818 | 12 | 49085493 | 6.75E-06 | 0.521917 | 1.17E-05 | 0.034565 | 0.00362 | 0.335757 | 0.780284 | 0.000162 | 0.744108 | 0.491277 | 0.000932 | 0.000252 |
| M1GA0017195 | 12 | 60768750 | 1.03E-05 | 0.03858 | 3.55E-06 | 0.236993 | 0.092772 | 0.812676 | 0.580293 | 0.016417 | 0.954221 | 0.964615 | 0.026655 | 1.48E-05 |
| M1GA0017151 | 12 | 60577245 | 1.38E-05 | 0.628895 | 7.75E-06 | 0.160411 | 0.000118 | 0.123336 | 0.721059 | 3.31E-05 | 0.007424 | 0.716126 | 0.006733 | 0.000463 |
| DIAS0003416 | 12 | 59250231 | 2.34E-05 | 0.39322 | 0.006709 | 0.131717 | 0.000242 | 0.313481 | 0.432351 | 0.000929 | 0.097932 | 0.731731 | 0.054121 | 0.02033 |
| ALGA0066985 | 12 | 56063944 | 2.55E-05 | 0.378693 | 1.04E-05 | 0.03374 | 0.051674 | 0.619563 | 0.678768 | 0.047053 | 0.723545 | 0.220215 | 0.014319 | 0.000103 |
| ASGA0055110 | 12 | 56142147 | 3.49E-05 | 0.441065 | 1.18E-05 | 0.044354 | 0.045583 | 0.699512 | 0.74948 | 0.04174 | 0.622585 | 0.135128 | 0.016285 | 7.7E-05 |
| ASGA0097613 | 0 | 0 | 4.15E-05 | 0.018471 | 0.000151 | 0.202405 | 0.000251 | 0.635935 | 0.638275 | 3.06E-05 | 0.052727 | 0.595638 | 0.033639 | 7.73E-05 |
| ALGA0066981 | 12 | 56035133 | 4.97E-05 | 0.472572 | 0.015837 | 0.074314 | 0.004301 | 0.80724 | 0.042339 | 0.012129 | 0.649381 | 0.414877 | 0.25456 | 0.017017 |
| ASGA0099873 | 12 | 61061041 | 5.68E-05 | 0.038582 | 1.61E-07 | 0.043441 | 0.009059 | 0.408143 | 0.78658 | 0.000282 | 0.187136 | 0.884096 | 0.000187 | 0.000212 |
| ALGA0067189 | 12 | 60604972 | 8.29E-05 | 0.00092 | 5.27E-05 | 0.601538 | 0.010739 | 0.235722 | 0.622336 | 0.000279 | 0.231137 | 0.998407 | 0.006424 | 4.02E-05 |
| ALGA0111196 | 12 | 51584975 | 0.000135 | 0.703006 | 9.34E-05 | 0.32338 | 0.006661 | 0.212975 | 0.02161 | 0.000317 | 0.008527 | 0.46933 | 0.000235 | 0.000778 |
| ASGA0084548 | 12 | 60923280 | 0.000168 | 0.06375 | 6.17E-07 | 0.110743 | 0.012761 | 0.433353 | 0.71711 | 0.000442 | 0.143271 | 0.902607 | 0.001051 | 7.74E-05 |
| DRGA0009478 | 9 | 80677221 | 0.000262 | 0.470335 | 0.01505 | 0.001468 | 0.021257 | 0.803088 | 0.16569 | 0.68683 | 0.618861 | 0.155208 | 0.832525 | 0.011028 |
| MARC0091838 | 9 | 75391264 | 0.000271 | 0.912881 | 0.007282 | 0.00151 | 0.033879 | 0.593506 | 0.16657 | 0.509192 | 0.564412 | 0.12643 | 0.8128 | 0.000987 |
| ALGA0104951 | 12 | 42865678 | 0.000279 | 0.789946 | 0.000608 | 0.293276 | 0.170594 | 0.318344 | 0.887685 | 0.017643 | 0.884137 | 0.708134 | 0.002466 | 0.003808 |
| MARC0097241 | 14 | 63058384 | 0.000315 | 0.666559 | 0.008254 | 0.250733 | 0.003025 | 0.52769 | 0.497281 | 0.211978 | 0.402889 | 0.258525 | 0.694102 | 0.005224 |
| ALGA0067173 | 12 | 60510869 | 0.000345 | 0.014784 | 0.001191 | 0.605463 | 0.004916 | 0.407035 | 0.923049 | 0.001135 | 0.228591 | 0.407103 | 0.170035 | 6.52E-06 |
| ASGA0063160 | 14 | 47589611 | 0.00035 | 0.902418 | 0.013997 | 0.590945 | 0.011753 | 0.302721 | 0.465445 | 0.036576 | 0.465993 | 0.158948 | 0.017177 | 0.219264 |
| ASGA0090653 | 12 | 42945563 | 0.000398 | 0.925006 | 0.002285 | 0.22808 | 0.152248 | 0.400277 | 0.944553 | 0.007947 | 0.503779 | 0.728662 | 0.00699 | 0.005672 |
| ALGA0077287 | 14 | 47079961 | 0.000398 | 0.856043 | 0.016656 | 0.727903 | 0.01195 | 0.252743 | 0.455265 | 0.033433 | 0.520279 | 0.131045 | 0.013822 | 0.174747 |
| H3GA0034668 | 12 | 52620382 | 0.000418 | 0.243086 | 0.007941 | 0.158738 | 0.031865 | 0.547341 | 0.545716 | 0.098243 | 0.901763 | 0.431032 | 0.079766 | 0.017835 |
| ALGA0066876 | 12 | 51984256 | 0.000509 | 0.069652 | 0.014096 | 0.281757 | 0.00033 | 0.498634 | 0.178039 | 0.000595 | 0.488189 | 0.547865 | 0.495807 | 0.005168 |
| DRGA0009473 | 9 | 79550062 | 0.000674 | 0.37645 | 0.010541 | 0.000726 | 0.036979 | 0.976123 | 0.310636 | 0.914051 | 0.907412 | 0.298373 | 0.332909 | 0.022573 |
| M1GA0008850 | 6 | 92900402 | 0.000674 | 0.409459 | 0.022884 | 0.207312 | 0.019615 | 0.558639 | 0.838124 | 0.106959 | 0.085923 | 0.340712 | 0.127562 | 0.084276 |
| ALGA0067220 | 12 | 60725589 | 0.000708 | 0.001179 | 0.0002 | 0.730957 | 0.035168 | 0.430034 | 0.696976 | 0.00186 | 0.433044 | 0.761639 | 0.01311 | 7.1E-05 |
| ALGA0109191 | 6 | 89504250 | 0.000716 | 0.415203 | 0.03101 | 0.172154 | 0.00704 | 0.442517 | 0.968228 | 0.062213 | 0.050065 | 0.200595 | 0.082627 | 0.168234 |
| H3GA0034455 | 12 | 43506714 | 0.000735 | 0.476441 | 0.003269 | 0.0628 | 0.148703 | 0.765099 | 0.753995 | 0.020639 | 0.999754 | 0.984382 | 0.001188 | 0.075351 |
| M1GA0001931 | 1 | 3.07E+08 | 0.000765 | 0.779002 | 0.052746 | 0.126883 | 0.143099 | 0.495883 | 0.368469 | 0.780323 | 0.778978 | 0.954669 | 0.158075 | 0.042653 |
| MARC0074986 | 6 | 89986075 | 0.000773 | 0.454687 | 0.032373 | 0.082416 | 0.007043 | 0.557962 | 0.95356 | 0.082723 | 0.077203 | 0.195549 | 0.090243 | 0.169132 |
| ALGA0109059 | 12 | 46857615 | 0.000784 | 0.354103 | 0.000511 | 0.31122 | 0.009868 | 0.050136 | 0.111163 | 8.1E-05 | 0.100312 | 0.992461 | 0.000775 | 0.004003 |
| ALGA0053729 | 9 | 76797613 | 0.000805 | 0.47019 | 0.038207 | 0.00251 | 0.030979 | 0.763981 | 0.129642 | 0.678168 | 0.666891 | 0.208508 | 0.976833 | 0.024831 |
| H3GA0034538 | 12 | 46257013 | 0.000805 | 0.358871 | 0.00026 | 0.398762 | 0.014221 | 0.062519 | 0.106559 | 0.000193 | 0.064179 | 0.915504 | 0.001157 | 0.00371 |
| ALGA0118422 | 14 | 16110030 | 0.000882 | 0.353653 | 0.026806 | 0.820927 | 0.219932 | 0.167486 | 0.891455 | 0.486627 | 0.064811 | 0.695002 | 0.241787 | 0.000295 |
| ASGA0060872 | 14 | 7471763 | 0.001016 | 0.704025 | 0.018047 | 0.065529 | 0.59389 | 0.952418 | 0.775354 | 0.306541 | 0.841523 | 0.116738 | 0.03247 | 0.222776 |
| MARC0056114 | 8 | 1.09E+08 | 0.001029 | 0.936708 | 0.029004 | 0.008377 | 0.445571 | 0.697575 | 0.056717 | 0.566499 | 0.155297 | 0.349416 | 0.132374 | 0.014681 |
| ASGA0055169 | 12 | 56768525 | 0.001034 | 0.547094 | 0.0031 | 0.738915 | 0.047287 | 0.129671 | 0.040481 | 0.018798 | 0.045863 | 0.553184 | 0.007815 | 0.00637 |
| ALGA0077312 | 14 | 46933287 | 0.001067 | 0.988711 | 0.052818 | 0.696995 | 0.007792 | 0.256695 | 0.299301 | 0.024905 | 0.576331 | 0.08044 | 0.04478 | 0.222263 |
| ASGA0084764 | 6 | 1.3E+08 | 0.001137 | 0.948424 | 0.008022 | 0.029433 | 0.365665 | 0.454311 | 0.308424 | 0.987986 | 0.784123 | 0.467963 | 0.473665 | 0.679144 |
| ALGA0078729 | 14 | 79038257 | 0.00116 | 0.383413 | 0.019275 | 0.235144 | 0.000938 | 0.49519 | 0.638126 | 0.224946 | 0.321424 | 0.288969 | 0.791479 | 0.01158 |
| ASGA0064345 | 14 | 78995471 | 0.00116 | 0.383413 | 0.019275 | 0.235144 | 0.000938 | 0.49519 | 0.638126 | 0.224946 | 0.321424 | 0.288969 | 0.791479 | 0.01158 |
| ASGA0063619 | 14 | 61227999 | 0.001239 | 0.263976 | 0.018854 | 0.937838 | 0.07577 | 0.342624 | 0.245666 | 0.226113 | 0.321558 | 0.464444 | 0.577558 | 0.003366 |
| H3GA0054731 | 9 | 71989525 | 0.00127 | 0.737276 | 0.004262 | 5.89E-05 | 0.176953 | 0.094703 | 0.046398 | 0.996635 | 0.455653 | 0.047057 | 0.708246 | 0.003801 |
| H3GA0039131 | 14 | 15754408 | 0.001296 | 0.328192 | 0.050659 | 0.830844 | 0.244275 | 0.162929 | 0.995387 | 0.553097 | 0.053371 | 0.602893 | 0.232112 | 0.000378 |
| ALGA0074803 | 14 | 8496813 | 0.001316 | 0.554692 | 0.056626 | 0.391451 | 0.544428 | 0.465022 | 0.287632 | 0.620719 | 0.381052 | 0.85034 | 0.664491 | 0.018383 |
| H3GA0040031 | 14 | 47037143 | 0.001357 | 0.706713 | 0.027303 | 0.589847 | 0.013188 | 0.686769 | 0.544651 | 0.075608 | 0.389738 | 0.515636 | 0.285684 | 0.039999 |
| MARC0051399 | 12 | 52231845 | 0.001366 | 0.915583 | 0.000277 | 0.051048 | 0.009587 | 0.691784 | 0.826964 | 0.002728 | 0.63208 | 0.134286 | 0.005723 | 0.009449 |
| ASGA0061721 | 14 | 16911337 | 0.001399 | 0.192123 | 0.019295 | 0.827592 | 0.203995 | 0.398154 | 0.838236 | 0.529796 | 0.146715 | 0.627938 | 0.182725 | 0.00023 |
| ALGA0036046 | 6 | 88024202 | 0.001444 | 0.427738 | 0.04331 | 0.059448 | 0.01016 | 0.624788 | 0.976674 | 0.081263 | 0.077526 | 0.152965 | 0.060913 | 0.213165 |
| MARC0112737 | 14 | 50917403 | 0.001495 | 0.488245 | 0.012084 | 0.943214 | 0.041955 | 0.23384 | 0.110865 | 0.044622 | 0.459251 | 0.854295 | 0.182654 | 0.497086 |
| CASI0006488 | 0 | 0 | 0.00154 | 0.304773 | 0.062646 | 0.563401 | 0.027981 | 0.424023 | 0.867159 | 0.134029 | 0.145054 | 0.201588 | 0.148376 | 0.127041 |
| ALGA0116962 | 0 | 0 | 0.001605 | 0.704304 | 0.005734 | 0.034432 | 0.605783 | 0.48532 | 0.058523 | 0.07924 | 0.505805 | 0.590557 | 0.055598 | 0.147731 |
| ASGA0085522 | 0 | 0 | 0.00163 | 0.229246 | 2E-06 | 0.238565 | 0.065258 | 0.084103 | 0.067573 | 0.005854 | 0.04917 | 0.775614 | 6.12E-05 | 0.000421 |
| DIAS0000949 | 6 | 1.37E+08 | 0.001735 | 0.710592 | 0.044446 | 0.323764 | 0.263918 | 0.860164 | 0.500899 | 0.644542 | 0.246365 | 0.704228 | 0.835425 | 0.31788 |
| ALGA0074780 | 14 | 7684355 | 0.00175 | 0.495723 | 0.027626 | 0.051373 | 0.383984 | 0.830546 | 0.719711 | 0.759721 | 0.806535 | 0.139629 | 0.035034 | 0.337782 |
| ALGA0106235 | 0 | 0 | 0.001798 | 0.78121 | 0.004712 | 0.436659 | 0.351067 | 0.9404 | 0.489654 | 0.10061 | 0.878024 | 0.670993 | 0.156774 | 0.034241 |
| ASGA0054989 | 12 | 51375066 | 0.001803 | 0.459201 | 0.00023 | 0.048392 | 0.018866 | 0.932412 | 0.682364 | 0.00608 | 0.777085 | 0.244584 | 0.003274 | 0.003407 |
| ASGA0022527 | 4 | 1.3E+08 | 0.001815 | 0.558919 | 0.009518 | 0.101424 | 0.069358 | 0.881761 | 0.855202 | 0.765506 | 0.600918 | 0.897522 | 0.959851 | 0.023945 |
| MARC0010680 | 6 | 45460886 | 0.001847 | 0.25026 | 0.022383 | 0.0631 | 0.01541 | 0.653515 | 0.782624 | 0.038428 | 0.150759 | 0.167973 | 0.158611 | 0.144875 |
| ALGA0066905 | 12 | 52804872 | 0.00185 | 0.857886 | 0.00034 | 0.054944 | 0.008697 | 0.742431 | 0.990869 | 0.002929 | 0.556516 | 0.212808 | 0.002399 | 0.010495 |
| ALGA0119023 | 12 | 59454215 | 0.001875 | 0.931982 | 0.000335 | 0.106724 | 0.013215 | 0.522247 | 0.611412 | 0.023079 | 0.061587 | 0.091282 | 0.00287 | 0.01586 |
| ALGA0066930 | 12 | 54679228 | 0.00191 | 0.152583 | 0.011827 | 0.99418 | 0.002688 | 0.064672 | 0.035062 | 0.000185 | 0.001875 | 0.941569 | 0.005869 | 0.003732 |
| ALGA0066729 | 12 | 50373701 | 0.001946 | 0.672232 | 0.000517 | 0.038404 | 0.021169 | 0.937842 | 0.720536 | 0.007855 | 0.799026 | 0.223706 | 0.003743 | 0.006458 |
| ALGA0120796 | 12 | 54066400 | 0.002121 | 0.561063 | 0.004659 | 0.654737 | 0.065697 | 0.145915 | 0.035423 | 0.05047 | 0.025038 | 0.503894 | 0.007642 | 0.011031 |
| ALGA0077379 | 14 | 48759426 | 0.002139 | 0.972881 | 0.045037 | 0.930339 | 0.00226 | 0.203521 | 0.068401 | 0.004731 | 0.618966 | 0.464044 | 0.1501 | 0.293195 |
| MARC0047822 | 14 | 48792477 | 0.00217 | 0.971994 | 0.044976 | 0.931686 | 0.00222 | 0.202554 | 0.068006 | 0.004641 | 0.617873 | 0.465556 | 0.15034 | 0.291964 |
| MARC0095891 | 0 | 0 | 0.00217 | 0.971994 | 0.044976 | 0.931686 | 0.00222 | 0.202554 | 0.068006 | 0.004641 | 0.617873 | 0.465556 | 0.15034 | 0.291964 |
| ASGA0023013 | 4 | 1.36E+08 | 0.002203 | 0.249341 | 0.000635 | 0.009428 | 0.423556 | 0.022475 | 0.006045 | 0.567492 | 0.074394 | 0.113953 | 0.332814 | 0.004768 |
| ALGA0107077 | 12 | 57123997 | 0.002207 | 0.214651 | 0.012608 | 0.572775 | 0.029005 | 0.327465 | 0.705097 | 0.038123 | 0.235301 | 0.380893 | 0.038858 | 0.003577 |
| INRA0009581 | 0 | 0 | 0.002223 | 0.363348 | 0.056965 | 0.316787 | 0.039431 | 0.549089 | 0.947007 | 0.415268 | 0.977384 | 0.99093 | 0.196685 | 0.403244 |
| ALGA0077433 | 14 | 50479636 | 0.002229 | 0.36654 | 0.029121 | 0.918347 | 0.041137 | 0.231862 | 0.073626 | 0.034185 | 0.491456 | 0.938279 | 0.232863 | 0.62131 |
| ALGA0113364 | 6 | 53254212 | 0.002265 | 0.301544 | 0.034215 | 0.060208 | 0.011689 | 0.608913 | 0.861413 | 0.034393 | 0.107989 | 0.160956 | 0.17154 | 0.183271 |
| ASGA0091097 | 6 | 53157140 | 0.002265 | 0.301544 | 0.034215 | 0.060208 | 0.011689 | 0.608913 | 0.861413 | 0.034393 | 0.107989 | 0.160956 | 0.17154 | 0.183271 |
| ALGA0075667 | 3 | 19164312 | 0.002398 | 0.280695 | 0.019423 | 0.748315 | 0.176461 | 0.34554 | 0.697071 | 0.469761 | 0.135808 | 0.802807 | 0.34236 | 0.000164 |
| MARC0031583 | 11 | 24221827 | 0.002559 | 0.713427 | 0.012361 | 0.026876 | 0.841712 | 0.570388 | 0.965082 | 0.822355 | 0.411749 | 0.224688 | 0.019589 | 0.187351 |
| DRGA0009658 | 9 | 1.15E+08 | 0.002569 | 0.108536 | 0.006917 | 0.083304 | 0.00965 | 0.949305 | 0.571638 | 0.670493 | 0.223567 | 0.195901 | 0.451379 | 0.022729 |
| CASI0006620 | 6 | 90352248 | 0.002569 | 0.681789 | 0.068502 | 0.131104 | 0.008613 | 0.568166 | 0.867318 | 0.142612 | 0.076457 | 0.243413 | 0.111018 | 0.395025 |
| ASGA0043629 | 9 | 71352198 | 0.002605 | 0.905396 | 0.00056 | 0.000397 | 0.369908 | 0.160185 | 0.183835 | 0.892455 | 0.983567 | 0.137954 | 0.570727 | 0.011021 |
| MARC0029403 | 2 | 1.27E+08 | 0.002645 | 0.642135 | 0.056158 | 0.676378 | 0.030674 | 0.234349 | 0.634416 | 0.412502 | 0.094948 | 0.191843 | 0.074087 | 0.583032 |
| M1GA0026465 | 0 | 0 | 0.002666 | 0.003921 | 0.000264 | 0.453609 | 0.0192 | 0.500452 | 0.727906 | 0.001221 | 0.189306 | 0.837795 | 0.021526 | 0.000231 |
| ASGA0055300 | 12 | 59921056 | 0.00267 | 0.020487 | 0.031894 | 0.408253 | 0.057287 | 0.554057 | 0.290927 | 0.001161 | 0.376077 | 0.909579 | 0.306832 | 0.007924 |
| H3GA0034708 | 12 | 54636448 | 0.002718 | 0.862961 | 0.058329 | 0.053662 | 0.44029 | 0.167784 | 0.036187 | 0.697155 | 0.461178 | 0.157699 | 0.999637 | 0.079912 |
| DRGA0013810 | 14 | 49925754 | 0.002758 | 0.416671 | 0.025084 | 0.889252 | 0.03498 | 0.185973 | 0.098333 | 0.034224 | 0.399167 | 0.955943 | 0.27484 | 0.554942 |
| INRA0044497 | 14 | 67670130 | 0.002803 | 0.132626 | 0.025861 | 0.187392 | 0.089993 | 0.795701 | 0.734396 | 0.990545 | 0.179195 | 0.408351 | 0.816332 | 0.07478 |
| ALGA0119981 | 0 | 0 | 0.002837 | 0.906796 | 0.039368 | 0.051176 | 0.247197 | 0.897479 | 0.842967 | 0.731515 | 0.805951 | 0.606871 | 0.476268 | 0.042838 |
| ALGA0077427 | 14 | 50406565 | 0.002859 | 0.371023 | 0.032716 | 0.947684 | 0.040511 | 0.23803 | 0.067788 | 0.034252 | 0.483993 | 0.982792 | 0.284244 | 0.623646 |
| ASGA0063229 | 14 | 49885158 | 0.002859 | 0.371023 | 0.032716 | 0.947684 | 0.040511 | 0.23803 | 0.067788 | 0.034252 | 0.483993 | 0.982792 | 0.284244 | 0.623646 |
| ASGA0063274 | 14 | 50964903 | 0.002859 | 0.371023 | 0.032716 | 0.947684 | 0.040511 | 0.23803 | 0.067788 | 0.034252 | 0.483993 | 0.982792 | 0.284244 | 0.623646 |
| H3GA0040085 | 14 | 49977303 | 0.002859 | 0.371023 | 0.032716 | 0.947684 | 0.040511 | 0.23803 | 0.067788 | 0.034252 | 0.483993 | 0.982792 | 0.284244 | 0.623646 |
| H3GA0040130 | 14 | 50992818 | 0.002859 | 0.371023 | 0.032716 | 0.947684 | 0.040511 | 0.23803 | 0.067788 | 0.034252 | 0.483993 | 0.982792 | 0.284244 | 0.623646 |
| INRA0043976 | 14 | 49990314 | 0.002859 | 0.371023 | 0.032716 | 0.947684 | 0.040511 | 0.23803 | 0.067788 | 0.034252 | 0.483993 | 0.982792 | 0.284244 | 0.623646 |
| INRA0043998 | 14 | 50652198 | 0.002859 | 0.371023 | 0.032716 | 0.947684 | 0.040511 | 0.23803 | 0.067788 | 0.034252 | 0.483993 | 0.982792 | 0.284244 | 0.623646 |
| ISU10000541 | 14 | 50261752 | 0.002859 | 0.371023 | 0.032716 | 0.947684 | 0.040511 | 0.23803 | 0.067788 | 0.034252 | 0.483993 | 0.982792 | 0.284244 | 0.623646 |
| MARC0093590 | 14 | 50614768 | 0.002859 | 0.371023 | 0.032716 | 0.947684 | 0.040511 | 0.23803 | 0.067788 | 0.034252 | 0.483993 | 0.982792 | 0.284244 | 0.623646 |
| MARC0094212 | 14 | 50568869 | 0.002859 | 0.371023 | 0.032716 | 0.947684 | 0.040511 | 0.23803 | 0.067788 | 0.034252 | 0.483993 | 0.982792 | 0.284244 | 0.623646 |
| ASGA0094334 | 13 | 19865120 | 0.002879 | 0.656575 | 0.036678 | 0.236031 | 0.429131 | 0.922163 | 0.021224 | 0.290919 | 0.450247 | 0.322206 | 0.750879 | 0.030681 |
| ALGA0077417 | 14 | 50106154 | 0.002993 | 0.404515 | 0.030011 | 0.931388 | 0.040497 | 0.215347 | 0.075057 | 0.032004 | 0.440834 | 0.855375 | 0.313792 | 0.591395 |
| ALGA0077425 | 14 | 50282254 | 0.002993 | 0.404515 | 0.030011 | 0.931388 | 0.040497 | 0.215347 | 0.075057 | 0.032004 | 0.440834 | 0.855375 | 0.313792 | 0.591395 |
| MARC0042884 | 14 | 50012997 | 0.00316 | 0.394202 | 0.036617 | 0.901076 | 0.029987 | 0.198501 | 0.06434 | 0.026574 | 0.429329 | 0.940094 | 0.330503 | 0.614733 |
| ASGA0086721 | 12 | 54332861 | 0.003171 | 0.66846 | 0.006294 | 0.009491 | 0.450236 | 0.062929 | 0.009099 | 0.584077 | 0.152481 | 0.262718 | 0.92479 | 0.032462 |
| ALGA0078789 | 14 | 79417669 | 0.003175 | 0.338471 | 0.019486 | 0.100814 | 0.401308 | 0.984972 | 0.637609 | 0.481133 | 0.395918 | 0.996896 | 0.29532 | 0.06601 |
| ALGA0077394 | 14 | 49438982 | 0.0032 | 0.341357 | 0.037289 | 0.974733 | 0.045635 | 0.224938 | 0.069219 | 0.037116 | 0.483823 | 0.954918 | 0.302314 | 0.66396 |
| DBNP0002145 | 14 | 49401845 | 0.0032 | 0.341357 | 0.037289 | 0.974733 | 0.045635 | 0.224938 | 0.069219 | 0.037116 | 0.483823 | 0.954918 | 0.302314 | 0.66396 |
| INRA0043964 | 14 | 49383214 | 0.0032 | 0.341357 | 0.037289 | 0.974733 | 0.045635 | 0.224938 | 0.069219 | 0.037116 | 0.483823 | 0.954918 | 0.302314 | 0.66396 |
| INRA0043965 | 0 | 0 | 0.0032 | 0.341357 | 0.037289 | 0.974733 | 0.045635 | 0.224938 | 0.069219 | 0.037116 | 0.483823 | 0.954918 | 0.302314 | 0.66396 |
| H3GA0031647 | 11 | 25106814 | 0.003214 | 0.793243 | 0.007186 | 0.002256 | 0.772258 | 0.074295 | 0.596213 | 0.531173 | 0.873236 | 0.413357 | 0.363512 | 0.064395 |
| ALGA0077409 | 14 | 49904380 | 0.00326 | 0.421357 | 0.039107 | 0.888462 | 0.029255 | 0.182876 | 0.088637 | 0.034504 | 0.409939 | 0.96202 | 0.28918 | 0.636814 |
| ALGA0077446 | 14 | 50761608 | 0.00326 | 0.421357 | 0.039107 | 0.888462 | 0.029255 | 0.182876 | 0.088637 | 0.034504 | 0.409939 | 0.96202 | 0.28918 | 0.636814 |
| ALGA0077450 | 14 | 50792395 | 0.00326 | 0.421357 | 0.039107 | 0.888462 | 0.029255 | 0.182876 | 0.088637 | 0.034504 | 0.409939 | 0.96202 | 0.28918 | 0.636814 |
| ALGA0077463 | 14 | 51006694 | 0.00326 | 0.421357 | 0.039107 | 0.888462 | 0.029255 | 0.182876 | 0.088637 | 0.034504 | 0.409939 | 0.96202 | 0.28918 | 0.636814 |
| ALGA0077464 | 14 | 51110365 | 0.00326 | 0.421357 | 0.039107 | 0.888462 | 0.029255 | 0.182876 | 0.088637 | 0.034504 | 0.409939 | 0.96202 | 0.28918 | 0.636814 |
| ASGA0063232 | 14 | 49949815 | 0.00326 | 0.421357 | 0.039107 | 0.888462 | 0.029255 | 0.182876 | 0.088637 | 0.034504 | 0.409939 | 0.96202 | 0.28918 | 0.636814 |
| ASGA0063238 | 14 | 50223650 | 0.00326 | 0.421357 | 0.039107 | 0.888462 | 0.029255 | 0.182876 | 0.088637 | 0.034504 | 0.409939 | 0.96202 | 0.28918 | 0.636814 |
| ASGA0063245 | 14 | 50243068 | 0.00326 | 0.421357 | 0.039107 | 0.888462 | 0.029255 | 0.182876 | 0.088637 | 0.034504 | 0.409939 | 0.96202 | 0.28918 | 0.636814 |
| ASGA0063261 | 14 | 50672335 | 0.00326 | 0.421357 | 0.039107 | 0.888462 | 0.029255 | 0.182876 | 0.088637 | 0.034504 | 0.409939 | 0.96202 | 0.28918 | 0.636814 |
| ASGA0063267 | 14 | 50776775 | 0.00326 | 0.421357 | 0.039107 | 0.888462 | 0.029255 | 0.182876 | 0.088637 | 0.034504 | 0.409939 | 0.96202 | 0.28918 | 0.636814 |
| ASGA0063279 | 14 | 51020901 | 0.00326 | 0.421357 | 0.039107 | 0.888462 | 0.029255 | 0.182876 | 0.088637 | 0.034504 | 0.409939 | 0.96202 | 0.28918 | 0.636814 |
| INRA0043972 | 14 | 49831490 | 0.00326 | 0.421357 | 0.039107 | 0.888462 | 0.029255 | 0.182876 | 0.088637 | 0.034504 | 0.409939 | 0.96202 | 0.28918 | 0.636814 |
| INRA0043983 | 14 | 50203210 | 0.00326 | 0.421357 | 0.039107 | 0.888462 | 0.029255 | 0.182876 | 0.088637 | 0.034504 | 0.409939 | 0.96202 | 0.28918 | 0.636814 |
| MARC0006678 | 14 | 50044448 | 0.00326 | 0.421357 | 0.039107 | 0.888462 | 0.029255 | 0.182876 | 0.088637 | 0.034504 | 0.409939 | 0.96202 | 0.28918 | 0.636814 |
| MARC0024032 | 14 | 50824208 | 0.00326 | 0.421357 | 0.039107 | 0.888462 | 0.029255 | 0.182876 | 0.088637 | 0.034504 | 0.409939 | 0.96202 | 0.28918 | 0.636814 |
| ASGA0011670 | 2 | 1.29E+08 | 0.003407 | 0.574578 | 0.088215 | 0.700023 | 0.048594 | 0.821964 | 0.649098 | 0.898347 | 0.464453 | 0.676688 | 0.501613 | 0.09906 |
| MARC0077968 | 9 | 58108396 | 0.003431 | 0.375255 | 0.031347 | 0.003945 | 0.021889 | 0.225587 | 0.254575 | 0.617776 | 0.803628 | 0.097705 | 0.839574 | 0.011609 |
| H3GA0040104 | 14 | 50313732 | 0.003468 | 0.450797 | 0.050231 | 0.93973 | 0.035024 | 0.220625 | 0.072708 | 0.038302 | 0.495913 | 0.974706 | 0.33082 | 0.658207 |
| ALGA0066960 | 12 | 55218591 | 0.003483 | 0.248189 | 0.088463 | 0.055524 | 0.017038 | 0.649262 | 0.012962 | 0.081508 | 0.5331 | 0.749788 | 0.707042 | 0.198764 |
| MARC0036724 | 14 | 49772549 | 0.003536 | 0.363188 | 0.041683 | 0.92813 | 0.033996 | 0.18691 | 0.065722 | 0.02889 | 0.429137 | 0.99721 | 0.350583 | 0.655008 |
| ALGA0066401 | 12 | 42685899 | 0.003554 | 0.585736 | 0.005175 | 0.558374 | 0.579517 | 0.423538 | 0.659462 | 0.100684 | 0.972018 | 0.99778 | 0.030682 | 0.018863 |
| ASGA0093543 | 12 | 60803086 | 0.003556 | 0.003484 | 0.00033 | 0.435099 | 0.020727 | 0.540182 | 0.746234 | 0.0013 | 0.203815 | 0.899827 | 0.019766 | 0.000301 |
| M1GA0026329 | 12 | 60802132 | 0.003556 | 0.003484 | 0.00033 | 0.435099 | 0.020727 | 0.540182 | 0.746234 | 0.0013 | 0.203815 | 0.899827 | 0.019766 | 0.000301 |
| H3GA0027713 | 9 | 71439437 | 0.003564 | 0.955494 | 0.001024 | 0.000271 | 0.361264 | 0.092364 | 0.113697 | 0.966087 | 0.891498 | 0.177205 | 0.863997 | 0.008128 |
| MARC0008762 | 14 | 49674562 | 0.00363 | 0.404504 | 0.041495 | 0.892192 | 0.035189 | 0.236824 | 0.097577 | 0.042392 | 0.369903 | 0.975979 | 0.355517 | 0.762552 |
| ALGA0045650 | 0 | 0 | 0.003635 | 0.694146 | 0.002144 | 0.048344 | 0.010307 | 0.968665 | 0.449287 | 0.537974 | 0.575742 | 0.69759 | 0.412484 | 0.054382 |
| ASGA0022861 | 4 | 1.34E+08 | 0.003665 | 0.761742 | 0.055875 | 0.066447 | 0.233602 | 0.969748 | 0.7359 | 0.866284 | 0.972822 | 0.645881 | 0.599521 | 0.047969 |
| ASGA0063597 | 14 | 60731369 | 0.00381 | 0.229364 | 0.043255 | 0.820883 | 0.088164 | 0.481023 | 0.185279 | 0.28221 | 0.430845 | 0.592682 | 0.708617 | 0.007723 |
| ALGA0122230 | 6 | 1.3E+08 | 0.003826 | 0.923006 | 0.117914 | 0.040761 | 0.105776 | 0.660305 | 0.359708 | 0.829544 | 0.460713 | 0.525783 | 0.607628 | 0.831234 |
| MARC0073795 | 14 | 46408884 | 0.003837 | 0.959727 | 0.098997 | 0.722134 | 0.015694 | 0.60375 | 0.251559 | 0.035683 | 0.600216 | 0.213916 | 0.458645 | 0.036509 |
| ASGA0043639 | 9 | 71483102 | 0.003875 | 0.797832 | 0.002453 | 0.000241 | 0.339746 | 0.161506 | 0.269314 | 0.915694 | 0.866275 | 0.328017 | 0.682355 | 0.011985 |
| ASGA0085741 | 0 | 0 | 0.00391 | 0.6935 | 0.147112 | 0.652144 | 0.046004 | 0.304051 | 0.523993 | 0.146203 | 0.28478 | 0.256858 | 0.876919 | 0.048137 |
| DRGA0005371 | 4 | 1.37E+08 | 0.003922 | 0.259337 | 0.003558 | 0.002483 | 0.608012 | 0.121984 | 0.050108 | 0.862249 | 0.158912 | 0.33464 | 0.60707 | 0.03651 |
| MARC0051912 | 13 | 1.37E+08 | 0.003946 | 0.065222 | 0.301218 | 0.001718 | 0.042793 | 0.312297 | 0.025456 | 0.359325 | 0.53014 | 0.194966 | 0.823088 | 0.028005 |
| H3GA0006388 | 2 | 27612628 | 0.003954 | 0.394695 | 0.109507 | 0.1982 | 0.428568 | 0.465476 | 0.218056 | 0.48325 | 0.720088 | 0.530233 | 0.976485 | 0.102512 |
| ALGA0123888 | 13 | 24391349 | 0.003994 | 0.881905 | 0.206407 | 0.777273 | 0.198669 | 0.298588 | 0.706893 | 0.589859 | 0.618628 | 0.263947 | 0.604612 | 0.041524 |
| MARC0044555 | 2 | 1.26E+08 | 0.004025 | 0.876406 | 0.013588 | 0.775817 | 0.086771 | 0.336035 | 0.391241 | 0.758795 | 0.248343 | 0.144319 | 0.010736 | 0.342115 |
| MARC0094795 | 12 | 52773511 | 0.004027 | 0.726757 | 0.001233 | 0.120159 | 0.028783 | 0.6106 | 0.866811 | 0.013363 | 0.505498 | 0.191988 | 0.008283 | 0.03381 |
| ALGA0066986 | 12 | 56093886 | 0.004045 | 0.116798 | 0.003566 | 0.540588 | 0.005184 | 0.27428 | 0.968423 | 0.000995 | 0.058151 | 0.7184 | 0.009808 | 0.004088 |
| MARC0024247 | 0 | 0 | 0.00405 | 0.364872 | 0.094444 | 0.129803 | 0.049371 | 0.97079 | 0.86181 | 0.266308 | 0.191636 | 0.485034 | 0.169396 | 0.231155 |
| ALGA0053536 | 9 | 67599042 | 0.004084 | 0.825303 | 0.016432 | 0.002054 | 0.060418 | 0.120387 | 0.295985 | 0.664248 | 0.726538 | 0.259446 | 0.659666 | 0.01008 |
| ASGA0105202 | 12 | 55918933 | 0.004114 | 0.355779 | 0.02368 | 0.104375 | 0.035874 | 0.852399 | 0.06668 | 0.045991 | 0.879007 | 0.308106 | 0.339357 | 0.074894 |
| ASGA0032135 | 7 | 29960357 | 0.004202 | 0.368619 | 0.02857 | 0.065977 | 0.282832 | 0.592491 | 0.133202 | 0.449474 | 0.834921 | 0.6442 | 0.754921 | 0.004387 |
| MARC0081365 | 6 | 68530065 | 0.004242 | 0.233488 | 0.023671 | 0.052646 | 0.038945 | 0.626213 | 0.920676 | 0.055986 | 0.090297 | 0.092737 | 0.05972 | 0.35903 |
| MARC0044698 | 12 | 42662420 | 0.004258 | 0.475897 | 0.006579 | 0.523856 | 0.62859 | 0.537489 | 0.6293 | 0.124043 | 0.8557 | 0.922835 | 0.032454 | 0.024637 |
| ALGA0123988 | 0 | 0 | 0.004264 | 0.303998 | 0.144791 | 0.204207 | 0.164555 | 0.867076 | 0.847773 | 0.996427 | 0.493956 | 0.844794 | 0.358977 | 0.275642 |
| MARC0085521 | 13 | 20991970 | 0.004327 | 0.884794 | 0.039679 | 0.474434 | 0.398181 | 0.62695 | 0.124242 | 0.389659 | 0.897282 | 0.384318 | 0.67341 | 0.032126 |
| ASGA0091260 | 13 | 1.97E+08 | 0.004351 | 0.198048 | 0.006858 | 0.222653 | 0.601483 | 0.427006 | 0.133621 | 0.127268 | 0.768752 | 0.435026 | 0.740198 | 0.180661 |
| ASGA0027983 | 6 | 28512075 | 0.004378 | 0.259769 | 0.029633 | 0.023702 | 0.031434 | 0.416675 | 0.930323 | 0.5024 | 0.964821 | 0.215051 | 0.669423 | 0.264095 |
| MARC0027759 | 12 | 57625866 | 0.004413 | 0.646557 | 4.37E-06 | 0.851037 | 0.043565 | 0.006897 | 0.014101 | 0.000894 | 0.021674 | 0.493181 | 2.05E-06 | 0.001345 |
| ALGA0068481 | 13 | 17492219 | 0.004478 | 0.677403 | 0.074939 | 0.263988 | 0.387275 | 0.700018 | 0.024127 | 0.154162 | 0.874585 | 0.988819 | 0.897703 | 0.022425 |
| ALGA0121166 | 9 | 72946896 | 0.004524 | 0.819598 | 0.022909 | 0.002184 | 0.099342 | 0.079466 | 0.174141 | 0.751892 | 0.511474 | 0.225305 | 0.438026 | 0.010027 |
| ASGA0099858 | 13 | 1.4E+08 | 0.004567 | 0.178881 | 0.226147 | 0.001522 | 0.13777 | 0.499376 | 0.125634 | 0.616239 | 0.347214 | 0.06236 | 0.869247 | 0.006497 |
| ALGA0066975 | 12 | 55864166 | 0.00469 | 0.530473 | 6.33E-05 | 0.060158 | 0.224156 | 0.653878 | 0.682498 | 0.121499 | 0.950548 | 0.362854 | 0.010587 | 0.002136 |
| ASGA0063228 | 14 | 49872197 | 0.004691 | 0.402936 | 0.051996 | 0.829343 | 0.032406 | 0.220331 | 0.056392 | 0.034421 | 0.512687 | 0.814494 | 0.399452 | 0.62819 |
| ALGA0077308 | 14 | 47386749 | 0.004715 | 0.356716 | 0.055009 | 0.895968 | 0.049383 | 0.27053 | 0.061674 | 0.047685 | 0.551113 | 0.920097 | 0.371331 | 0.735353 |
| ALGA0121140 | 9 | 63154704 | 0.004717 | 0.723835 | 0.005338 | 0.000127 | 0.074278 | 0.19851 | 0.20913 | 0.948301 | 0.653755 | 0.243515 | 0.928534 | 0.006589 |
| DRGA0009386 | 9 | 62966472 | 0.004717 | 0.723835 | 0.005338 | 0.000127 | 0.074278 | 0.19851 | 0.20913 | 0.948301 | 0.653755 | 0.243515 | 0.928534 | 0.006589 |
| MARC0089635 | 6 | 92353184 | 0.004767 | 0.590424 | 0.144458 | 0.080132 | 0.046538 | 0.938101 | 0.304347 | 0.940316 | 0.940671 | 0.440011 | 0.230573 | 0.216189 |
| ALGA0077486 | 14 | 51937785 | 0.004841 | 0.94164 | 0.113751 | 0.854957 | 0.001282 | 0.175024 | 0.055184 | 0.003298 | 0.573227 | 0.375867 | 0.234099 | 0.36804 |
| ASGA0063297 | 14 | 51606037 | 0.004841 | 0.94164 | 0.113751 | 0.854957 | 0.001282 | 0.175024 | 0.055184 | 0.003298 | 0.573227 | 0.375867 | 0.234099 | 0.36804 |
| DRGA0015493 | 15 | 1.3E+08 | 0.004842 | 0.851882 | 0.021939 | 0.345648 | 0.287395 | 0.889096 | 0.52719 | 0.051589 | 0.292031 | 0.759617 | 0.117476 | 0.366017 |
| ASGA0103675 | 0 | 0 | 0.004879 | 0.68832 | 0.07297 | 0.098877 | 0.096871 | 0.635589 | 0.278283 | 0.721989 | 0.966853 | 0.733575 | 0.522451 | 0.05073 |
| MARC0070309 | 6 | 1.48E+08 | 0.004886 | 0.467389 | 0.254955 | 0.207358 | 0.066085 | 0.969162 | 0.386883 | 0.228975 | 0.826104 | 0.492245 | 0.56524 | 0.160652 |
| ASGA0100140 | 12 | 43645196 | 0.004894 | 0.133157 | 0.069521 | 0.235441 | 0.072628 | 0.533818 | 0.164254 | 0.011925 | 0.80231 | 0.918815 | 0.408185 | 0.020898 |
| ASGA0063319 | 14 | 52686447 | 0.004931 | 0.997406 | 0.093677 | 0.79176 | 0.001118 | 0.170728 | 0.053039 | 0.002011 | 0.622399 | 0.562479 | 0.353757 | 0.34255 |
| ASGA0055092 | 12 | 55839256 | 0.004942 | 0.316684 | 0.06637 | 0.467997 | 0.035342 | 0.450244 | 0.666465 | 0.021947 | 0.110714 | 0.719553 | 0.202357 | 0.116467 |
| ALGA0111908 | 9 | 71711489 | 0.004963 | 0.973574 | 0.010563 | 0.006074 | 0.137843 | 0.108305 | 0.349392 | 0.708867 | 0.963112 | 0.329611 | 0.555859 | 0.004286 |
| M1GA0011274 | 7 | 1.29E+08 | 0.004995 | 0.894443 | 0.020798 | 0.292192 | 0.010093 | 0.585795 | 0.938393 | 0.298427 | 0.245917 | 0.609519 | 0.2881 | 0.170675 |
| ALGA0028718 | 4 | 1.3E+08 | 0.005056 | 0.552722 | 0.057818 | 0.19522 | 0.141788 | 0.505537 | 0.789952 | 0.843733 | 0.877662 | 0.791273 | 0.326139 | 0.022503 |
| CASI0008458 | 12 | 57466146 | 0.005088 | 0.417399 | 0.000525 | 0.586664 | 0.029978 | 0.166798 | 0.039507 | 0.002323 | 0.00784 | 0.305221 | 0.001869 | 0.003042 |
| INRA0050142 | 15 | 1.29E+08 | 0.005164 | 0.186994 | 0.0436 | 0.000523 | 0.337483 | 0.145197 | 0.148804 | 0.964515 | 0.685159 | 0.767677 | 0.247423 | 0.098337 |
| DIAS0000861 | 12 | 58132333 | 0.005173 | 0.53296 | 7.24E-07 | 0.177021 | 0.194722 | 0.351694 | 0.069157 | 0.003684 | 0.036065 | 0.632062 | 1.35E-05 | 0.000627 |
| MARC0054945 | 4 | 1.3E+08 | 0.005186 | 0.932544 | 0.008711 | 0.104108 | 0.371096 | 0.210202 | 0.126382 | 0.997527 | 0.9602 | 0.889632 | 0.985338 | 0.065469 |
| MARC0042944 | 2 | 1.07E+08 | 0.005253 | 0.043722 | 0.178152 | 0.149481 | 0.032624 | 0.972415 | 0.086587 | 0.13079 | 0.874372 | 0.336048 | 0.402452 | 0.046382 |
| ALGA0103994 | 12 | 60479145 | 0.005266 | 0.062066 | 0.000905 | 0.137358 | 0.142793 | 0.452267 | 0.851667 | 0.163011 | 0.741899 | 0.477009 | 0.047466 | 0.009259 |
| ASGA0029640 | 6 | 1.33E+08 | 0.00528 | 0.485905 | 0.018001 | 0.005424 | 0.652817 | 0.153189 | 0.520149 | 0.989924 | 0.620741 | 0.79482 | 0.346273 | 0.642612 |
| ASGA0022785 | 4 | 1.34E+08 | 0.005334 | 0.962228 | 0.008566 | 0.052461 | 0.420579 | 0.310079 | 0.138925 | 0.781804 | 0.499254 | 0.924802 | 0.637861 | 0.031119 |
| ASGA0083731 | 2 | 1.42E+08 | 0.00534 | 0.458223 | 0.206557 | 0.418901 | 0.328381 | 0.998055 | 0.16992 | 0.623629 | 0.821705 | 0.200111 | 0.705067 | 0.142024 |
| ALGA0077484 | 14 | 51869913 | 0.005346 | 0.87363 | 0.100392 | 0.781715 | 0.001279 | 0.1486 | 0.069472 | 0.004201 | 0.494194 | 0.291676 | 0.227539 | 0.332826 |
| ASGA0063176 | 14 | 47930150 | 0.005353 | 0.909897 | 0.134415 | 0.887072 | 0.001605 | 0.177664 | 0.063474 | 0.004381 | 0.553854 | 0.339546 | 0.265456 | 0.41883 |
| ASGA0063315 | 14 | 52402744 | 0.005355 | 0.949948 | 0.109421 | 0.809496 | 0.000777 | 0.146231 | 0.073776 | 0.003228 | 0.498035 | 0.519925 | 0.304571 | 0.377381 |
| ASGA0063323 | 14 | 52601343 | 0.005355 | 0.949948 | 0.109421 | 0.809496 | 0.000777 | 0.146231 | 0.073776 | 0.003228 | 0.498035 | 0.519925 | 0.304571 | 0.377381 |
| DIAS0001036 | 14 | 52621172 | 0.005355 | 0.949948 | 0.109421 | 0.809496 | 0.000777 | 0.146231 | 0.073776 | 0.003228 | 0.498035 | 0.519925 | 0.304571 | 0.377381 |
| H3GA0040160 | 14 | 52555810 | 0.005355 | 0.949948 | 0.109421 | 0.809496 | 0.000777 | 0.146231 | 0.073776 | 0.003228 | 0.498035 | 0.519925 | 0.304571 | 0.377381 |
| ASGA0063286 | 14 | 51173806 | 0.00536 | 0.999586 | 0.127759 | 0.809637 | 0.00087 | 0.140186 | 0.068559 | 0.00333 | 0.509937 | 0.34682 | 0.237731 | 0.375666 |
| ASGA0063293 | 14 | 51327964 | 0.00536 | 0.999586 | 0.127759 | 0.809637 | 0.00087 | 0.140186 | 0.068559 | 0.00333 | 0.509937 | 0.34682 | 0.237731 | 0.375666 |
| ASGA0063298 | 14 | 51646254 | 0.00536 | 0.999586 | 0.127759 | 0.809637 | 0.00087 | 0.140186 | 0.068559 | 0.00333 | 0.509937 | 0.34682 | 0.237731 | 0.375666 |
| ASGA0070241 | 15 | 1.14E+08 | 0.005479 | 0.86843 | 0.177277 | 0.184159 | 0.213978 | 0.696086 | 0.1743 | 0.534107 | 0.776634 | 0.361051 | 0.799615 | 0.048196 |
| ALGA0103808 | 0 | 0 | 0.005552 | 0.500722 | 0.120697 | 0.336941 | 0.153819 | 0.713786 | 0.189042 | 0.05217 | 0.85014 | 0.863791 | 0.733886 | 0.013138 |
| ASGA0025412 | 0 | 0 | 0.005567 | 0.171329 | 0.022498 | 0.437111 | 0.399087 | 0.293481 | 0.188077 | 0.40457 | 0.957915 | 0.367736 | 0.911744 | 0.007869 |
| ASGA0092862 | 6 | 1.37E+08 | 0.00562 | 0.625221 | 0.092272 | 0.04438 | 0.491265 | 0.505852 | 0.431674 | 0.398732 | 0.712004 | 0.888879 | 0.731458 | 0.609527 |
| H3GA0020098 | 7 | 16370134 | 0.005659 | 0.964145 | 0.164335 | 0.058792 | 0.473769 | 0.854923 | 0.63599 | 0.580356 | 0.908022 | 0.544222 | 0.776711 | 0.276937 |
| MARC0069201 | 14 | 58914805 | 0.005669 | 0.848117 | 0.036695 | 0.818271 | 0.002597 | 0.170736 | 0.129541 | 0.008636 | 0.702868 | 0.47093 | 0.141734 | 0.520494 |
| MARC0029326 | 6 | 66727937 | 0.00567 | 0.31269 | 0.0203 | 0.252319 | 0.029169 | 0.572328 | 0.537653 | 0.160767 | 0.046848 | 0.727591 | 0.115045 | 0.294803 |
| H3GA0048218 | 17 | 30132824 | 0.00568 | 0.814727 | 0.006735 | 0.616355 | 0.455551 | 0.555032 | 0.760001 | 0.755098 | 0.698531 | 0.329678 | 0.285201 | 0.129971 |
| H3GA0031535 | 11 | 21598394 | 0.005696 | 0.220076 | 0.019262 | 0.614006 | 0.618002 | 0.771058 | 0.883847 | 0.611284 | 0.586401 | 0.791647 | 0.215182 | 0.560606 |
| ALGA0028834 | 4 | 1.31E+08 | 0.005731 | 0.705052 | 0.021734 | 0.070588 | 0.364263 | 0.975147 | 0.777256 | 0.528399 | 0.71723 | 0.627122 | 0.42598 | 0.069517 |
| ALGA0067055 | 12 | 56800080 | 0.005738 | 0.452829 | 0.000987 | 0.010979 | 0.164938 | 0.194797 | 0.032583 | 0.105758 | 0.666942 | 0.304043 | 0.169921 | 0.023087 |
| H3GA0040153 | 14 | 52286293 | 0.005755 | 0.974207 | 0.116766 | 0.786474 | 0.000828 | 0.126227 | 0.079129 | 0.003622 | 0.483591 | 0.404765 | 0.27082 | 0.386215 |
| H3GA0056191 | 0 | 0 | 0.005766 | 0.856442 | 0.069435 | 0.083767 | 0.205699 | 0.944528 | 0.774893 | 0.848293 | 0.890998 | 0.608558 | 0.707898 | 0.058731 |
| DIAS0002565 | 4 | 1.31E+08 | 0.005801 | 0.766542 | 0.015418 | 0.048952 | 0.691572 | 0.370066 | 0.550407 | 0.392433 | 0.78279 | 0.758219 | 0.771864 | 0.05934 |
| H3GA0031190 | 11 | 6903915 | 0.005817 | 0.336285 | 0.122875 | 0.015134 | 0.048462 | 0.990744 | 0.627957 | 0.946683 | 0.742091 | 0.320747 | 0.308759 | 0.474152 |
| ASGA0085359 | 0 | 0 | 0.005824 | 0.726088 | 0.018511 | 0.491669 | 0.156345 | 0.631681 | 0.196489 | 0.687416 | 0.45469 | 0.657903 | 0.390441 | 0.151236 |
| ALGA0061162 | 11 | 18500507 | 0.005851 | 0.225417 | 0.21681 | 0.01055 | 0.041228 | 0.964954 | 0.938267 | 0.953946 | 0.635413 | 0.792247 | 0.13627 | 0.329374 |
| DRGA0006102 | 5 | 86520889 | 0.005856 | 0.504578 | 0.033515 | 0.074178 | 0.132322 | 0.333102 | 0.738293 | 0.178066 | 0.268344 | 0.937531 | 0.106985 | 0.079677 |
| ALGA0103480 | 12 | 57016714 | 0.005857 | 0.676876 | 0.097176 | 0.273489 | 0.060681 | 0.862106 | 0.674488 | 0.037392 | 0.216603 | 0.848988 | 0.44051 | 0.16546 |
| ASGA0055316 | 12 | 59580256 | 0.005861 | 0.022022 | 0.028001 | 0.413503 | 0.45532 | 0.284211 | 0.111337 | 0.073157 | 0.821895 | 0.230457 | 0.879556 | 0.004349 |
| ASGA0047716 | 10 | 45331909 | 0.005893 | 0.065896 | 0.15729 | 0.238834 | 0.001969 | 0.715025 | 0.754146 | 0.179389 | 0.028857 | 0.206449 | 0.802348 | 0.536639 |
| ALGA0117283 | 16 | 24475791 | 0.005895 | 0.746297 | 0.008198 | 0.143071 | 0.039586 | 0.491909 | 0.519296 | 0.26238 | 0.511421 | 0.089697 | 0.089479 | 0.067263 |
| MARC0089468 | 15 | 1.14E+08 | 0.005943 | 0.90346 | 0.190472 | 0.196373 | 0.196406 | 0.747956 | 0.195868 | 0.536527 | 0.733996 | 0.378667 | 0.794628 | 0.049251 |
| M1GA0015042 | 11 | 21568871 | 0.005949 | 0.654459 | 0.086084 | 0.107374 | 0.447814 | 0.838761 | 0.628047 | 0.475924 | 0.203678 | 0.459772 | 0.197392 | 0.213357 |
| H3GA0034813 | 12 | 56827665 | 0.00596 | 0.45273 | 0.000797 | 0.00951 | 0.203846 | 0.164518 | 0.027743 | 0.103649 | 0.519533 | 0.37596 | 0.162798 | 0.020929 |
| ASGA0063186 | 14 | 48076374 | 0.005992 | 0.955004 | 0.141463 | 0.835512 | 0.001049 | 0.131942 | 0.070245 | 0.003725 | 0.510032 | 0.309978 | 0.253895 | 0.406435 |
| ASGA0063205 | 14 | 48562060 | 0.005992 | 0.955004 | 0.141463 | 0.835512 | 0.001049 | 0.131942 | 0.070245 | 0.003725 | 0.510032 | 0.309978 | 0.253895 | 0.406435 |
| MARC0030251 | 14 | 48356407 | 0.005992 | 0.955004 | 0.141463 | 0.835512 | 0.001049 | 0.131942 | 0.070245 | 0.003725 | 0.510032 | 0.309978 | 0.253895 | 0.406435 |
| ALGA0077324 | 14 | 47529480 | 0.006026 | 0.886433 | 0.142868 | 0.993007 | 0.002134 | 0.244003 | 0.047935 | 0.006247 | 0.660136 | 0.340883 | 0.315927 | 0.435851 |
| H3GA0053415 | 0 | 0 | 0.006073 | 0.217785 | 0.067852 | 0.119839 | 0.473076 | 0.421551 | 0.175628 | 0.553999 | 0.826048 | 0.812503 | 0.390755 | 0.278886 |
| ALGA0120329 | 13 | 1.97E+08 | 0.006206 | 0.309478 | 0.083664 | 0.057891 | 0.162093 | 0.319798 | 0.006135 | 0.057542 | 0.633472 | 0.00891 | 0.290958 | 0.061291 |
| ASGA0105016 | 14 | 8658560 | 0.006217 | 0.586986 | 0.127116 | 0.17322 | 0.356208 | 0.687167 | 0.393286 | 0.412472 | 0.857175 | 0.199325 | 0.710651 | 0.008368 |
| ASGA0028753 | 6 | 80624462 | 0.006283 | 0.761269 | 0.090646 | 0.082158 | 0.014453 | 0.716765 | 0.886989 | 0.165786 | 0.070897 | 0.22438 | 0.06516 | 0.542024 |
| DRGA0015422 | 15 | 1.24E+08 | 0.006328 | 0.553894 | 0.091674 | 0.39151 | 0.044383 | 0.309668 | 0.861055 | 0.048513 | 0.051921 | 0.776506 | 0.21808 | 0.402761 |
| ALGA0120651 | 12 | 60466900 | 0.006383 | 0.21333 | 0.171907 | 0.845815 | 0.009281 | 0.316067 | 0.753444 | 0.058733 | 0.187778 | 0.901048 | 0.614477 | 0.069071 |
| ASGA0063147 | 14 | 47229793 | 0.006395 | 0.9464 | 0.150508 | 0.974379 | 0.002281 | 0.266286 | 0.048661 | 0.006333 | 0.66611 | 0.34073 | 0.325317 | 0.427635 |
| ASGA0104638 | 15 | 38675000 | 0.00641 | 0.422744 | 0.008568 | 0.098404 | 0.467162 | 0.885776 | 0.940393 | 0.758379 | 0.768396 | 0.355216 | 0.060735 | 0.384522 |
| ALGA0106317 | 11 | 20902453 | 0.006478 | 0.443553 | 0.457183 | 0.093261 | 0.101728 | 0.24333 | 0.351552 | 0.242144 | 0.632663 | 0.754592 | 0.781843 | 0.036486 |
| H3GA0034580 | 12 | 49990871 | 0.006518 | 0.700626 | 0.000745 | 0.062318 | 0.022764 | 0.88817 | 0.791415 | 0.010675 | 0.574577 | 0.26319 | 0.003656 | 0.031041 |
| MARC0110411 | 13 | 1.99E+08 | 0.006526 | 0.619491 | 0.168522 | 0.391867 | 0.112709 | 0.810399 | 0.054735 | 0.004284 | 0.557171 | 0.168097 | 0.699131 | 0.038237 |
| ALGA0037516 | 6 | 1.45E+08 | 0.00655 | 0.95717 | 0.307719 | 0.022437 | 0.125783 | 0.16134 | 0.131762 | 0.070953 | 0.475249 | 0.989147 | 0.943429 | 0.085606 |
| MARC0022997 | 13 | 18890472 | 0.006656 | 0.768907 | 0.022052 | 0.420338 | 0.69917 | 0.716018 | 0.549653 | 0.976065 | 0.95326 | 0.364826 | 0.258675 | 0.059992 |
| CASI0011743 | 12 | 50168066 | 0.006673 | 0.641005 | 0.000703 | 0.059018 | 0.021712 | 0.877905 | 0.783734 | 0.010023 | 0.558414 | 0.25759 | 0.002873 | 0.029052 |
| ASGA0039476 | 8 | 1.1E+08 | 0.006715 | 0.726797 | 0.065434 | 0.12371 | 0.650242 | 0.799438 | 0.209203 | 0.678725 | 0.560433 | 0.177324 | 0.176618 | 0.027157 |
| ASGA0017123 | 4 | 2684210 | 0.006721 | 0.972856 | 0.019825 | 0.074034 | 0.111721 | 0.25238 | 0.233246 | 0.58258 | 0.810074 | 0.902682 | 0.289434 | 0.332362 |
| M1GA0016964 | 12 | 55801721 | 0.006757 | 0.792146 | 0.000107 | 0.452914 | 0.042671 | 0.132305 | 0.015441 | 0.001225 | 0.007748 | 0.944983 | 7.91E-05 | 0.005608 |
| ASGA0072659 | 16 | 24615470 | 0.006773 | 0.614524 | 0.026853 | 0.178911 | 0.010251 | 0.572322 | 0.275654 | 0.278612 | 0.219886 | 0.416138 | 0.358734 | 0.059046 |
| ALGA0066707 | 12 | 49872027 | 0.006782 | 0.742028 | 0.000746 | 0.07321 | 0.022535 | 0.805857 | 0.880932 | 0.012124 | 0.541506 | 0.195351 | 0.004821 | 0.032275 |
| MARC0052739 | 13 | 18888739 | 0.006853 | 0.763863 | 0.024091 | 0.434845 | 0.701795 | 0.718738 | 0.569177 | 0.977049 | 0.953089 | 0.332826 | 0.242773 | 0.0659 |
| ASGA0054824 | 12 | 47292193 | 0.006856 | 0.295886 | 0.024528 | 0.615341 | 0.002879 | 0.031629 | 0.252073 | 0.000113 | 0.074745 | 0.627612 | 0.045568 | 0.00726 |
| ALGA0053842 | 9 | 80174286 | 0.006861 | 0.335994 | 0.070597 | 0.004036 | 0.067878 | 0.370525 | 0.089001 | 0.817609 | 0.902661 | 0.173628 | 0.687458 | 0.056102 |
| ISU10000717 | 9 | 80235109 | 0.006861 | 0.335994 | 0.070597 | 0.004036 | 0.067878 | 0.370525 | 0.089001 | 0.817609 | 0.902661 | 0.173628 | 0.687458 | 0.056102 |
| ALGA0071712 | 13 | 1.2E+08 | 0.00687 | 0.457449 | 0.15186 | 0.107974 | 0.430142 | 0.944273 | 0.090716 | 0.546738 | 0.588986 | 0.836865 | 0.959963 | 0.008713 |
| ALGA0053099 | 9 | 57557876 | 0.006887 | 0.491898 | 0.01719 | 0.002742 | 0.078836 | 0.128586 | 0.275691 | 0.720733 | 0.921515 | 0.110463 | 0.608856 | 0.034677 |
| ASGA0101662 | 6 | 67268784 | 0.0069 | 0.879969 | 0.039257 | 0.085684 | 0.038617 | 0.843241 | 0.78246 | 0.292981 | 0.140976 | 0.538776 | 0.030207 | 0.516302 |
| H3GA0045193 | 15 | 1.39E+08 | 0.00694 | 0.613895 | 0.024798 | 0.318917 | 0.929744 | 0.572814 | 0.477852 | 0.495683 | 0.733974 | 0.740386 | 0.082439 | 0.043576 |
| ASGA0011613 | 2 | 1.26E+08 | 0.006967 | 0.506558 | 0.007661 | 0.582814 | 0.308721 | 0.674592 | 0.881651 | 0.972189 | 0.523503 | 0.269739 | 0.019977 | 0.37481 |
| ALGA0074865 | 14 | 9089555 | 0.006973 | 0.749865 | 0.105843 | 0.113609 | 0.760702 | 0.496628 | 0.305727 | 0.905009 | 0.347775 | 0.89237 | 0.921069 | 0.186623 |
| DIAS0000441 | 13 | 1.31E+08 | 0.007002 | 0.976852 | 0.078899 | 0.077711 | 0.150235 | 0.845694 | 0.260561 | 0.553625 | 0.805029 | 0.300107 | 0.94237 | 0.068672 |
| ALGA0104524 | 12 | 53965746 | 0.007075 | 0.039037 | 0.092578 | 0.510151 | 0.034253 | 0.495018 | 0.414388 | 0.120615 | 0.76857 | 0.649805 | 0.492701 | 0.078452 |
| ALGA0074557 | 14 | 5575601 | 0.007085 | 0.447897 | 0.080283 | 0.026408 | 0.207396 | 0.282768 | 0.392569 | 0.743827 | 0.965089 | 0.992635 | 0.611441 | 0.282911 |
| ALGA0078240 | 14 | 66320818 | 0.007112 | 0.857418 | 0.046884 | 0.930143 | 0.007975 | 0.214287 | 0.614108 | 0.129964 | 0.347464 | 0.597375 | 0.307264 | 0.227198 |
| ALGA0086873 | 15 | 1.28E+08 | 0.007117 | 0.990855 | 0.015315 | 0.27376 | 0.144351 | 0.666384 | 0.263624 | 0.029888 | 0.206119 | 0.424905 | 0.473277 | 0.289864 |
| MARC0034353 | 14 | 46983442 | 0.007136 | 0.93918 | 0.155122 | 0.965176 | 0.002192 | 0.272693 | 0.04588 | 0.006067 | 0.664911 | 0.37098 | 0.348769 | 0.448907 |
| ASGA0065190 | 14 | 97758410 | 0.007175 | 0.732058 | 0.059047 | 0.764122 | 0.075782 | 0.083807 | 0.48437 | 0.274021 | 0.187414 | 0.578893 | 0.528836 | 0.012186 |
| MARC0050311 | 8 | 67597907 | 0.007199 | 0.564271 | 0.002089 | 0.01842 | 0.955384 | 0.204048 | 0.30723 | 0.541676 | 0.871269 | 0.655939 | 0.206717 | 0.061013 |
| ALGA0081834 | 14 | 1.36E+08 | 0.0072 | 0.676331 | 0.242286 | 0.036122 | 0.09223 | 0.702849 | 0.123089 | 0.289231 | 0.532143 | 0.950468 | 0.405699 | 0.387531 |
| ALGA0117657 | 0 | 0 | 0.007315 | 0.930619 | 0.036597 | 0.040996 | 0.013503 | 0.884062 | 0.537619 | 0.06569 | 0.09344 | 0.264669 | 0.046269 | 0.583643 |
| ASGA0094661 | 6 | 71878583 | 0.007392 | 0.319839 | 0.006391 | 0.089098 | 0.093408 | 0.503463 | 0.595506 | 0.02727 | 0.493518 | 0.893553 | 0.280691 | 0.150181 |
| DIAS0000187 | 6 | 71876872 | 0.007392 | 0.319839 | 0.006391 | 0.089098 | 0.093408 | 0.503463 | 0.595506 | 0.02727 | 0.493518 | 0.893553 | 0.280691 | 0.150181 |
| DIAS0003072 | 0 | 0 | 0.007392 | 0.319839 | 0.006391 | 0.089098 | 0.093408 | 0.503463 | 0.595506 | 0.02727 | 0.493518 | 0.893553 | 0.280691 | 0.150181 |
| MARC0073796 | 14 | 46409010 | 0.007532 | 0.913928 | 0.14892 | 0.858236 | 0.014205 | 0.552068 | 0.378715 | 0.071825 | 0.575232 | 0.221471 | 0.494572 | 0.05336 |
| MARC0040397 | 5 | 12659477 | 0.007539 | 0.707239 | 0.160993 | 0.235256 | 0.8535 | 0.546066 | 0.201737 | 0.650317 | 0.290206 | 0.271853 | 0.02144 | 0.196071 |
| ASGA0104019 | 4 | 19262923 | 0.00754 | 0.441929 | 0.05796 | 0.034329 | 0.377758 | 0.65071 | 0.883857 | 0.839014 | 0.446152 | 0.504204 | 0.050763 | 0.149765 |
| ALGA0118591 | 4 | 54919219 | 0.007561 | 0.095686 | 0.090433 | 0.195917 | 0.08462 | 0.640571 | 0.368251 | 0.522688 | 0.378979 | 0.739622 | 0.861381 | 0.361923 |
| H3GA0001444 | 1 | 38635057 | 0.007583 | 0.681407 | 0.03951 | 0.946344 | 0.176944 | 0.677581 | 0.828317 | 0.116549 | 0.473261 | 0.71404 | 0.868961 | 0.012487 |
| MARC0086017 | 12 | 53795262 | 0.007627 | 0.71035 | 0.072664 | 0.018292 | 0.398674 | 0.086636 | 0.015599 | 0.778376 | 0.198972 | 0.461685 | 0.975774 | 0.11571 |
| ALGA0121169 | 6 | 71914783 | 0.007658 | 0.318509 | 0.006611 | 0.090463 | 0.081867 | 0.492596 | 0.559806 | 0.025393 | 0.501884 | 0.837675 | 0.303659 | 0.149522 |
| ASGA0099154 | 9 | 75435052 | 0.00769 | 0.788406 | 0.02821 | 0.031305 | 0.431584 | 0.642712 | 0.270928 | 0.642739 | 0.633648 | 0.112481 | 0.759769 | 0.021771 |
| ALGA0058142 | 10 | 33153012 | 0.007692 | 0.381952 | 0.139256 | 0.330202 | 0.003257 | 0.556485 | 0.77443 | 0.18118 | 0.040672 | 0.088957 | 0.606977 | 0.094794 |
| DRGA0011751 | 12 | 38196871 | 0.007728 | 0.550684 | 0.259097 | 0.422996 | 0.001893 | 0.043135 | 0.967241 | 0.000214 | 0.036686 | 0.404173 | 0.336259 | 0.060634 |
| MARC0034221 | 4 | 2035615 | 0.007737 | 0.365723 | 0.145604 | 0.060763 | 0.008662 | 0.93072 | 0.268133 | 0.264219 | 0.277403 | 0.910978 | 0.229085 | 0.48358 |
| H3GA0025203 | 8 | 1.04E+08 | 0.007778 | 0.946358 | 0.091994 | 0.027495 | 0.417603 | 0.660575 | 0.193609 | 0.187684 | 0.291661 | 0.496289 | 0.115908 | 0.336302 |
| MARC0069132 | 4 | 1.31E+08 | 0.00783 | 0.66191 | 0.037997 | 0.008581 | 0.212629 | 0.122443 | 0.391416 | 0.929178 | 0.555063 | 0.569377 | 0.923623 | 0.004742 |
| MARC0007223 | 2 | 29680871 | 0.007866 | 0.609326 | 0.059244 | 0.211905 | 0.367503 | 0.837665 | 0.535607 | 0.553673 | 0.911768 | 0.613226 | 0.825773 | 0.148752 |
| MARC0097508 | 13 | 1.97E+08 | 0.007869 | 0.352621 | 0.099197 | 0.566271 | 0.171942 | 0.997729 | 0.139648 | 0.018279 | 0.413397 | 0.269073 | 0.775837 | 0.056241 |
| ALGA0037624 | 6 | 1.52E+08 | 0.007893 | 0.569969 | 0.12293 | 0.356469 | 0.039561 | 0.387146 | 0.909438 | 0.023634 | 0.0601 | 0.874982 | 0.176317 | 0.227516 |
| ASGA0083592 | 5 | 67959560 | 0.007978 | 0.187485 | 0.393913 | 0.680188 | 0.069843 | 0.350575 | 0.993781 | 0.087638 | 0.440685 | 0.592282 | 0.284305 | 0.146356 |
| ASGA0059126 | 13 | 1.59E+08 | 0.007987 | 0.247303 | 0.384119 | 0.287559 | 0.011762 | 0.50192 | 0.096971 | 0.084278 | 0.366475 | 0.108227 | 0.980363 | 0.078582 |
| MARC0021429 | 9 | 92569522 | 0.007998 | 0.571225 | 0.034877 | 0.253973 | 0.259817 | 0.232661 | 0.658078 | 0.562934 | 0.169231 | 0.958707 | 0.103806 | 0.099647 |
| MARC0026342 | 1 | 42229637 | 0.008056 | 0.654846 | 0.02727 | 0.87415 | 0.240446 | 0.77063 | 0.716023 | 0.209665 | 0.580822 | 0.639192 | 0.989035 | 0.010701 |
| ASGA0025408 | 0 | 0 | 0.008068 | 0.834482 | 0.081387 | 0.437644 | 0.330254 | 0.923991 | 0.350702 | 0.66937 | 0.755561 | 0.169857 | 0.609849 | 0.045501 |
| ASGA0029617 | 6 | 1.3E+08 | 0.008108 | 0.641364 | 0.062001 | 0.195083 | 0.704767 | 0.957455 | 0.882009 | 0.941567 | 0.65518 | 0.374945 | 0.357231 | 0.864408 |
| MARC0004712 | 0 | 0 | 0.00812 | 0.668635 | 9.85E-07 | 0.232772 | 0.201659 | 0.359399 | 0.051772 | 0.003183 | 0.026951 | 0.616684 | 7.55E-06 | 0.000459 |
| ALGA0071822 | 13 | 1.28E+08 | 0.008156 | 0.393144 | 0.187544 | 0.091981 | 0.303683 | 0.992441 | 0.076582 | 0.449572 | 0.500392 | 0.999351 | 0.959652 | 0.037072 |
| H3GA0018480 | 6 | 87321831 | 0.008176 | 0.692597 | 0.117871 | 0.132474 | 0.041895 | 0.927177 | 0.514494 | 0.700584 | 0.716574 | 0.409759 | 0.292565 | 0.337 |
| ALGA0077469 | 14 | 51195960 | 0.008183 | 0.457772 | 0.064294 | 0.757946 | 0.038296 | 0.182217 | 0.095525 | 0.03273 | 0.501088 | 0.856245 | 0.347672 | 0.885964 |
| H3GA0040144 | 14 | 51732537 | 0.008183 | 0.457772 | 0.064294 | 0.757946 | 0.038296 | 0.182217 | 0.095525 | 0.03273 | 0.501088 | 0.856245 | 0.347672 | 0.885964 |
| MARC0008226 | 2 | 1.28E+08 | 0.008188 | 0.262193 | 0.026932 | 0.541032 | 0.078868 | 0.072236 | 0.736038 | 0.198578 | 0.044843 | 0.61286 | 0.148861 | 0.596036 |
| ALGA0071819 | 13 | 1.27E+08 | 0.008216 | 0.376875 | 0.205899 | 0.085295 | 0.308384 | 0.921679 | 0.065972 | 0.431452 | 0.45198 | 0.992871 | 0.989759 | 0.039334 |
| H3GA0037212 | 13 | 1.27E+08 | 0.008216 | 0.376875 | 0.205899 | 0.085295 | 0.308384 | 0.921679 | 0.065972 | 0.431452 | 0.45198 | 0.992871 | 0.989759 | 0.039334 |
| DRGA0013344 | 13 | 1.97E+08 | 0.008231 | 0.389244 | 0.107826 | 0.591974 | 0.224986 | 0.957788 | 0.117415 | 0.020037 | 0.447263 | 0.291244 | 0.61593 | 0.050001 |
| ASGA0070140 | 15 | 95218876 | 0.008245 | 0.965378 | 0.022204 | 0.148572 | 0.148728 | 0.545917 | 0.101563 | 0.108889 | 0.5757 | 0.12019 | 0.837777 | 0.255111 |
| ASGA0060622 | 14 | 5140018 | 0.00826 | 0.572603 | 0.029323 | 0.84911 | 0.108534 | 0.385928 | 0.169524 | 0.08286 | 0.094018 | 0.615913 | 0.783119 | 0.11482 |
| ASGA0060992 | 14 | 9053896 | 0.008314 | 0.63452 | 0.045996 | 0.006773 | 0.429334 | 0.154487 | 0.279127 | 0.724913 | 0.740415 | 0.475261 | 0.361503 | 0.372332 |
| ALGA0015670 | 2 | 1.3E+08 | 0.008322 | 0.681413 | 0.03394 | 0.657371 | 0.151278 | 0.186238 | 0.088081 | 0.582609 | 0.050307 | 0.210788 | 0.006916 | 0.423362 |
| MARC0065219 | 9 | 1.36E+08 | 0.008378 | 0.43021 | 0.098284 | 0.204269 | 0.435162 | 0.682552 | 0.231001 | 0.555489 | 0.095956 | 0.359166 | 0.591347 | 0.231112 |
| H3GA0052434 | 6 | 71496000 | 0.008383 | 0.465523 | 0.026788 | 0.079071 | 0.030419 | 0.663217 | 0.914004 | 0.148753 | 0.229763 | 0.608299 | 0.288124 | 0.169222 |
| ALGA0110206 | 18 | 28845095 | 0.008386 | 0.279838 | 0.036977 | 0.17213 | 0.524398 | 0.33451 | 0.462368 | 0.524729 | 0.370049 | 0.828194 | 0.549969 | 0.266037 |
| MARC0043480 | 10 | 63867699 | 0.008402 | 0.74605 | 0.0169 | 0.091485 | 0.146111 | 0.617065 | 0.102354 | 0.612668 | 0.26512 | 0.984412 | 0.689658 | 0.046943 |
| ALGA0039828 | 7 | 30166967 | 0.00854 | 0.9248 | 0.034878 | 0.08944 | 0.347582 | 0.955222 | 0.240312 | 0.830994 | 0.19051 | 0.715262 | 0.193347 | 0.025513 |
| ALGA0072397 | 13 | 1.59E+08 | 0.008557 | 0.213963 | 0.373177 | 0.288624 | 0.01359 | 0.498942 | 0.100321 | 0.090506 | 0.364808 | 0.1099 | 0.96731 | 0.079197 |
| H3GA0005137 | 0 | 0 | 0.008571 | 0.839944 | 0.155576 | 0.013873 | 0.216577 | 0.13429 | 0.010122 | 0.185658 | 0.185203 | 0.500766 | 0.87393 | 0.333173 |
| MARC0099960 | 12 | 42797135 | 0.00859 | 0.401964 | 0.013756 | 0.503166 | 0.656766 | 0.385495 | 0.799875 | 0.372252 | 0.776263 | 0.651153 | 0.009704 | 0.084289 |
| ALGA0002763 | 1 | 42916036 | 0.008648 | 0.633466 | 0.028411 | 0.780885 | 0.292243 | 0.900905 | 0.658258 | 0.257053 | 0.694768 | 0.746351 | 0.915181 | 0.011368 |
| ALGA0086875 | 15 | 1.28E+08 | 0.008655 | 0.801961 | 0.015106 | 0.248722 | 0.091183 | 0.806444 | 0.259319 | 0.022932 | 0.130551 | 0.778783 | 0.275173 | 0.393958 |
| ASGA0089115 | 4 | 1.34E+08 | 0.008661 | 0.933285 | 0.000394 | 0.255077 | 0.082992 | 0.573306 | 0.179284 | 0.003004 | 0.252826 | 0.56026 | 0.000366 | 0.008732 |
| ALGA0073365 | 13 | 1.98E+08 | 0.008728 | 0.397157 | 0.083076 | 0.115796 | 0.262143 | 0.503046 | 0.09088 | 0.004997 | 0.704095 | 0.040416 | 0.660877 | 0.00564 |
| ALGA0011510 | 2 | 5075058 | 0.008812 | 0.272217 | 0.064501 | 0.310173 | 0.408114 | 0.476875 | 0.220659 | 0.445554 | 0.35237 | 0.467669 | 0.565613 | 0.046102 |
| ASGA0022543 | 4 | 1.3E+08 | 0.008816 | 0.927336 | 0.076714 | 0.258603 | 0.105186 | 0.708783 | 0.398532 | 0.339015 | 0.605023 | 0.62612 | 0.854017 | 0.018403 |
| ALGA0120042 | 12 | 42880363 | 0.008816 | 0.417366 | 0.01359 | 0.530494 | 0.618678 | 0.387198 | 0.783778 | 0.384961 | 0.759992 | 0.644593 | 0.009041 | 0.093974 |
| ALGA0074871 | 14 | 9149685 | 0.008817 | 0.912333 | 0.111377 | 0.111748 | 0.82392 | 0.497365 | 0.302598 | 0.944322 | 0.357014 | 0.935341 | 0.933569 | 0.199169 |
| DRGA0011511 | 11 | 80671962 | 0.008842 | 0.972669 | 0.064431 | 0.194689 | 0.447466 | 0.268783 | 0.103917 | 0.627219 | 0.448989 | 0.709949 | 0.627883 | 0.233015 |
| ASGA0100525 | 12 | 57622308 | 0.00885 | 0.898686 | 2.49E-06 | 0.454447 | 0.038684 | 0.018706 | 0.039631 | 0.001259 | 0.022932 | 0.792579 | 1.52E-05 | 0.002244 |
| ALGA0116086 | 12 | 45275338 | 0.008853 | 0.80284 | 0.012069 | 0.024509 | 0.131534 | 0.941016 | 0.315322 | 0.029561 | 0.693922 | 0.572118 | 0.022631 | 0.333094 |
| H3GA0040467 | 14 | 63490479 | 0.008915 | 0.612961 | 0.091335 | 0.937879 | 0.094349 | 0.099442 | 0.718937 | 0.416144 | 0.393436 | 0.863063 | 0.575691 | 0.001156 |
| ASGA0039480 | 8 | 1.1E+08 | 0.008974 | 0.662974 | 0.142132 | 0.058622 | 0.656208 | 0.719585 | 0.284096 | 0.600082 | 0.110728 | 0.147019 | 0.131795 | 0.109913 |
| H3GA0046653 | 16 | 56735969 | 0.008986 | 0.952893 | 0.097025 | 0.154322 | 0.09289 | 0.239599 | 0.033652 | 0.267251 | 0.868711 | 0.385805 | 0.609062 | 0.27084 |
| ALGA0077404 | 14 | 49579147 | 0.009041 | 0.42413 | 0.072195 | 0.783849 | 0.043132 | 0.171516 | 0.097347 | 0.03545 | 0.500862 | 0.917608 | 0.368008 | 0.930046 |
| ASGA0089637 | 1 | 8803333 | 0.009122 | 0.505753 | 0.132935 | 0.386305 | 0.029204 | 0.919749 | 0.550681 | 0.882661 | 0.323564 | 0.539807 | 0.77363 | 0.601587 |
| ASGA0090543 | 0 | 0 | 0.00913 | 0.389365 | 0.039569 | 0.067306 | 0.068367 | 0.818442 | 0.618902 | 0.950738 | 0.897893 | 0.531428 | 0.228373 | 0.090106 |
| H3GA0050708 | 18 | 33407094 | 0.009158 | 0.605141 | 0.267933 | 0.109678 | 0.359369 | 0.875604 | 0.212894 | 0.619883 | 0.598501 | 0.882116 | 0.599004 | 0.270006 |
| ALGA0103755 | 13 | 1.57E+08 | 0.009226 | 0.189837 | 0.229718 | 0.091797 | 0.065033 | 0.925226 | 0.08776 | 0.258646 | 0.706922 | 0.23822 | 0.937143 | 0.073604 |
| MARC0113027 | 0 | 0 | 0.009226 | 0.189837 | 0.229718 | 0.091797 | 0.065033 | 0.925226 | 0.08776 | 0.258646 | 0.706922 | 0.23822 | 0.937143 | 0.073604 |
| MARC0091257 | 5 | 13481761 | 0.009235 | 0.63806 | 0.13036 | 0.524274 | 0.822079 | 0.327357 | 0.373446 | 0.557015 | 0.614616 | 0.034067 | 0.027982 | 0.041973 |
| ALGA0105621 | 12 | 46545494 | 0.009276 | 0.310824 | 0.002446 | 0.663922 | 0.017583 | 0.068305 | 0.052071 | 0.001528 | 0.06645 | 0.802536 | 0.000885 | 0.04311 |
| ALGA0112318 | 12 | 60418556 | 0.009367 | 0.049025 | 0.02121 | 0.732456 | 0.579074 | 0.464769 | 0.14745 | 0.066849 | 0.555862 | 0.168714 | 0.749139 | 0.018465 |
| ALGA0077471 | 14 | 51547355 | 0.009382 | 0.417258 | 0.074751 | 0.753732 | 0.042235 | 0.204053 | 0.104056 | 0.036834 | 0.512103 | 0.849024 | 0.338653 | 0.939365 |
| DRGA0013821 | 14 | 51464087 | 0.009382 | 0.417258 | 0.074751 | 0.753732 | 0.042235 | 0.204053 | 0.104056 | 0.036834 | 0.512103 | 0.849024 | 0.338653 | 0.939365 |
| ASGA0059617 | 13 | 1.97E+08 | 0.009403 | 0.59646 | 0.33762 | 0.062739 | 0.251667 | 0.700343 | 0.078905 | 0.120583 | 0.882318 | 0.023513 | 0.593975 | 0.012457 |
| MARC0065078 | 12 | 42958511 | 0.009443 | 0.347459 | 0.017802 | 0.421021 | 0.582686 | 0.445484 | 0.83321 | 0.331485 | 0.700493 | 0.695638 | 0.008005 | 0.090208 |
| DRGA0011803 | 12 | 51300469 | 0.009443 | 0.460203 | 0.003511 | 0.006065 | 0.364793 | 0.084426 | 0.027614 | 0.215503 | 0.442151 | 0.175823 | 0.278072 | 0.038213 |
| ASGA0095947 | 5 | 83380222 | 0.009455 | 0.991229 | 0.04892 | 0.136451 | 0.089596 | 0.474641 | 0.633796 | 0.188516 | 0.405141 | 0.915854 | 0.20094 | 0.094567 |
| ASGA0089284 | 13 | 1.97E+08 | 0.009525 | 0.588337 | 0.342828 | 0.060129 | 0.273152 | 0.688628 | 0.093099 | 0.13344 | 0.896147 | 0.026555 | 0.625511 | 0.013382 |
| DRGA0009379 | 9 | 62097098 | 0.009529 | 0.686999 | 0.007953 | 0.000596 | 0.029025 | 0.272839 | 0.440309 | 0.997398 | 0.933133 | 0.177117 | 0.658817 | 0.02753 |
| H3GA0018914 | 6 | 1.3E+08 | 0.00958 | 0.669202 | 0.084081 | 0.226032 | 0.884918 | 0.98733 | 0.969619 | 0.942981 | 0.643825 | 0.590342 | 0.299013 | 0.790883 |
| ALGA0071815 | 13 | 1.27E+08 | 0.009625 | 0.473734 | 0.143071 | 0.11825 | 0.524804 | 0.854233 | 0.089585 | 0.596099 | 0.530758 | 0.705591 | 0.921202 | 0.024609 |
| H3GA0025242 | 8 | 1.09E+08 | 0.009646 | 0.727303 | 0.143932 | 0.380139 | 0.763933 | 0.835415 | 0.159762 | 0.811679 | 0.650046 | 0.32512 | 0.289655 | 0.062681 |
| DIAS0000878 | 5 | 67981416 | 0.009646 | 0.188971 | 0.594518 | 0.692808 | 0.070945 | 0.243051 | 0.959256 | 0.118426 | 0.429544 | 0.555781 | 0.221867 | 0.124351 |
| MARC0087675 | 2 | 1.28E+08 | 0.009701 | 0.406989 | 0.038038 | 0.558167 | 0.063809 | 0.049987 | 0.697298 | 0.18794 | 0.020243 | 0.81866 | 0.235473 | 0.624093 |
| MARC0093869 | 12 | 57219759 | 0.009706 | 0.827412 | 2.34E-05 | 0.417188 | 0.143081 | 0.116903 | 0.077982 | 0.002622 | 0.019911 | 0.955288 | 0.000108 | 0.002723 |
| ASGA0097955 | 1 | 11234129 | 0.009715 | 0.049171 | 0.01209 | 0.255306 | 0.640533 | 0.284618 | 0.641765 | 0.318683 | 0.528961 | 0.459238 | 0.918373 | 0.032858 |
| MARC0052559 | 8 | 15012662 | 0.009726 | 0.63848 | 0.262122 | 0.010504 | 0.300986 | 0.892998 | 0.333442 | 0.738632 | 0.901772 | 0.463377 | 0.254684 | 0.043785 |
| MARC0052560 | 8 | 15012713 | 0.009726 | 0.63848 | 0.262122 | 0.010504 | 0.300986 | 0.892998 | 0.333442 | 0.738632 | 0.901772 | 0.463377 | 0.254684 | 0.043785 |
| ALGA0058020 | 10 | 31842508 | 0.009758 | 0.693174 | 0.072376 | 0.363776 | 0.087743 | 0.974542 | 0.236467 | 0.585501 | 0.339008 | 0.616272 | 0.437471 | 0.265617 |
| ASGA0054449 | 12 | 40297843 | 0.009759 | 0.703141 | 0.047928 | 0.845698 | 0.019137 | 0.041029 | 0.192032 | 0.002693 | 0.098947 | 0.479494 | 0.011043 | 0.114655 |
| ALGA0039804 | 7 | 29987393 | 0.009794 | 0.553349 | 0.083932 | 0.003377 | 0.551658 | 0.182346 | 0.023556 | 0.819104 | 0.451403 | 0.618946 | 0.577302 | 0.381178 |
| ASGA0096753 | 13 | 1.96E+08 | 0.009795 | 0.377441 | 0.139532 | 0.73062 | 0.208122 | 0.846617 | 0.145893 | 0.018215 | 0.376469 | 0.375721 | 0.720387 | 0.096714 |
| ALGA0067072 | 12 | 57831831 | 0.009814 | 0.738597 | 3.11E-06 | 0.468669 | 0.083894 | 0.08838 | 0.023406 | 0.002792 | 0.013541 | 0.957048 | 1.06E-05 | 0.001112 |
| ALGA0106730 | 13 | 1.98E+08 | 0.009865 | 0.739445 | 0.099037 | 0.083896 | 0.102261 | 0.588806 | 0.037641 | 0.109175 | 0.492006 | 0.064664 | 0.414596 | 0.076213 |
| ALGA0114714 | 12 | 41895461 | 0.009965 | 0.87261 | 0.022919 | 0.890837 | 0.031994 | 0.026669 | 0.142264 | 0.002824 | 0.159959 | 0.394504 | 0.005435 | 0.099623 |
| ALGA0106919 | 0 | 0 | 0.009966 | 0.484575 | 0.020398 | 0.15077 | 0.11276 | 0.625523 | 0.848542 | 0.986998 | 0.305678 | 0.869696 | 0.193382 | 0.165485 |
| ALGA0119636 | 12 | 51487891 | 0.010187 | 0.393682 | 0.00386 | 0.009717 | 0.400343 | 0.086266 | 0.032703 | 0.242592 | 0.452005 | 0.200225 | 0.281697 | 0.039154 |
| MARC0048650 | 14 | 49485578 | 0.010222 | 0.479447 | 0.084869 | 0.725818 | 0.031117 | 0.127849 | 0.125621 | 0.035705 | 0.424382 | 0.973228 | 0.374119 | 0.945786 |
| H3GA0008378 | 2 | 1.58E+08 | 0.010237 | 0.403228 | 0.086867 | 0.198497 | 0.884917 | 0.658167 | 0.592513 | 0.379086 | 0.862389 | 0.170279 | 0.008981 | 0.184241 |
| H3GA0044706 | 15 | 95279515 | 0.010276 | 0.621854 | 0.038212 | 0.127633 | 0.12471 | 0.412093 | 0.105756 | 0.124484 | 0.679966 | 0.265621 | 0.861838 | 0.336791 |
| ASGA0075303 | 17 | 12690963 | 0.010289 | 0.865695 | 0.125004 | 0.175353 | 0.139857 | 0.84404 | 0.174949 | 0.395704 | 0.730508 | 0.696145 | 0.84953 | 0.083864 |
| ASGA0055256 | 12 | 58105269 | 0.010289 | 0.010795 | 0.000166 | 0.809836 | 0.05119 | 0.062578 | 0.061706 | 0.003574 | 0.022266 | 0.693913 | 0.002767 | 0.000851 |
| ALGA0030701 | 5 | 14102836 | 0.010307 | 0.527656 | 0.192087 | 0.594911 | 0.952764 | 0.137312 | 0.349187 | 0.651351 | 0.371855 | 0.042064 | 0.021484 | 0.132644 |
| ALGA0108452 | 12 | 48842443 | 0.010312 | 0.910288 | 0.002586 | 0.102127 | 0.018226 | 0.849942 | 0.85656 | 0.014151 | 0.967545 | 0.214856 | 0.011602 | 0.054532 |
| ASGA0082284 | 12 | 61949270 | 0.010339 | 0.088357 | 0.001903 | 0.545849 | 0.006459 | 0.356728 | 0.701322 | 4.96E-05 | 0.084232 | 0.203098 | 0.200479 | 0.000699 |
| H3GA0040656 | 14 | 66437563 | 0.010352 | 0.800043 | 0.024624 | 0.566861 | 0.023083 | 0.320899 | 0.676042 | 0.195582 | 0.64405 | 0.778764 | 0.105734 | 0.525739 |
| H3GA0046642 | 16 | 55928777 | 0.010429 | 0.967813 | 0.072118 | 0.256743 | 0.213697 | 0.336947 | 0.044055 | 0.358128 | 0.852397 | 0.360443 | 0.702081 | 0.263775 |
| M1GA0021598 | 17 | 13184778 | 0.010502 | 0.494173 | 0.092138 | 0.5215 | 0.023916 | 0.960662 | 0.487897 | 0.633154 | 0.040316 | 0.804242 | 0.700343 | 0.431296 |
| MARC0051357 | 8 | 1.09E+08 | 0.01056 | 0.633915 | 0.153886 | 0.16593 | 0.301057 | 0.704298 | 0.101494 | 0.272228 | 0.977858 | 0.804057 | 0.829167 | 0.018906 |
| H3GA0007455 | 2 | 1.22E+08 | 0.010571 | 0.197479 | 0.019383 | 0.975876 | 0.053815 | 0.328951 | 0.724487 | 0.517306 | 0.171416 | 0.817815 | 0.327722 | 0.129914 |
| ALGA0046619 | 8 | 16515963 | 0.010588 | 0.543224 | 0.314373 | 0.075898 | 0.280091 | 0.170167 | 0.94935 | 0.520291 | 0.495681 | 0.5371 | 0.634682 | 0.035368 |
| H3GA0040146 | 14 | 51760840 | 0.010603 | 0.472048 | 0.087795 | 0.696355 | 0.030433 | 0.154222 | 0.13385 | 0.037101 | 0.434691 | 0.903893 | 0.344382 | 0.955142 |
| ASGA0099275 | 0 | 0 | 0.010607 | 0.141194 | 0.211745 | 0.015413 | 0.010508 | 0.941919 | 0.917072 | 0.068542 | 0.250325 | 0.90945 | 0.102525 | 0.202879 |
| ALGA0085375 | 15 | 57507804 | 0.010657 | 0.666381 | 0.10115 | 0.001479 | 0.144524 | 0.152342 | 0.054862 | 0.683174 | 0.320336 | 0.445631 | 0.943356 | 0.308585 |
| ALGA0085405 | 15 | 58085034 | 0.010657 | 0.666381 | 0.10115 | 0.001479 | 0.144524 | 0.152342 | 0.054862 | 0.683174 | 0.320336 | 0.445631 | 0.943356 | 0.308585 |
| H3GA0046170 | 16 | 20589937 | 0.010708 | 0.236285 | 0.041584 | 0.133657 | 0.109985 | 0.772911 | 0.256511 | 0.597805 | 0.715703 | 0.093265 | 0.032187 | 0.475014 |
| ASGA0090485 | 4 | 16070229 | 0.010731 | 0.59958 | 0.196888 | 0.449939 | 0.025079 | 0.188147 | 0.692289 | 0.340131 | 0.016628 | 0.91664 | 0.518175 | 0.284351 |
| ASGA0093554 | 4 | 16069475 | 0.010731 | 0.59958 | 0.196888 | 0.449939 | 0.025079 | 0.188147 | 0.692289 | 0.340131 | 0.016628 | 0.91664 | 0.518175 | 0.284351 |
| MARC0010384 | 9 | 72264961 | 0.010743 | 0.85098 | 0.018715 | 0.000353 | 0.1237 | 0.189274 | 0.184726 | 0.931083 | 0.493135 | 0.190841 | 0.913081 | 0.010273 |
| ALGA0081364 | 14 | 1.27E+08 | 0.010756 | 0.155337 | 0.066154 | 0.480878 | 0.187582 | 0.916 | 0.624797 | 0.436573 | 0.162577 | 0.478439 | 0.297927 | 0.064888 |
| MARC0068513 | 6 | 91682009 | 0.010774 | 0.417029 | 0.045935 | 0.067045 | 0.062642 | 0.959517 | 0.67546 | 0.937733 | 0.776321 | 0.539856 | 0.229295 | 0.150563 |
| ALGA0113503 | 8 | 94427398 | 0.010783 | 0.632575 | 0.411857 | 0.303104 | 0.279723 | 0.740294 | 0.031344 | 0.25975 | 0.980295 | 0.675591 | 0.820755 | 0.052873 |
| DRGA0012065 | 0 | 0 | 0.010826 | 0.547905 | 0.05688 | 0.707677 | 0.339651 | 0.8853 | 0.087103 | 0.087657 | 0.71841 | 0.619123 | 0.849018 | 0.083298 |
| ALGA0109312 | 0 | 0 | 0.010834 | 0.474999 | 0.046986 | 0.070101 | 0.059525 | 0.957633 | 0.806817 | 0.995594 | 0.751168 | 0.546863 | 0.22501 | 0.148622 |
| ALGA0074984 | 14 | 10254612 | 0.010851 | 0.797311 | 0.073798 | 0.107812 | 0.531966 | 0.946169 | 0.994556 | 0.525034 | 0.758089 | 0.486448 | 0.287871 | 0.415224 |
| ASGA0027977 | 6 | 28284785 | 0.010933 | 0.18043 | 0.032795 | 0.046384 | 0.015662 | 0.546305 | 0.864315 | 0.371794 | 0.52859 | 0.180231 | 0.985452 | 0.257998 |
| ALGA0067099 | 12 | 57950908 | 0.010973 | 0.741077 | 1.39E-06 | 0.307656 | 0.251275 | 0.375189 | 0.053389 | 0.003773 | 0.035577 | 0.62533 | 8.25E-06 | 0.000656 |
| ASGA0054611 | 12 | 43330452 | 0.011002 | 0.306912 | 0.017867 | 0.518668 | 0.679865 | 0.491355 | 0.782557 | 0.449903 | 0.850121 | 0.62757 | 0.007416 | 0.118438 |
| ALGA0077977 | 14 | 62467689 | 0.011015 | 0.994991 | 0.220949 | 0.776196 | 0.029807 | 0.405173 | 0.736715 | 0.567715 | 0.277401 | 0.393371 | 0.548128 | 0.01068 |
| MARC0017865 | 14 | 62514781 | 0.011015 | 0.994991 | 0.220949 | 0.776196 | 0.029807 | 0.405173 | 0.736715 | 0.567715 | 0.277401 | 0.393371 | 0.548128 | 0.01068 |
| DRGA0006079 | 5 | 84048852 | 0.011041 | 0.967385 | 0.061185 | 0.097457 | 0.085944 | 0.675149 | 0.515065 | 0.19088 | 0.462352 | 0.978111 | 0.303778 | 0.079018 |
| H3GA0044704 | 15 | 95239799 | 0.011043 | 0.602349 | 0.038884 | 0.136556 | 0.126804 | 0.417455 | 0.108709 | 0.124392 | 0.659957 | 0.26772 | 0.847446 | 0.349041 |
| ASGA0054390 | 12 | 37778827 | 0.011048 | 0.602662 | 0.211793 | 0.615715 | 0.002786 | 0.072502 | 0.856511 | 0.000538 | 0.020507 | 0.456182 | 0.615076 | 0.086052 |
| ASGA0101872 | 8 | 1.09E+08 | 0.011071 | 0.643146 | 0.156141 | 0.156908 | 0.297316 | 0.700268 | 0.109644 | 0.28402 | 0.944417 | 0.741689 | 0.767075 | 0.021141 |
| ASGA0076016 | 17 | 31265855 | 0.011073 | 0.906772 | 0.008181 | 0.285048 | 0.090735 | 0.977327 | 0.644506 | 0.29778 | 0.532241 | 0.407561 | 0.370792 | 0.00619 |
| H3GA0040441 | 14 | 62131617 | 0.011095 | 0.882477 | 0.181127 | 0.782872 | 0.034625 | 0.363434 | 0.734671 | 0.573372 | 0.248736 | 0.409752 | 0.608085 | 0.010018 |
| DRGA0012199 | 13 | 22086155 | 0.011113 | 0.232856 | 0.046724 | 0.73556 | 0.349298 | 0.599649 | 0.280587 | 0.181101 | 0.86051 | 0.795526 | 0.69838 | 0.009628 |
| MARC0030345 | 12 | 58934290 | 0.011156 | 0.819271 | 1.49E-06 | 0.153671 | 0.150039 | 0.332066 | 0.059175 | 0.002767 | 0.03762 | 0.668872 | 5.42E-06 | 0.001849 |
| ASGA0091031 | 13 | 1.97E+08 | 0.011199 | 0.923567 | 0.091544 | 0.467683 | 0.440589 | 0.636905 | 0.042232 | 0.016351 | 0.497234 | 0.101818 | 0.603471 | 0.061319 |
| ALGA0066969 | 12 | 55602201 | 0.011352 | 0.092461 | 0.015122 | 0.96735 | 0.129285 | 0.347455 | 0.043974 | 0.025776 | 0.025887 | 0.412552 | 0.029068 | 0.011595 |
| ASGA0102070 | 6 | 76470683 | 0.011355 | 0.112652 | 0.240565 | 0.027447 | 0.011578 | 0.98897 | 0.989625 | 0.075572 | 0.216532 | 0.91703 | 0.109939 | 0.18756 |
| ALGA0069653 | 13 | 39946118 | 0.011356 | 0.499203 | 0.022262 | 0.715559 | 0.927666 | 0.936784 | 0.452942 | 0.465704 | 0.999853 | 0.509944 | 0.56499 | 0.0018 |
| H3GA0053692 | 12 | 45921271 | 0.011368 | 0.822711 | 0.003023 | 0.431525 | 0.040555 | 0.175786 | 0.103041 | 0.002868 | 0.099234 | 0.220955 | 0.004709 | 0.017896 |
| ALGA0116426 | 6 | 1.26E+08 | 0.011375 | 0.480862 | 0.02209 | 0.164342 | 0.117177 | 0.632485 | 0.872472 | 0.970133 | 0.307752 | 0.861122 | 0.214948 | 0.176524 |
| ASGA0087376 | 6 | 1.38E+08 | 0.011375 | 0.596943 | 0.186342 | 0.054018 | 0.625732 | 0.24124 | 0.113097 | 0.745425 | 0.897492 | 0.895308 | 0.828941 | 0.415451 |
| ALGA0112297 | 12 | 31679376 | 0.011397 | 0.637012 | 0.472595 | 0.61951 | 0.001726 | 0.045958 | 0.632593 | 0.001078 | 0.04771 | 0.289176 | 0.531922 | 0.06737 |
| ASGA0053987 | 12 | 31626704 | 0.011397 | 0.637012 | 0.472595 | 0.61951 | 0.001726 | 0.045958 | 0.632593 | 0.001078 | 0.04771 | 0.289176 | 0.531922 | 0.06737 |
| M1GA0002263 | 2 | 3024804 | 0.011403 | 0.432187 | 0.047435 | 0.310369 | 0.98178 | 0.306494 | 0.391357 | 0.488688 | 0.265374 | 0.369654 | 0.958651 | 0.069226 |
| M1GA0002265 | 2 | 3037491 | 0.011403 | 0.432187 | 0.047435 | 0.310369 | 0.98178 | 0.306494 | 0.391357 | 0.488688 | 0.265374 | 0.369654 | 0.958651 | 0.069226 |
| MARC0001281 | 6 | 66571073 | 0.011408 | 0.0738 | 0.019982 | 0.190589 | 0.028286 | 0.24729 | 0.866193 | 0.008607 | 0.06193 | 0.391798 | 0.178185 | 0.115732 |
| ASGA0024585 | 5 | 13502602 | 0.011408 | 0.666705 | 0.185765 | 0.568881 | 0.952798 | 0.142226 | 0.295904 | 0.612166 | 0.452589 | 0.023168 | 0.015449 | 0.127726 |
| ASGA0102491 | 2 | 31093481 | 0.011456 | 0.62814 | 0.055548 | 0.255903 | 0.666667 | 0.712635 | 0.751264 | 0.877679 | 0.949549 | 0.770767 | 0.624802 | 0.177451 |
| M1GA0017055 | 12 | 57032666 | 0.01152 | 0.101877 | 0.047627 | 0.972143 | 0.057903 | 0.06263 | 0.559117 | 0.04545 | 0.300999 | 0.690993 | 0.013327 | 0.036709 |
| MARC0093812 | 4 | 22317715 | 0.011545 | 0.167924 | 0.161255 | 0.015582 | 0.504697 | 0.338903 | 0.684212 | 0.82172 | 0.971951 | 0.522357 | 0.098502 | 0.341543 |
| H3GA0013026 | 4 | 81527282 | 0.011547 | 0.917222 | 0.045614 | 0.037149 | 0.343908 | 0.551752 | 0.511944 | 0.242465 | 0.420659 | 0.417833 | 0.043181 | 0.879043 |
| ALGA0039826 | 7 | 30162032 | 0.011552 | 0.854613 | 0.046054 | 0.112566 | 0.328083 | 0.951351 | 0.199097 | 0.78095 | 0.193634 | 0.526214 | 0.196839 | 0.039301 |
| ASGA0054455 | 12 | 41107246 | 0.011562 | 0.526909 | 0.054562 | 0.972008 | 0.011615 | 0.017429 | 0.156709 | 0.000855 | 0.131027 | 0.42904 | 0.013555 | 0.095264 |
| ASGA0048431 | 10 | 61210473 | 0.011586 | 0.552651 | 0.118179 | 0.056027 | 0.265479 | 0.18725 | 0.193727 | 0.627122 | 0.492732 | 0.269973 | 0.520187 | 0.265968 |
| ALGA0073427 | 13 | 1.99E+08 | 0.011614 | 0.38763 | 0.013549 | 0.148668 | 0.247489 | 0.383048 | 0.05837 | 0.006726 | 0.512041 | 0.061328 | 0.335923 | 0.012411 |
| ALGA0069649 | 13 | 39657443 | 0.011617 | 0.392529 | 0.025501 | 0.634973 | 0.906846 | 0.851779 | 0.4246 | 0.644887 | 0.94989 | 0.429824 | 0.653559 | 0.000721 |
| ASGA0058822 | 13 | 1.39E+08 | 0.011652 | 0.051935 | 0.240785 | 0.001804 | 0.110745 | 0.164951 | 0.097441 | 0.94765 | 0.515968 | 0.034604 | 0.591091 | 0.014642 |
| ALGA0048765 | 8 | 1.04E+08 | 0.011667 | 0.706636 | 0.124902 | 0.052721 | 0.407362 | 0.566594 | 0.136049 | 0.181062 | 0.273613 | 0.607368 | 0.167065 | 0.476194 |
| ALGA0048774 | 0 | 0 | 0.011667 | 0.706636 | 0.124902 | 0.052721 | 0.407362 | 0.566594 | 0.136049 | 0.181062 | 0.273613 | 0.607368 | 0.167065 | 0.476194 |
| ALGA0010673 | 1 | 3.03E+08 | 0.011687 | 0.913564 | 0.185841 | 0.421473 | 0.147683 | 0.839413 | 0.162382 | 0.236565 | 0.634754 | 0.854542 | 0.404497 | 0.260375 |
| DRGA0017466 | 12 | 39226571 | 0.011697 | 0.848006 | 0.08569 | 0.685612 | 0.018348 | 0.046146 | 0.324097 | 0.001061 | 0.102477 | 0.510717 | 0.012845 | 0.175534 |
| MARC0099264 | 6 | 92685161 | 0.011746 | 0.391496 | 0.066654 | 0.05052 | 0.065248 | 0.747724 | 0.472871 | 0.414351 | 0.881928 | 0.652783 | 0.311487 | 0.107551 |
| ASGA0098453 | 4 | 16081245 | 0.011765 | 0.590455 | 0.209828 | 0.487343 | 0.026511 | 0.180244 | 0.671097 | 0.350854 | 0.016233 | 0.884481 | 0.532806 | 0.297496 |
| H3GA0023764 | 7 | 1.29E+08 | 0.011776 | 0.409864 | 0.078724 | 0.611765 | 0.017719 | 0.996814 | 0.249493 | 0.067707 | 0.616924 | 0.693604 | 0.983661 | 0.533347 |
| H3GA0040186 | 14 | 53037996 | 0.011779 | 0.938727 | 0.161567 | 0.945894 | 0.003329 | 0.308268 | 0.054412 | 0.000623 | 0.905068 | 0.395847 | 0.228273 | 0.627992 |
| ASGA0079477 | 18 | 33106672 | 0.011805 | 0.650984 | 0.26292 | 0.125802 | 0.470564 | 0.650199 | 0.253926 | 0.661479 | 0.573632 | 0.742853 | 0.661312 | 0.269012 |
| ASGA0050198 | 11 | 21456138 | 0.011816 | 0.403496 | 0.176651 | 0.021018 | 0.128869 | 0.930234 | 0.533084 | 0.173742 | 0.709748 | 0.898401 | 0.067713 | 0.573647 |
| ALGA0114110 | 8 | 87012660 | 0.011817 | 0.73818 | 0.028159 | 0.077638 | 0.387765 | 0.660857 | 0.78422 | 0.425217 | 0.858414 | 0.05621 | 0.017473 | 0.164168 |
| ASGA0054862 | 12 | 47862623 | 0.011819 | 0.966371 | 0.002306 | 0.080904 | 0.016961 | 0.953821 | 0.665834 | 0.016504 | 0.894659 | 0.289249 | 0.013379 | 0.060773 |
| ASGA0066341 | 14 | 1.28E+08 | 0.011829 | 0.107699 | 0.098813 | 0.50482 | 0.190527 | 0.97644 | 0.768272 | 0.416659 | 0.247227 | 0.503483 | 0.291701 | 0.088414 |
| ALGA0086269 | 15 | 93647963 | 0.011841 | 0.579005 | 0.128461 | 0.156491 | 0.242216 | 0.539181 | 0.285009 | 0.570822 | 0.822199 | 0.432017 | 0.737534 | 0.144913 |
| ASGA0059611 | 13 | 1.97E+08 | 0.011874 | 0.324477 | 0.063115 | 0.145319 | 0.631291 | 0.54736 | 0.153316 | 0.026495 | 0.55088 | 0.032064 | 0.3329 | 0.005956 |
| MARC0034934 | 9 | 1.15E+08 | 0.011883 | 0.233848 | 0.032428 | 0.087949 | 0.004683 | 0.835711 | 0.941448 | 0.718558 | 0.185942 | 0.683258 | 0.287368 | 0.286673 |
| MARC0015264 | 12 | 60301903 | 0.011895 | 0.229604 | 0.000224 | 0.18573 | 0.859735 | 0.692659 | 0.576274 | 0.088087 | 0.958641 | 0.453457 | 0.027074 | 0.02966 |
| ALGA0124063 | 12 | 41841267 | 0.011908 | 0.907482 | 0.036986 | 0.963953 | 0.016132 | 0.03087 | 0.216283 | 0.001134 | 0.114126 | 0.469473 | 0.005069 | 0.157716 |
| MARC0012805 | 6 | 1.1E+08 | 0.011966 | 0.630045 | 0.045736 | 0.843101 | 0.561469 | 0.246531 | 0.85153 | 0.756907 | 0.39173 | 0.341653 | 0.268353 | 0.290576 |
| ALGA0078296 | 14 | 67076542 | 0.011976 | 0.168761 | 0.02974 | 0.928912 | 0.100812 | 0.266049 | 0.740474 | 0.524637 | 0.23006 | 0.891152 | 0.338741 | 0.616781 |
| MARC0022741 | 12 | 57191332 | 0.012008 | 0.203894 | 0.055563 | 0.638794 | 0.035512 | 0.081024 | 0.092326 | 0.000627 | 0.034317 | 0.580052 | 0.126419 | 0.018433 |
| ALGA0123808 | 6 | 1.26E+08 | 0.012013 | 0.462966 | 0.017207 | 0.131772 | 0.146539 | 0.487173 | 0.886819 | 0.959259 | 0.319182 | 0.952351 | 0.245237 | 0.137308 |
| ALGA0033200 | 5 | 85743522 | 0.012023 | 0.520856 | 0.032895 | 0.051374 | 0.232133 | 0.790923 | 0.332567 | 0.320919 | 0.996413 | 0.672647 | 0.331429 | 0.18289 |
| CASI0007903 | 15 | 1.25E+08 | 0.012058 | 0.550192 | 0.040934 | 0.034829 | 0.152401 | 0.730651 | 0.585668 | 0.100836 | 0.447335 | 0.161553 | 0.250391 | 0.7942 |
| ALGA0097816 | 18 | 32645078 | 0.012085 | 0.645916 | 0.295901 | 0.148418 | 0.484406 | 0.716331 | 0.299684 | 0.720134 | 0.640565 | 0.89035 | 0.590325 | 0.291976 |
| ALGA0097819 | 18 | 32774299 | 0.012085 | 0.645916 | 0.295901 | 0.148418 | 0.484406 | 0.716331 | 0.299684 | 0.720134 | 0.640565 | 0.89035 | 0.590325 | 0.291976 |
| ALGA0073323 | 13 | 1.96E+08 | 0.012093 | 0.356755 | 0.120269 | 0.061913 | 0.195485 | 0.271567 | 0.013439 | 0.006489 | 0.652965 | 0.033641 | 0.246325 | 0.045034 |
| ALGA0036982 | 6 | 1.29E+08 | 0.012143 | 0.415314 | 0.059413 | 0.230849 | 0.075492 | 0.583001 | 0.944189 | 0.298737 | 0.208737 | 0.170481 | 0.13717 | 0.370662 |
| MARC0060671 | 0 | 0 | 0.012181 | 0.135852 | 0.227145 | 0.017495 | 0.011725 | 0.956789 | 0.999019 | 0.076104 | 0.224281 | 0.957747 | 0.093325 | 0.204748 |
| H3GA0040188 | 14 | 53050597 | 0.012198 | 0.982387 | 0.152535 | 0.931377 | 0.003322 | 0.283519 | 0.059655 | 0.000567 | 0.855393 | 0.477243 | 0.250528 | 0.598888 |
| ALGA0023588 | 4 | 16552732 | 0.012231 | 0.999154 | 0.019262 | 0.04526 | 0.82382 | 0.613884 | 0.975771 | 0.761607 | 0.307966 | 0.988373 | 0.326357 | 0.539064 |
| ASGA0043673 | 9 | 75310438 | 0.012261 | 0.333221 | 0.012273 | 0.0396 | 0.743613 | 0.305303 | 0.400564 | 0.656776 | 0.318524 | 0.138213 | 0.662901 | 0.008048 |
| ALGA0112803 | 0 | 0 | 0.012276 | 0.381435 | 0.004926 | 0.052534 | 0.151589 | 0.220018 | 0.515295 | 0.057513 | 0.360688 | 0.136606 | 0.46564 | 0.023853 |
| ASGA0002303 | 1 | 39468495 | 0.012284 | 0.425866 | 0.03437 | 0.800294 | 0.186665 | 0.663164 | 0.648615 | 0.119742 | 0.508069 | 0.636964 | 0.864148 | 0.05856 |
| ALGA0063853 | 11 | 80996526 | 0.012296 | 0.696324 | 0.013062 | 0.10937 | 0.313858 | 0.812502 | 0.621236 | 0.668342 | 0.689546 | 0.839198 | 0.284316 | 0.396207 |
| ASGA0065151 | 14 | 97219551 | 0.0123 | 0.054571 | 0.068847 | 0.121555 | 0.001089 | 0.759063 | 0.682627 | 0.325951 | 0.258172 | 0.141428 | 0.624054 | 0.206483 |
| ASGA0068142 | 14 | 1.51E+08 | 0.012309 | 0.899348 | 0.005032 | 0.0988 | 0.12679 | 0.932845 | 0.430286 | 0.963493 | 0.631644 | 0.61327 | 0.690331 | 0.415273 |
| DRGA0007943 | 7 | 90209262 | 0.012326 | 0.054295 | 0.017907 | 0.194166 | 0.065507 | 0.291366 | 0.592345 | 0.142888 | 0.231456 | 0.352299 | 0.258593 | 0.008267 |
| ASGA0105466 | 6 | 71689642 | 0.012361 | 0.346395 | 0.009942 | 0.111429 | 0.119213 | 0.483071 | 0.59378 | 0.031669 | 0.561857 | 0.996051 | 0.260649 | 0.178418 |
| ALGA0049048 | 8 | 1.13E+08 | 0.012377 | 0.174386 | 0.021766 | 0.066602 | 0.102792 | 0.46474 | 0.855665 | 0.405087 | 0.633526 | 0.247993 | 0.072549 | 0.164118 |
| ALGA0043059 | 7 | 90175363 | 0.01239 | 0.055435 | 0.021975 | 0.233004 | 0.05796 | 0.209022 | 0.807216 | 0.161072 | 0.16281 | 0.39197 | 0.305975 | 0.010025 |
| ALGA0034758 | 6 | 19523664 | 0.012393 | 0.91029 | 0.047095 | 0.567218 | 0.828689 | 0.929606 | 0.034434 | 0.229658 | 0.555852 | 0.326451 | 0.629944 | 0.252752 |
| ALGA0117579 | 3 | 1.44E+08 | 0.012397 | 0.212571 | 0.254295 | 0.050003 | 0.169424 | 0.941949 | 0.246985 | 0.07261 | 0.736397 | 0.174221 | 0.982147 | 0.214229 |
| ALGA0066702 | 12 | 47828412 | 0.012429 | 0.9842 | 0.002077 | 0.084721 | 0.017087 | 0.90735 | 0.701163 | 0.015433 | 0.950881 | 0.222215 | 0.01607 | 0.054847 |
| ASGA0028671 | 6 | 74814725 | 0.012456 | 0.12848 | 0.274927 | 0.011763 | 0.010661 | 0.889589 | 0.719926 | 0.063909 | 0.334205 | 0.984452 | 0.145579 | 0.130234 |
| ASGA0096690 | 12 | 58429105 | 0.012461 | 0.014264 | 0.001213 | 0.807857 | 0.053569 | 0.014294 | 0.100878 | 0.002695 | 0.062399 | 0.995954 | 0.002694 | 0.004463 |
| MARC0028970 | 6 | 74136089 | 0.012464 | 0.100842 | 0.249101 | 0.01028 | 0.008887 | 0.886052 | 0.719747 | 0.080037 | 0.348859 | 0.998453 | 0.170755 | 0.122583 |
| H3GA0022758 | 12 | 59449674 | 0.01248 | 0.871693 | 9.63E-05 | 0.553707 | 0.033702 | 0.012528 | 0.029125 | 0.004215 | 0.039233 | 0.829279 | 7.09E-05 | 0.024302 |
| ALGA0015690 | 2 | 1.3E+08 | 0.012506 | 0.070452 | 0.084142 | 0.349015 | 0.096094 | 0.979977 | 0.089254 | 0.396207 | 0.830922 | 0.950326 | 0.866954 | 0.197697 |
| ALGA0073927 | 13 | 2.12E+08 | 0.012547 | 0.724829 | 0.096756 | 0.568697 | 0.333037 | 0.544424 | 0.793754 | 0.953026 | 0.718792 | 0.019401 | 0.011389 | 0.221479 |
| DIAS0002526 | 6 | 77759337 | 0.01256 | 0.130349 | 0.298002 | 0.077988 | 0.013018 | 0.840067 | 0.843759 | 0.082925 | 0.154154 | 0.736977 | 0.074635 | 0.264825 |
| ALGA0049179 | 8 | 1.18E+08 | 0.012562 | 0.59331 | 0.057189 | 0.086661 | 0.568724 | 0.203819 | 0.063743 | 0.742012 | 0.267606 | 0.949483 | 0.508416 | 0.114626 |
| ASGA0064360 | 14 | 79206815 | 0.012597 | 0.453037 | 0.177226 | 0.44156 | 0.000902 | 0.315922 | 0.44221 | 0.211034 | 0.46618 | 0.680201 | 0.99678 | 0.035169 |
| MARC0009546 | 12 | 58942845 | 0.012669 | 0.888296 | 1.07E-06 | 0.215303 | 0.172131 | 0.382518 | 0.044564 | 0.003066 | 0.033123 | 0.658043 | 8.62E-06 | 0.000941 |
| INRA0043947 | 14 | 49115640 | 0.01273 | 0.860827 | 0.152669 | 0.937865 | 0.014547 | 0.441754 | 0.529762 | 0.065176 | 0.472066 | 0.177457 | 0.385554 | 0.120349 |
| ALGA0119112 | 10 | 71078073 | 0.012803 | 0.416278 | 0.086819 | 0.241296 | 0.254038 | 0.58675 | 0.605196 | 0.9283 | 0.677823 | 0.785372 | 0.401176 | 0.796793 |
| ALGA0015281 | 2 | 1.22E+08 | 0.012813 | 0.902292 | 0.099496 | 0.880211 | 0.016849 | 0.052099 | 0.215684 | 0.622884 | 0.069921 | 0.193216 | 0.029837 | 0.571759 |
| MARC0008298 | 9 | 77710647 | 0.012817 | 0.664319 | 0.111176 | 0.318196 | 0.204242 | 0.17651 | 0.602845 | 0.680334 | 0.168656 | 0.776261 | 0.184762 | 0.104383 |
| ALGA0107852 | 12 | 62158019 | 0.012838 | 0.539127 | 0.005415 | 0.547557 | 0.111429 | 0.995256 | 0.750243 | 0.016156 | 0.319571 | 0.183269 | 0.652306 | 0.000608 |
| MARC0062541 | 12 | 62148072 | 0.012838 | 0.539127 | 0.005415 | 0.547557 | 0.111429 | 0.995256 | 0.750243 | 0.016156 | 0.319571 | 0.183269 | 0.652306 | 0.000608 |
| ALGA0119119 | 13 | 24416165 | 0.012888 | 0.80686 | 0.289924 | 0.247608 | 0.506393 | 0.891484 | 0.425126 | 0.963729 | 0.416557 | 0.16224 | 0.610092 | 0.404117 |
| ALGA0060441 | 11 | 4751774 | 0.012903 | 0.643844 | 0.254123 | 0.159558 | 0.632025 | 0.544629 | 0.596449 | 0.460229 | 0.778443 | 0.297924 | 0.054875 | 0.180021 |
| DRGA0008346 | 8 | 15886903 | 0.012905 | 0.688076 | 0.382446 | 0.06905 | 0.306646 | 0.16764 | 0.816557 | 0.477057 | 0.510344 | 0.550307 | 0.554456 | 0.10083 |
| ASGA0029619 | 6 | 1.3E+08 | 0.012916 | 0.471068 | 0.450679 | 0.271164 | 0.205802 | 0.521972 | 0.727965 | 0.510953 | 0.382065 | 0.514399 | 0.556729 | 0.785872 |
| H3GA0056765 | 6 | 1.37E+08 | 0.012927 | 0.338728 | 0.84887 | 0.076333 | 0.316512 | 0.48953 | 0.187555 | 0.137629 | 0.921306 | 0.580371 | 0.764215 | 0.527272 |
| MARC0089742 | 0 | 0 | 0.012969 | 0.091193 | 0.045908 | 0.712047 | 0.294347 | 0.653654 | 0.94532 | 0.718182 | 0.678892 | 0.819244 | 0.218818 | 0.078487 |
| MARC0009444 | 13 | 1.98E+08 | 0.012989 | 0.332405 | 0.037181 | 0.137445 | 0.579743 | 0.549048 | 0.220726 | 0.033545 | 0.509183 | 0.019436 | 0.623481 | 0.005 |
| DRGA0006536 | 6 | 19473300 | 0.012998 | 0.626927 | 0.288499 | 0.027191 | 0.763254 | 0.528631 | 0.203986 | 0.669815 | 0.371227 | 0.830617 | 0.858802 | 0.745951 |
| DIAS0004797 | 12 | 48514682 | 0.013001 | 0.894965 | 0.003431 | 0.112153 | 0.018895 | 0.869883 | 0.813475 | 0.018665 | 0.950973 | 0.217923 | 0.013875 | 0.071238 |
| H3GA0015257 | 5 | 3813901 | 0.013021 | 0.163891 | 0.08542 | 0.151462 | 0.730931 | 0.173917 | 0.166825 | 0.825333 | 0.341091 | 0.442415 | 0.127391 | 0.757193 |
| ALGA0077532 | 14 | 52942907 | 0.013022 | 0.999595 | 0.181196 | 0.896981 | 0.002289 | 0.252773 | 0.068561 | 0.000628 | 0.826267 | 0.363913 | 0.231978 | 0.639097 |
| ALGA0077535 | 14 | 52921125 | 0.013022 | 0.999595 | 0.181196 | 0.896981 | 0.002289 | 0.252773 | 0.068561 | 0.000628 | 0.826267 | 0.363913 | 0.231978 | 0.639097 |
| ASGA0063329 | 0 | 0 | 0.013022 | 0.999595 | 0.181196 | 0.896981 | 0.002289 | 0.252773 | 0.068561 | 0.000628 | 0.826267 | 0.363913 | 0.231978 | 0.639097 |
| H3GA0040193 | 14 | 53073486 | 0.013022 | 0.999595 | 0.181196 | 0.896981 | 0.002289 | 0.252773 | 0.068561 | 0.000628 | 0.826267 | 0.363913 | 0.231978 | 0.639097 |
| MARC0063524 | 14 | 52754051 | 0.013022 | 0.999595 | 0.181196 | 0.896981 | 0.002289 | 0.252773 | 0.068561 | 0.000628 | 0.826267 | 0.363913 | 0.231978 | 0.639097 |
| MARC0071472 | 14 | 18382899 | 0.013088 | 0.476558 | 0.204848 | 0.882486 | 0.297738 | 0.050781 | 0.471027 | 0.245718 | 0.086243 | 0.07713 | 0.400729 | 0.281988 |
| ASGA0087467 | 13 | 23805685 | 0.013096 | 0.8221 | 0.0584 | 0.095661 | 0.880074 | 0.58005 | 0.065479 | 0.907916 | 0.211836 | 0.459464 | 0.857608 | 0.234168 |
| DRGA0013350 | 13 | 1.97E+08 | 0.013106 | 0.353061 | 0.063666 | 0.144726 | 0.667807 | 0.478087 | 0.194069 | 0.033711 | 0.539612 | 0.027313 | 0.390126 | 0.004383 |
| INRA0041449 | 13 | 1.95E+08 | 0.013126 | 0.379406 | 0.099843 | 0.191592 | 0.584694 | 0.420838 | 0.033524 | 0.0072 | 0.845691 | 0.020475 | 0.283952 | 0.004173 |
| ALGA0071870 | 13 | 1.33E+08 | 0.01313 | 0.329785 | 0.523931 | 0.185824 | 0.124642 | 0.813713 | 0.054991 | 0.148315 | 0.869145 | 0.882923 | 0.909542 | 0.052853 |
| H3GA0042471 | 14 | 1.37E+08 | 0.013145 | 0.30101 | 0.060312 | 0.017008 | 0.864573 | 0.414733 | 0.369235 | 0.930238 | 0.92747 | 0.499242 | 0.35388 | 0.521324 |
| H3GA0056170 | 0 | 0 | 0.013179 | 0.472456 | 5.6E-07 | 0.185062 | 0.404965 | 0.347282 | 0.037596 | 0.036258 | 0.065912 | 0.449224 | 1.98E-05 | 0.000499 |
| MARC0085508 | 12 | 42868692 | 0.013181 | 0.246172 | 0.023885 | 0.75026 | 0.632698 | 0.336857 | 0.993787 | 0.721735 | 0.689443 | 0.675989 | 0.015186 | 0.083675 |
| ALGA0063821 | 11 | 80774774 | 0.01321 | 0.751477 | 0.033283 | 0.146918 | 0.4561 | 0.237484 | 0.090541 | 0.682799 | 0.946945 | 0.384825 | 0.493525 | 0.064658 |
| ALGA0001216 | 1 | 16618413 | 0.013235 | 0.090153 | 0.03485 | 0.126186 | 0.465475 | 0.153128 | 0.445705 | 0.888592 | 0.770445 | 0.804212 | 0.901216 | 0.043213 |
| MARC0046250 | 2 | 85779599 | 0.01326 | 0.053227 | 0.023773 | 0.866177 | 0.097048 | 0.223206 | 0.876617 | 0.634792 | 0.171306 | 0.546333 | 0.252997 | 0.228442 |
| ALGA0113970 | 13 | 23475283 | 0.013276 | 0.154601 | 0.060742 | 0.67353 | 0.574674 | 0.820761 | 0.30171 | 0.330262 | 0.772248 | 0.791149 | 0.796266 | 0.008812 |
| MARC0069889 | 13 | 1.49E+08 | 0.01334 | 0.526602 | 0.10994 | 0.001479 | 0.134628 | 0.254347 | 0.027244 | 0.465304 | 0.718481 | 0.175909 | 0.375958 | 0.08242 |
| ALGA0118344 | 12 | 42867238 | 0.013344 | 0.289602 | 0.027397 | 0.627925 | 0.568577 | 0.470819 | 0.789338 | 0.592384 | 0.582694 | 0.956408 | 0.024783 | 0.073202 |
| ASGA0098997 | 8 | 8367943 | 0.013353 | 0.652666 | 0.009641 | 0.165313 | 0.963138 | 0.641022 | 0.535309 | 0.542707 | 0.926831 | 0.762761 | 0.132677 | 0.017201 |
| H3GA0043199 | 14 | 1.48E+08 | 0.013379 | 0.959589 | 0.02738 | 0.148889 | 0.145011 | 0.861175 | 0.463589 | 0.942224 | 0.875453 | 0.190143 | 0.815966 | 0.549995 |
| ALGA0113811 | 10 | 70638252 | 0.01339 | 0.680555 | 0.071338 | 0.110709 | 0.41838 | 0.335797 | 0.325501 | 0.685509 | 0.56602 | 0.419876 | 0.362634 | 0.534941 |
| CASI0007637 | 18 | 32611054 | 0.013392 | 0.597699 | 0.282902 | 0.130967 | 0.480527 | 0.647685 | 0.278592 | 0.723021 | 0.601935 | 0.972832 | 0.571162 | 0.267314 |
| ASGA0093216 | 17 | 31163121 | 0.013394 | 0.811138 | 0.035497 | 0.964137 | 0.614681 | 0.999091 | 0.544105 | 0.752977 | 0.932827 | 0.9444 | 0.877853 | 0.020118 |
| H3GA0047920 | 17 | 13605158 | 0.013415 | 0.963512 | 0.040905 | 0.204598 | 0.246143 | 0.816267 | 0.79852 | 0.734385 | 0.239914 | 0.38814 | 0.142952 | 0.136415 |
| ALGA0028846 | 4 | 1.32E+08 | 0.013428 | 0.40595 | 0.03135 | 0.037147 | 0.575681 | 0.292672 | 0.826857 | 0.858616 | 0.858611 | 0.438927 | 0.73418 | 0.103407 |
| ASGA0097645 | 6 | 77180996 | 0.013468 | 0.195158 | 0.2201 | 0.027026 | 0.015396 | 0.883301 | 0.989483 | 0.109897 | 0.28908 | 0.87905 | 0.082811 | 0.250854 |
| MARC0092020 | 3 | 1.36E+08 | 0.013506 | 0.368244 | 0.065349 | 0.425443 | 0.697969 | 0.499369 | 0.8283 | 0.744138 | 0.950313 | 0.700916 | 0.303343 | 0.027767 |
| ALGA0087491 | 15 | 1.35E+08 | 0.013549 | 0.31697 | 0.030911 | 0.004588 | 0.169145 | 0.274339 | 0.15095 | 0.659605 | 0.707334 | 0.903218 | 0.4768 | 0.096285 |
| ASGA0064027 | 14 | 67035481 | 0.013568 | 0.770034 | 0.030958 | 0.549853 | 0.024598 | 0.336813 | 0.719281 | 0.182683 | 0.599594 | 0.990303 | 0.136656 | 0.532051 |
| ASGA0001096 | 1 | 14213367 | 0.013606 | 0.130183 | 0.003288 | 0.389886 | 0.134896 | 0.457564 | 0.6151 | 0.465906 | 0.454606 | 0.634047 | 0.958634 | 0.006764 |
| ALGA0049011 | 8 | 1.13E+08 | 0.01367 | 0.207163 | 0.015379 | 0.054122 | 0.084714 | 0.798308 | 0.743948 | 0.345264 | 0.572176 | 0.293418 | 0.065071 | 0.101115 |
| ALGA0047083 | 0 | 0 | 0.013681 | 0.46239 | 0.006541 | 0.005575 | 0.407417 | 0.027934 | 0.05009 | 0.415299 | 0.333616 | 0.664865 | 0.55096 | 0.145975 |
| ALGA0058821 | 10 | 48604454 | 0.013684 | 0.090067 | 0.644469 | 0.409745 | 0.016477 | 0.491501 | 0.750624 | 0.3718 | 0.254704 | 0.565449 | 0.869232 | 0.64134 |
| DRGA0014656 | 14 | 1.37E+08 | 0.013813 | 0.304592 | 0.0633 | 0.01279 | 0.838824 | 0.397917 | 0.390453 | 0.897779 | 0.899769 | 0.52545 | 0.334985 | 0.489795 |
| ALGA0115519 | 9 | 88276225 | 0.013813 | 0.54921 | 0.098548 | 0.009461 | 0.113998 | 0.6831 | 0.780726 | 0.66787 | 0.309058 | 0.476172 | 0.314809 | 0.094889 |
| ASGA0089445 | 0 | 0 | 0.013834 | 0.970038 | 0.349717 | 0.012046 | 0.122436 | 0.475236 | 0.085166 | 0.089162 | 0.959731 | 0.348238 | 0.891024 | 0.334717 |
| ASGA0079381 | 18 | 27560376 | 0.013859 | 0.53475 | 0.205986 | 0.110019 | 0.751147 | 0.598944 | 0.309484 | 0.978584 | 0.596566 | 0.783589 | 0.637064 | 0.576542 |
| ALGA0122915 | 0 | 0 | 0.013889 | 0.39908 | 0.098822 | 0.720081 | 0.832987 | 0.9459 | 0.595877 | 0.989361 | 0.862132 | 0.949079 | 0.151453 | 0.627697 |
| DIAS0001212 | 0 | 0 | 0.013889 | 0.39908 | 0.098822 | 0.720081 | 0.832987 | 0.9459 | 0.595877 | 0.989361 | 0.862132 | 0.949079 | 0.151453 | 0.627697 |
| ALGA0047251 | 8 | 30820808 | 0.013897 | 0.328752 | 0.033245 | 0.031385 | 0.112706 | 0.877369 | 0.957715 | 0.664881 | 0.157468 | 0.019968 | 0.078806 | 0.246134 |
| ALGA0071606 | 13 | 1.07E+08 | 0.013944 | 0.29043 | 0.086625 | 0.409686 | 0.650901 | 0.464711 | 0.344039 | 0.652841 | 0.922839 | 0.836904 | 0.483992 | 0.013086 |
| ASGA0087803 | 0 | 0 | 0.013945 | 0.27015 | 0.046571 | 0.173096 | 0.287587 | 0.676501 | 0.034336 | 0.744189 | 0.424897 | 0.74314 | 0.592745 | 0.409419 |
| ASGA0007897 | 1 | 3.04E+08 | 0.013956 | 0.587515 | 0.152368 | 0.058392 | 0.160224 | 0.416631 | 0.256705 | 0.410607 | 0.896191 | 0.81627 | 0.752946 | 0.540303 |
| H3GA0037266 | 13 | 1.38E+08 | 0.013965 | 0.492045 | 0.129622 | 0.179378 | 0.494771 | 0.929891 | 0.120294 | 0.245451 | 0.71405 | 0.975076 | 0.601271 | 0.031302 |
| DIAS0003067 | 9 | 71614113 | 0.013969 | 0.852925 | 0.032567 | 0.082544 | 0.550435 | 0.691252 | 0.935274 | 0.800219 | 0.827656 | 0.628041 | 0.331776 | 0.330398 |
| H3GA0053054 | 1 | 39433881 | 0.014112 | 0.439323 | 0.032811 | 0.742968 | 0.202712 | 0.607515 | 0.680731 | 0.115741 | 0.531887 | 0.42867 | 0.670299 | 0.060899 |
| ALGA0028850 | 4 | 1.32E+08 | 0.014182 | 0.399253 | 0.028768 | 0.039773 | 0.545929 | 0.303507 | 0.836543 | 0.821699 | 0.839128 | 0.475557 | 0.66745 | 0.116998 |
| H3GA0014475 | 4 | 1.31E+08 | 0.014182 | 0.399253 | 0.028768 | 0.039773 | 0.545929 | 0.303507 | 0.836543 | 0.821699 | 0.839128 | 0.475557 | 0.66745 | 0.116998 |
| H3GA0000571 | 1 | 8600391 | 0.014237 | 0.7008 | 0.329629 | 0.304297 | 0.029272 | 0.583709 | 0.98206 | 0.8718 | 0.23925 | 0.811199 | 0.574524 | 0.660195 |
| MARC0073399 | 13 | 5411978 | 0.014249 | 0.598317 | 0.261316 | 0.010723 | 0.638943 | 0.136043 | 0.056723 | 0.995236 | 0.061808 | 0.79594 | 0.722252 | 0.515585 |
| MARC0030249 | 15 | 57452214 | 0.014303 | 0.928281 | 0.11314 | 0.003932 | 0.10597 | 0.303419 | 0.093659 | 0.409838 | 0.542539 | 0.694729 | 0.996936 | 0.514357 |
| INRA0057755 | 8 | 1.09E+08 | 0.014304 | 0.73068 | 0.182259 | 0.185054 | 0.337103 | 0.729772 | 0.116115 | 0.309096 | 0.975688 | 0.842753 | 0.832787 | 0.027883 |
| MARC0020237 | 8 | 69070421 | 0.014313 | 0.167385 | 0.000924 | 0.01676 | 0.421226 | 0.26138 | 0.319543 | 0.730528 | 0.660961 | 0.80896 | 0.179043 | 0.22374 |
| ALGA0104212 | 12 | 46023473 | 0.014336 | 0.906644 | 0.003494 | 0.400862 | 0.033543 | 0.197571 | 0.1507 | 0.001409 | 0.188768 | 0.196201 | 0.007277 | 0.045956 |
| ALGA0030716 | 5 | 14322030 | 0.014339 | 0.605601 | 0.169788 | 0.71194 | 0.814032 | 0.214514 | 0.294853 | 0.523845 | 0.419908 | 0.06792 | 0.043178 | 0.041635 |
| ALGA0075834 | 14 | 19322091 | 0.014364 | 0.720506 | 0.030336 | 0.582806 | 0.115537 | 0.582464 | 0.658123 | 0.747224 | 0.225283 | 0.530174 | 0.188688 | 0.486571 |
| ALGA0071608 | 13 | 1.07E+08 | 0.014365 | 0.279535 | 0.083555 | 0.379001 | 0.665402 | 0.494978 | 0.310319 | 0.654765 | 0.960792 | 0.852894 | 0.536738 | 0.01296 |
| ALGA0071744 | 13 | 1.22E+08 | 0.014376 | 0.367181 | 0.216796 | 0.102112 | 0.492228 | 0.897453 | 0.111009 | 0.356039 | 0.591303 | 0.842424 | 0.974782 | 0.043169 |
| DRGA0014654 | 14 | 1.37E+08 | 0.014412 | 0.355207 | 0.054228 | 0.01365 | 0.839878 | 0.454438 | 0.435458 | 0.933106 | 0.8022 | 0.72121 | 0.391649 | 0.437963 |
| ASGA0079405 | 18 | 29095800 | 0.014427 | 0.349327 | 0.290676 | 0.144851 | 0.306275 | 0.408999 | 0.241369 | 0.762497 | 0.607008 | 0.386937 | 0.616839 | 0.203459 |
| ALGA0045511 | 7 | 1.27E+08 | 0.014453 | 0.414872 | 0.031152 | 0.06517 | 0.007905 | 0.885628 | 0.43347 | 0.149359 | 0.488045 | 0.459359 | 0.690104 | 0.073751 |
| ALGA0123050 | 4 | 131971 | 0.014477 | 0.825116 | 0.20015 | 0.262629 | 0.040556 | 0.45797 | 0.863117 | 0.277336 | 0.129898 | 0.207641 | 0.437727 | 0.088329 |
| M1GA0005042 | 4 | 233274 | 0.014477 | 0.825116 | 0.20015 | 0.262629 | 0.040556 | 0.45797 | 0.863117 | 0.277336 | 0.129898 | 0.207641 | 0.437727 | 0.088329 |
| ASGA0008247 | 0 | 0 | 0.014488 | 0.499743 | 0.152739 | 0.313758 | 0.285939 | 0.971056 | 0.32772 | 0.19912 | 0.651621 | 0.636423 | 0.178798 | 0.413636 |
| ASGA0101797 | 13 | 1.98E+08 | 0.014511 | 0.728103 | 0.380891 | 0.797504 | 0.30935 | 0.974882 | 0.019467 | 0.125484 | 0.778805 | 0.443626 | 0.874387 | 0.237422 |
| MARC0085835 | 13 | 1.98E+08 | 0.014511 | 0.728103 | 0.380891 | 0.797504 | 0.30935 | 0.974882 | 0.019467 | 0.125484 | 0.778805 | 0.443626 | 0.874387 | 0.237422 |
| MARC0034448 | 1 | 14131868 | 0.014519 | 0.112452 | 0.003508 | 0.305572 | 0.121221 | 0.450832 | 0.577535 | 0.443833 | 0.458291 | 0.558551 | 0.942646 | 0.006346 |
| MARC0099274 | 6 | 29437492 | 0.014542 | 0.354074 | 0.075954 | 0.200918 | 0.353471 | 0.62479 | 0.055153 | 0.841853 | 0.382673 | 0.925902 | 0.709936 | 0.522648 |
| MARC0068181 | 1 | 8728718 | 0.014557 | 0.866712 | 0.125297 | 0.049998 | 0.020342 | 0.899918 | 0.359635 | 0.19382 | 0.506436 | 0.834815 | 0.871595 | 0.008087 |
| ALGA0069655 | 13 | 40050308 | 0.014612 | 0.438808 | 0.02336 | 0.750318 | 0.993043 | 0.873117 | 0.322175 | 0.404472 | 0.882316 | 0.568812 | 0.693646 | 0.002118 |
| ASGA0064744 | 14 | 87970866 | 0.014653 | 0.16179 | 0.110107 | 0.196826 | 0.001167 | 0.642273 | 0.83572 | 0.202642 | 0.37106 | 0.313955 | 0.605942 | 0.282925 |
| ALGA0049057 | 8 | 1.14E+08 | 0.014655 | 0.194144 | 0.998161 | 0.064617 | 0.441721 | 0.290744 | 0.184387 | 0.613688 | 0.17112 | 0.773811 | 0.821941 | 0.386596 |
| MARC0011772 | 2 | 1.17E+08 | 0.014661 | 0.084808 | 0.078987 | 0.077939 | 0.360247 | 0.515641 | 0.044968 | 0.700831 | 0.117057 | 0.963332 | 0.761086 | 0.307759 |
| ASGA0001086 | 1 | 14111830 | 0.014663 | 0.113615 | 0.003797 | 0.32281 | 0.117103 | 0.487498 | 0.643845 | 0.457504 | 0.417623 | 0.576099 | 0.972949 | 0.006804 |
| ALGA0103570 | 16 | 23225447 | 0.014665 | 0.165149 | 0.021939 | 0.294955 | 0.259192 | 0.716491 | 0.472874 | 0.657651 | 0.603597 | 0.326491 | 0.159449 | 0.281935 |
| ALGA0074772 | 14 | 7637360 | 0.014673 | 0.327777 | 0.131876 | 0.210055 | 0.919756 | 0.804011 | 0.65236 | 0.342713 | 0.463564 | 0.099866 | 0.143436 | 0.428121 |
| ASGA0060896 | 14 | 7718162 | 0.014673 | 0.327777 | 0.131876 | 0.210055 | 0.919756 | 0.804011 | 0.65236 | 0.342713 | 0.463564 | 0.099866 | 0.143436 | 0.428121 |
| ALGA0066854 | 12 | 51261422 | 0.014684 | 0.223378 | 0.004817 | 0.011164 | 0.31404 | 0.101694 | 0.032712 | 0.18436 | 0.4103 | 0.183009 | 0.296431 | 0.072741 |
| ASGA0072998 | 16 | 35614082 | 0.014717 | 0.371772 | 0.081759 | 0.225013 | 0.100644 | 0.598926 | 0.773624 | 0.173991 | 0.577533 | 0.316024 | 0.054517 | 0.581656 |
| ALGA0086328 | 15 | 96617570 | 0.014747 | 0.759029 | 0.034472 | 0.847155 | 0.124339 | 0.352308 | 0.751276 | 0.173828 | 0.054492 | 0.119717 | 0.080478 | 0.428449 |
| ALGA0077688 | 14 | 58206487 | 0.014752 | 0.930586 | 0.122718 | 0.932096 | 0.00444 | 0.311873 | 0.043424 | 0.00137 | 0.956919 | 0.156886 | 0.199434 | 0.765328 |
| ASGA0054385 | 12 | 37593431 | 0.014772 | 0.503853 | 0.341964 | 0.475275 | 0.001458 | 0.056563 | 0.713709 | 0.000101 | 0.029339 | 0.459066 | 0.629887 | 0.076507 |
| ASGA0022483 | 4 | 1.29E+08 | 0.014784 | 0.916056 | 0.198947 | 0.163434 | 0.036362 | 0.901263 | 0.205944 | 0.429247 | 0.943948 | 0.478953 | 0.16036 | 0.505861 |
| MARC0010355 | 17 | 30355867 | 0.014795 | 0.778358 | 0.017777 | 0.737282 | 0.513036 | 0.149121 | 0.926383 | 0.898122 | 0.553949 | 0.334157 | 0.206045 | 0.072627 |
| DRGA0013341 | 13 | 1.96E+08 | 0.01487 | 0.274942 | 0.061026 | 0.120392 | 0.811698 | 0.313444 | 0.067588 | 0.017833 | 0.713508 | 0.007577 | 0.327681 | 0.008207 |
| ASGA0076020 | 17 | 31332142 | 0.014882 | 0.5708 | 0.021261 | 0.539708 | 0.295266 | 0.633045 | 0.96999 | 0.390202 | 0.57506 | 0.476104 | 0.306523 | 0.000784 |
| MARC0031010 | 7 | 93819194 | 0.014886 | 0.055886 | 0.039671 | 0.282021 | 0.065098 | 0.299282 | 0.668459 | 0.143139 | 0.220532 | 0.392066 | 0.441044 | 0.012738 |
| DIAS0001629 | 14 | 59116784 | 0.014928 | 0.968514 | 0.110924 | 0.76694 | 0.002062 | 0.153059 | 0.085136 | 0.007204 | 0.721159 | 0.328268 | 0.28871 | 0.695684 |
| ALGA0071962 | 13 | 1.39E+08 | 0.01499 | 0.06801 | 0.30978 | 0.001594 | 0.083457 | 0.181535 | 0.07663 | 0.990863 | 0.473767 | 0.027056 | 0.540291 | 0.020478 |
| MARC0042258 | 14 | 1.41E+08 | 0.015017 | 0.573912 | 0.19092 | 0.038021 | 0.220296 | 0.821914 | 0.627226 | 0.780143 | 0.332554 | 0.410064 | 0.531376 | 0.39555 |
| ALGA0048521 | 8 | 91798086 | 0.015024 | 0.342085 | 0.06298 | 0.988296 | 0.080724 | 0.15719 | 0.555151 | 0.230855 | 0.111041 | 0.186246 | 0.100917 | 0.217497 |
| DRGA0014662 | 14 | 1.37E+08 | 0.015027 | 0.34795 | 0.070446 | 0.011909 | 0.847107 | 0.358839 | 0.424649 | 0.909361 | 0.938708 | 0.595239 | 0.286704 | 0.52549 |
| H3GA0040445 | 14 | 62339756 | 0.015034 | 0.988064 | 0.212334 | 0.638425 | 0.034464 | 0.515365 | 0.839548 | 0.562289 | 0.345935 | 0.445861 | 0.643074 | 0.007618 |
| M1GA0008657 | 6 | 65787016 | 0.015037 | 0.614985 | 0.062431 | 0.277443 | 0.022845 | 0.508487 | 0.669713 | 0.179208 | 0.068094 | 0.784877 | 0.215029 | 0.403205 |
| H3GA0007271 | 2 | 1.08E+08 | 0.015093 | 0.049885 | 0.215804 | 0.068986 | 0.126599 | 0.98361 | 0.292966 | 0.599747 | 0.824772 | 0.889364 | 0.795625 | 0.163344 |
| ALGA0053654 | 0 | 0 | 0.015127 | 0.25329 | 0.017186 | 0.04512 | 0.79321 | 0.289011 | 0.410978 | 0.686427 | 0.273994 | 0.199346 | 0.721294 | 0.00867 |
| ASGA0039155 | 8 | 87740951 | 0.015164 | 0.788747 | 0.017748 | 0.030036 | 0.504521 | 0.530839 | 0.529042 | 0.309376 | 0.681576 | 0.298676 | 0.026408 | 0.35019 |
| H3GA0054964 | 12 | 34026709 | 0.015201 | 0.773456 | 0.360249 | 0.483748 | 0.008339 | 0.0059 | 0.426294 | 0.008343 | 0.03581 | 0.280727 | 0.323495 | 0.044777 |
| SIRI0000276 | 4 | 15102584 | 0.015225 | 0.985097 | 0.29047 | 0.884292 | 0.193094 | 0.279539 | 0.685356 | 0.802001 | 0.136691 | 0.927884 | 0.941328 | 0.398433 |
| ALGA0106138 | 2 | 1.27E+08 | 0.015266 | 0.515779 | 0.202162 | 0.606356 | 0.009233 | 0.111538 | 0.357024 | 0.508599 | 0.076656 | 0.053071 | 0.020714 | 0.892712 |
| ALGA0044402 | 7 | 1.13E+08 | 0.015266 | 0.505221 | 0.530507 | 0.589343 | 0.007154 | 0.656483 | 0.453803 | 0.152114 | 0.23157 | 0.734938 | 0.745289 | 0.301502 |
| ASGA0098633 | 6 | 68267328 | 0.015279 | 0.326467 | 0.036191 | 0.031082 | 0.022728 | 0.592424 | 0.726935 | 0.015912 | 0.200733 | 0.246113 | 0.113151 | 0.277355 |
| H3GA0037766 | 13 | 1.95E+08 | 0.015327 | 0.307121 | 0.117411 | 0.26479 | 0.510318 | 0.550015 | 0.046356 | 0.005762 | 0.752796 | 0.025462 | 0.333687 | 0.007967 |
| ASGA0054436 | 12 | 40148323 | 0.015332 | 0.821492 | 0.245294 | 0.948458 | 0.004441 | 0.027653 | 0.419262 | 0.010253 | 0.093154 | 0.38253 | 0.119872 | 0.017415 |
| ALGA0081939 | 14 | 1.36E+08 | 0.015377 | 0.905476 | 0.392968 | 0.025989 | 0.101698 | 0.72437 | 0.482808 | 0.428715 | 0.756389 | 0.982165 | 0.780049 | 0.089633 |
| ASGA0066750 | 14 | 1.36E+08 | 0.015377 | 0.905476 | 0.392968 | 0.025989 | 0.101698 | 0.72437 | 0.482808 | 0.428715 | 0.756389 | 0.982165 | 0.780049 | 0.089633 |
| ALGA0032465 | 5 | 67994769 | 0.015395 | 0.197542 | 0.518136 | 0.684072 | 0.085145 | 0.20993 | 0.353612 | 0.116281 | 0.9809 | 0.065276 | 0.079777 | 0.799711 |
| ALGA0122613 | 2 | 1.55E+08 | 0.015411 | 0.094694 | 0.049569 | 0.445798 | 0.486779 | 0.276983 | 0.074128 | 0.766772 | 0.156237 | 0.263089 | 0.361461 | 0.026662 |
| ASGA0083415 | 12 | 42766036 | 0.015412 | 0.526809 | 0.009606 | 0.333016 | 0.303843 | 0.784597 | 0.890576 | 0.099486 | 0.996601 | 0.518565 | 0.017347 | 0.047546 |
| MARC0108602 | 12 | 40115667 | 0.015413 | 0.958901 | 0.068096 | 0.983147 | 0.04272 | 0.067877 | 0.210975 | 0.04251 | 0.150946 | 0.567289 | 0.006217 | 0.130209 |
| ASGA0059105 | 13 | 1.57E+08 | 0.015444 | 0.153812 | 0.208435 | 0.100469 | 0.069598 | 0.860264 | 0.252039 | 0.339116 | 0.657665 | 0.14799 | 0.915407 | 0.128564 |
| ALGA0066740 | 12 | 50578018 | 0.01551 | 0.82704 | 0.024139 | 0.063836 | 0.140494 | 0.61664 | 0.029358 | 0.143056 | 0.542647 | 0.293522 | 0.470405 | 0.146606 |
| DIAS0000173 | 1 | 3.03E+08 | 0.015523 | 0.555685 | 0.192478 | 0.076947 | 0.140645 | 0.590775 | 0.284093 | 0.291306 | 0.708427 | 0.971423 | 0.517 | 0.656971 |
| MARC0076935 | 9 | 1.35E+08 | 0.015547 | 0.445052 | 0.038293 | 0.00788 | 0.545351 | 0.080046 | 0.108527 | 0.795417 | 0.095959 | 0.474224 | 0.982738 | 0.078938 |
| MARC0053051 | 0 | 0 | 0.01556 | 0.115328 | 0.271325 | 0.019427 | 0.014083 | 0.897892 | 0.973899 | 0.084191 | 0.234279 | 0.961556 | 0.096621 | 0.247217 |
| MARC0100557 | 5 | 3398453 | 0.015565 | 0.555117 | 0.181156 | 0.185472 | 0.293142 | 0.446926 | 0.160713 | 0.718895 | 0.197452 | 0.568119 | 0.06515 | 0.194937 |
| ALGA0118086 | 16 | 18836023 | 0.015569 | 0.262459 | 0.109019 | 0.295988 | 0.042276 | 0.312179 | 0.834759 | 0.057254 | 0.41124 | 0.273197 | 0.229849 | 0.104016 |
| H3GA0028426 | 9 | 1.42E+08 | 0.015626 | 0.86838 | 0.15811 | 0.555423 | 0.117181 | 0.232044 | 0.756608 | 0.687522 | 0.632206 | 0.048413 | 0.139991 | 0.263477 |
| MARC0057006 | 14 | 1.37E+08 | 0.015669 | 0.21409 | 0.067493 | 0.013451 | 0.865124 | 0.314437 | 0.442986 | 0.85964 | 0.902678 | 0.47119 | 0.25589 | 0.479447 |
| ALGA0066725 | 12 | 50281949 | 0.015679 | 0.774949 | 0.019246 | 0.068217 | 0.156704 | 0.654419 | 0.034189 | 0.164274 | 0.748288 | 0.246985 | 0.465253 | 0.177863 |
| INRA0029438 | 8 | 27921662 | 0.015691 | 0.448964 | 0.022432 | 0.017078 | 0.059538 | 0.432976 | 0.08218 | 0.385184 | 0.93588 | 0.560259 | 0.349404 | 0.252465 |
| H3GA0040291 | 14 | 59004595 | 0.015703 | 0.808764 | 0.122243 | 0.683931 | 0.001369 | 0.113754 | 0.098396 | 0.007412 | 0.651869 | 0.263852 | 0.292862 | 0.68223 |
| ASGA0059890 | 13 | 2.08E+08 | 0.015737 | 0.262047 | 0.373767 | 0.381194 | 0.55767 | 0.96638 | 0.547845 | 0.77133 | 0.8013 | 0.293042 | 0.357663 | 0.379448 |
| MARC0113549 | 14 | 1.51E+08 | 0.015767 | 0.989483 | 0.009163 | 0.094193 | 0.143212 | 0.897275 | 0.409022 | 0.981719 | 0.699419 | 0.550461 | 0.643211 | 0.413918 |
| MARC0048217 | 8 | 1.12E+08 | 0.015844 | 0.169764 | 0.01646 | 0.045126 | 0.155608 | 0.873943 | 0.812368 | 0.412007 | 0.556929 | 0.2514 | 0.075575 | 0.103789 |
| ASGA0051928 | 11 | 80719071 | 0.015845 | 0.840207 | 0.050676 | 0.154517 | 0.440216 | 0.191428 | 0.066408 | 0.612172 | 0.864992 | 0.446681 | 0.433564 | 0.124168 |
| H3GA0034642 | 12 | 51348817 | 0.015852 | 0.227548 | 0.007563 | 0.013518 | 0.2437 | 0.179015 | 0.047209 | 0.18382 | 0.652558 | 0.219871 | 0.225529 | 0.146321 |
| ALGA0117588 | 6 | 89421512 | 0.015877 | 0.648625 | 0.319021 | 0.061422 | 0.211545 | 0.457211 | 0.202104 | 0.492452 | 0.435866 | 0.337259 | 0.219396 | 0.502838 |
| MARC0040826 | 9 | 76083199 | 0.015889 | 0.49691 | 0.037453 | 6.25E-05 | 0.403031 | 0.046871 | 0.089793 | 0.177492 | 0.370609 | 0.117502 | 0.75611 | 0.05376 |
| ASGA0102230 | 0 | 0 | 0.01589 | 0.325896 | 0.062424 | 0.130971 | 0.251582 | 0.960436 | 0.309944 | 0.518213 | 0.934858 | 0.978124 | 0.830372 | 0.894644 |
| ALGA0086348 | 15 | 97866585 | 0.015898 | 0.423435 | 0.030041 | 0.513132 | 0.420545 | 0.671322 | 0.158321 | 0.425862 | 0.797005 | 0.757053 | 0.472875 | 0.130745 |
| INRA0049855 | 15 | 97940788 | 0.015898 | 0.423435 | 0.030041 | 0.513132 | 0.420545 | 0.671322 | 0.158321 | 0.425862 | 0.797005 | 0.757053 | 0.472875 | 0.130745 |
| MARC0101961 | 0 | 0 | 0.015904 | 0.902283 | 0.591033 | 0.007489 | 0.035002 | 0.199492 | 0.031856 | 0.335814 | 0.337886 | 0.704454 | 0.63548 | 0.496077 |
| ALGA0053793 | 9 | 78113024 | 0.015917 | 0.343306 | 0.098849 | 0.004443 | 0.117007 | 0.501115 | 0.919968 | 0.482121 | 0.681849 | 0.488332 | 0.159863 | 0.165993 |
| ALGA0120489 | 12 | 26315684 | 0.015924 | 0.888171 | 0.564069 | 0.859008 | 0.001584 | 0.033759 | 0.986074 | 0.001422 | 0.024343 | 0.530284 | 0.886975 | 0.018939 |
| ASGA0084743 | 12 | 53907898 | 0.015941 | 0.0372 | 0.128655 | 0.546722 | 0.030208 | 0.56882 | 0.448748 | 0.09685 | 0.765197 | 0.659177 | 0.500788 | 0.076591 |
| ASGA0054883 | 12 | 50340265 | 0.015948 | 0.7741 | 0.021557 | 0.062341 | 0.120806 | 0.666325 | 0.037181 | 0.137124 | 0.633415 | 0.318832 | 0.4355 | 0.143025 |
| ALGA0071739 | 13 | 1.22E+08 | 0.015965 | 0.338454 | 0.222625 | 0.158418 | 0.481905 | 0.845711 | 0.214064 | 0.415274 | 0.703475 | 0.880921 | 0.622977 | 0.045916 |
| MARC0000554 | 8 | 67026060 | 0.015987 | 0.168988 | 0.000573 | 0.013252 | 0.434662 | 0.273299 | 0.379452 | 0.757566 | 0.806505 | 0.761658 | 0.134441 | 0.219702 |
| ALGA0066249 | 12 | 37725107 | 0.016038 | 0.421462 | 0.303713 | 0.40325 | 0.001657 | 0.128664 | 0.516143 | 8.66E-05 | 0.036704 | 0.610246 | 0.718725 | 0.031978 |
| MARC0016953 | 17 | 12967074 | 0.016059 | 0.620363 | 0.082576 | 0.181957 | 0.275006 | 0.395608 | 0.065905 | 0.655802 | 0.586339 | 0.773147 | 0.92726 | 0.300254 |
| DRGA0011752 | 12 | 39538432 | 0.016106 | 0.266723 | 0.111261 | 0.549768 | 0.027221 | 0.088211 | 0.742609 | 0.001093 | 0.127489 | 0.60723 | 0.090959 | 0.261863 |
| MARC0074396 | 8 | 89821072 | 0.016109 | 0.480183 | 0.180858 | 0.279156 | 0.465049 | 0.738994 | 0.426493 | 0.399579 | 0.93529 | 0.236978 | 0.058266 | 0.516241 |
| ALGA0070703 | 13 | 72118478 | 0.016112 | 0.187707 | 0.109979 | 0.450708 | 0.805889 | 0.586634 | 0.411073 | 0.977944 | 0.798029 | 0.967707 | 0.403477 | 0.014864 |
| ALGA0048866 | 8 | 1.09E+08 | 0.016125 | 0.767092 | 0.18714 | 0.19709 | 0.356083 | 0.752931 | 0.118172 | 0.325724 | 0.991496 | 0.794458 | 0.808589 | 0.029774 |
| ALGA0119395 | 2 | 26321934 | 0.016133 | 0.124819 | 0.001548 | 0.007463 | 0.4631 | 0.17257 | 0.391477 | 0.843257 | 0.716973 | 0.732901 | 0.119042 | 0.262822 |
| MARC0012087 | 6 | 10713304 | 0.016152 | 0.144451 | 0.076627 | 0.009857 | 0.288269 | 0.425457 | 0.194682 | 0.576032 | 0.467363 | 0.403696 | 0.991746 | 0.045999 |
| ALGA0120537 | 0 | 0 | 0.016157 | 0.115385 | 0.177164 | 0.332115 | 0.112804 | 0.113869 | 0.626778 | 0.092112 | 0.226091 | 0.388894 | 0.059224 | 0.890442 |
| ALGA0119522 | 13 | 1.14E+08 | 0.01625 | 0.300543 | 0.090087 | 0.345462 | 0.550868 | 0.399577 | 0.28452 | 0.599909 | 0.899891 | 0.904206 | 0.623651 | 0.011451 |
| ALGA0028999 | 4 | 1.34E+08 | 0.016252 | 0.751892 | 0.111801 | 0.079356 | 0.977519 | 0.149205 | 0.290971 | 0.894022 | 0.396112 | 0.330235 | 0.880184 | 0.02708 |
| ALGA0097635 | 18 | 27610592 | 0.016291 | 0.538958 | 0.209784 | 0.152189 | 0.772691 | 0.687209 | 0.373588 | 0.983051 | 0.66529 | 0.923069 | 0.506716 | 0.615038 |
| ALGA0097638 | 18 | 27637420 | 0.016291 | 0.538958 | 0.209784 | 0.152189 | 0.772691 | 0.687209 | 0.373588 | 0.983051 | 0.66529 | 0.923069 | 0.506716 | 0.615038 |
| DIAS0001265 | 18 | 27666993 | 0.016291 | 0.538958 | 0.209784 | 0.152189 | 0.772691 | 0.687209 | 0.373588 | 0.983051 | 0.66529 | 0.923069 | 0.506716 | 0.615038 |
| DIAS0001266 | 18 | 27699432 | 0.016291 | 0.538958 | 0.209784 | 0.152189 | 0.772691 | 0.687209 | 0.373588 | 0.983051 | 0.66529 | 0.923069 | 0.506716 | 0.615038 |
| MARC0075832 | 12 | 60955830 | 0.016307 | 0.804389 | 0.001883 | 0.051653 | 0.095781 | 0.913556 | 0.59578 | 0.072459 | 0.21273 | 0.690232 | 0.005737 | 0.081369 |
| ALGA0068539 | 13 | 18485202 | 0.016366 | 0.156378 | 0.119589 | 0.412806 | 0.621899 | 0.567046 | 0.072623 | 0.146612 | 0.937847 | 0.815191 | 0.981599 | 0.008494 |
| ALGA0023792 | 4 | 19548139 | 0.016372 | 0.419267 | 0.051646 | 0.278252 | 0.667829 | 0.92055 | 0.693155 | 0.14467 | 0.776079 | 0.592764 | 0.313759 | 0.444963 |
| DRGA0001385 | 1 | 1.05E+08 | 0.016427 | 0.700352 | 0.055324 | 0.365074 | 0.389237 | 0.747495 | 0.516416 | 0.185935 | 0.832743 | 0.548398 | 0.28865 | 0.106396 |
| ALGA0078209 | 14 | 66064581 | 0.016442 | 0.962569 | 0.057452 | 0.369622 | 0.032564 | 0.459137 | 0.442451 | 0.195209 | 0.663416 | 0.749464 | 0.275869 | 0.622994 |
| ALGA0028868 | 4 | 1.32E+08 | 0.016485 | 0.644061 | 0.030423 | 0.029409 | 0.61184 | 0.134797 | 0.217552 | 0.426551 | 0.973485 | 0.50865 | 0.551564 | 0.125236 |
| MARC0043488 | 15 | 59388666 | 0.016519 | 0.181061 | 0.009449 | 0.007916 | 0.721278 | 0.0772 | 0.027183 | 0.939524 | 0.155779 | 0.140169 | 0.685557 | 0.159399 |
| ASGA0048164 | 10 | 52281702 | 0.016522 | 0.070947 | 0.177502 | 0.129166 | 0.092749 | 0.851865 | 0.17652 | 0.492408 | 0.976368 | 0.247771 | 0.856535 | 0.76 |
| ASGA0084103 | 2 | 1.62E+08 | 0.016527 | 0.011827 | 0.005672 | 0.015191 | 0.436805 | 0.051163 | 0.042653 | 0.65971 | 0.325622 | 0.993678 | 0.521523 | 0.125691 |
| MARC0041467 | 12 | 46855900 | 0.016535 | 0.645252 | 0.010423 | 0.211952 | 0.766084 | 0.956195 | 0.687959 | 0.048082 | 0.889278 | 0.757088 | 0.060532 | 0.265393 |
| ASGA0023892 | 5 | 4142177 | 0.016538 | 0.474771 | 0.279478 | 0.388045 | 0.370032 | 0.305483 | 0.162597 | 0.975403 | 0.214261 | 0.374507 | 0.068297 | 0.358364 |
| ASGA0037216 | 7 | 1.3E+08 | 0.016538 | 0.629397 | 0.087676 | 0.417499 | 0.041663 | 0.834178 | 0.694895 | 0.209404 | 0.64586 | 0.638552 | 0.95017 | 0.372198 |
| M1GA0005056 | 4 | 481531 | 0.016569 | 0.848669 | 0.212062 | 0.337264 | 0.044107 | 0.412262 | 0.903052 | 0.281184 | 0.132579 | 0.162531 | 0.474171 | 0.095412 |
| ASGA0000589 | 1 | 7781841 | 0.016573 | 0.867419 | 0.050684 | 0.092416 | 0.093482 | 0.672886 | 0.507744 | 0.727108 | 0.922033 | 0.475632 | 0.424008 | 0.249655 |
| MARC0041890 | 2 | 1.28E+08 | 0.016577 | 0.213306 | 0.052149 | 0.574413 | 0.069343 | 0.07765 | 0.817658 | 0.187889 | 0.040034 | 0.393885 | 0.21837 | 0.686276 |
| ALGA0097188 | 18 | 16373370 | 0.016627 | 0.101718 | 0.027702 | 0.426421 | 0.642824 | 0.423833 | 0.833182 | 0.463545 | 0.868978 | 0.951613 | 0.457288 | 0.07079 |
| MARC0024930 | 13 | 25799857 | 0.016636 | 0.39371 | 0.081893 | 0.548651 | 0.554127 | 0.997292 | 0.305052 | 0.385934 | 0.883277 | 0.93536 | 0.809945 | 0.014155 |
| ALGA0066283 | 12 | 39865928 | 0.016681 | 0.665326 | 0.313707 | 0.949714 | 0.000686 | 0.007532 | 0.477133 | 8.31E-05 | 0.039876 | 0.415718 | 0.2121 | 0.054426 |
| ALGA0104820 | 12 | 57002739 | 0.016719 | 0.852942 | 0.021179 | 0.305131 | 0.161067 | 0.81215 | 0.758983 | 0.033846 | 0.4708 | 0.541363 | 0.136755 | 0.127428 |
| ALGA0038110 | 7 | 4855447 | 0.016766 | 0.94853 | 0.235729 | 0.836848 | 0.671917 | 0.7442 | 0.503293 | 0.688089 | 0.741239 | 0.648716 | 0.787312 | 0.94342 |
| ALGA0063305 | 11 | 73852273 | 0.016772 | 0.614226 | 0.020553 | 0.082614 | 0.393849 | 0.967055 | 0.944782 | 0.649043 | 0.945037 | 0.444309 | 0.015541 | 0.521147 |
| ASGA0058690 | 13 | 1.22E+08 | 0.01678 | 0.305371 | 0.209225 | 0.165121 | 0.482801 | 0.795981 | 0.233958 | 0.399252 | 0.761625 | 0.978705 | 0.673754 | 0.040696 |
| DRGA0013936 | 14 | 67136417 | 0.0168 | 0.870898 | 0.134319 | 0.891922 | 0.003627 | 0.15128 | 0.57721 | 0.081833 | 0.251677 | 0.431807 | 0.383273 | 0.328212 |
| ALGA0047982 | 8 | 64602131 | 0.0168 | 0.184215 | 0.002097 | 0.010227 | 0.390906 | 0.255021 | 0.296639 | 0.752067 | 0.596417 | 0.851398 | 0.196688 | 0.254911 |
| ASGA0030016 | 6 | 1.45E+08 | 0.016807 | 0.491005 | 0.661773 | 0.007247 | 0.050901 | 0.178596 | 0.006488 | 0.035136 | 0.096205 | 0.981669 | 0.736263 | 0.564444 |
| ALGA0110143 | 0 | 0 | 0.016813 | 0.184647 | 0.035899 | 0.810001 | 0.741123 | 0.689218 | 0.706349 | 0.100475 | 0.979167 | 0.209463 | 0.351014 | 0.169799 |
| ALGA0015546 | 2 | 1.27E+08 | 0.016918 | 0.338281 | 0.080971 | 0.664418 | 0.088984 | 0.196458 | 0.554899 | 0.820191 | 0.126635 | 0.138652 | 0.03433 | 0.723686 |
| DRGA0013914 | 14 | 63336064 | 0.016939 | 0.881377 | 0.135721 | 0.788797 | 0.004334 | 0.174756 | 0.435973 | 0.09062 | 0.370067 | 0.398753 | 0.427824 | 0.347889 |
| ASGA0096327 | 6 | 71303189 | 0.016998 | 0.759208 | 0.041167 | 0.118497 | 0.031927 | 0.593149 | 0.995861 | 0.173431 | 0.269084 | 0.538632 | 0.290897 | 0.34847 |
| MARC0015128 | 1 | 8106889 | 0.017061 | 0.817551 | 0.04614 | 0.023809 | 0.018315 | 0.427719 | 0.080214 | 0.252941 | 0.693748 | 0.299942 | 0.71685 | 0.133008 |
| MARC0091319 | 10 | 59082877 | 0.017067 | 0.291159 | 0.355012 | 0.140918 | 0.193685 | 0.604471 | 0.421627 | 0.583682 | 0.422276 | 0.41142 | 0.986465 | 0.451263 |
| DRGA0012271 | 13 | 31183056 | 0.017074 | 0.652133 | 0.106777 | 0.600136 | 0.974867 | 0.747654 | 0.266065 | 0.454567 | 0.712021 | 0.879261 | 0.551606 | 0.006072 |
| INRA0030204 | 8 | 1.08E+08 | 0.017082 | 0.870246 | 0.19354 | 0.28037 | 0.285639 | 0.761322 | 0.062848 | 0.249638 | 0.984165 | 0.998247 | 0.962391 | 0.033498 |
| H3GA0055422 | 12 | 13901807 | 0.017084 | 0.820674 | 0.386235 | 0.466344 | 0.009237 | 0.132141 | 0.981937 | 0.186488 | 0.24648 | 0.801777 | 0.395667 | 0.633996 |
| ALGA0048019 | 8 | 67115392 | 0.017159 | 0.165344 | 0.000924 | 0.010014 | 0.393592 | 0.26291 | 0.352521 | 0.728918 | 0.779957 | 0.790107 | 0.138482 | 0.240328 |
| ALGA0048032 | 8 | 67669269 | 0.017159 | 0.165344 | 0.000924 | 0.010014 | 0.393592 | 0.26291 | 0.352521 | 0.728918 | 0.779957 | 0.790107 | 0.138482 | 0.240328 |
| ALGA0102491 | 8 | 69215722 | 0.017159 | 0.165344 | 0.000924 | 0.010014 | 0.393592 | 0.26291 | 0.352521 | 0.728918 | 0.779957 | 0.790107 | 0.138482 | 0.240328 |
| ALGA0103392 | 8 | 69146919 | 0.017159 | 0.165344 | 0.000924 | 0.010014 | 0.393592 | 0.26291 | 0.352521 | 0.728918 | 0.779957 | 0.790107 | 0.138482 | 0.240328 |
| ASGA0038884 | 8 | 69393260 | 0.017159 | 0.165344 | 0.000924 | 0.010014 | 0.393592 | 0.26291 | 0.352521 | 0.728918 | 0.779957 | 0.790107 | 0.138482 | 0.240328 |
| ASGA0085207 | 8 | 69065977 | 0.017159 | 0.165344 | 0.000924 | 0.010014 | 0.393592 | 0.26291 | 0.352521 | 0.728918 | 0.779957 | 0.790107 | 0.138482 | 0.240328 |
| H3GA0024937 | 8 | 69466912 | 0.017159 | 0.165344 | 0.000924 | 0.010014 | 0.393592 | 0.26291 | 0.352521 | 0.728918 | 0.779957 | 0.790107 | 0.138482 | 0.240328 |
| H3GA0024938 | 8 | 69622289 | 0.017159 | 0.165344 | 0.000924 | 0.010014 | 0.393592 | 0.26291 | 0.352521 | 0.728918 | 0.779957 | 0.790107 | 0.138482 | 0.240328 |
| MARC0084543 | 8 | 67568200 | 0.017159 | 0.165344 | 0.000924 | 0.010014 | 0.393592 | 0.26291 | 0.352521 | 0.728918 | 0.779957 | 0.790107 | 0.138482 | 0.240328 |
| MARC0095739 | 8 | 69146481 | 0.017159 | 0.165344 | 0.000924 | 0.010014 | 0.393592 | 0.26291 | 0.352521 | 0.728918 | 0.779957 | 0.790107 | 0.138482 | 0.240328 |
| ASGA0090983 | 12 | 43861937 | 0.017199 | 0.555077 | 0.020011 | 0.047878 | 0.190602 | 0.717723 | 0.709353 | 0.050171 | 0.924114 | 0.322843 | 0.005789 | 0.055642 |
| H3GA0055486 | 2 | 1.27E+08 | 0.017201 | 0.375039 | 0.177443 | 0.610468 | 0.01981 | 0.178549 | 0.444495 | 0.516024 | 0.056762 | 0.073457 | 0.036996 | 0.75915 |
| ASGA0092217 | 0 | 0 | 0.017211 | 0.106429 | 0.120104 | 0.274337 | 0.126961 | 0.243423 | 0.817036 | 0.066269 | 0.363699 | 0.194248 | 0.067426 | 0.689236 |
| ASGA0037601 | 8 | 7713312 | 0.017216 | 0.473579 | 0.080571 | 0.293012 | 0.52795 | 0.508129 | 0.994084 | 0.181546 | 0.438953 | 0.439039 | 0.526991 | 0.028142 |
| ALGA0119834 | 2 | 1.28E+08 | 0.017262 | 0.633035 | 0.081882 | 0.681374 | 0.123245 | 0.107476 | 0.063133 | 0.5065 | 0.012899 | 0.220541 | 0.012458 | 0.690827 |
| ALGA0048843 | 8 | 1.08E+08 | 0.017319 | 0.812469 | 0.195084 | 0.294122 | 0.283786 | 0.74503 | 0.065868 | 0.257595 | 0.972437 | 0.946552 | 0.962391 | 0.03303 |
| DBWU0000939 | 2 | 1.07E+08 | 0.017334 | 0.055045 | 0.293122 | 0.069735 | 0.104685 | 0.934132 | 0.246497 | 0.592459 | 0.923286 | 0.915473 | 0.992043 | 0.110489 |
| ALGA0120079 | 0 | 0 | 0.017341 | 0.329509 | 0.156859 | 0.078969 | 0.069949 | 0.134162 | 0.002786 | 0.074923 | 0.51876 | 0.815886 | 0.781191 | 0.114902 |
| ALGA0043153 | 7 | 93838774 | 0.01735 | 0.135684 | 0.079805 | 0.948022 | 0.004651 | 0.1637 | 0.484941 | 0.022066 | 0.066096 | 0.603132 | 0.694995 | 0.001326 |
| ASGA0057461 | 13 | 50531515 | 0.017377 | 0.214507 | 0.077898 | 0.846383 | 0.969019 | 0.856058 | 0.790298 | 0.388671 | 0.979949 | 0.30188 | 0.186379 | 0.025426 |
| ALGA0030374 | 5 | 9320895 | 0.017377 | 0.697529 | 0.045805 | 0.601214 | 0.19611 | 0.719191 | 0.424108 | 0.19153 | 0.790997 | 0.701593 | 0.181675 | 0.122335 |
| ASGA0094562 | 6 | 66500589 | 0.017381 | 0.092646 | 0.03073 | 0.175282 | 0.037847 | 0.276894 | 0.992828 | 0.00912 | 0.094121 | 0.354909 | 0.200128 | 0.14235 |
| DRGA0003514 | 2 | 1.3E+08 | 0.017399 | 0.643898 | 0.027416 | 0.804532 | 0.347219 | 0.323902 | 0.10921 | 0.809917 | 0.046743 | 0.307074 | 0.016945 | 0.297446 |
| ALGA0097668 | 18 | 27798704 | 0.017425 | 0.554783 | 0.187305 | 0.133775 | 0.824606 | 0.666687 | 0.374577 | 0.956745 | 0.68683 | 0.76375 | 0.574529 | 0.566947 |
| ASGA0047280 | 10 | 31882839 | 0.017431 | 0.287779 | 0.014669 | 0.923763 | 0.95651 | 0.432604 | 0.82637 | 0.72096 | 0.9342 | 0.518601 | 0.442606 | 0.171766 |
| ALGA0022092 | 4 | 2776716 | 0.01755 | 0.074829 | 0.063347 | 0.11588 | 0.062273 | 0.537111 | 0.27488 | 0.042526 | 0.953746 | 0.282734 | 0.333935 | 0.541933 |
| MARC0067252 | 4 | 1.3E+08 | 0.017578 | 0.793484 | 0.000961 | 0.226144 | 0.688199 | 0.516985 | 0.371426 | 0.689822 | 0.65733 | 0.753255 | 0.935084 | 0.052871 |
| ASGA0017613 | 4 | 6074828 | 0.017628 | 0.481886 | 0.007216 | 0.945328 | 0.376713 | 0.652767 | 0.196797 | 0.082638 | 0.455227 | 0.49692 | 0.245666 | 0.275541 |
| ASGA0029311 | 6 | 1.15E+08 | 0.017649 | 0.797345 | 0.112183 | 0.212001 | 0.322182 | 0.411776 | 0.753567 | 0.985719 | 0.811 | 0.221754 | 0.193938 | 0.603071 |
| ASGA0068126 | 14 | 1.51E+08 | 0.017676 | 0.935828 | 0.011324 | 0.108013 | 0.135838 | 0.981339 | 0.51496 | 0.953967 | 0.588347 | 0.540151 | 0.619051 | 0.480469 |
| ASGA0079479 | 0 | 0 | 0.017701 | 0.75106 | 0.292808 | 0.188476 | 0.656713 | 0.646865 | 0.225956 | 0.618844 | 0.464577 | 0.578601 | 0.778534 | 0.297213 |
| ASGA0091638 | 4 | 779106 | 0.017719 | 0.91133 | 0.060404 | 0.092664 | 0.506233 | 0.560853 | 0.490395 | 0.625074 | 0.379385 | 0.695336 | 0.132641 | 0.383348 |
| MARC0112726 | 9 | 1.36E+08 | 0.017723 | 0.633257 | 0.141977 | 0.147327 | 0.339266 | 0.964591 | 0.821873 | 0.329987 | 0.436664 | 0.412104 | 0.110334 | 0.524751 |
| ASGA0097135 | 2 | 1.43E+08 | 0.017723 | 0.906322 | 0.319063 | 0.079885 | 0.362964 | 0.425344 | 0.289004 | 0.328585 | 0.88878 | 0.13236 | 0.753558 | 0.101437 |
| ALGA0082976 | 14 | 1.48E+08 | 0.017756 | 0.857234 | 0.035614 | 0.148441 | 0.121026 | 0.977834 | 0.671493 | 0.919474 | 0.703973 | 0.22139 | 0.785184 | 0.6263 |
| MARC0097527 | 14 | 66239887 | 0.017771 | 0.81058 | 0.147453 | 0.868762 | 0.004857 | 0.172086 | 0.555974 | 0.100693 | 0.302583 | 0.498136 | 0.401134 | 0.34883 |
| ASGA0048359 | 10 | 59776918 | 0.017827 | 0.205295 | 0.191523 | 0.121348 | 0.271337 | 0.274598 | 0.399779 | 0.432109 | 0.634393 | 0.254318 | 0.50148 | 0.343822 |
| ASGA0096879 | 0 | 0 | 0.017856 | 0.403799 | 0.035854 | 0.416915 | 0.04854 | 0.575606 | 0.484626 | 0.267913 | 0.091947 | 0.352442 | 0.139912 | 0.092122 |
| ASGA0089299 | 0 | 0 | 0.017898 | 0.119114 | 0.228083 | 0.003755 | 0.00595 | 0.976874 | 0.72897 | 0.095054 | 0.202573 | 0.50231 | 0.192267 | 0.262511 |
| ALGA0084305 | 15 | 22394509 | 0.017902 | 0.357479 | 0.035474 | 0.165507 | 0.49928 | 0.746645 | 0.367698 | 0.299389 | 0.793487 | 0.771291 | 0.303715 | 0.739335 |
| MARC0012689 | 6 | 94404857 | 0.017908 | 0.437326 | 0.03168 | 0.022718 | 0.150856 | 0.935507 | 0.33534 | 0.413748 | 0.939921 | 0.714623 | 0.492474 | 0.183632 |
| ALGA0035073 | 6 | 29414327 | 0.017932 | 0.417451 | 0.042847 | 0.079844 | 0.744065 | 0.385175 | 0.031377 | 0.357735 | 0.218043 | 0.709029 | 0.872569 | 0.494039 |
| ASGA0039540 | 8 | 1.13E+08 | 0.017969 | 0.841768 | 0.235828 | 0.58311 | 0.155981 | 0.143966 | 0.452652 | 0.299246 | 0.498423 | 0.318837 | 0.410112 | 0.177914 |
| ALGA0102031 | 6 | 1.27E+08 | 0.018044 | 0.507515 | 0.147004 | 0.536761 | 0.556414 | 0.37431 | 0.910251 | 0.88946 | 0.316113 | 0.213095 | 0.252788 | 0.694054 |
| ALGA0104388 | 7 | 1.25E+08 | 0.018084 | 0.669527 | 0.039474 | 0.041422 | 0.241037 | 0.532011 | 0.106812 | 0.435789 | 0.856273 | 0.199657 | 0.941907 | 0.06086 |
| ALGA0053613 | 9 | 71333635 | 0.018113 | 0.241108 | 0.02134 | 0.137616 | 0.899292 | 0.237747 | 0.573386 | 0.631806 | 0.560056 | 0.89955 | 0.765755 | 0.289255 |
| ASGA0079373 | 18 | 27454936 | 0.018201 | 0.772991 | 0.248308 | 0.259684 | 0.811945 | 0.786812 | 0.631843 | 0.957236 | 0.915759 | 0.889492 | 0.492538 | 0.65789 |
| ASGA0054868 | 12 | 49919795 | 0.018222 | 0.44893 | 0.002755 | 0.183931 | 0.111598 | 0.837162 | 0.704403 | 0.051992 | 0.700805 | 0.169299 | 0.025635 | 0.082942 |
| DIAS0000181 | 13 | 40202551 | 0.018252 | 0.53393 | 0.035487 | 0.771345 | 0.944895 | 0.969 | 0.465295 | 0.499992 | 0.982909 | 0.539171 | 0.576002 | 0.003438 |
| ASGA0098229 | 12 | 60440086 | 0.018281 | 0.593909 | 0.00655 | 0.231404 | 0.011099 | 0.122882 | 0.254883 | 0.034604 | 0.042564 | 0.429543 | 0.030576 | 0.044914 |
| ALGA0069635 | 13 | 39343080 | 0.018329 | 0.41162 | 0.043444 | 0.939291 | 0.910538 | 0.84116 | 0.440587 | 0.437484 | 0.929115 | 0.383785 | 0.78325 | 0.000606 |
| ALGA0069640 | 13 | 39432625 | 0.018329 | 0.41162 | 0.043444 | 0.939291 | 0.910538 | 0.84116 | 0.440587 | 0.437484 | 0.929115 | 0.383785 | 0.78325 | 0.000606 |
| DRGA0012191 | 13 | 22051370 | 0.018338 | 0.513315 | 0.037462 | 0.036914 | 0.153717 | 0.487692 | 0.062834 | 0.398462 | 0.32212 | 0.950508 | 0.678396 | 0.168707 |
| M1GA0020474 | 15 | 1.38E+08 | 0.01846 | 0.918593 | 0.711857 | 0.480242 | 0.037386 | 0.693704 | 0.127131 | 0.084701 | 0.959625 | 0.265515 | 0.561076 | 0.456802 |
| ASGA0069878 | 15 | 85698696 | 0.018472 | 0.791814 | 0.152421 | 0.004024 | 0.1084 | 0.274828 | 0.085636 | 0.493082 | 0.414391 | 0.797204 | 0.965195 | 0.517834 |
| ALGA0069645 | 13 | 39535679 | 0.018472 | 0.311577 | 0.046762 | 0.90428 | 0.930723 | 0.812581 | 0.443323 | 0.576769 | 0.916729 | 0.314599 | 0.788268 | 0.000344 |
| MARC0011323 | 13 | 39594138 | 0.018472 | 0.311577 | 0.046762 | 0.90428 | 0.930723 | 0.812581 | 0.443323 | 0.576769 | 0.916729 | 0.314599 | 0.788268 | 0.000344 |
| ASGA0001985 | 1 | 33581035 | 0.01848 | 0.189073 | 0.039127 | 0.851866 | 0.760119 | 0.733063 | 0.704252 | 0.100023 | 0.918884 | 0.195449 | 0.355634 | 0.155824 |
| ASGA0022654 | 4 | 1.32E+08 | 0.018496 | 0.282958 | 0.072474 | 0.081422 | 0.470402 | 0.435059 | 0.654179 | 0.649773 | 0.721166 | 0.667688 | 0.52064 | 0.186826 |
| DIAS0003410 | 13 | 72412075 | 0.018609 | 0.289104 | 0.065135 | 0.287165 | 0.551614 | 0.368954 | 0.343285 | 0.914822 | 0.826918 | 0.942899 | 0.714255 | 0.15163 |
| MARC0047431 | 13 | 18515847 | 0.018647 | 0.718991 | 0.09294 | 0.209852 | 0.437277 | 0.349415 | 0.01482 | 0.14389 | 0.431781 | 0.344882 | 0.85931 | 0.27225 |
| ALGA0115592 | 12 | 49609581 | 0.018647 | 0.658689 | 0.005165 | 0.203079 | 0.084534 | 0.777704 | 0.622191 | 0.04036 | 0.976068 | 0.142632 | 0.038597 | 0.130037 |
| H3GA0024905 | 8 | 65027400 | 0.018657 | 0.185754 | 0.001338 | 0.008038 | 0.403739 | 0.266544 | 0.353002 | 0.778786 | 0.735172 | 0.8039 | 0.149253 | 0.250262 |
| INRA0029839 | 8 | 64448692 | 0.018657 | 0.185754 | 0.001338 | 0.008038 | 0.403739 | 0.266544 | 0.353002 | 0.778786 | 0.735172 | 0.8039 | 0.149253 | 0.250262 |
| ASGA0030017 | 6 | 1.45E+08 | 0.018664 | 0.266629 | 0.285283 | 0.002589 | 0.017829 | 0.219742 | 0.014478 | 0.016115 | 0.099688 | 0.873832 | 0.990321 | 0.502886 |
| ALGA0107518 | 0 | 0 | 0.018722 | 0.53993 | 9.67E-07 | 0.243003 | 0.352849 | 0.262198 | 0.029173 | 0.032637 | 0.048423 | 0.504292 | 3.06E-05 | 0.000637 |
| ALGA0069124 | 13 | 28893435 | 0.018747 | 0.193117 | 0.142665 | 0.514843 | 0.98583 | 0.787139 | 0.194162 | 0.518402 | 0.625929 | 0.822386 | 0.66943 | 0.004744 |
| ASGA0057937 | 13 | 71634446 | 0.018771 | 0.178871 | 0.260379 | 0.364085 | 0.38392 | 0.76985 | 0.154484 | 0.493999 | 0.92132 | 0.866841 | 0.531619 | 0.012081 |
| CASI0005843 | 10 | 40212472 | 0.018791 | 0.296542 | 0.426636 | 0.760947 | 0.006108 | 0.345912 | 0.431856 | 0.092419 | 0.160461 | 0.32358 | 0.822785 | 0.20401 |
| ALGA0094202 | 17 | 33214799 | 0.018827 | 0.292272 | 0.08378 | 0.053609 | 0.199082 | 0.614301 | 0.300795 | 0.625147 | 0.526564 | 0.90264 | 0.269844 | 0.062945 |
| DRGA0014668 | 14 | 1.37E+08 | 0.018832 | 0.668867 | 0.239855 | 0.034464 | 0.285434 | 0.993307 | 0.804197 | 0.962679 | 0.396853 | 0.456037 | 0.701737 | 0.413685 |
| ASGA0092318 | 1 | 2.49E+08 | 0.018854 | 0.332264 | 0.110427 | 0.731507 | 0.006857 | 0.050192 | 0.720717 | 0.035349 | 0.121154 | 0.414798 | 0.143905 | 0.079599 |
| ASGA0038898 | 8 | 70033358 | 0.018911 | 0.946838 | 0.016325 | 0.010026 | 0.765566 | 0.182678 | 0.386615 | 0.078251 | 0.162098 | 0.698829 | 0.19826 | 0.044185 |
| ASGA0085630 | 5 | 19332302 | 0.018937 | 0.969913 | 0.905616 | 0.424253 | 0.147201 | 0.394097 | 0.670156 | 0.253631 | 0.126069 | 0.4964 | 0.239064 | 0.373418 |
| MARC0077581 | 16 | 3289936 | 0.018955 | 0.973863 | 0.442866 | 0.008623 | 0.200995 | 0.174274 | 0.009368 | 0.397872 | 0.452 | 0.401147 | 0.755564 | 0.970208 |
| H3GA0056180 | 12 | 41548842 | 0.019012 | 0.534288 | 0.038915 | 0.801637 | 0.032237 | 0.032844 | 0.073118 | 0.00385 | 0.134256 | 0.551855 | 0.00496 | 0.185698 |
| DRGA0009899 | 9 | 1.41E+08 | 0.01905 | 0.795649 | 0.602361 | 0.917037 | 0.02525 | 0.026358 | 0.984747 | 0.041889 | 0.548307 | 0.040156 | 0.03473 | 0.440861 |
| ALGA0068519 | 13 | 17956613 | 0.019051 | 0.583103 | 0.104697 | 0.244945 | 0.398829 | 0.407342 | 0.008554 | 0.09826 | 0.457744 | 0.464505 | 0.702912 | 0.313603 |
| ALGA0028873 | 4 | 1.32E+08 | 0.019087 | 0.438553 | 0.106646 | 0.086 | 0.496162 | 0.384617 | 0.826214 | 0.766518 | 0.721581 | 0.82574 | 0.270294 | 0.224667 |
| ASGA0093354 | 6 | 68230725 | 0.019104 | 0.204174 | 0.103201 | 0.091219 | 0.041092 | 0.649155 | 0.252553 | 0.487023 | 0.504577 | 0.871546 | 0.758896 | 0.165512 |
| ALGA0000673 | 1 | 8942360 | 0.019129 | 0.609343 | 0.268512 | 0.446711 | 0.011918 | 0.53694 | 0.695121 | 0.966284 | 0.310635 | 0.640152 | 0.839496 | 0.452912 |
| ALGA0081025 | 14 | 1.2E+08 | 0.019145 | 0.99442 | 0.283936 | 0.394717 | 0.168611 | 0.464137 | 0.221386 | 0.483061 | 0.707459 | 0.09963 | 0.986685 | 0.129812 |
| ALGA0077736 | 14 | 58940195 | 0.019159 | 0.683825 | 0.082313 | 0.804207 | 0.022056 | 0.199969 | 0.444714 | 0.12746 | 0.724358 | 0.399341 | 0.131268 | 0.706056 |
| ALGA0079138 | 14 | 84662469 | 0.019181 | 0.0681 | 0.218391 | 0.273043 | 0.071761 | 0.816995 | 0.48227 | 0.905179 | 0.543033 | 0.520562 | 0.777037 | 0.197816 |
| H3GA0041092 | 14 | 84850432 | 0.019181 | 0.0681 | 0.218391 | 0.273043 | 0.071761 | 0.816995 | 0.48227 | 0.905179 | 0.543033 | 0.520562 | 0.777037 | 0.197816 |
| DRGA0014290 | 14 | 1.02E+08 | 0.019195 | 0.3014 | 0.019287 | 0.63695 | 0.549462 | 0.230459 | 0.210599 | 0.662828 | 0.174907 | 0.22764 | 0.026897 | 0.532895 |
| ASGA0073168 | 16 | 41694847 | 0.019204 | 0.714369 | 0.051312 | 0.219257 | 0.036304 | 0.22218 | 0.62874 | 0.032133 | 0.325377 | 0.223077 | 0.200181 | 0.450604 |
| ALGA0121798 | 9 | 72174783 | 0.01923 | 0.494209 | 0.068004 | 0.102912 | 0.232495 | 0.591407 | 0.639129 | 0.662324 | 0.671545 | 0.541329 | 0.562829 | 0.115489 |
| INRA0043833 | 14 | 45918568 | 0.019248 | 0.360783 | 0.095198 | 0.811308 | 0.066287 | 0.325487 | 0.537551 | 0.393745 | 0.423998 | 0.489842 | 0.672919 | 0.441022 |
| MARC0034108 | 8 | 11323614 | 0.019259 | 0.397529 | 0.07163 | 0.094435 | 0.235046 | 0.88095 | 0.427245 | 0.045978 | 0.957289 | 0.160899 | 0.540083 | 0.050294 |
| INRA0028058 | 7 | 1.14E+08 | 0.019262 | 0.527252 | 0.042276 | 0.719337 | 0.78844 | 0.702466 | 0.299297 | 0.472937 | 0.61995 | 0.154697 | 0.052839 | 0.233686 |
| H3GA0006352 | 2 | 27409944 | 0.019322 | 0.356012 | 0.114232 | 0.937239 | 0.543692 | 0.300022 | 0.305232 | 0.892883 | 0.084331 | 0.724647 | 0.442412 | 0.573952 |
| ASGA0060629 | 14 | 5179572 | 0.019351 | 0.927453 | 0.085416 | 0.99405 | 0.093563 | 0.301381 | 0.922799 | 0.298448 | 0.41631 | 0.159095 | 0.295141 | 0.367276 |
| ASGA0094373 | 1 | 3.07E+08 | 0.019373 | 0.930285 | 0.055229 | 0.019353 | 0.948967 | 0.548056 | 0.702708 | 0.690552 | 0.63428 | 0.592988 | 0.125059 | 0.140373 |
| M1GA0016889 | 12 | 52061917 | 0.019403 | 0.232269 | 0.006315 | 0.016396 | 0.348588 | 0.102557 | 0.047628 | 0.218447 | 0.445219 | 0.166312 | 0.272696 | 0.090074 |
| ASGA0053920 | 12 | 26371789 | 0.019403 | 0.834531 | 0.472495 | 0.586673 | 0.011134 | 0.02225 | 0.743177 | 0.004592 | 0.010028 | 0.295336 | 0.461856 | 0.044551 |
| MARC0041437 | 11 | 54023378 | 0.019429 | 0.184806 | 0.054226 | 0.000708 | 0.435864 | 0.427566 | 0.721426 | 0.496731 | 0.438689 | 0.717096 | 0.144225 | 0.661703 |
| ALGA0123414 | 0 | 0 | 0.019431 | 0.745612 | 0.461341 | 0.366485 | 0.216554 | 0.917851 | 0.170456 | 0.275476 | 0.41566 | 0.773247 | 0.43537 | 0.269807 |
| ALGA0001317 | 1 | 17786299 | 0.019441 | 0.083279 | 0.079571 | 0.47694 | 0.190871 | 0.867313 | 0.902207 | 0.661055 | 0.724289 | 0.687449 | 0.677869 | 0.034534 |
| ALGA0001328 | 1 | 17771632 | 0.019441 | 0.083279 | 0.079571 | 0.47694 | 0.190871 | 0.867313 | 0.902207 | 0.661055 | 0.724289 | 0.687449 | 0.677869 | 0.034534 |
| ASGA0070437 | 15 | 1.28E+08 | 0.019483 | 0.718272 | 0.030402 | 0.324297 | 0.219638 | 0.233377 | 0.567312 | 0.129814 | 0.231711 | 0.270264 | 0.003743 | 0.83869 |
| ASGA0028507 | 6 | 69006213 | 0.019552 | 0.033871 | 0.095086 | 0.053363 | 0.023422 | 0.578873 | 0.190233 | 0.0107 | 0.260681 | 0.792709 | 0.500817 | 0.096996 |
| ALGA0048014 | 8 | 66622716 | 0.019561 | 0.12608 | 0.001177 | 0.017509 | 0.400534 | 0.299031 | 0.519877 | 0.842222 | 0.835363 | 0.66239 | 0.117374 | 0.158355 |
| ASGA0038870 | 8 | 66562625 | 0.019561 | 0.12608 | 0.001177 | 0.017509 | 0.400534 | 0.299031 | 0.519877 | 0.842222 | 0.835363 | 0.66239 | 0.117374 | 0.158355 |
| DRGA0008621 | 8 | 69950857 | 0.019561 | 0.12608 | 0.001177 | 0.017509 | 0.400534 | 0.299031 | 0.519877 | 0.842222 | 0.835363 | 0.66239 | 0.117374 | 0.158355 |
| DRGA0008622 | 8 | 69973631 | 0.019561 | 0.12608 | 0.001177 | 0.017509 | 0.400534 | 0.299031 | 0.519877 | 0.842222 | 0.835363 | 0.66239 | 0.117374 | 0.158355 |
| H3GA0032558 | 11 | 80624389 | 0.019565 | 0.548573 | 0.106643 | 0.395578 | 0.210893 | 0.359288 | 0.293192 | 0.816638 | 0.936039 | 0.871856 | 0.73706 | 0.282498 |
| MARC0016187 | 13 | 1.93E+08 | 0.019565 | 0.982593 | 0.06206 | 0.331584 | 0.082464 | 0.571416 | 0.662802 | 0.07136 | 0.17114 | 0.614979 | 0.522166 | 0.111494 |
| DRGA0009450 | 9 | 77844909 | 0.019579 | 0.393918 | 0.115266 | 0.000172 | 0.326531 | 0.15175 | 0.08116 | 0.266451 | 0.314099 | 0.120075 | 0.92046 | 0.043196 |
| H3GA0049633 | 17 | 61860123 | 0.019587 | 0.984723 | 0.091168 | 0.242329 | 0.077456 | 0.931152 | 0.381598 | 0.650872 | 0.680758 | 0.75319 | 0.971636 | 0.12885 |
| H3GA0015260 | 5 | 3844970 | 0.019598 | 0.456637 | 0.291081 | 0.42475 | 0.394435 | 0.285804 | 0.122053 | 0.897122 | 0.215126 | 0.427241 | 0.06439 | 0.366541 |
| ASGA0029546 | 6 | 1.27E+08 | 0.019612 | 0.356956 | 0.023184 | 0.021578 | 0.157628 | 0.202362 | 0.828632 | 0.53461 | 0.91026 | 0.928909 | 0.290024 | 0.221418 |
| ALGA0116869 | 0 | 0 | 0.019614 | 0.487794 | 0.134013 | 0.730934 | 0.982771 | 0.935757 | 0.36841 | 0.532767 | 0.751579 | 0.740473 | 0.418512 | 0.009515 |
| ASGA0037921 | 8 | 15710241 | 0.019719 | 0.478018 | 0.428751 | 0.199637 | 0.86632 | 0.378315 | 0.80369 | 0.632242 | 0.462381 | 0.03766 | 0.008418 | 0.676104 |
| ALGA0122390 | 12 | 42939598 | 0.019744 | 0.772993 | 0.118916 | 0.030372 | 0.373689 | 0.496469 | 0.029861 | 0.054015 | 0.401035 | 0.954743 | 0.862071 | 0.029384 |
| ALGA0097669 | 18 | 27826942 | 0.019751 | 0.606836 | 0.23997 | 0.16172 | 0.951054 | 0.560198 | 0.256226 | 0.934313 | 0.440816 | 0.727627 | 0.718075 | 0.642625 |
| ALGA0073131 | 13 | 1.91E+08 | 0.019768 | 0.812604 | 0.058949 | 0.243342 | 0.035062 | 0.618711 | 0.337917 | 0.103043 | 0.225025 | 0.440424 | 0.863389 | 0.076892 |
| ALGA0064081 | 11 | 83426107 | 0.019773 | 0.190737 | 0.129324 | 0.001136 | 0.376565 | 0.12886 | 0.52311 | 0.578102 | 0.848907 | 0.651296 | 0.124408 | 0.938602 |
| ALGA0071621 | 13 | 1.09E+08 | 0.019795 | 0.462122 | 0.088238 | 0.146413 | 0.373731 | 0.43044 | 0.382892 | 0.79029 | 0.830257 | 0.722674 | 0.627398 | 0.140152 |
| H3GA0035763 | 13 | 21338975 | 0.019806 | 0.387241 | 0.079767 | 0.144419 | 0.638364 | 0.629546 | 0.054667 | 0.348463 | 0.285556 | 0.94398 | 0.972731 | 0.154851 |
| MARC0105773 | 0 | 0 | 0.019897 | 0.141646 | 0.001236 | 0.010256 | 0.420507 | 0.232189 | 0.378517 | 0.771413 | 0.764621 | 0.798858 | 0.132862 | 0.274567 |
| ASGA0097565 | 9 | 79191592 | 0.019929 | 0.943732 | 0.265333 | 0.07882 | 0.031104 | 0.587472 | 0.236289 | 0.359524 | 0.570539 | 0.234909 | 0.688931 | 0.023451 |
| ALGA0028681 | 4 | 1.3E+08 | 0.019937 | 0.80108 | 0.005754 | 0.291423 | 0.552233 | 0.776626 | 0.615783 | 0.717171 | 0.68118 | 0.406825 | 0.574936 | 0.158628 |
| ALGA0066230 | 12 | 37220391 | 0.019941 | 0.628704 | 0.504919 | 0.957378 | 0.001107 | 0.008796 | 0.316301 | 0.000256 | 0.02043 | 0.348412 | 0.205291 | 0.114556 |
| MARC0002042 | 5 | 3350076 | 0.019996 | 0.502663 | 0.213563 | 0.226779 | 0.304607 | 0.511846 | 0.183718 | 0.962607 | 0.1969 | 0.522168 | 0.057514 | 0.261287 |
| ALGA0023800 | 4 | 19775001 | 0.02005 | 0.643679 | 0.026695 | 0.209232 | 0.731705 | 0.841629 | 0.952258 | 0.200192 | 0.958895 | 0.449373 | 0.335595 | 0.413223 |
| CASI0007582 | 11 | 75662097 | 0.020082 | 0.430132 | 0.083759 | 0.023864 | 0.058252 | 0.889503 | 0.395977 | 0.554314 | 0.714628 | 0.413842 | 0.096263 | 0.846366 |
| MARC0089979 | 8 | 73786978 | 0.020085 | 0.185729 | 0.001217 | 0.010129 | 0.377679 | 0.287038 | 0.386981 | 0.730691 | 0.865274 | 0.712026 | 0.120252 | 0.246101 |
| H3GA0042395 | 0 | 0 | 0.020115 | 0.453693 | 0.126155 | 0.09113 | 0.387743 | 0.752698 | 0.464925 | 0.447877 | 0.502073 | 0.487201 | 0.139988 | 0.513327 |
| ALGA0112879 | 8 | 1.11E+08 | 0.020132 | 0.639746 | 0.169356 | 0.492591 | 0.758299 | 0.775857 | 0.358803 | 0.597506 | 0.762119 | 0.599154 | 0.729745 | 0.0326 |
| ALGA0000972 | 1 | 13760382 | 0.020181 | 0.068318 | 0.004571 | 0.239865 | 0.102481 | 0.432013 | 0.439872 | 0.379268 | 0.524711 | 0.48364 | 0.977397 | 0.008661 |
| ASGA0106099 | 2 | 1.42E+08 | 0.020196 | 0.744903 | 0.340281 | 0.46902 | 0.605205 | 0.84665 | 0.502914 | 0.896899 | 0.957178 | 0.36142 | 0.558096 | 0.383758 |
| MARC0018791 | 14 | 1217329 | 0.020204 | 0.832203 | 0.001436 | 0.050734 | 0.895266 | 0.129922 | 0.727631 | 0.809588 | 0.981889 | 0.63603 | 0.542591 | 0.063468 |
| M1GA0003286 | 2 | 1.49E+08 | 0.020228 | 0.25642 | 0.010385 | 0.546345 | 0.517394 | 0.637963 | 0.539034 | 0.921119 | 0.965913 | 0.49486 | 0.260612 | 0.042346 |
| M1GA0026615 | 6 | 71836849 | 0.020235 | 0.17617 | 0.014728 | 0.389849 | 0.131702 | 0.328372 | 0.898415 | 0.026553 | 0.224965 | 0.860762 | 0.323259 | 0.371602 |
| MARC0029824 | 14 | 64026325 | 0.020246 | 0.960062 | 0.071443 | 0.671608 | 0.018796 | 0.209443 | 0.540597 | 0.174859 | 0.579867 | 0.590587 | 0.217463 | 0.643975 |
| ASGA0060745 | 14 | 6086000 | 0.020262 | 0.865803 | 0.255182 | 0.089494 | 0.766477 | 0.126635 | 0.397719 | 0.638385 | 0.510347 | 0.561789 | 0.704771 | 0.122235 |
| H3GA0039782 | 14 | 37677563 | 0.020323 | 0.168767 | 0.018918 | 0.465711 | 0.538869 | 0.490367 | 0.334669 | 0.636143 | 0.935726 | 0.189086 | 0.305382 | 0.572051 |
| MARC0059262 | 6 | 91608952 | 0.02037 | 0.370122 | 0.031799 | 0.495308 | 0.052294 | 0.518682 | 0.55174 | 0.216287 | 0.074652 | 0.335388 | 0.139269 | 0.17056 |
| MARC0112885 | 9 | 71762247 | 0.020389 | 0.784534 | 0.023065 | 0.000409 | 0.849479 | 0.001244 | 0.019535 | 0.470184 | 0.101253 | 0.170288 | 0.419116 | 0.01618 |
| ALGA0122512 | 14 | 8613934 | 0.0204 | 0.822794 | 0.193651 | 0.039576 | 0.771395 | 0.124251 | 0.074803 | 0.667783 | 0.739186 | 0.499279 | 0.935818 | 0.38458 |
| MARC0045606 | 8 | 1.02E+08 | 0.020542 | 0.739852 | 0.2148 | 0.556694 | 0.285279 | 0.417558 | 0.103781 | 0.455799 | 0.704516 | 0.735246 | 0.781472 | 0.010017 |
| ASGA0066474 | 14 | 1.33E+08 | 0.020567 | 0.463146 | 0.204608 | 0.134584 | 0.368289 | 0.919356 | 0.540122 | 0.311005 | 0.687422 | 0.280376 | 0.094361 | 0.622541 |
| H3GA0050660 | 18 | 27862609 | 0.020641 | 0.494303 | 0.246025 | 0.180502 | 0.994401 | 0.593263 | 0.239933 | 0.949475 | 0.483937 | 0.755844 | 0.706694 | 0.688631 |
| M1GA0015084 | 11 | 23769494 | 0.020665 | 0.808591 | 0.971794 | 0.032115 | 0.016894 | 0.248868 | 0.013828 | 0.126479 | 0.741803 | 0.042575 | 0.367503 | 0.095527 |
| ASGA0054485 | 0 | 0 | 0.020692 | 0.780483 | 0.241784 | 0.816827 | 0.00172 | 0.040658 | 0.606349 | 0.000297 | 0.12272 | 0.61484 | 0.200353 | 0.011682 |
| ALGA0077530 | 14 | 52878916 | 0.020701 | 0.510207 | 0.116617 | 0.779822 | 0.054543 | 0.262873 | 0.100403 | 0.00812 | 0.786793 | 0.957296 | 0.307819 | 0.729713 |
| ALGA0078226 | 14 | 66148715 | 0.020712 | 0.767569 | 0.164111 | 0.933322 | 0.004772 | 0.20704 | 0.454019 | 0.102483 | 0.347517 | 0.468015 | 0.448009 | 0.364411 |
| H3GA0012131 | 4 | 16139302 | 0.02074 | 0.872896 | 0.729851 | 0.53038 | 0.030195 | 0.837426 | 0.563946 | 0.555179 | 0.356348 | 0.991552 | 0.818434 | 0.145316 |
| ASGA0052069 | 11 | 82350055 | 0.020749 | 0.651993 | 0.474352 | 0.088869 | 0.16347 | 0.221113 | 0.00504 | 0.199755 | 0.479794 | 0.591649 | 0.499027 | 0.133376 |
| ALGA0000557 | 1 | 7814251 | 0.020774 | 0.808116 | 0.056648 | 0.070217 | 0.086321 | 0.662523 | 0.413916 | 0.705744 | 0.997862 | 0.517698 | 0.334549 | 0.28026 |
| MARC0076988 | 6 | 79836472 | 0.020829 | 0.666238 | 0.167678 | 0.051232 | 0.1609 | 0.293129 | 0.465084 | 0.070487 | 0.858192 | 0.691243 | 0.664592 | 0.18622 |
| DIAS0002202 | 17 | 13038588 | 0.020836 | 0.439 | 0.057397 | 0.428333 | 0.077675 | 0.693138 | 0.316652 | 0.985339 | 0.504488 | 0.479027 | 0.865251 | 0.391644 |
| ALGA0023479 | 4 | 15411782 | 0.02089 | 0.13201 | 0.159693 | 0.840686 | 0.092344 | 0.283493 | 0.575686 | 0.75459 | 0.030479 | 0.977975 | 0.765612 | 0.177556 |
| MARC0038322 | 2 | 1.44E+08 | 0.0209 | 0.744074 | 0.152252 | 0.199325 | 0.174821 | 0.49558 | 0.219275 | 0.576881 | 0.699211 | 0.073097 | 0.319503 | 0.020721 |
| ALGA0115991 | 6 | 1.33E+08 | 0.020909 | 0.70416 | 0.096521 | 0.023473 | 0.780568 | 0.093259 | 0.427218 | 0.825371 | 0.647352 | 0.590232 | 0.527799 | 0.514846 |
| MARC0086054 | 0 | 0 | 0.020918 | 0.329303 | 0.151501 | 0.000751 | 0.365321 | 0.027243 | 0.144656 | 0.168719 | 0.323278 | 0.728032 | 0.667614 | 0.343991 |
| ALGA0028649 | 4 | 1.29E+08 | 0.020953 | 0.998444 | 0.055969 | 0.172444 | 0.753227 | 0.787117 | 0.584512 | 0.533973 | 0.393351 | 0.557471 | 0.483684 | 0.025796 |
| H3GA0027806 | 9 | 82601158 | 0.020965 | 0.212907 | 0.083757 | 0.002476 | 0.101473 | 0.59368 | 0.185105 | 0.956996 | 0.625905 | 0.25751 | 0.746876 | 0.123501 |
| ALGA0111421 | 11 | 3450801 | 0.021085 | 0.124719 | 0.32732 | 0.035548 | 0.756292 | 0.251942 | 0.697807 | 0.863129 | 0.428439 | 0.390349 | 0.160572 | 0.308184 |
| ASGA0103125 | 0 | 0 | 0.021108 | 0.595943 | 0.140405 | 0.857516 | 0.8798 | 0.116076 | 0.953844 | 0.846351 | 0.360893 | 0.138487 | 0.044681 | 0.427594 |
| ASGA0106167 | 10 | 31302650 | 0.02114 | 0.955827 | 0.168222 | 0.53217 | 0.141432 | 0.964176 | 0.85336 | 0.170587 | 0.385632 | 0.821885 | 0.739213 | 0.165007 |
| H3GA0017081 | 5 | 93878862 | 0.021151 | 0.366591 | 0.171788 | 0.876747 | 0.224328 | 0.67184 | 0.680629 | 0.694243 | 0.152526 | 0.783028 | 0.645005 | 0.051586 |
| ALGA0051647 | 9 | 16108063 | 0.021152 | 0.573438 | 0.108946 | 0.641475 | 0.240069 | 0.594858 | 0.952196 | 0.067418 | 0.16992 | 0.632074 | 0.487483 | 0.268404 |
| MARC0001829 | 14 | 90431975 | 0.021153 | 0.083008 | 0.105084 | 0.132419 | 0.001418 | 0.864788 | 0.950385 | 0.309799 | 0.398725 | 0.20002 | 0.757276 | 0.297003 |
| ALGA0035084 | 6 | 29509178 | 0.021155 | 0.673348 | 0.011195 | 0.486075 | 0.145308 | 0.517607 | 0.65305 | 0.276602 | 0.380054 | 0.304714 | 0.877424 | 0.220077 |
| H3GA0001315 | 1 | 33735482 | 0.021175 | 0.227634 | 0.048165 | 0.781106 | 0.652808 | 0.603574 | 0.824751 | 0.100704 | 0.964858 | 0.223795 | 0.362317 | 0.161224 |
| INRA0019954 | 5 | 75287664 | 0.021229 | 0.988012 | 0.073292 | 0.064193 | 0.999651 | 0.441747 | 0.309889 | 0.844916 | 0.932387 | 0.990081 | 0.934366 | 0.114651 |
| ALGA0061341 | 11 | 22033990 | 0.021244 | 0.541417 | 0.364057 | 0.023725 | 0.107719 | 0.869595 | 0.34389 | 0.229859 | 0.787671 | 0.369246 | 0.191699 | 0.199577 |
| H3GA0025193 | 8 | 1.01E+08 | 0.021263 | 0.853279 | 0.218774 | 0.543749 | 0.266462 | 0.433473 | 0.064016 | 0.377252 | 0.762493 | 0.566223 | 0.669967 | 0.029601 |
| ALGA0021307 | 3 | 1.28E+08 | 0.0213 | 0.894448 | 0.20987 | 0.922653 | 0.253075 | 0.424001 | 0.343122 | 0.491621 | 0.301945 | 0.211682 | 0.054809 | 0.338121 |
| ASGA0105911 | 9 | 72403297 | 0.021317 | 0.706949 | 0.069803 | 0.000706 | 0.662943 | 0.010292 | 0.201891 | 0.217561 | 0.253642 | 0.552443 | 0.942299 | 0.054108 |
| M1GA0017697 | 13 | 1.48E+08 | 0.02137 | 0.824815 | 0.368764 | 0.018101 | 0.307301 | 0.981953 | 0.090485 | 0.136135 | 0.558674 | 0.626756 | 0.770034 | 0.120505 |
| ASGA0100497 | 12 | 61074050 | 0.021389 | 0.0578 | 2.37E-05 | 0.383455 | 0.07218 | 0.237085 | 0.357585 | 0.000619 | 0.206605 | 0.85304 | 0.001837 | 0.022279 |
| ASGA0012834 | 2 | 1.57E+08 | 0.021487 | 0.258156 | 0.188795 | 0.249343 | 0.635793 | 0.937959 | 0.43348 | 0.267789 | 0.925796 | 0.539127 | 0.477664 | 0.146746 |
| DRGA0011741 | 12 | 36663335 | 0.021514 | 0.519153 | 0.070678 | 0.856337 | 0.158915 | 0.138237 | 0.248701 | 0.013145 | 0.325491 | 0.306463 | 0.049995 | 0.093002 |
| ALGA0117693 | 6 | 1.49E+08 | 0.02154 | 0.62667 | 0.518806 | 0.17703 | 0.060153 | 0.913191 | 0.527995 | 0.292571 | 0.746781 | 0.898087 | 0.843934 | 0.240694 |
| ASGA0063141 | 14 | 47196197 | 0.021596 | 0.684301 | 0.175494 | 0.916217 | 0.0122 | 0.53698 | 0.13551 | 0.003312 | 0.963901 | 0.454805 | 0.243554 | 0.829825 |
| DRGA0005442 | 5 | 5816856 | 0.021612 | 0.367839 | 0.112029 | 0.796386 | 0.588243 | 0.281303 | 0.679931 | 0.91001 | 0.23476 | 0.346759 | 0.037652 | 0.779071 |
| H3GA0043424 | 14 | 1.51E+08 | 0.021612 | 0.619592 | 0.040143 | 0.062215 | 0.068134 | 0.94403 | 0.608981 | 0.998291 | 0.570044 | 0.398849 | 0.640517 | 0.599188 |
| H3GA0015764 | 5 | 14161325 | 0.02165 | 0.538494 | 0.386414 | 0.809223 | 0.995564 | 0.083155 | 0.283395 | 0.573207 | 0.344019 | 0.016702 | 0.020272 | 0.192326 |
| H3GA0039542 | 14 | 28412152 | 0.021716 | 0.551218 | 0.026452 | 0.202925 | 0.473623 | 0.471917 | 0.079779 | 0.086451 | 0.872936 | 0.29677 | 0.415925 | 0.480086 |
| ALGA0039819 | 7 | 30062235 | 0.021728 | 0.450166 | 0.13564 | 0.017561 | 0.445423 | 0.668735 | 0.478009 | 0.733422 | 0.93898 | 0.781827 | 0.277341 | 0.341775 |
| ALGA0042164 | 7 | 58806078 | 0.021751 | 0.039533 | 0.02801 | 0.126112 | 0.083927 | 0.313715 | 0.663133 | 0.201899 | 0.216136 | 0.671747 | 0.965857 | 0.013477 |
| ASGA0082570 | 12 | 39922366 | 0.021808 | 0.786472 | 0.096521 | 0.688166 | 0.018206 | 0.067231 | 0.240043 | 0.001309 | 0.086449 | 0.738882 | 0.005508 | 0.205434 |
| ALGA0119620 | 12 | 60223854 | 0.021814 | 0.412243 | 0.04501 | 0.247459 | 0.069442 | 0.280341 | 0.925813 | 0.881988 | 0.389319 | 0.476238 | 0.161184 | 0.228921 |
| ALGA0111463 | 0 | 0 | 0.02183 | 0.211162 | 0.819016 | 0.151368 | 0.18187 | 0.50952 | 0.215603 | 0.742235 | 0.183151 | 0.198394 | 0.544355 | 0.460577 |
| MARC0061822 | 10 | 31937937 | 0.021838 | 0.276095 | 0.267939 | 0.609249 | 0.029904 | 0.710544 | 0.45177 | 0.12257 | 0.080094 | 0.197917 | 0.998448 | 0.339632 |
| ASGA0099886 | 12 | 59480153 | 0.021841 | 0.625924 | 0.36194 | 0.352894 | 0.094556 | 0.32664 | 0.07308 | 0.01786 | 0.812371 | 0.262737 | 0.826749 | 0.092679 |
| MARC0012307 | 10 | 18821110 | 0.021861 | 0.912605 | 0.03748 | 0.205488 | 0.242622 | 0.46634 | 0.277284 | 0.501587 | 0.968815 | 0.804922 | 0.922119 | 0.368811 |
| ASGA0073294 | 16 | 48994238 | 0.021876 | 0.296635 | 0.121496 | 0.235502 | 0.023702 | 0.042081 | 0.859947 | 0.197445 | 0.189199 | 0.551258 | 0.269954 | 0.537202 |
| ALGA0097733 | 18 | 30380948 | 0.021878 | 0.390059 | 0.510374 | 0.179627 | 0.122259 | 0.497938 | 0.299325 | 0.798051 | 0.792719 | 0.655657 | 0.679629 | 0.240702 |
| ALGA0080216 | 14 | 1.07E+08 | 0.021891 | 0.465585 | 0.073298 | 0.144419 | 0.049952 | 0.791368 | 0.99156 | 0.344716 | 0.755891 | 0.725554 | 0.075652 | 0.99533 |
| ASGA0037172 | 7 | 1.3E+08 | 0.021902 | 0.544356 | 0.114681 | 0.381778 | 0.047699 | 0.834457 | 0.704821 | 0.185523 | 0.59314 | 0.643692 | 0.933856 | 0.358995 |
| ASGA0025495 | 5 | 46692142 | 0.021926 | 0.11555 | 0.066686 | 0.373543 | 0.449655 | 0.549115 | 0.13086 | 0.416861 | 0.903932 | 0.388173 | 0.65403 | 0.032324 |
| ALGA0069574 | 13 | 38221795 | 0.021972 | 0.431589 | 0.189255 | 0.779042 | 0.855672 | 0.754045 | 0.946321 | 0.743131 | 0.620929 | 0.438846 | 0.724491 | 0.000586 |
| ASGA0059227 | 13 | 1.66E+08 | 0.021995 | 0.271678 | 0.441381 | 0.357871 | 0.05588 | 0.557099 | 0.376258 | 0.098005 | 0.082669 | 0.530973 | 0.423847 | 0.436738 |
| ALGA0100550 | 6 | 10082573 | 0.022026 | 0.604514 | 0.022158 | 0.150233 | 0.776407 | 0.936858 | 0.943494 | 0.328699 | 0.741363 | 0.573866 | 0.059276 | 0.465234 |
| ASGA0076027 | 17 | 31459408 | 0.022036 | 0.326074 | 0.183544 | 0.155941 | 0.020356 | 0.196989 | 0.941298 | 0.371058 | 0.464699 | 0.432399 | 0.041676 | 0.056909 |
| ALGA0121161 | 1 | 8770199 | 0.02208 | 0.724181 | 0.382584 | 0.365612 | 0.036053 | 0.522212 | 0.987276 | 0.777978 | 0.242016 | 0.894067 | 0.540175 | 0.786871 |
| ALGA0122535 | 13 | 19859830 | 0.022106 | 0.791611 | 0.200508 | 0.344129 | 0.357164 | 0.736112 | 0.232847 | 0.649395 | 0.702507 | 0.262772 | 0.559987 | 0.198612 |
| ASGA0055250 | 12 | 58093644 | 0.022128 | 0.88844 | 0.696064 | 0.316284 | 0.092513 | 0.463373 | 0.046727 | 0.442651 | 0.888331 | 0.241745 | 0.487335 | 0.230877 |
| M1GA0015353 | 11 | 81053745 | 0.022166 | 0.895766 | 0.009808 | 0.362742 | 0.480252 | 0.770491 | 0.848456 | 0.948858 | 0.303229 | 0.595552 | 0.769836 | 0.363076 |
| ASGA0101987 | 0 | 0 | 0.022179 | 0.796071 | 0.114494 | 0.125241 | 0.953402 | 0.24375 | 0.257637 | 0.966184 | 0.424054 | 0.970872 | 0.905285 | 0.045339 |
| H3GA0041549 | 14 | 1E+08 | 0.022219 | 0.43732 | 0.035341 | 0.734172 | 0.235684 | 0.686356 | 0.947854 | 0.671766 | 0.439885 | 0.507234 | 0.197431 | 0.440559 |
| ASGA0037025 | 7 | 1.29E+08 | 0.022269 | 0.355689 | 0.012864 | 0.066518 | 0.114504 | 0.731211 | 0.434732 | 0.982062 | 0.938508 | 0.963462 | 0.72868 | 0.275958 |
| MARC0026903 | 4 | 22012168 | 0.022273 | 0.612644 | 0.18835 | 0.403784 | 0.074189 | 0.64157 | 0.423963 | 0.818071 | 0.652059 | 0.667948 | 0.550916 | 0.089826 |
| ASGA0058041 | 13 | 75539907 | 0.022292 | 0.117265 | 0.143871 | 0.59512 | 0.459158 | 0.25257 | 0.431105 | 0.7008 | 0.750851 | 0.719447 | 0.258406 | 0.039343 |
| DIAS0001965 | 13 | 2.07E+08 | 0.02237 | 0.946552 | 0.228569 | 0.179251 | 0.153308 | 0.842782 | 0.522871 | 0.330009 | 0.832948 | 0.750976 | 0.737919 | 0.361039 |
| MARC0004327 | 9 | 29540228 | 0.022375 | 0.488887 | 0.140764 | 0.223942 | 0.377681 | 0.174179 | 0.089622 | 0.295687 | 0.008694 | 0.469175 | 0.178533 | 0.212144 |
| ALGA0110526 | 0 | 0 | 0.022455 | 0.259221 | 0.300216 | 0.064388 | 0.199628 | 0.565396 | 0.141157 | 0.073085 | 0.419146 | 0.163013 | 0.95941 | 0.247508 |
| ASGA0000601 | 1 | 7836573 | 0.022459 | 0.885968 | 0.077977 | 0.132602 | 0.100654 | 0.792351 | 0.504371 | 0.71344 | 0.83605 | 0.518218 | 0.407789 | 0.345645 |
| H3GA0043694 | 15 | 8749939 | 0.022537 | 0.518012 | 0.292175 | 0.049186 | 0.139064 | 0.70276 | 0.452008 | 0.240289 | 0.355891 | 0.867013 | 0.818435 | 0.560544 |
| DRGA0015844 | 16 | 11509437 | 0.022581 | 0.906745 | 0.381994 | 0.220175 | 0.010294 | 0.577732 | 0.221254 | 0.052558 | 0.457152 | 0.664901 | 0.904183 | 0.078639 |
| H3GA0037233 | 13 | 1.33E+08 | 0.022592 | 0.275699 | 0.505348 | 0.283347 | 0.245593 | 0.672977 | 0.158731 | 0.164895 | 0.963584 | 0.900539 | 0.741966 | 0.10183 |
| ALGA0035318 | 6 | 51678926 | 0.022605 | 0.798963 | 0.063102 | 0.03367 | 0.093117 | 0.969338 | 0.932929 | 0.226949 | 0.160545 | 0.306946 | 0.018939 | 0.756099 |
| ALGA0035323 | 6 | 51611976 | 0.022605 | 0.798963 | 0.063102 | 0.03367 | 0.093117 | 0.969338 | 0.932929 | 0.226949 | 0.160545 | 0.306946 | 0.018939 | 0.756099 |
| ALGA0035324 | 6 | 51636474 | 0.022605 | 0.798963 | 0.063102 | 0.03367 | 0.093117 | 0.969338 | 0.932929 | 0.226949 | 0.160545 | 0.306946 | 0.018939 | 0.756099 |
| ASGA0047214 | 10 | 30481283 | 0.022626 | 0.936524 | 0.029997 | 0.454942 | 0.056028 | 0.845775 | 0.734827 | 0.241337 | 0.083293 | 0.752832 | 0.410648 | 0.304962 |
| ASGA0063397 | 14 | 55036020 | 0.022649 | 0.571721 | 0.085917 | 0.555797 | 0.058686 | 0.552018 | 0.582832 | 0.04396 | 0.957377 | 0.82102 | 0.071965 | 0.796942 |
| ASGA0063398 | 14 | 55057069 | 0.022649 | 0.571721 | 0.085917 | 0.555797 | 0.058686 | 0.552018 | 0.582832 | 0.04396 | 0.957377 | 0.82102 | 0.071965 | 0.796942 |
| DIAS0002207 | 8 | 1.1E+08 | 0.022711 | 0.888308 | 0.251605 | 0.166487 | 0.821274 | 0.750514 | 0.260981 | 0.656645 | 0.404648 | 0.599558 | 0.860119 | 0.01241 |
| ALGA0117560 | 6 | 72740781 | 0.022731 | 0.134874 | 0.048422 | 0.070639 | 0.040066 | 0.462752 | 0.729866 | 0.008751 | 0.478156 | 0.552605 | 0.25809 | 0.106979 |
| ALGA0124017 | 8 | 7875856 | 0.022755 | 0.558316 | 0.009173 | 0.08342 | 0.78741 | 0.13275 | 0.176935 | 0.745082 | 0.201844 | 0.489747 | 0.94477 | 0.135776 |
| M1GA0018651 | 14 | 52903091 | 0.022768 | 0.439192 | 0.150533 | 0.699638 | 0.043271 | 0.187978 | 0.135775 | 0.006488 | 0.670327 | 0.780471 | 0.213929 | 0.655454 |
| INRA0044164 | 14 | 58984594 | 0.022778 | 0.608051 | 0.092311 | 0.732225 | 0.014759 | 0.205932 | 0.405737 | 0.126071 | 0.745947 | 0.450038 | 0.186794 | 0.600767 |
| MARC0109449 | 12 | 40101831 | 0.022796 | 0.885959 | 0.300174 | 0.64413 | 0.009119 | 0.011918 | 0.375992 | 0.001003 | 0.064944 | 0.378362 | 0.189342 | 0.059771 |
| ASGA0023202 | 4 | 1.38E+08 | 0.0228 | 0.395705 | 0.027658 | 0.001158 | 0.975639 | 0.027357 | 0.075428 | 0.386267 | 0.074903 | 0.711259 | 0.713402 | 0.165867 |
| DIAS0000319 | 18 | 30296116 | 0.022832 | 0.31868 | 0.460791 | 0.153362 | 0.14253 | 0.563152 | 0.280828 | 0.91796 | 0.858344 | 0.271393 | 0.577636 | 0.211763 |
| ALGA0036849 | 6 | 1.26E+08 | 0.022873 | 0.687641 | 0.015587 | 0.073727 | 0.106036 | 0.294957 | 0.996883 | 0.94396 | 0.432585 | 0.68432 | 0.310752 | 0.265072 |
| MARC0040328 | 0 | 0 | 0.022894 | 0.403783 | 0.041462 | 0.533781 | 0.065928 | 0.530198 | 0.548342 | 0.242613 | 0.079373 | 0.391441 | 0.169144 | 0.175689 |
| ALGA0090447 | 16 | 41985204 | 0.022902 | 0.025355 | 0.202106 | 0.135761 | 0.039916 | 0.678514 | 0.559021 | 0.63536 | 0.209365 | 0.989623 | 0.644356 | 0.355383 |
| DRGA0005264 | 4 | 1.3E+08 | 0.022915 | 0.906505 | 0.092028 | 0.141486 | 0.577954 | 0.219315 | 0.35548 | 0.797274 | 0.255432 | 0.98324 | 0.746407 | 0.027277 |
| DRGA0017433 | 0 | 0 | 0.022935 | 0.127529 | 0.034145 | 0.13408 | 0.146175 | 0.803881 | 0.65182 | 0.213423 | 0.163919 | 0.100954 | 0.047531 | 0.323633 |
| ASGA0100644 | 0 | 0 | 0.022958 | 0.189175 | 0.812367 | 0.149724 | 0.145689 | 0.421914 | 0.178524 | 0.612637 | 0.140241 | 0.272426 | 0.546512 | 0.730007 |
| ALGA0061167 | 11 | 18628696 | 0.022982 | 0.672474 | 0.192189 | 0.036916 | 0.58887 | 0.230804 | 0.690348 | 0.946747 | 0.298146 | 0.909166 | 0.411531 | 0.633402 |
| MARC0103954 | 0 | 0 | 0.022985 | 0.131519 | 0.210258 | 0.225566 | 0.103585 | 0.756701 | 0.748552 | 0.124112 | 0.259601 | 0.306467 | 0.269397 | 0.800155 |
| ALGA0105488 | 8 | 1.21E+08 | 0.023019 | 0.955999 | 0.311089 | 0.204678 | 0.768501 | 0.861811 | 0.053467 | 0.682179 | 0.611275 | 0.54369 | 0.451747 | 0.431304 |
| ASGA0057028 | 13 | 31608940 | 0.023027 | 0.835461 | 0.200854 | 0.954699 | 0.656526 | 0.453723 | 0.999169 | 0.573771 | 0.578378 | 0.943565 | 0.34459 | 0.008989 |
| ALGA0062571 | 11 | 63898071 | 0.023041 | 0.260339 | 0.125688 | 0.000361 | 0.524353 | 0.2341 | 0.418551 | 0.666429 | 0.724178 | 0.838875 | 0.152214 | 0.92985 |
| ASGA0066239 | 14 | 1.25E+08 | 0.023058 | 0.133831 | 0.034439 | 0.801658 | 0.435209 | 0.634021 | 0.42121 | 0.113002 | 0.235359 | 0.450927 | 0.283782 | 0.009048 |
| MARC0069139 | 9 | 96887177 | 0.023073 | 0.763227 | 0.057768 | 0.311708 | 0.304833 | 0.38756 | 0.226673 | 0.684779 | 0.096658 | 0.980368 | 0.057396 | 0.474376 |
| ALGA0078256 | 0 | 0 | 0.02309 | 0.863425 | 0.080862 | 0.604446 | 0.015214 | 0.2634 | 0.56444 | 0.166389 | 0.635166 | 0.563159 | 0.182982 | 0.693654 |
| DIAS0002445 | 14 | 66479026 | 0.02309 | 0.863425 | 0.080862 | 0.604446 | 0.015214 | 0.2634 | 0.56444 | 0.166389 | 0.635166 | 0.563159 | 0.182982 | 0.693654 |
| ALGA0113336 | 6 | 1.28E+08 | 0.023091 | 0.395451 | 0.058862 | 0.24049 | 0.102391 | 0.779648 | 0.869798 | 0.356816 | 0.181452 | 0.243759 | 0.164214 | 0.348588 |
| ALGA0088449 | 15 | 1.57E+08 | 0.023157 | 0.733673 | 0.036161 | 0.159047 | 0.436241 | 0.92839 | 0.249127 | 0.680027 | 0.772112 | 0.317202 | 0.621929 | 0.097025 |
| MARC0083766 | 6 | 1.27E+08 | 0.023186 | 0.490161 | 0.084821 | 0.164715 | 0.807354 | 0.785386 | 0.388762 | 0.327804 | 0.575551 | 0.110251 | 0.316495 | 0.964994 |
| H3GA0019180 | 6 | 1.47E+08 | 0.023195 | 0.785966 | 0.519609 | 0.04294 | 0.03592 | 0.799079 | 0.295027 | 0.307486 | 0.895607 | 0.727883 | 0.739194 | 0.376298 |
| MARC0016323 | 12 | 60428948 | 0.023236 | 0.648987 | 0.007281 | 0.234196 | 0.013449 | 0.113212 | 0.196926 | 0.053662 | 0.039333 | 0.29903 | 0.013344 | 0.055551 |
| INRA0051258 | 16 | 23853929 | 0.02325 | 0.542581 | 0.035161 | 0.337697 | 0.280584 | 0.483032 | 0.889607 | 0.77454 | 0.669391 | 0.223745 | 0.033631 | 0.123007 |
| ALGA0119811 | 6 | 73201165 | 0.023265 | 0.150262 | 0.044207 | 0.072983 | 0.039605 | 0.427124 | 0.764799 | 0.007933 | 0.434779 | 0.450451 | 0.28437 | 0.096507 |
| MARC0105202 | 1 | 31113659 | 0.02329 | 0.057756 | 0.05914 | 0.777852 | 0.525203 | 0.667308 | 0.296333 | 0.037827 | 0.935874 | 0.256809 | 0.803798 | 0.320796 |
| H3GA0017058 | 5 | 92073891 | 0.023308 | 0.489946 | 0.122882 | 0.083275 | 0.182694 | 0.820729 | 0.87946 | 0.170292 | 0.402396 | 0.907802 | 0.388254 | 0.090218 |
| SIRI0000575 | 14 | 90511729 | 0.023321 | 0.061406 | 0.110523 | 0.112257 | 0.001105 | 0.827366 | 0.811403 | 0.312909 | 0.311417 | 0.1296 | 0.751041 | 0.262944 |
| DRGA0013104 | 13 | 1.81E+08 | 0.023338 | 0.671316 | 0.281092 | 0.571927 | 0.034316 | 0.812324 | 0.124463 | 0.006869 | 0.212383 | 0.238781 | 0.792859 | 0.066656 |
| MARC0051601 | 8 | 1.21E+08 | 0.023345 | 0.985956 | 0.308508 | 0.240894 | 0.763219 | 0.852368 | 0.051721 | 0.670126 | 0.633212 | 0.545616 | 0.481838 | 0.418939 |
| ALGA0085969 | 15 | 86123500 | 0.023388 | 0.469906 | 0.076895 | 0.004274 | 0.371241 | 0.104609 | 0.093427 | 0.855668 | 0.202124 | 0.571731 | 0.837618 | 0.641909 |
| ASGA0025505 | 5 | 49588554 | 0.023403 | 0.799007 | 0.154503 | 0.16147 | 0.437086 | 0.702617 | 0.245774 | 0.785455 | 0.981708 | 0.132317 | 0.474835 | 0.119467 |
| DRGA0000154 | 1 | 16441812 | 0.023422 | 0.322963 | 0.066281 | 0.324497 | 0.318594 | 0.195665 | 0.8251 | 0.743453 | 0.867961 | 0.398519 | 0.306553 | 0.058 |
| ALGA0001958 | 1 | 30382543 | 0.023437 | 0.176951 | 0.058063 | 0.221531 | 0.587371 | 0.289853 | 0.105899 | 0.146067 | 0.272417 | 0.242857 | 0.070171 | 0.015495 |
| ALGA0107710 | 6 | 81060607 | 0.023442 | 0.215748 | 0.076803 | 0.122803 | 0.097832 | 0.823154 | 0.560505 | 0.353427 | 0.059495 | 0.15735 | 0.016503 | 0.396894 |
| ASGA0094634 | 6 | 81597202 | 0.023442 | 0.215748 | 0.076803 | 0.122803 | 0.097832 | 0.823154 | 0.560505 | 0.353427 | 0.059495 | 0.15735 | 0.016503 | 0.396894 |
| DIAS0004429 | 6 | 81553636 | 0.023442 | 0.215748 | 0.076803 | 0.122803 | 0.097832 | 0.823154 | 0.560505 | 0.353427 | 0.059495 | 0.15735 | 0.016503 | 0.396894 |
| ASGA0053372 | 12 | 14741359 | 0.023472 | 0.963427 | 0.155429 | 0.799234 | 0.178557 | 0.317109 | 0.547353 | 0.692138 | 0.964919 | 0.442289 | 0.247268 | 0.304239 |
| ALGA0077290 | 14 | 47120045 | 0.023481 | 0.456841 | 0.142416 | 0.951325 | 0.108242 | 0.398399 | 0.084196 | 0.012726 | 0.920355 | 0.837196 | 0.301218 | 0.691718 |
| ALGA0003346 | 1 | 57841633 | 0.023531 | 0.91663 | 0.032019 | 0.685336 | 0.984828 | 0.31884 | 0.894814 | 0.514603 | 0.597111 | 0.663075 | 0.148549 | 0.121018 |
| ALGA0019055 | 3 | 56277492 | 0.023538 | 0.496055 | 0.078239 | 0.565206 | 0.591734 | 0.869315 | 0.942349 | 0.844371 | 0.830399 | 0.297049 | 0.23073 | 0.55849 |
| DRGA0006105 | 5 | 86480453 | 0.023705 | 0.748914 | 0.083836 | 0.085526 | 0.094434 | 0.715381 | 0.789072 | 0.170829 | 0.372195 | 0.804866 | 0.190385 | 0.318088 |
| ASGA0056025 | 13 | 12971106 | 0.023813 | 0.768246 | 0.044578 | 0.607953 | 0.70378 | 0.997245 | 0.746075 | 0.299059 | 0.849509 | 0.41518 | 0.70311 | 0.221726 |
| MARC0027370 | 14 | 1.44E+08 | 0.023847 | 0.485885 | 0.039406 | 0.185075 | 0.224271 | 0.988867 | 0.591895 | 0.939277 | 0.468573 | 0.433628 | 0.991193 | 0.417877 |
| DRGA0014794 | 15 | 4432327 | 0.02388 | 0.969112 | 0.66707 | 0.25138 | 0.028046 | 0.821317 | 0.097186 | 0.015152 | 0.325883 | 0.501458 | 0.632825 | 0.168864 |
| ASGA0067012 | 14 | 1.41E+08 | 0.023887 | 0.868918 | 0.017637 | 0.225511 | 0.286169 | 0.783362 | 0.77916 | 0.823384 | 0.124955 | 0.451909 | 0.818822 | 0.244823 |
| MARC0098236 | 13 | 89220372 | 0.023931 | 0.25695 | 0.069875 | 0.31787 | 0.360685 | 0.184008 | 0.689449 | 0.995074 | 0.450932 | 0.645834 | 0.709169 | 0.15851 |
| ALGA0030706 | 5 | 14137895 | 0.024011 | 0.411125 | 0.260484 | 0.973199 | 0.624559 | 0.15007 | 0.103299 | 0.805317 | 0.236453 | 0.267839 | 0.044152 | 0.167645 |
| ASGA0016106 | 3 | 1.19E+08 | 0.024023 | 0.726676 | 0.083663 | 0.280651 | 0.18004 | 0.497657 | 0.547922 | 0.850697 | 0.714226 | 0.681083 | 0.678049 | 0.016747 |
| M1GA0017465 | 13 | 29861003 | 0.024129 | 0.377935 | 0.369508 | 0.636462 | 0.471733 | 0.420801 | 0.4664 | 0.275745 | 0.733582 | 0.871291 | 0.705247 | 0.005542 |
| MARC0061219 | 13 | 60124449 | 0.024148 | 0.220118 | 0.081628 | 0.001951 | 0.060271 | 0.585571 | 0.13501 | 0.780169 | 0.939236 | 0.111998 | 0.661759 | 0.129112 |
| H3GA0011208 | 0 | 0 | 0.024183 | 0.056229 | 0.131905 | 0.367761 | 0.094653 | 0.201713 | 0.625276 | 0.234909 | 0.90326 | 0.442355 | 0.209366 | 0.155272 |
| MARC0040203 | 1 | 3.05E+08 | 0.024247 | 0.685488 | 0.504926 | 0.271808 | 0.182896 | 0.811494 | 0.283162 | 0.347734 | 0.454803 | 0.960747 | 0.335199 | 0.391914 |
| ASGA0085559 | 8 | 32103030 | 0.024285 | 0.290438 | 0.096854 | 0.523176 | 0.940862 | 0.507605 | 0.743961 | 0.855581 | 0.327106 | 0.550912 | 0.394986 | 0.165726 |
| DIAS0003271 | 9 | 73652335 | 0.024314 | 0.650117 | 0.193011 | 0.009793 | 0.122427 | 0.245093 | 0.224836 | 0.846185 | 0.663365 | 0.359276 | 0.757018 | 0.207068 |
| ALGA0073288 | 13 | 1.94E+08 | 0.024331 | 0.914332 | 0.077554 | 0.359569 | 0.097384 | 0.614297 | 0.528832 | 0.076181 | 0.284752 | 0.596378 | 0.851676 | 0.120454 |
| ASGA0098019 | 13 | 13883524 | 0.024341 | 0.850976 | 0.059519 | 0.251045 | 0.587918 | 0.819952 | 0.36369 | 0.230365 | 0.739723 | 0.33238 | 0.587098 | 0.223406 |
| ALGA0111468 | 13 | 2.01E+08 | 0.024355 | 0.240242 | 0.109181 | 0.04928 | 0.176523 | 0.954052 | 0.365076 | 0.183368 | 0.979098 | 0.296827 | 0.805569 | 0.085155 |
| MARC0077442 | 7 | 29913003 | 0.024356 | 0.047827 | 0.064541 | 0.536569 | 0.384386 | 0.759614 | 0.362145 | 0.350816 | 0.991307 | 0.424845 | 0.94767 | 0.012274 |
| ALGA0063591 | 11 | 77791518 | 0.024366 | 0.16684 | 0.106245 | 0.002891 | 0.20467 | 0.3045 | 0.875445 | 0.713578 | 0.785699 | 0.751958 | 0.165459 | 0.726143 |
| ASGA0059675 | 13 | 2E+08 | 0.024381 | 0.477579 | 0.070804 | 0.199842 | 0.28263 | 0.560988 | 0.249559 | 0.011169 | 0.49339 | 0.04593 | 0.490271 | 0.016464 |
| ALGA0062332 | 11 | 55001359 | 0.024453 | 0.711484 | 0.046483 | 0.08985 | 0.163215 | 0.7718 | 0.561306 | 0.781723 | 0.659476 | 0.863462 | 0.28133 | 0.428339 |
| ALGA0110174 | 13 | 2E+08 | 0.024467 | 0.192577 | 0.065686 | 0.04023 | 0.120785 | 0.985061 | 0.372352 | 0.134796 | 0.856358 | 0.070352 | 0.951398 | 0.058166 |
| MARC0009720 | 0 | 0 | 0.024467 | 0.192577 | 0.065686 | 0.04023 | 0.120785 | 0.985061 | 0.372352 | 0.134796 | 0.856358 | 0.070352 | 0.951398 | 0.058166 |
| MARC0084798 | 13 | 2E+08 | 0.024467 | 0.192577 | 0.065686 | 0.04023 | 0.120785 | 0.985061 | 0.372352 | 0.134796 | 0.856358 | 0.070352 | 0.951398 | 0.058166 |
| UMB10000032 | 13 | 1.57E+08 | 0.024508 | 0.42656 | 0.298616 | 0.066835 | 0.054254 | 0.932554 | 0.231999 | 0.616969 | 0.592964 | 0.086718 | 0.950746 | 0.187495 |
| ASGA0043574 | 9 | 70368654 | 0.024556 | 0.63781 | 0.031016 | 0.000788 | 0.267954 | 0.777371 | 0.906266 | 0.199263 | 0.956434 | 0.174393 | 0.526742 | 0.022758 |
| MARC0098463 | 0 | 0 | 0.024573 | 0.224036 | 0.040981 | 0.596893 | 0.099401 | 0.442733 | 0.400973 | 0.1723 | 0.043302 | 0.682873 | 0.20983 | 0.215615 |
| ALGA0073434 | 13 | 2E+08 | 0.024575 | 0.497077 | 0.069364 | 0.221561 | 0.269448 | 0.580606 | 0.279126 | 0.011219 | 0.448231 | 0.04224 | 0.514883 | 0.015711 |
| ASGA0057265 | 13 | 38310437 | 0.024597 | 0.553158 | 0.191206 | 0.672924 | 0.708466 | 0.660992 | 0.973767 | 0.5823 | 0.700368 | 0.526579 | 0.694539 | 0.001691 |
| ASGA0018563 | 4 | 15524055 | 0.024628 | 0.354221 | 0.279649 | 0.490135 | 0.028921 | 0.221421 | 0.789297 | 0.336436 | 0.033431 | 0.911976 | 0.616032 | 0.564899 |
| ASGA0057312 | 13 | 40238900 | 0.024664 | 0.515823 | 0.043444 | 0.824874 | 0.957471 | 0.935158 | 0.465691 | 0.527504 | 0.912522 | 0.510736 | 0.580703 | 0.005177 |
| ALGA0114175 | 16 | 16691379 | 0.024674 | 0.705211 | 0.040486 | 0.489907 | 0.167386 | 0.54521 | 0.335476 | 0.624461 | 0.94954 | 0.086837 | 0.057905 | 0.012106 |
| MARC0006792 | 13 | 82533353 | 0.02472 | 0.333503 | 0.205727 | 0.170879 | 0.131872 | 0.388997 | 0.245931 | 0.574437 | 0.995295 | 0.778898 | 0.692026 | 0.217689 |
| ALGA0065830 | 12 | 28426018 | 0.024772 | 0.782908 | 0.128108 | 0.864718 | 0.037825 | 0.084848 | 0.213358 | 0.003148 | 0.079702 | 0.50183 | 0.067902 | 0.347463 |
| ALGA0111092 | 9 | 18015415 | 0.02479 | 0.500538 | 0.065308 | 0.39882 | 0.332073 | 0.579456 | 0.577656 | 0.482031 | 0.180176 | 0.982821 | 0.616869 | 0.119367 |
| H3GA0031494 | 11 | 18764524 | 0.024793 | 0.723816 | 0.232454 | 0.069141 | 0.626609 | 0.317857 | 0.714289 | 0.935353 | 0.28691 | 0.801167 | 0.457401 | 0.299631 |
| INRA0030289 | 8 | 1.12E+08 | 0.024808 | 0.779916 | 0.591409 | 0.182335 | 0.648273 | 0.756772 | 0.42793 | 0.637004 | 0.548567 | 0.638637 | 0.869288 | 0.025198 |
| H3GA0053266 | 6 | 71995060 | 0.024843 | 0.895351 | 0.073005 | 0.12777 | 0.165633 | 0.919235 | 0.15583 | 0.38091 | 0.369282 | 0.425845 | 0.920763 | 0.149627 |
| MARC0032599 | 10 | 42416796 | 0.024886 | 0.23695 | 0.372469 | 0.723433 | 0.010655 | 0.507392 | 0.893758 | 0.367044 | 0.08032 | 0.253871 | 0.975836 | 0.074213 |
| ALGA0077232 | 14 | 45850137 | 0.024941 | 0.402633 | 0.097172 | 0.825131 | 0.064447 | 0.300367 | 0.545032 | 0.377443 | 0.369476 | 0.320556 | 0.835319 | 0.410592 |
| MARC0045269 | 9 | 1.03E+08 | 0.024979 | 0.327572 | 0.109767 | 0.036449 | 0.505427 | 0.386265 | 0.210008 | 0.763652 | 0.320738 | 0.064637 | 0.340305 | 0.030205 |
| ALGA0112022 | 0 | 0 | 0.024983 | 0.365657 | 0.054384 | 0.46987 | 0.086812 | 0.623308 | 0.691542 | 0.273894 | 0.125314 | 0.375785 | 0.186489 | 0.105815 |
| ALGA0119127 | 12 | 60230338 | 0.02499 | 0.251734 | 0.020272 | 0.838467 | 0.394009 | 0.591794 | 0.604032 | 0.066548 | 0.94207 | 0.395181 | 0.674677 | 0.001305 |
| ASGA0095878 | 13 | 2.07E+08 | 0.02501 | 0.784969 | 0.026007 | 0.08201 | 0.059019 | 0.847985 | 0.489564 | 0.166713 | 0.514114 | 0.747109 | 0.679405 | 0.118219 |
